# Conformational Control of [2]Rotaxane by Hydrogen Bond

**DOI:** 10.1021/acs.joc.2c00086

**Published:** 2022-04-07

**Authors:** Yusuke Kawasaki, Showkat Rashid, Katsuhiko Ikeyatsu, Yuichiro Mutoh, Yusuke Yoshigoe, Shoko Kikkawa, Isao Azumaya, Shoichi Hosoya, Shinichi Saito

**Affiliations:** † Department of Chemistry, Faculty of Science, Tokyo University of Science, 1-3 Kagurazaka, Shinjuku, Tokyo 162-8601, Japan; ‡ Faculty of Pharmaceutical Sciences, Toho University, 2-2-1 Miyama, Funabashi, Chiba 274-8510, Japan; § Research Center for Medical and Dental Sciences, Tokyo Medical and Dental University, 1-5-45 Yushima, Bunkyo-ku, Tokyo 113-8510, Japan

## Abstract

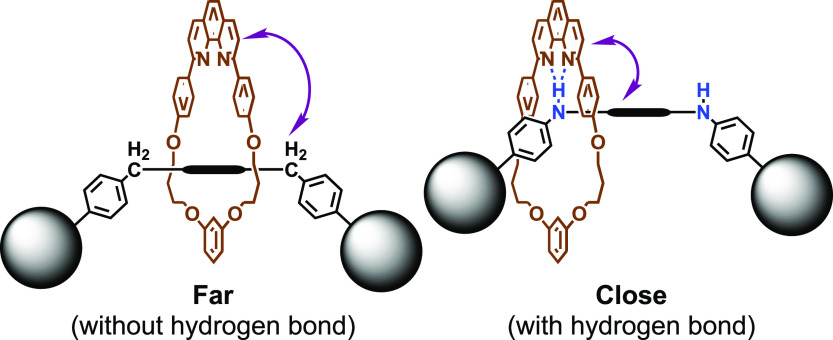

A series of [2]rotaxanes with various functional groups in the axle component was synthesized by the oxidative dimerization of alkynes, which is mediated by a macrocyclic phenanthroline–Cu complex. The rotaxanes were fully characterized by spectroscopic methods, and the structure of a rotaxane was determined by X-ray crystallographic analysis. The interaction between the ring component and the axle component was studied in detail to understand the conformation of the rotaxanes. The presence of the hydrogen bond between the phenanthroline moiety in the macrocyclic component and the acidic proton in the axle component influenced the conformation of rotaxane.

## Introduction

[2]Rotaxane is an important class of interlocked compounds, and extensive studies related to the synthesis, structure, and dynamic behavior have been reported.^1^ Following the seminal study of Dietrich–Buchecker and Sauvage, who reported the synthesis of [2]catenates from macrocyclic phenanthrolines by the metal-template method,^2^ Gibson and co-workers reported the synthesis of [2]rotaxane based on a similar strategy.^3^ These synthetic approaches were extensively applied to the synthesis of various interlocked compounds.^4^

**Scheme 1:**
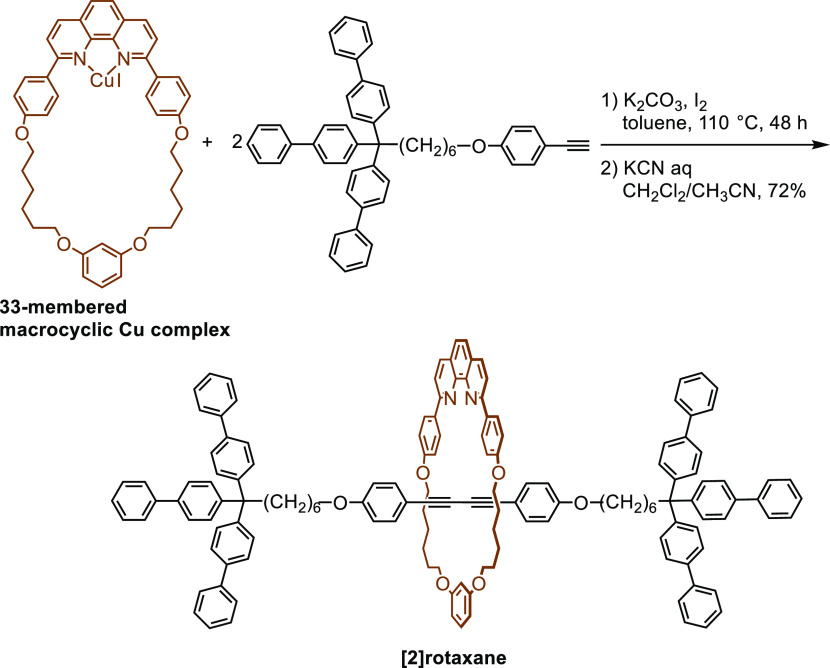
Synthesis of [2]Rotaxane by Glaser Coupling^7a^

Recent development of the synthetic methods related to interlocked compounds includes the use of a macrocyclic metal complex as a promotor for the bond-forming reaction. The metal-mediated reaction proceeded inside the macrocyclic metal complex so that the interlocked compounds could be synthesized efficiently.^5^ Leigh and co-workers reported the first example of this approach, who employed a macrocyclic pyridine–Cu complex.^6^ Assuming that the Cu complex could mediate coupling reactions such as the oxidative dimerization of alkynes (Glaser coupling), we reported the synthesis of [2]rotaxanes from macrocyclic phenanthroline–Cu complex and alkynes with bulky substituents (Scheme 1).^7^ Interlocked compounds with polyyne structures have been synthesized by this method, and the properties of these compounds have been studied by several research groups.^8^

**Scheme 2:**
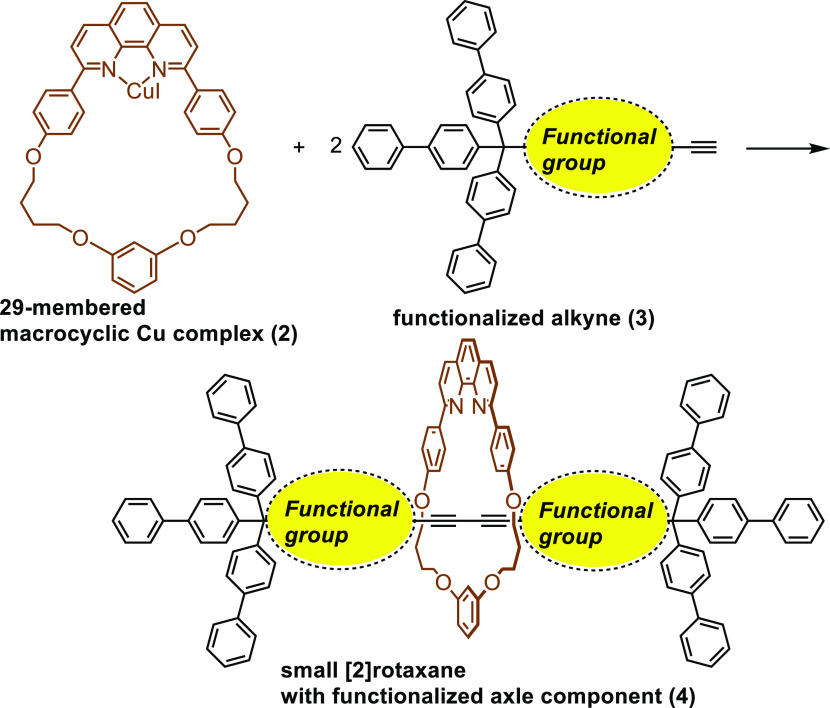
Synthesis of Small [2]Rotaxanes with a Functionalized Axle Component

We have been interested in the conformation of [2]rotaxane with a macrocyclic phenanthroline ring. The phenanthroline moiety of the ring component would interact with the acidic hydrogen atom located in the axle component and the conformation of the [2]rotaxane could be affected, especially when the size of the ring component is small.^9^ In this paper, we report the synthesis of small [2]rotaxanes with functionalized axle components (Scheme 2). The interaction between the ring and axle components was studied to understand the conformation of [2]rotaxanes.

## Results and Discussion

### Synthesis of the Precursors for [2]Rotaxane

**Scheme 3:**
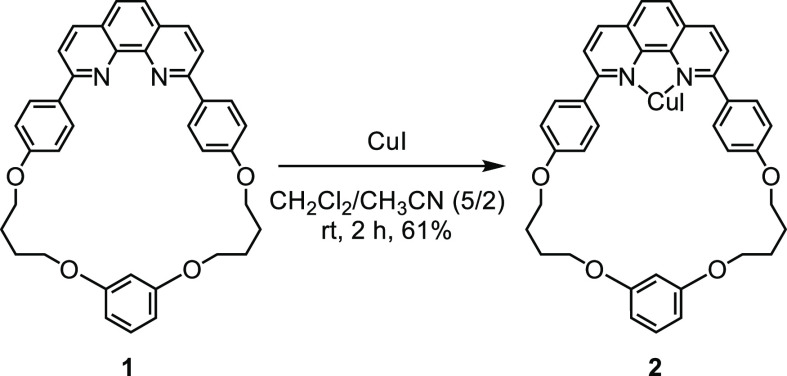
Synthesis of Macrocyclic Cu(I)–Phenanthroline Complex **2**

A macrocyclic phenanthroline–Cu complex (**2**) was synthesized by the reaction of **1**(8f) with CuI (Scheme 3). The reaction proceeded smoothly, and **2** was isolated in 61% yield.

**Scheme 4:**
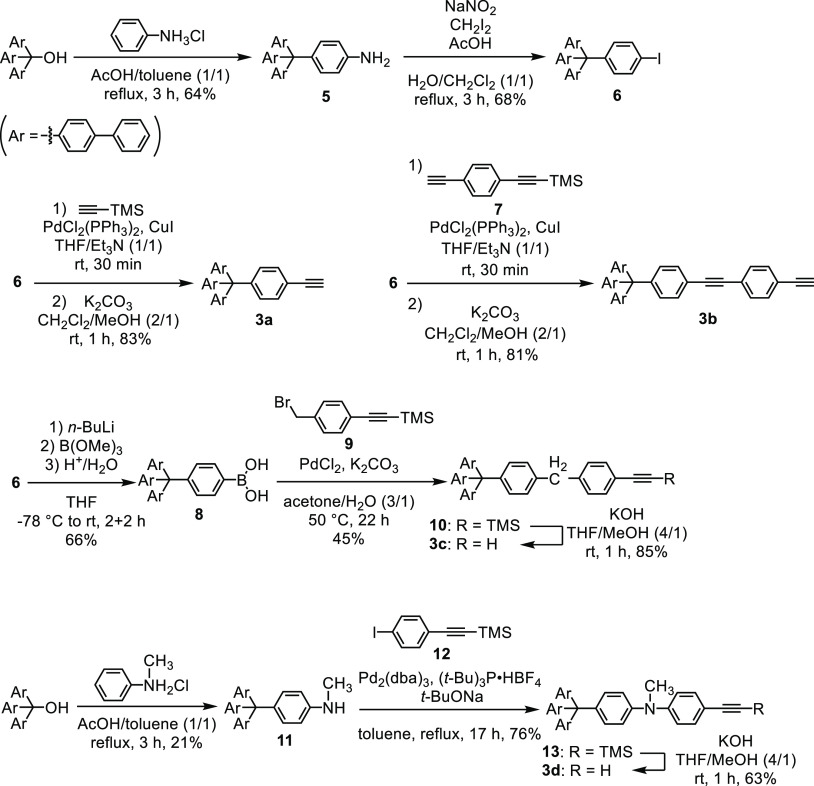
Synthesis of Precursors **3a–d**

**Scheme 5:**
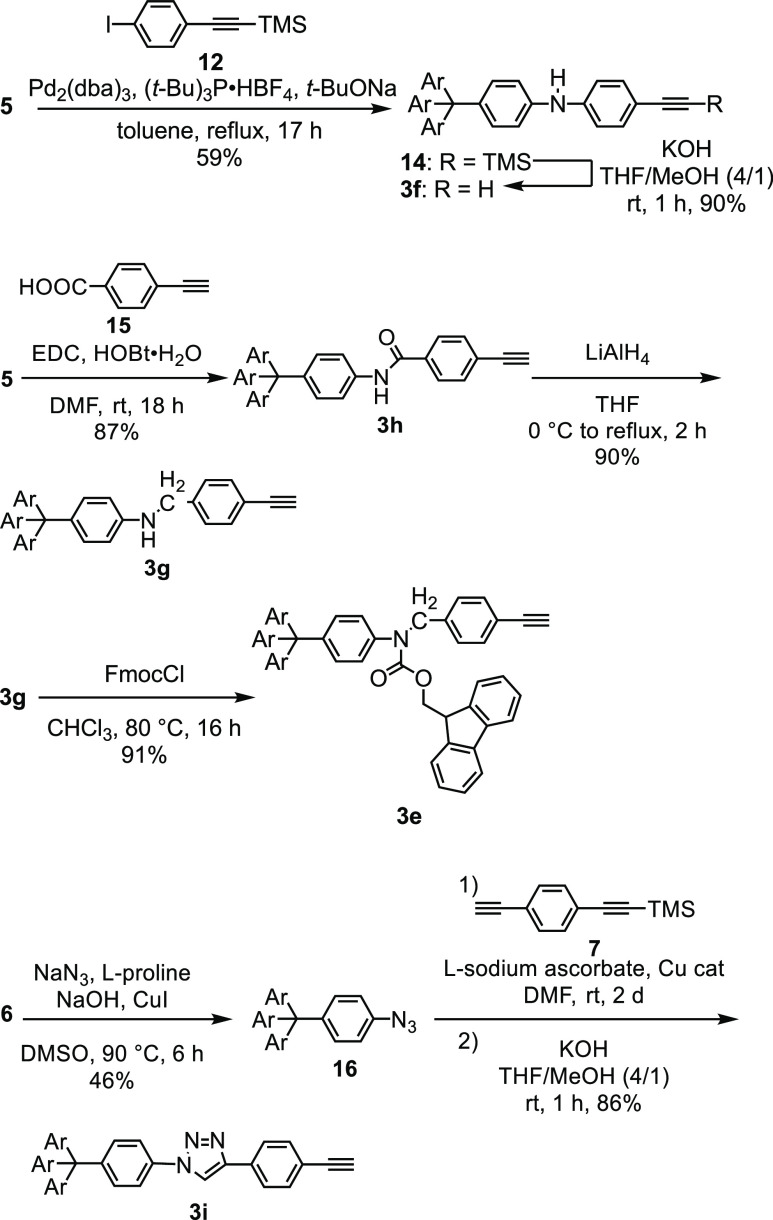
Synthesis of Precursors **3e–i**

As the precursor for the axle component, we designed a series of terminal alkynes with the tris(biphenyl)methyl group. The syntheses of the alkynes **3a–i** are summarized in Schemes 4 and 5.

Tris([1,1′-biphenyl]-4-yl)methanol reacted with aniline hydrochloride under acidic conditions, and the substituted aniline **5**(10) was isolated in 64% yield (Scheme 4). Aniline **5** was converted to aryl iodide **6** by the Sandmeyer reaction.^11^ Compound **6** was further converted to alkynes with various functional groups. For example, the Sonogashira reaction of **6** with (trimethylsilyl)acetylene and the removal of the trimethylsilyl (TMS) group gave **3a** in 83% yield. Similarly, the reaction of **6** with **7**(12) gave alkyne **3b** in comparable yield. Iodide **6** was converted to boronic acid **8** in 66% yield. The introduction of the benzyl group was achieved by the Suzuki–Miyaura reaction of **8** with 4-(trimethylsilylethynyl)benzyl bromide **9**.^13^ The deprotection of **10** under basic conditions gave **3c** in 85% yield. An *N*-methylaniline derivative **11** was synthesized by the reaction of tris([1,1′-biphenyl]-4-yl)methanol and *N*-methylaniline hydrochloride in 21% yield. Compound **11** reacted with iodide **12**(14) to give **13** in 76% yield,^15^ and further removal of the TMS group gave **3d** in 63% yield.

Secondary amine **3f** was prepared by the Pd-catalyzed arylation of **5** and the removal of the TMS group (Scheme 5). Amide **3h** was synthesized by the condensation of **5** with acid **15**.^16^ The reduction of **3h** gave amine **3g** in a high yield. Fluorenylmethoxycarbonyl (Fmoc)-protected compound **3e** was synthesized by treating **3g** with FmocCl. Triazole derivative **3i** was prepared from **6** in three steps.^6^

### Synthesis of [2]Rotaxanes

With the axle precursors in hand, we studied the synthesis of rotaxanes by the reaction of **2** with **3a–i**. The results are summarized in Table 1.

**Table 1:**
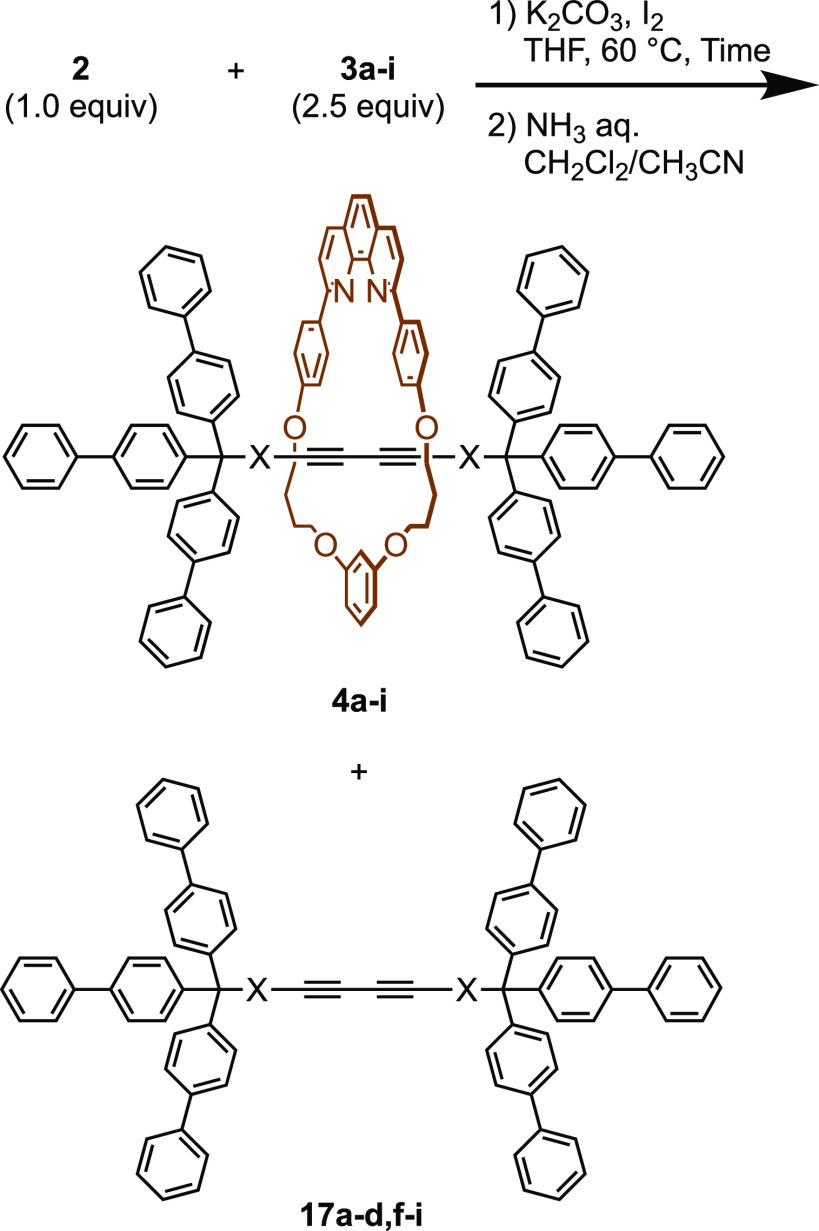

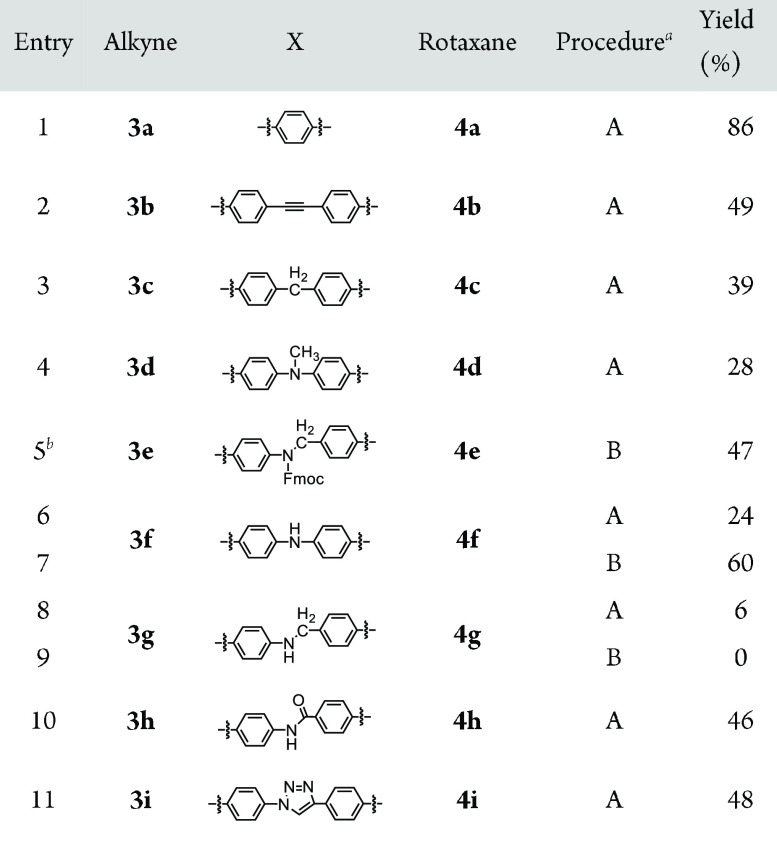
Synthesis of a [2]Rotaxane **4** by Cu-Mediated Oxidative Coupling

a Procedure A: K_2_CO_3_ (10 + 10 equiv), I_2_ (1.0 + 1.0 equiv), time (24 + 24 h); procedure B: K_2_CO_3_ (3.75 equiv), I_2_ (1.25 equiv), time (48 h).

b KCN was employed for the removal of the Cu ion.

**Scheme 6:**
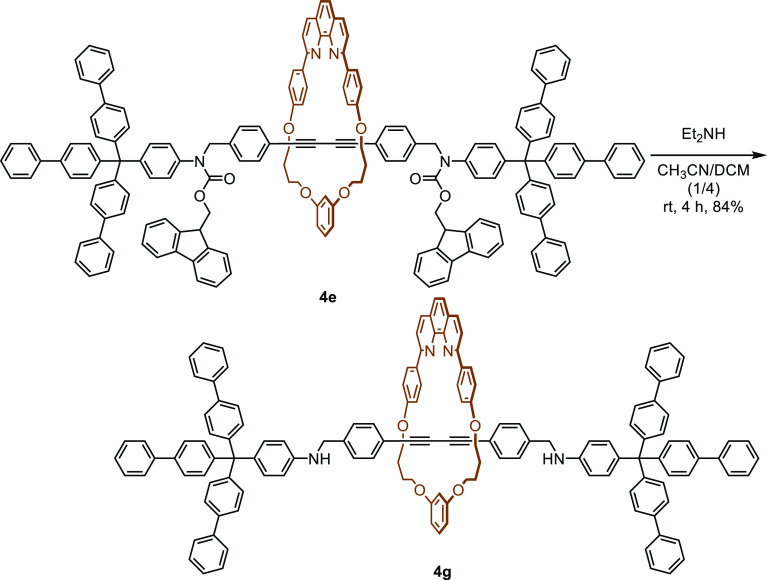
Synthesis of [2]Rotaxane **4g** by Deprotection of **4e**

A mixture of phenanthroline–Cu complex **2** (1 equiv), alkyne **3a** (2.5 equiv), I_2_ (1.0 equiv), and K_2_CO_3_ (10 equiv) in tetrahydrofuran (THF) was heated at 60 °C for 24 h. To the mixture was added I_2_ (1.0 equiv) and K_2_CO_3_ (10 equiv), and the resulting mixture was heated again for 24 h. After the removal of the Cu ion by ammonia, product **4a** was isolated in 86% yield (entry 1, procedure A). Alkynes **3b** and **3c** were reacted with **2** under the same conditions, and [2]rotaxanes were isolated in 49% (**4b**) and 39% (**4c**) yields, respectively (entries 2 and 3). The yield of rotaxane decreased when **3d** was employed as the starting material (28%, entry 4). Rotaxane **4e** was isolated in 47% yield under modified conditions using smaller amounts of K_2_CO_3_ (3.75 equiv) and I_2_ (1.25 equiv, entry 5, procedure B): to prevent the cleavage of the Fmoc group, KCN was used to remove the Cu ion. The synthesis of **4f** was examined under two reaction conditions, and the yield was better (60%) when procedure B was employed (entries 6 and 7). The reaction of benzylamine derivative **3g** gave the corresponding rotaxane **4g** in very low yield regardless of the procedures (entries 8 and 9). We assumed that the diarylamino group induced the removal of the copper ion from the phenanthroline moiety and suppressed the formation of rotaxane. Compound **4g** was synthesized in a better yield by the removal of the Fmoc group from **4e** (Scheme 6). Rotaxanes **4h** and **4i** were synthesized in 46% and 48% yields, respectively, by procedure A (entries 10 and 11).

**Figure 1:**
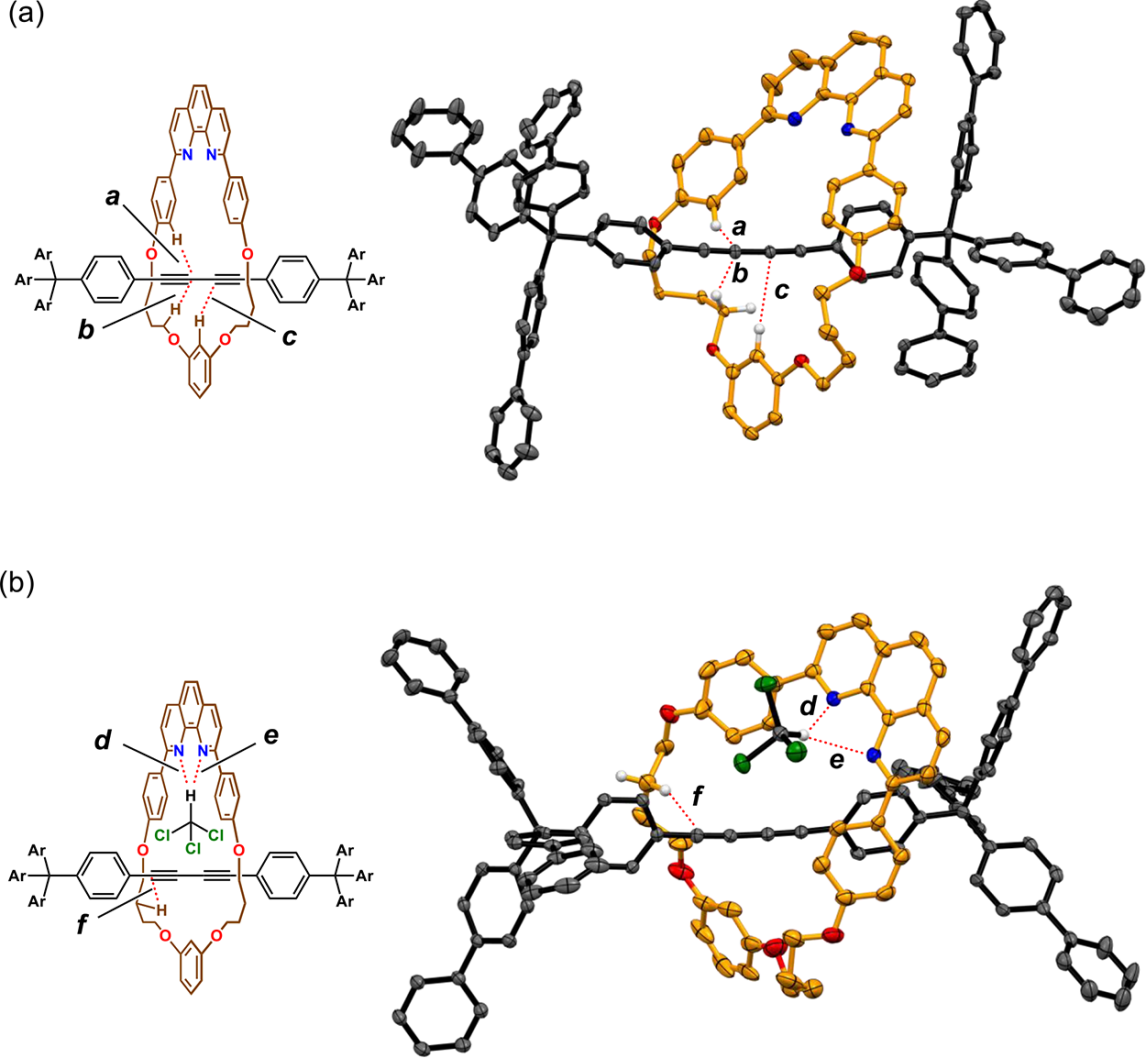
Molecular structure of [2]rotaxane **4a** with thermal ellipsoids at 50% probability. Most hydrogen atoms are omitted for clarity, and noncovalent interactions are shown by red dotted lines. (a) Sample obtained by recrystallization from hexane–toluene. Co-crystallized solvent molecules (toluene and hexane) are omitted for clarity. Only the position with higher occupancy of the disordered methylene groups is shown. *d*(CH/C_sp_): (a) 2.80; (b) 2.79; (c) 2.79 Å. (b) Sample obtained by recrystallization from MTBE–chloroform. Only the position with higher occupancy of the disordered methylene and phenyl groups are shown. *d*(CH/N and CH/C_sp_): (d) 2.32; (e) 2.50; (f) 2.70 Å.

The structure of **4a** was elucidated by X-ray crystallographic analysis, and the results are summarized in Figure 1. The molecular structure of **4a** provided insights into the conformation of the rotaxanes. In the molecular structure obtained by the recrystallization of **4a** from hexane–toluene, short contacts between the C_sp_ carbon atoms and the hydrogen atoms bound to the aromatic ring were observed (Figure 1a).^8f^ We also succeeded in determining the molecular structure of **4a** from another sample, which was obtained by the recrystallization of **4a** from methyl *tert*-butyl ether (MTBE)–chloroform (Figure 1b). In the structure, the C–H···N interaction between chloroform and the phenanthroline moiety, in addition to the short contact between the C_sp_ carbon atom and the hydrogen atom, was detected (Figure 1b). A similar interaction has been reported in the literature.^17^

### Comparison of the ^1^H NMR Spectra of [2]Rotaxanes

**Figure 2:**
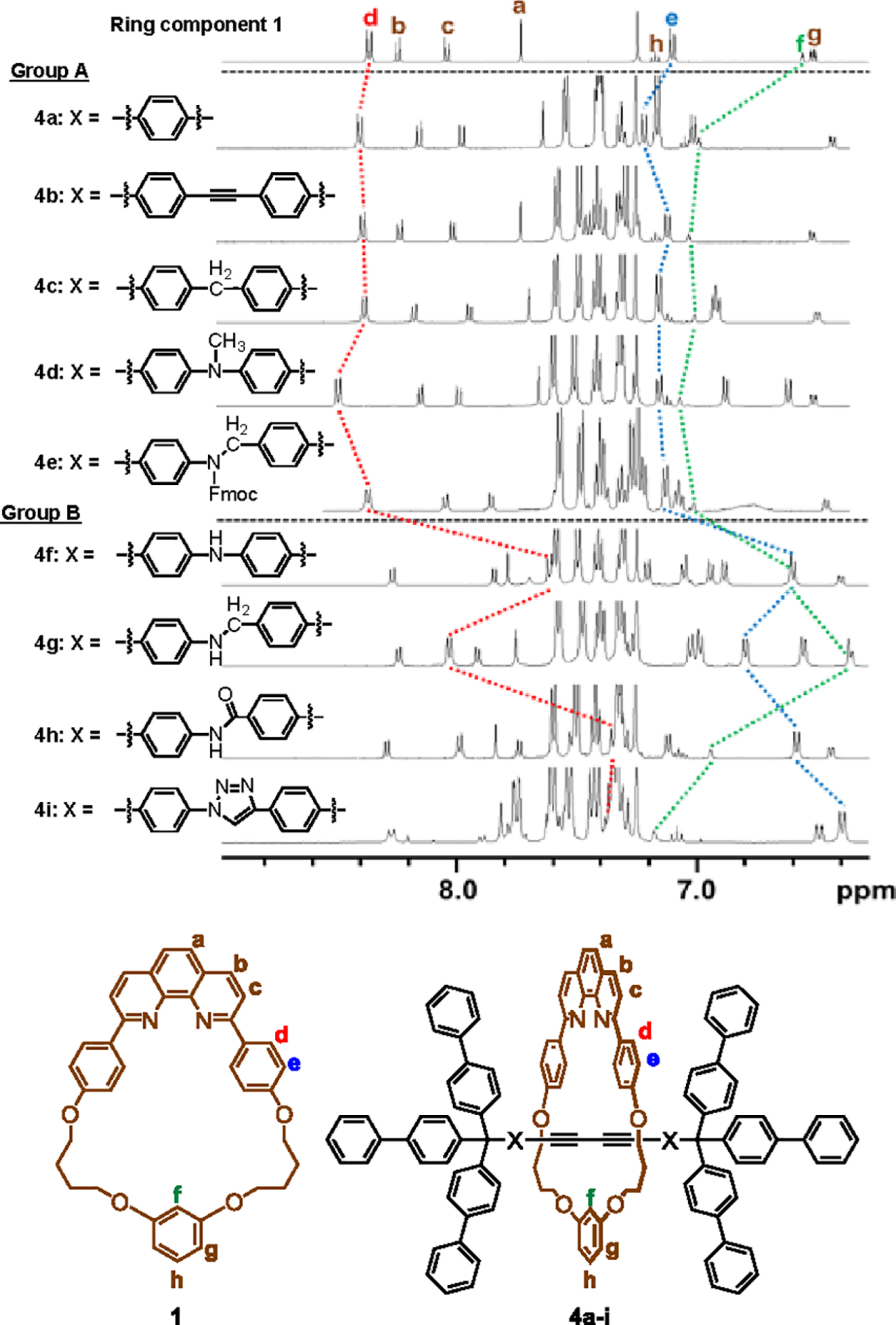
^1^H NMR spectra of [2]rotaxanes **4a–i** (500 MHz, CDCl_3_, 295 K).

Further analysis of the structure and conformation of [2]rotaxanes was done by ^1^H NMR spectroscopy. In the spectra of [2]rotaxanes we studied, sharp signals were detected in most compounds and the localization of the ring component to a specific position was not observed at rt.^18,19^ Based on these results, we assume that the movement of the ring component along the axle component is fast, and the observed chemical shifts are the average of the conformers. Partial ^1^H NMR spectra of ring component **1** and [2]rotaxanes (**4a–i**) are shown in Figure 2. We assigned the signals^20^ that correspond to H^d^, H^e^, and H^f^ of the macrocyclic components, and the chemical shifts were compared (Table 2). Based on the observed chemical shifts, rotaxanes were classified into two groups. In the compounds classified into group A (**4a–e**), the chemical shifts of H^d^, H^e^, and H^f^ appeared at 8.3–8.5, 7.1–7.2, and 7.0–7.1 ppm, respectively. It is noteworthy that the difference in the chemical shifts is small, regardless of the structure of the axle moiety. The chemical shifts of H^d^ and H^e^ in the phenanthroline moiety are similar to those of macrocyclic phenanthroline **1**, while the chemical shift of H^f^, which is bound to the resorcinol framework, shifted downfield (0.4–0.5 ppm) compared to the corresponding signal of **1**. Because a larger difference of the chemical shift in the resorcinol moiety was induced by the formation of the [2]rotaxane, we assume that the “distance”^21^ between the resorcinol moiety and the axle component is short: the axle component is not located in the proximity of the phenanthroline moiety.

**Table 2:**
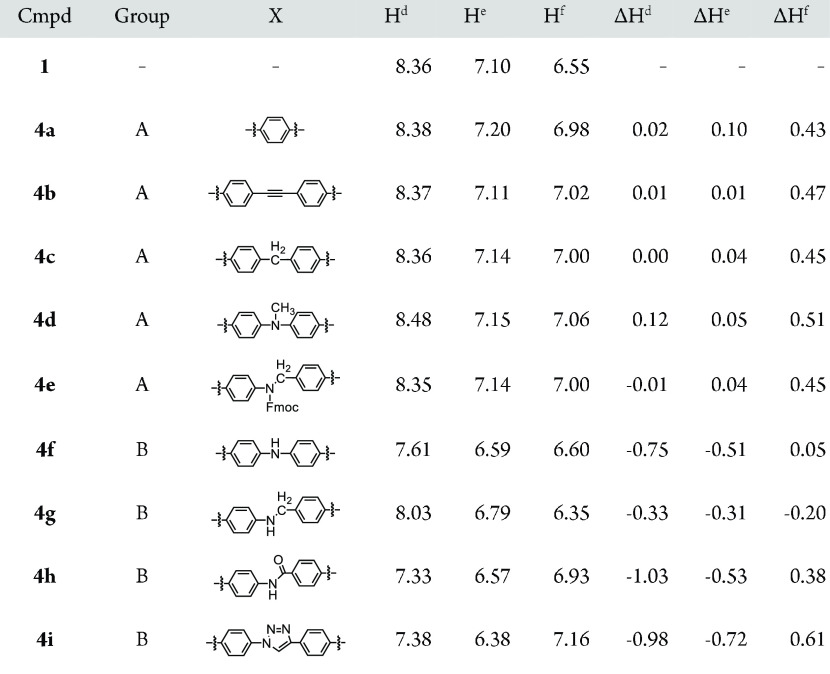
Comparison of the Chemical Shifts (ppm) of [2]Rotaxanes **4a–i** and the Ring Component **1**^a^

a Δ*H*^d–f^ = H^d–f^(**4**) – H^d–f^(**1**).

In contrast, the chemical shifts (H^d^, H^e^, and H^f^) of the compounds classified into group B (**4f–i**) were significantly different from those of **4a**. The chemical shifts of H^d^ and H^e^ in compounds that belong to group B shifted upfield compared to the corresponding chemical shifts of the compounds that belong to group A. For example, the chemical shift of H^d^ in **4a** appeared at 8.38 ppm, while the corresponding signal in **4f** appeared at 7.61 ppm.

Furthermore, the difference in the chemical shifts strongly depends on the structure of the axle moiety, implying that the axle moiety is located in the proximity of the phenanthroline moiety. Next, we compared the chemical shifts of rotaxanes (**4**) with those of the corresponding axle components (**17**, Table 3). The difference between the chemical shifts of the methylene group (H^y^) of **4c** and **17c** was small (ΔH^y^ = −0.19 ppm): in rotaxane **4c**, the signal appeared at 3.77 ppm, while the corresponding signal appeared at 3.96 ppm in **17c**. A similar trend was observed when we compared the chemical shift of the methyl group of **4d** with that of **17d**. The difference between the chemical shifts of the methyl group was small (ΔH^y^ = −0.16 ppm). Rotaxanes **4c** and **4d** belong to group A. When similar analyses were conducted with rotaxanes that belongs to group B, the difference in the chemical shifts was significantly large. The chemical shift (7.68 ppm) of the proton bound to the nitrogen atom in rotaxane (**4f**), for example, shifted upfield (5.87 ppm) in the axle component (**17f**): the difference in the chemical shifts was large (ΔH^y^ = 1.81 ppm). Similar results were obtained when we compared the chemical shifts of rotaxanes **4g–i** with diynes **17g–i**. The signal assigned to H^y^ in **4g–i** shifted upfield (1.03–1.67 ppm) in **17g–i**.

**Table 3:**
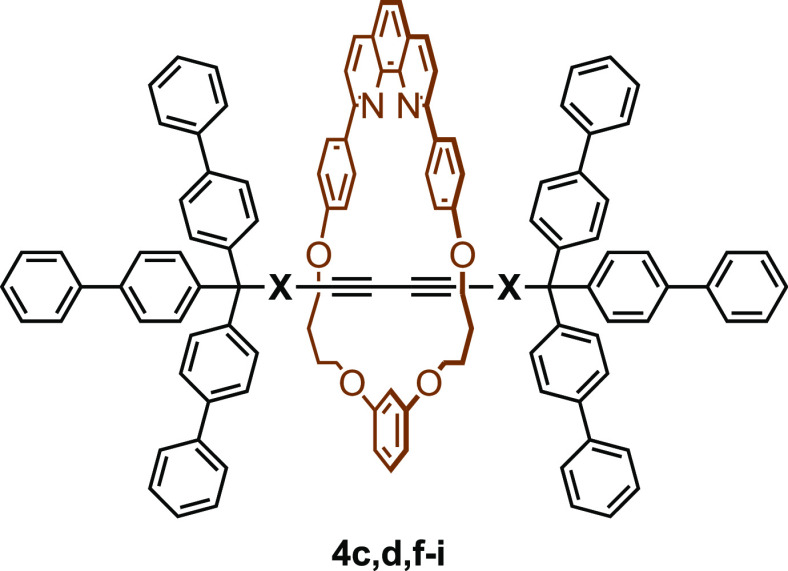

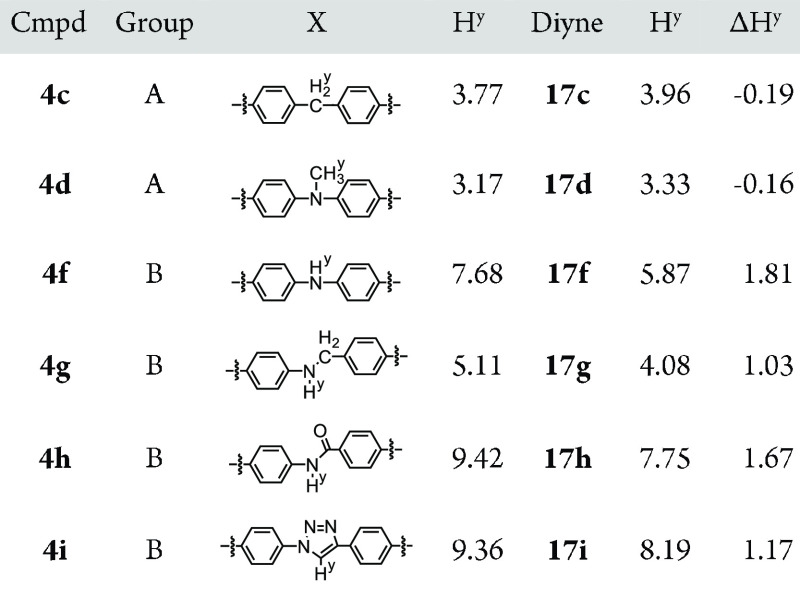
Comparison of the Chemical Shifts (ppm) of [2]Rotaxanes **4c,d,f–i** with the Axle Components **17c,d,f–i**^a^

a ΔH^y^ = H^y^(**4**) – H^y^(**17**).

**Figure 3:**
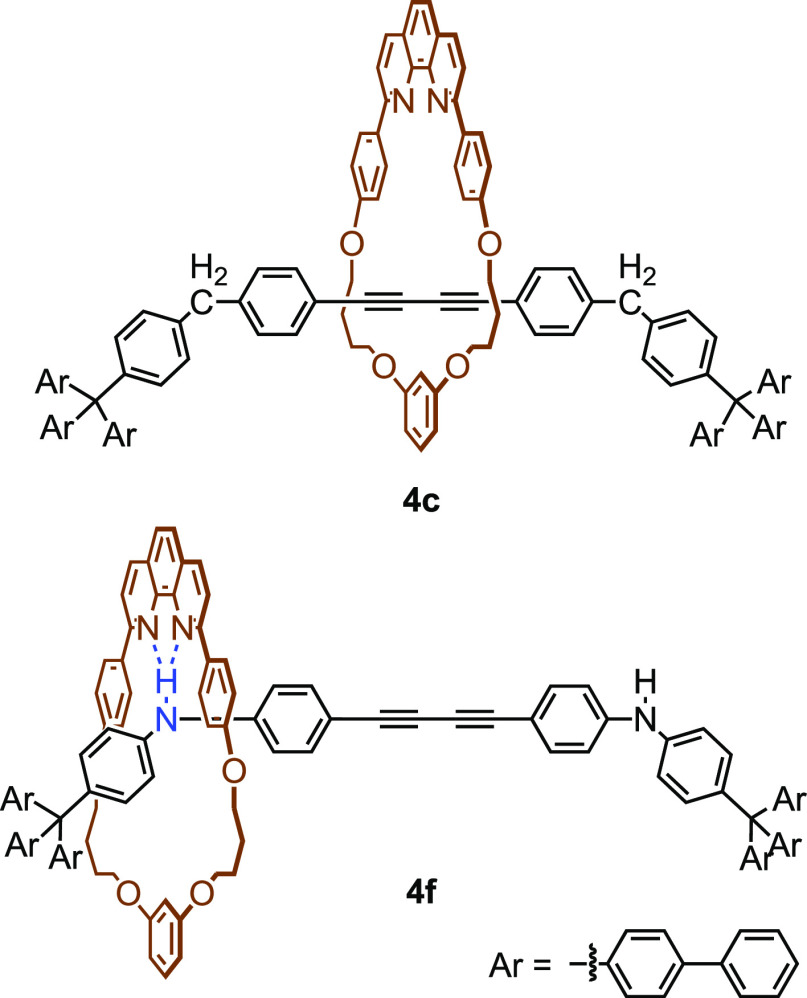
Supposed major conformation of **4c** and **4f** in CDCl_3_.

The results summarized in Tables 2 and 3 could be explained by assuming the presence (or absence) of the hydrogen bond between the axle component and the ring component of rotaxane. In **4c**, which belong to group A, no strong interaction between the axle component and the ring component would be present, and the axle component would be located in the proximity of the resorcinol moiety to minimize the steric interaction between the bulky phenanthroline moiety and the axle component (Figure 3). Consequently, the chemical shifts of H^d^, H^e^, and H^y^ are less affected by the presence of the axle component, while the signal of H^f^ shift downfield. The situation would change significantly in the rotaxanes that belong to group B.

In **4f**, for example, the presence of the hydrogen bond between the axle component and the ring component would affect the conformation of rotaxane (Figure 3). The axle component would be located in the proximity of the phenanthroline moiety. Consequently, the chemical shifts of H^d^ and H^e^ would be significantly affected by the presence of the axle component. The chemical shift of H^y^ would also be strongly affected by the presence of the macrocycle because H^y^ would form a hydrogen bond with the phenanthroline moiety. The formation of the hydrogen bond between the acidic triazole proton and the amine moiety in [2]rotaxane has been postulated by several research groups.^22,23^

**Figure 4:**
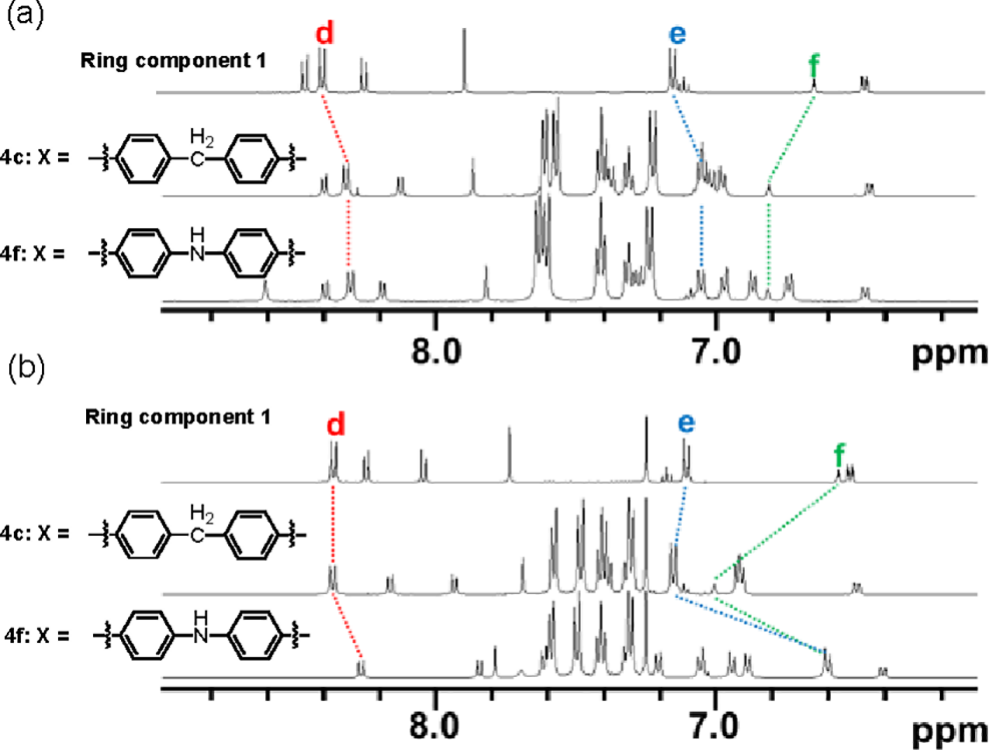
Partial ^1^H NMR spectra of [2]rotaxanes **4c** and **4f** (500 MHz, 295 K); (a) in DMSO-*d*_6_, (b) in CDCl_3_.

If the conformation of rotaxane was influenced by the presence of the hydrogen bond, a notable solvent effect on the chemical shifts of rotaxanes would be observed. In a highly polar solvent, the hydrogen bond between the axle component and the ring component would be cleaved, and this would affect the conformation as well as the chemical shifts of rotaxanes. To confirm the presence of the intramolecular hydrogen bond, we selected **4c**, in which the intramolecular hydrogen bond would not be present, and **4f**, in which the hydrogen bond between the phenanthroline moiety and the amino group would be present. We observed the ^1^H NMR spectra of **4c** and **4f** in two solvents (DMSO-*d*_6_ and CDCl_3_), and the results are shown in Figure 4. The chemical shifts of rotaxanes **4c** and **4f** and ring component **1** are summarized in Table 4. When we observed the ^1^H NMR spectra of **4c** and **4f** in DMSO-*d*_6_, the difference in the chemical shifts of H^d^, H^e^, and H^f^ was small (less than 0.2 ppm), implying that **4c** and **4f** would adopt a similar conformation in DMSO-*d*_6_ (Table 4). Meanwhile, the ^1^H NMR spectra of **4c** and **4f** were different in CDCl_3_. In the NMR spectrum of **4c**, the signal of H^f^ shifted downfield (0.44 ppm) compared to the corresponding signal of macrocyclic phenanthroline **1**, and the difference in other signals (H^d^ and H^e^) was negligible.^24^

**Table 4:**
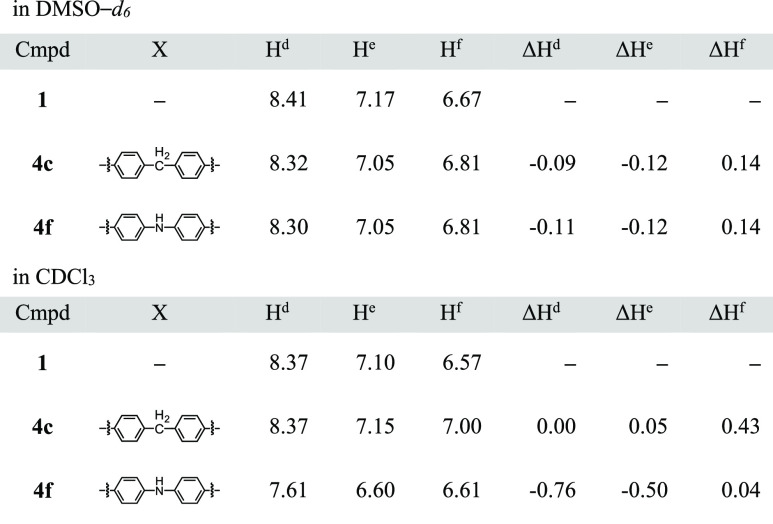
Comparison of Chemical Shifts (ppm) of [2]Rotaxanes **4c,f** and Ring Component **1**^a^

a Δ*H*^d–f^ = H^d–f^(**4**) – H^d–f^(**1**).

The result implies that the axle component of **4c** is located in the proximity of the resorcinol moiety (Figure 3). In contrast, the chemical shifts of H^d^ and H^e^ shifted upfield (0.76 and 0.50 ppm, respectively) in **4f** compared to the corresponding signals of **1**, while the difference in the chemical shifts of H^f^ was small (0.04 ppm). The result could be explained by postulating the presence of the intramolecular hydrogen bond between the axle component and the ring component of **4f** (Figure 3). The axle component of **4f** would be located in the proximity of the phenanthroline moiety, and the chemical shifts of H^d^ and H^e^ would be strongly affected.

The presence of the intramolecular hydrogen bond was also supported by comparing the ^1^H NMR chemical shifts of the axle moiety of rotaxanes and related compounds in different solvents (Table 5). The difference in the chemical shifts of the methylene group of **4c** and that of **17c** in DMSO-*d*_6_ was small (−0.20 ppm). Similar results were observed when the chemical shifts of the NH group of **4f** and that of **17f** in DMSO-*d*_6_ were compared or the chemical shifts of the methylene group of **4c** and that of **17c** in CDCl_3_ were compared. In contrast, a large difference (1.81 ppm) was observed when the chemical shifts of the NH group of **4f** and that of **17f** in CDCl_3_ were compared. The results could be reasonably interpreted by postulating that the intramolecular hydrogen bond is present in a solution of **4f** in CDCl_3_.^25^

**Table 5:**
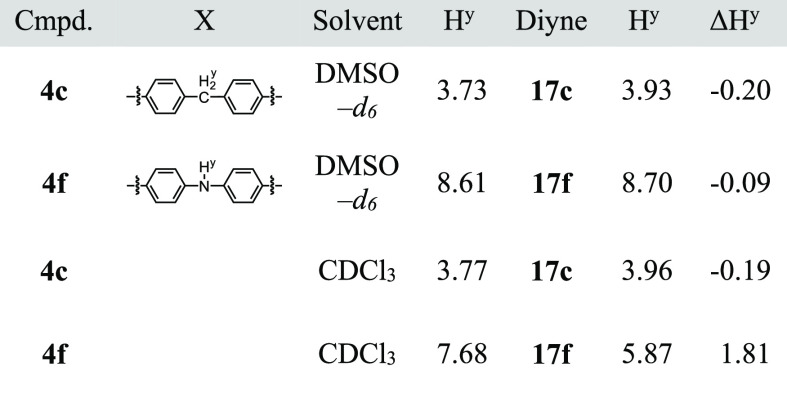
Comparison of the Chemical Shifts (ppm) of [2]Rotaxanes **4c**,**f** and the Axle Components **17c**,**f**^a^

a ΔH^y^ = H^y^(**4**) – H^y^(**17**).

### Variable-Temperature ^1^H NMR Experiments

**Figure 5:**
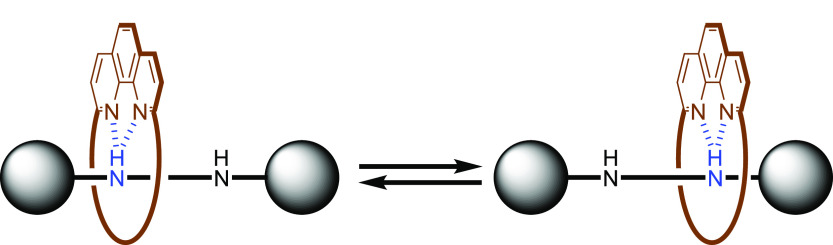
Expected shuttling behavior of group B rotaxanes.

We assumed that the conformation of [2]rotaxanes of group B adopted a structure with a low symmetry (Figure 3b). The observed NMR spectra at 295 K, however, do not directly correspond to the assumed conformation; the signals of the two dumbbell moieties, for example, were equivalent. The observed NMR spectra of [2]rotaxanes of group B could be explained in terms of the fast shuttling of the ring component at 295 K (Figure 5).^22,26^

**Figure 6:**
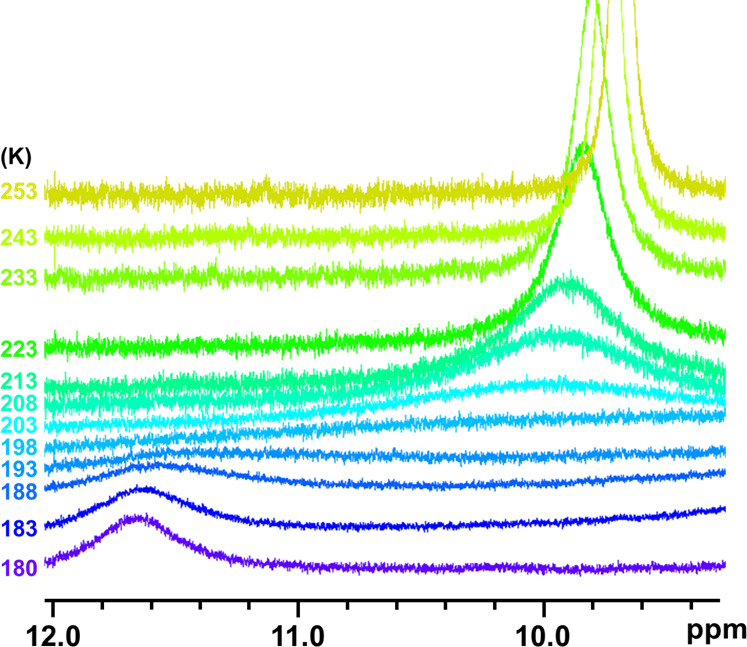
Partial VT ^1^H NMR spectra of **4h** (500 MHz, CD_2_Cl_2_).

Expecting that the rate of the shuttling would decrease at low temperatures and that the signals that reflect the less symmetric structure of [2]rotaxane would appear, we conducted the variable-temperature ^1^H NMR experiments of **4c**, **4f**, and **4h** in CD_2_Cl_2_. When the ^1^H NMR spectrum of **4c**, a negative control, was observed at low temperatures, only the broadening of the signals was observed, and the difference in the chemical shifts was small (Figure S1). Similar results were obtained when the ^1^H NMR spectrum of **4f**, a rotaxane that would form a hydrogen bond, was recorded (Figure S2). In **4h**, on the other hand, the chemical shift of the amide group (H^y^, 11.59 ppm) at 188 K was downfield (1.6 ppm) compared to the corresponding signal at 203 K (9.95 ppm, Figure 6). We assume that the signal observed at 203 K (9.95 ppm) split into two signals at a low temperature (188 K). One signal that appeared at 11.59 ppm would correspond to the amide proton that interacted with the phenanthroline moiety by the hydrogen bond, and the other signal was not detected because the signal overlapped with other signals.^27^ Based on the observed data, the activation energy for the shuttling process of **4h** was assumed to be 8 kcal/mol.^28,29^

**Figure 7:**
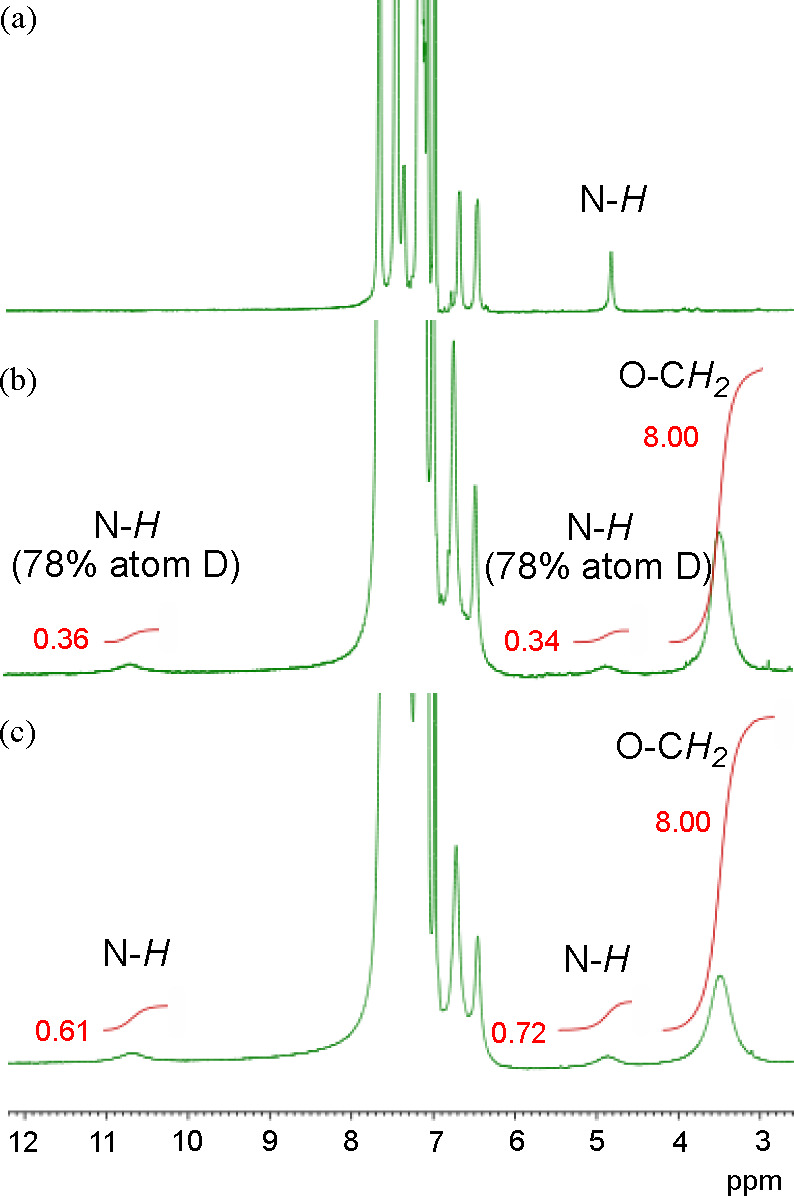
Partial ^1^H NMR spectra of **4f** (bottom, c), **4f-*d*_2_** (middle, b), and **17f** (top, a) at 193 K (400 MHz, toluene-*d*_8_).

We anticipated that the N–H···N interaction would be stronger in a less polar solvent and observed the ^1^H NMR spectra of **4f** in toluene-*d*_8_ (Figure 7c, bottom). Notably, two NH signals were observed at 4.86 and 10.70 ppm at 193 K. We confirmed that these signals correspond to the amino group by observing the ^1^H NMR spectra of the deuterated compound **4f-*d***_**2**_ (78 atom % D of the N–D bond, Figure 7b, middle). Because the signal of the amino group of **17f** (the axle component of **4f**) was observed at 4.83 ppm in toluene-*d*_8_ at 193 K (Figure 7a, top), the signal of **4f**, which appeared at 4.86 ppm, could be assigned to the free amino group, while the signal observed at 10.70 ppm would correspond to the amino group that interacted with the phenanthroline moiety. The amino groups in **4f** appeared as two non-equivalent signals at 193 K due to the decrease in the rate of the shuttling.^8h,30^

## Conclusions

In summary, we synthesized [2]rotaxanes with various functional groups and studied the conformation of the compounds. The comparison of ^1^H NMR spectra of [2]rotaxanes and related components in CDCl_3_ showed that the spectra of rotaxanes were significantly affected by the structure of the axle component. The result could be explained by postulating the presence of the intramolecular hydrogen bond between the phenanthroline moiety and the acidic hydrogen atom in the axle component. The observation of some non-equivalent ^1^H NMR signals at low temperatures supports the idea that the shuttling of the ring component occurs in some rotaxanes that form hydrogen bonds. The study would contribute to the understanding of the conformation of the interlocked compounds.

## Experimental Section

### General Methods

Reagents were commercially available and were used without further purification. An oil bath or a bead bath was used as the heat source, and the external temperature was reported. NMR spectra were recorded on a JEOL 400 or 500 MHz spectrometer or a Bruker 400 MHz NMR spectrometer. Chemical shifts were reported in delta units (δ) relative to chloroform (7.24 ppm for ^1^H NMR and 77.0 ppm for ^13^C NMR) or dimethyl sulfoxide (DMSO) (2.50 ppm for ^1^H NMR and 39.5 ppm for ^13^C NMR). Multiplicity is indicated by s (singlet), d (doublet), t (triplet), q (quartet), quint (quintet), m (multiplet), or br (broad). Coupling constants, *J*, are reported in Hertz. IR spectra were recorded on a Fourier transform infrared spectrometer using a diamond ATR module. A YMC-GPC T30000 (21.2 mm ID  × 600 mm L) column was used for GPC separation using CHCl_3_ as the eluent. Thin layer chromatography was performed on Merck silica gel 60F-254 plates. Column chromatography was performed using Kanto Chemical silica gel 60N (spherical, neutral 40–50 μm). High-resolution mass spectra (HRMS) were obtained by using a time-of-flight (TOF) mass analyzer.

#### Macrocyclic Cu(I)–Phenanthroline Complex (**2**)

To a solution of **1**(8f) (408 mg, 0.70 mmol) in CH_2_Cl_2_ (35 mL) was added a solution of CuI (133 mg, 0.70 mmol, 1 equiv) in CH_3_CN (14 mL), and the mixture was stirred at rt for 2 h. The solvent was removed in vacuo, and the residue was recrystallized from hexane–CH_2_Cl_2_ to yield **2** (328 mg, 0.42 mmol, 61%) as an orange powder: mp 171.1–172.2 °C; ^1^H NMR (400 MHz, CDCl_3_): δ 8.44 (d, *J* = 7.6 Hz, 2H), 8.02 (br, 8H), 7.13 (m, 5H), 7.10 (t, *J* = 8.6 Hz, 1H), 6.59 (s, 2H), 6.47 (d, *J* = 8.4 Hz, 2H), 4,29 (t, *J* = 6.2 Hz, 4H), 4.03 (s, 4H), 2.06 (br, 4H), 1.93 (br, 4H); ^13^C{^1^H} NMR (100 MHz, DMSO-*d*_6_, 423 K): δ 161.0, 160.9, 160.7, 144.6, 138.1, 130.6, 130.5, 130.3, 126.9, 126.7, 116.3, 108.1, 103.2, 79.6, 68.8, 26.3, 26.1 (one signal is missing); IR (ATR): 1603, 1582, 1487 cm^–1^; Anal. Calcd for C_38_H_34_CuIN_2_O_4_·1.6 (CH_2_Cl_2_): C, 52.32; H, 4.12; N, 3.08. Found: C, 52.38; H, 3.73; N, 3.01.

#### 4-[Tris([1,1′-biphenyl]-4-yl)methyl]iodobenzene (**6**)

A mixture of 4-[tris([1,1′-biphenyl]-4-yl)methyl]aniline **5**(10) (2.82 g, 5.0 mmol), NaNO_2_ (1.73 g, 25 mmol, 5 equiv), CH_2_I_2_ (2.68 g, 10 mmol, 2 equiv), CH_2_Cl_2_ (50 mL), and H_2_O (25 mL) was stirred at rt for 5 min under Ar. After the addition of acetic acid (6.01 g, 100 mmol, 20 equiv), the mixture was refluxed for 3 h, cooled to rt, and extracted with CH_2_Cl_2_ (3 × 30 mL). The combined organic layer was washed with water, dried over MgSO_4_, and concentrated in vacuo. The residue was purified by silica gel column chromatography (hexane/CH_2_Cl_2_ = 5/1) to yield **6** (2.26 g, 3.4 mmol, 68%) as a colorless solid; mp 215.7–218.4 °C; ^1^H NMR (500 MHz, CDCl_3_): δ 7.61 (d, *J* = 9.2 Hz, 2H), 7.59 (d, *J* = 8.0 Hz, 6H), 7.51 (d, *J* = 8.0 Hz, 6H), 7.41 (t, *J* = 8.0 Hz, 6H), 7.30–7.33 (m, 9H), 7.08 (d, *J* = 7.5 Hz, 2H); ^13^C{^1^H} NMR (100 MHz, CDCl_3_): δ 146.6, 145.2, 140.4, 138.8, 136.7, 133.2, 131.3, 128.8, 127.3, 127.0, 126.3, 91.9, 64.1; IR (ATR): 1484 cm^–1^; HRMS (FAB): calcd for C_43_H_31_I ([M]^+^), 674.1470; found, 674.1468.

#### 4-[Tris([1,1′-biphenyl]-4-yl)methyl]ethynylbenzene (**3a**)

A mixture of **6** (421 mg, 0.62 mmol), Pd[(PPh_3_)_2_]Cl_2_ (13.1 mg, 0.019 mmol, 3.0 mol %), and CuI (7.1 mg, 0.037 mmol, 6.0 mol %) in dry THF (10 mL) and dry triethylamine (10 mL) was stirred at rt for 5 min under Ar. (Trimethylsilyl)acetylene (0.10 mL, 0.72 mmol, 1.2 equiv) was added in one portion, and the mixture was stirred at rt for 30 min. Saturated NH_4_Cl aq (10 mL) was poured into the solution, and the mixture was extracted with MTBE (3 × 30 mL). The combined organic layer was washed with brine, dried over Na_2_SO_4_, and concentrated in vacuo. To the residue were added K_2_CO_3_ (172 mg, 1.3 mmol, 2.0 equiv), CH_2_Cl_2_ (20 mL), and MeOH (10 mL), and the mixture was stirred at rt for 1 h. After the addition of water, the mixture was extracted with CH_2_Cl_2_ (3 × 25 mL). The combined organic layer was washed with water, dried over MgSO_4_, and concentrated in vacuo. The residue was purified by silica gel column chromatography (hexane/CH_2_Cl_2_ = 5/1) to yield **3a** (298 mg, 0.52 mmol, 83%) as a white solid; mp 232.8–234.6 °C; ^1^H NMR (500 MHz, CDCl_3_): δ 7.59 (d, *J* = 7.5 Hz, 6H), 7.52 (d, *J* = 8.6 Hz, 6H), 7.40–7.45 (m, 8H), 7.30–7.34 (m, 11H), 3.05 (s, 1H); ^13^C{^1^H} NMR (126 MHz, CDCl_3_): δ 147.6, 145.3, 140.4, 138.8, 131.44, 131.39, 131.0, 128.8, 127.3, 127.0, 126.3, 119.7, 83.5, 77.1, 64.3; IR (ATR): 1486 cm^–1^; HRMS (ESI): calcd for C_45_H_32_ ([M]^+^), 572.2499; found, 572.2496.

#### 4-[Tris([1,1′-biphenyl]-4-yl)methyl]-([4-ethynylphenyl]ethynyl)benzene (**3b**)

A mixture of **6** (337 mg, 0.5 mmol), Pd[(PPh_3_)_2_]Cl_2_ (10.5 mg, 0.015 mmol, 3.0 mol %), and CuI (5.71 mg, 0.030 mmol, 6.0 mol %) in dry THF (10 mL) and dry triethylamine (10 mL) was stirred at rt for 5 min. To the mixture was added ([4-ethynylphenyl]ethynyl)trimethylsilane **7** (99.2 mg, 0.5 mmol, 1.0 equiv), and the resulting mixture was stirred for 30 min. The mixture was added to a saturated aqueous solution of NH_4_Cl (10 mL) and extracted with MTBE (3 × 30 mL). The combined organic layer was washed with brine, dried over Na_2_SO_4_, and concentrated in vacuo; To the residue was added K_2_CO_3_ (138 mg, 1.0 mmol, 2.0 equiv), CH_2_Cl_2_ (20 mL), and MeOH (10 mL), and the mixture was stirred at rt for 1 h. After the addition of water, the mixture was extracted with CH_2_Cl_2_ (3 × 25 mL). The combined organic layer was washed with water, dried over MgSO_4_, and concentrated in vacuo. The residue was purified by silica gel column chromatography (hexane/CH_2_Cl_2_ = 5/1) to yield **3b** (273 mg, 0.41 mmol, 81%) as a white solid; mp 238.8–240.4 °C; ^1^H NMR (500 MHz, CDCl_3_): δ 7.61 (d, *J* = 7.5 Hz, 6H), 7.53 (d, *J* = 8.6 Hz, 6H), 7.41–7.47 (m, 12H), 7.31–7.37 (m, 11H), 3.15 (s, 1H); ^13^C{^1^H} NMR (126 MHz, CDCl_3_): δ 147.4, 145.4, 140.4, 138.8, 132.0, 131.5, 131.4, 131.1, 130.9, 128.8, 127.3, 127.0, 126.3, 123.8, 121.8, 120.5, 91.2, 89.0, 83.3, 78.9, 64.3; IR (ATR): 1512, 1486 cm^–1^; HRMS (ESI): calcd for C_53_H_36_ ([M]^+^), 672.2812; found, 672.2803.

#### 4-[Tris([1,1′-biphenyl]-4-yl)methyl]phenylboronic Acid (**8**)

A mixture of **6** (880 mg, 1.3 mmol) in dry THF (8 mL) was cooled to −78 °C under Ar. Then, *n*-BuLi in hexane (1.00 mL, 1.57 M, 1.56 mmol, 1.2 equiv) and B(OMe)_3_ (0.35 mL, 0.33 mmol, 2.4 equiv) were added, and the mixture was stirred for 2 h. The mixture was allowed to warm to rt and stirred again for 2 h. Saturated NH_4_Cl aq (10 mL) was poured into the reaction mixture, and the resulting mixture was stirred for 5 min and extracted with MTBE (3 × 20 mL). The combined organic layer was washed with water, dried over MgSO_4_, and concentrated in vacuo. The residue was purified by silica gel column chromatography (hexane/CH_2_Cl_2_ = 1/2) to yield **8** (510 mg, 0.86 mmol, 66%) as a colorless amorphous solid; mp 231.1–232.7 °C; ^1^H NMR (500 MHz, DMSO-*d*_6_): δ 8.03 (s, 2H), 7.75 (d, *J* = 8.6 Hz, 2H), 7.66–7.69 (m, 12H), 7.45 (t, *J* = 8.0 Hz, 6H), 7.34–7.37 (m, 9H), 7.26 (d, *J* = 8.6 Hz, 2H); ^13^C{^1^H} NMR (100 MHz, DMSO-*d*_6_): δ 149.7, 147.1, 141.0, 139.2, 135.3, 133.5, 132.6, 131.1, 130.5, 129.0, 128.1, 127.6, 65.6; IR (ATR): 1485 cm^–1^; Anal. Calcd for C_43_H_33_BO_2_ (−1/6 H_2_O): C, 87.61; H, 5.59. Found: C, 87.68; H, 5.59.

#### 4-[Tris([1,1′-biphenyl]-4-yl)methyl]-(4-[trimethylsilyl]ethynylbenzyl)benzene (**10**)

To a mixture of **8** (398 mg, 0.67 mmol), [(4-[bromomethyl]phenyl)ethynyl]trimethylsilane **9** (179 mg, 0.67 mmol, 1.0 equiv), K_2_CO_3_ (232 mg, 1.7 mmol, 2.5 equiv), acetone (5.1 mL), and water (1.7 mL) was added PdCl_2_ (2.01 mg, 0.011 mmol, 1.7 mol %) at rt under Ar with stirring. The mixture was heated to 50 °C for 22 h. The solvent was cooled to rt and extracted with CH_2_Cl_2_ (3 × 20 mL). The combined organic layer was dried over Na_2_SO_4_ and concentrated in vacuo. The residue was purified by silica gel column chromatography (hexane/CH_2_Cl_2_ = 3/1) to yield **10** (224 mg, 0.31 mmol, 45%) as a white solid; mp 237.5–239.9 °C; ^1^H NMR (500 MHz, CDCl_3_): δ 7.59 (d, *J* = 7.5 Hz, 6H), 7.51 (d, *J* = 8.0 Hz, 6H), 7.38–7.42 (m, 8H), 7.30–7.34 (m, 9H), 7.22 (d, *J* = 8.0 Hz, 2H), 7.14 (d, *J* = 8.0 Hz, 2H), 7.07 (d, *J* = 8.0 Hz, 2H), 3.94 (s, 2H), 0.23 (s, 9H); ^13^C{^1^H} NMR (100 MHz, CDCl_3_): δ 145.9, 144.6, 141.6, 140.5, 138.6, 138.3, 132.1, 131.5, 131.2, 128.9, 128.7, 128.0, 127.2, 126.9, 126.1, 120.9, 105.1, 93.7, 64.0, 41.4, 0.0; IR (ATR): 1618, 1508, 1484 cm^–1^; HRMS (FAB): calcd for C_55_H_46_Si ([M]^+^), 734.3369; found, 734.3368.

#### 4-[Tris([1,1′-biphenyl]-4-yl)methyl]-(4-ethynylbenzyl)benzene (**3c**)

A mixture of **10** (184 mg, 0.25 mmol), KOH (21.0 mg, 0.38 mmol, 1.5 equiv), MeOH (2.4 mL), and THF (9.6 mL) was stirred at rt for 1 h. To the solution was added water, and the mixture was extracted with EtOAc (3 × 20 mL). The combined organic layer was washed with brine, dried over Na_2_SO_4_, and concentrated in vacuo. The residue was purified by silica gel column chromatography (hexane/CH_2_Cl_2_ = 3/1) to yield **3c** (140 mg, 0.21 mmol, 85%) as a white solid; mp 210.0–212.4 °C; ^1^H NMR (500 MHz, CDCl_3_): δ 7.59 (d, *J* = 8.0 Hz, 6H), 7.51 (d, *J* = 6.9 Hz, 6H), 7.39–7.42 (m, 8H), 7.30–7.35 (m, 9H), 7.23 (d, *J* = 6.9 Hz, 2H), 7.17 (d, *J* = 7.5 Hz, 2H), 7.08 (d, *J* = 8.0 Hz), 3.95 (s, 2H), 3.02 (s, 1H); ^13^C{^1^H} NMR (100 MHz, CDCl_3_): δ 145.8, 144.7, 141.9, 140.5, 138.6, 138.2, 132.3, 131.5, 131.3, 129.0, 128.7, 128.0, 127.2, 126.9, 126.1, 119.8, 83.6, 77.2, 76.8, 64.1, 41.3; IR (ATR): 1506, 1484 cm^–1^; HRMS (ESI): calcd for C_52_H_39_ ([M + H]^+^), 663.3046; found, 663.3035.

#### 4-[Tris([1,1′-biphenyl]-4-yl)methyl]-*N*-methylaniline (**11**)

A mixture of tris([1,1′-biphenyl]-4-yl)methanol (2.44 g, 5.0 mmol) and *N*-methylaniline hydrochloride (1.44 g, 10 mmol, 2 equiv) in dry toluene (10 mL) and acetic acid (10 mL) was refluxed under Ar for 3 h. The mixture was cooled to rt and extracted with CHCl_3_ (3 × 100 mL). Saturated NaHCO_3_ aq (100 mL) was added to the combined organic layer, and the mixture was stirred for 1 h. The organic layer was separated, and the water layer was extracted with CHCl_3_ (3 × 50 mL). The combined organic layer was dried over Na_2_SO_4_ and concentrated in vacuo. The residue was purified by silica gel column chromatography (hexane/CHCl_3_ = 1/2) to yield **11** (608 mg, 1.1 mmol, 21%) as a white solid; mp 230.5–233.1 °C; ^1^H NMR (500 MHz, CDCl_3_): δ 7.59 (d, *J* = 8.0 Hz, 6H), 7.50 (d, *J* = 8.6 Hz, 6H), 7.40 (t, *J* = 8.0 Hz, 6H), 7.35 (d, *J* = 8.0 Hz, 6H), 7.30 (t, *J* = 7.5 Hz, 3H), 7.11 (d, *J* = 8.6 Hz, 2H), 6.58 (d, *J* = 8.0 Hz, 2H), 4.07 (br, 1H), 2.83 (s, 3H); ^13^C{^1^H} NMR (100 MHz, CDCl_3_): δ 147.1, 146.4, 140.7, 138.3, 135.4, 132.0, 131.5, 128.7, 127.1, 126.9, 126.0, 111.5, 63.6, 30.7; IR (ATR): 1613, 1520, 1485, cm^–1^; HRMS (ESI): calcd for C_44_H_36_N ([M + H]^+^), 578.2842; found, 578.2845.

### General Procedure for Amination

A mixture of arylamine (1.0 equiv), ([4-iodophenyl]ethynyl)trimethylsilane **12** (1.1 equiv), NaO*t*-Bu in THF (1.0 M, 1.3 equiv), tri-*tert*-butylphosphonium tetrafluoroborate (10 mol %), Pd_2_(dba)_3_ (5 mol %), and toluene (4.0 mL/1.0 mmol of arylamine) was refluxed under Ar for 17 h. To the solution was added water, and the mixture was extracted with CH_2_Cl_2_. The combined organic layer was washed with water, dried over MgSO_4_, and concentrated in vacuo. The residue was purified by column chromatography.

#### 4-[Tris([1,1′-biphenyl]-4-yl)methyl]-*N*,*N*-methyl-(4-[trimethylsilyl]ethynylphenyl)aniline (**13**)

Following the general procedure for amination, **11** (289 mg, 0.5 mmol), **12** (165 mg, 0.55 mmol, 1.1 equiv), NaO*t*-Bu in THF (1.0 M, 0.65 mL, 0.65 mmol, 1.3 equiv), tri-*tert*-butylphosphonium tetrafluoroborate (14.5 mg, 0.050 mmol), Pd_2_(dba)_3_ (22.9 mg, 0.025 mmol), and toluene (2.0 mL) were used. The residue was purified by silica gel column chromatography (hexane/CH_2_Cl_2_ = 2/1) to yield **13** (284 mg, 0.38 mmol, 76%) as a white solid; mp 224.0–225.7 °C; ^1^H NMR (500 MHz, CDCl_3_): δ 7.60 (d, *J* = 7.5 Hz, 6H), 7.53 (d, *J* = 8.6 Hz, 6H), 7.41 (t, *J* = 7.5 Hz, 6H), 7.36 (d, *J* = 8.6 Hz, 6H),7.30–7.33 (m, 5H), 7.23 (d, *J* = 6.9 Hz, 2H), 7.01 (d, *J* = 9.2 Hz, 2H), 6.87 (d, *J* = 8.6 Hz, 2H), 3.31 (s, 3H), 0.21 (s, 9H); ^13^C{^1^H} NMR (100 MHz, CDCl_3_): δ 148.7, 145.9, 145.8, 141.1, 140.5, 138.6, 132.9, 132.0, 131.4, 128.7, 127.2, 126.9, 126.2, 121.5, 117.5, 113.8, 105.8, 92.3, 77.2, 63.8, 40.0, 0.1; IR (ATR): 1505, 1484 cm^–1^; HRMS (ESI): calcd for C_55_H_48_NSi ([M]^+^), 750.3551; found, 750.3541.

#### 4-[Tris([1,1′-biphenyl]-4-yl)methyl]-*N*-(4-[trimethylsilyl]ethynylphenyl)aniline (**14**)

Following the general procedure for amination, 4-[tris([1,1′-biphenyl]-4-yl)methyl]aniline **5** (564 mg, 1.0 mmol), **12** (330 mg, 1.1 mmol, 1.1 equiv), NaO*t-*Bu in THF (1.0 M, 1.3 mL, 1.3 mmol, 1.3 equiv), tri*-tert-*butylphosphonium tetrafluoroborate (29.0 mg, 0.10 mmol), Pd_2_(dba)_3_ (45.8 mg, 0.050 mmol), and toluene (4.0 mL) were used. The residue was purified by silica gel column chromatography (hexane/CH_2_Cl_2_ = 1/1) to yield **14** (437 mg, 0.594 mmol, 59%) as a white solid; mp 146.3–150.1 °C; ^1^H NMR (500 MHz, CDCl_3_): δ 7.60 (d, *J* = 7.5 Hz, 6H), 7.52 (d, *J* = 8.6 Hz, 6H), 7.41 (t, *J* = 7.5 Hz, 6H), 7.30–7.36 (m, 11H), 7.21 (d, *J* = 8.6 Hz, 2H), 7.02 (d, *J* = 8.6 Hz, 2H), 6.96 (d, *J* = 8.6 Hz, 2H), 5.81 (s, 1H), 0.21 (s, 9H); ^13^C{^1^H} NMR (126 MHz, CDCl_3_): δ 145.9, 143.3, 140.5, 140.0, 139.7, 138.6, 133.2, 132.1, 131.4, 128.7, 127.2, 126.9, 126.1, 117.5, 116.2, 114.4, 92.3, 77.2, 63.8, 0.1; IR (ATR): 1600, 1514, 1486 cm^–1^; HRMS (ESI): calcd for C_54_H_46_NSi ([M + H]^+^), 736.3394; found, 736.3409.

#### 4-[Tris([1,1′-biphenyl]-4-yl)methyl]-*N*,*N*-methyl-(4-ethynylphenyl)aniline (**3d**)

A mixture of **13** (298 mg 0.40 mmol), KOH (33.4 mg, 0.60 mmol, 1.5 equiv), MeOH (3.2 mL), and THF (12.8 mL) was stirred at rt for 1 h. To the solution was added water, and the mixture was extracted with EtOAc (3 × 20 mL). The combined organic layer was washed with brine, dried over Na_2_SO_4_, and concentrated in vacuo. The residue was purified by silica gel column chromatography (hexane/CH_2_Cl_2_ = 1/1) to yield **3d** (167 mg, 0.25 mmol, 63%) as a white solid; mp 217.1–219.0 °C; ^1^H NMR (500 MHz, CDCl_3_): δ 7.60 (d, *J* = 8.0 Hz, 6H), 7.53 (d, *J* = 8.0 Hz, 6H), 7.41 (t, *J* = 7.5 Hz, 6H), 7.30–7.37 (m, 11H), 7.25 (d, *J* = 5.2 Hz, 2H), 7.03 (d, *J* = 8.0 Hz, 2H), 6.88 (d, *J* = 8.0 Hz, 2H), 3.32 (s, 3H), 3.00 (s, 1H); ^13^C{^1^H} NMR (126 MHz, CDCl_3_): δ 148.9, 145.9, 145.7, 141.3, 140.5, 138.6, 133.1, 132.1, 131.5, 128.7, 127.2, 126.9, 126.2, 121.7, 117.3, 112.5, 84.3, 75.6, 63.9, 40.0; IR (ATR): 1598, 1505, 1485 cm^–1^; HRMS (ESI): calcd for C_52_H_40_N ([M + H]^+^), 678.3147; found, 678.3155.

#### 4-[Tris([1,1′-biphenyl]-4-yl)methyl]-*N*-(4-ethynylphenyl)aniline (**3f**)

A mixture of **14** (107 mg 0.15 mmol), KOH (12 mg, 0.22 mmol, 1.5 equiv), MeOH (1.6 mL), and THF (6.4 mL) was stirred at rt for 1 h. To the solution was added water, and the mixture was extracted with EtOAc (3 × 10 mL). The combined organic layer was washed with brine, dried over Na_2_SO_4_, and concentrated in vacuo. The residue was purified by silica gel column chromatography (hexane/CH_2_Cl_2_ = 1/1) to yield **3f** (87.0 mg, 0.073 mmol, 90%) as a white solid; mp 253.3–255.6 °C; ^1^H NMR (500 MHz, CDCl_3_): δ 7.60 (d, *J* = 7.5 Hz, 6H), 7.53 (d, *J* = 8.6 Hz, 6H), 7.42 (t, *J* = 7.5 Hz, 6H), 7.36 (d, *J* = 8.6 Hz, 6H), 7.36 (d, *J* = 8.6 Hz, 2H), 7.32 (t, *J* = 7.5 Hz, 3H), 7.23 (d, *J* = 8.6 Hz, 2H), 7.03 (d, *J* = 8.6 Hz, 2H), 6.98 (d, *J* = 8.6 Hz, 2H), 5.82 (s, 1H), 2.99 (s, 1H); ^13^C{^1^H} NMR (126 MHz, CDCl_3_): δ 145.9, 143.7, 140.5, 140.2, 139.6, 138.6, 133.4, 132.1, 131.4, 128.7, 127.2, 126.9, 126.2, 117.7, 116.2, 113.3, 84.1, 75.7, 63.8; IR (ATR): 1600, 1510, 1485 cm^–1^; HRMS (ESI): calcd for C_51_H_38_N ([M + H]^+^), 664.2999; found, 664.2996.

#### *N*-([4-Tris([1,1′-biphenyl]-4-yl)methyl]phenyl)-4-ethynylbenzamide (**3h**)

A mixture of 4-[tris([1,1′-biphenyl]-4-yl)methyl]benzenamine **5** (564 mg 1.0 mmol), 4-ethynylbenzoic acid **16** (146 mg, 1.0 mmol, 1.0 equiv), EDC (230 mg, 1.2 mmol, 1.2 equiv), and HOBt·H_2_O (184 mg, 1.20 mmol, 1.2 equiv) in anhydrous dimethylformamide (DMF) (5 mL) was stirred at rt for 18 h. To the solution was added water, and the mixture was extracted with EtOAc (3 × 20 mL). The combined organic layer was washed with brine, dried over Na_2_SO_4_, and concentrated in vacuo. The residue was purified by silica gel column chromatography (hexane/CH_2_Cl_2_ = 1/2) to yield **3h** (600 mg, 0.87 mmol, 87%) as a white solid; mp 238.2–240.2 °C; ^1^H NMR (500 MHz, CDCl_3_): δ 7.81 (d, *J* = 8.0 Hz, 2H), 7.75 (s, 1H), 7.58–7.60 (m, 8H), 7.56 (d, *J* = 9.2 Hz, 2H), 7.52 (d, *J* = 8.6 Hz, 6H), 7.41 (t, *J* = 7.5 Hz, 6H), 7.30–7.37 (m, 11H), 3.21 (s, 1H); ^13^C{^1^H} NMR (126 MHz, CDCl_3_): δ 164.9, 145.7, 143.2, 140.5, 138.7, 135.6, 134.8, 132.5, 131.8, 131.4, 128.7, 127.2, 127.0, 126.7, 126.2, 125.8, 119.4, 82.6, 79.9, 64.0; IR (ATR): 1673, 1597, 1518, 1487 cm^–1^; HRMS (ESI): calcd for C_52_H_38_NO ([M + H]^+^), 692.2948; found, 692.2954.

#### 4-[Tris([1,1′-biphenyl]-4-yl)methyl]-*N*-(4-ethynylbenzyl)aniline (**3g**)

To a solution of **3h** (257 mg, 0.37 mmol) in anhydrous THF (5 mL) was added a suspension of LiAlH_4_ (42.3 mg, 1.1 mmol, 3.0 equiv) in THF (1.1 mL) at 0 °C under Ar with stirring. The mixture was stirred at 70 °C for 2 h. To the mixture was added aqueous NaOH (7.5%, 0.2 mL), and the mixture was stirred for 5 min at rt. The mixture was filtered over Celite, and the filter cake was rinsed with EtOAc. The combined organic layer was dried over MgSO_4_ and concentrated in vacuo. The residue was purified by silica gel column chromatography (hexane/CH_2_Cl_2_ = 1/1) to yield **3g** (227 mg, 0.335 mmol, 90%) as a white solid; mp 118.9–120.0 °C; ^1^H NMR (500 MHz, CDCl_3_): δ 7.59 (d, *J* = 8.0 Hz, 6H), 7.49 (d, *J* = 8.0 Hz, 6H), 7.45 (d, *J* = 8.0 Hz, 2H), 7.41 (t, *J* = 7.5 Hz, 6H), 7.29–7.34 (m, 11H), 7.09 (d, *J* = 8.6 Hz, 2H), 6.56 (d, *J* = 7.5 Hz, 2H), 4.31, (s, 2H), 4.08 (br, 1H) 3.04 (s, 1H); ^13^C{^1^H} NMR (100 MHz, CDCl_3_): δ 146.3, 145.8, 140.6, 140.4, 138.4, 135.9, 132.4, 132.0, 131.5, 128.7, 127.4, 127.1, 126.9, 126.0, 120.9, 111.9, 83.5, 77.1, 63.6, 48.2; IR (ATR): 3425, 3286, 1611, 1514, 1485 cm^–1^; HRMS (ESI): calcd for C_52_H_40_N ([M + H]^+^), 678.3155; found, 678.3152.

#### 4-[Tris([1,1′-biphenyl]-4-yl)methyl]-*N*,*N*-(4-ethynylbenzyl)-[(9*H*-fluoren-9-ylmethoxy)carbonyl]aniline (**3e**)

A mixture of **3g** (482 mg, 0.71 mmol), (9*H-*fluoren-9-yl)methyl carbonochloridate (221 mg, 0.85 mmol, 1.2 equiv) in dry CHCl_3_ (10 mL) was stirred at 80 °C under Ar for 16 h. The solution was concentrated in vacuo. The residue was purified by silica gel column chromatography (hexane/CH_2_Cl_2_ = 1/1) to yield **3e** (583 mg, 0.65 mmol, 91%) as a white solid; mp 122.8–124.2 °C; ^1^H NMR (400 MHz, CDCl_3_, 333 K): δ 7.66 (d, *J* = 7.3 Hz, 2H), 7.60 (d, *J* = 7.8 Hz, 6H), 7.52 (d, *J* = 8.2 Hz, 6H), 7.41 (t, *J* = 7.3 Hz, 6H), 7.30–7.38 (m, 15H), 7.22 (dd, *J* = 7.3, 1.8 Hz, 2H), 7.17 (t, *J* = 7.3 Hz, 2H), 7.07 (d, *J* = 7.8 Hz, 2H), 6.93 (d, *J* = 7.8 Hz, 2H), 4.79 (s, 2H), 4.54 (d, *J* = 6.4 Hz, 2H), 4.11 (t, *J* = 6.4 Hz, 1H), 3.03 (s, *J* = 7.3 Hz, 1H); ^13^C{^1^H} NMR (126 MHz, CDCl_3_): δ 155.5, 145.5, 145.1, 143.7, 141.3, 140.4, 139.3, 138.7, 138.5, 132.2, 131.6, 131.4, 128.7, 128.5, 127.6, 127.2, 126.9, 126.9, 126.2, 124.9, 121.0, 119.8, 83.4, 77.3, 67.4, 63.9, 53.9, 47.2; IR (ATR): 1716, 1512, 1488 cm^–1^; HRMS (ESI): calcd for C_67_H_50_NO_2_ ([M + H]^+^), 900.3836; found, 900.3833.

#### 1-Azido-4-[tris([1,1′-biphenyl]-4-yl)methyl]benzene (**16**)

A mixture of **6** (1.08 g 1.6 mmol), NaN_3_ (125 mg, 1.9 mmol, 1.2 equiv), l-proline (36.8 mg, 0.32 mmol, 0.2 equiv), NaOH (12.8 mg, 0.32 mmol, 0.2 equiv), and CuI (30.5 mg, 0.160 mmol, 0.1 equiv) in dry DMSO (6 mL) was stirred at 90 °C under Ar for 6 h. To the mixture was added water at rt, and the mixture was extracted with EtOAc (3 × 20 mL). The combined organic layer was washed with brine, dried over Na_2_SO_4_, and concentrated in vacuo. The residue was purified by silica gel column chromatography (hexane/CH_2_Cl_2_ = 1/1) to yield **16** (430 mg, 0.73 mmol, 46%) as a white solid; mp 174.9–177.0 °C; ^1^H NMR (500 MHz, CDCl_3_): δ 7.59 (d, *J* = 7.5 Hz, 6H), 7.52 (d, *J* = 8.6 Hz, 6H), 7.41 (t, *J* = 8.0 Hz, 2H), 7.30–7.34 (m, 11H), 7.08 (d, *J* = 8.6 Hz, 2H); ^13^C{^1^H} NMR (75 MHz, CDCl_3_): δ 145.5, 143.6, 140.4, 138.8, 137.8, 132.5, 131.4, 128.8, 127.3, 126.9, 126.3, 118.2, 63.9; IR (ATR): 2118, 2082, 1600, 1504, 1487 cm^–1^; HRMS (FAB): calcd for C_43_H_31_N_3_ ([M]^+^), 589.2518; found, 589.2508.

#### 1-([4-Tris([1,1′-biphenyl]-4-yl)methyl]phenyl)-4-(4-ethynylphenyl)-1H-1,2,3-triazole (**3i**)

A mixture of **16** (177 mg, 0.30 mmol), ([4-ethynylphenyl]ethynyl)trimethylsilane (71.4 mg, 0.36 mmol, 1.15 equiv), l-sodium ascorbate (11.9 mg, 0.060 mmol, 0.2 equiv), and CuSO_4_·5H_2_O (15.0 mg, 0.060 mmol, 0.2 equiv) in dry DMF (5 mL) was stirred at rt for 2 days. Saturated NH_4_Cl aq (10 mL) was poured into the solution, and the mixture was extracted with EtOAc (3 × 15 mL). The combined organic layer was washed with brine, dried over Na_2_SO_4_, and concentrated in vacuo. The residue was treated with a solution of KOH (252 mg, 0.45 mmol, 1.5 equiv) in a mixture of THF (16 mL) and MeOH (4 mL), and the mixture was stirred at rt for 1 h. Water was added to the solution, and the mixture was extracted with CH_2_Cl_2_ (3 × 15 mL). The combined organic layer was washed with brine, dried over Na_2_SO_4_, and concentrated in vacuo. The residue was purified by silica gel column chromatography (hexane/CH_2_Cl_2_ = 1/1) to yield **3i** (184 mg, 0.26 mmol, 86%) as a white solid; mp 226.0–227.2 °C; ^1^H NMR (500 MHz, CDCl_3_): δ 8.18 (s, 1H), 7.86 (d, *J* = 8.6 Hz, 2H), 7.71 (d, *J* = 9.2 Hz, 2H), 7.60 (d, *J* = 8.6 Hz, 6H), 7.53–7.58 (m, 10H), 7.42 (t, *J* = 7.5 Hz, 6H), 7.38 (d, *J* = 8.6 Hz, 6H), 7.33 (t, *J* = 7.5 Hz, 3H), 3.13 (s, 1H); ^13^C{^1^H} NMR (126 MHz, CDCl_3_): δ 147.9, 147.6, 145.2, 140.3, 139.0, 134.8, 132.7, 132.4, 131.3, 130.5, 128.8, 127.4, 127.0, 126.4, 125.6, 122.0, 119.8, 117.9, 83.4, 78.1, 64.2; IR (ATR): 3285, 1516, 1486 cm^–1^; HRMS (FAB): calcd for C_53_H_38_N_3_ ([M]^+^), 716.3066; found, 716.3066.

### General Procedure A for the Synthesis of [2]Rotaxanes

A mixture of macrocyclic phenanthroline–CuI complex **2** (1.0 equiv), alkyne (2.5 equiv), K_2_CO_3_ (10 equiv), and I_2_ (1.0 equiv) in dry THF (6.25 mL/0.1 mmol of **2**) was stirred at 60 °C under Ar for 24 h. Then, K_2_CO_3_ (10 equiv) and I_2_ (1.0 equiv) were added, and the mixture was stirred again at 60 °C for 24 h. The mixture was cooled to rt, and CH_2_Cl_2_ (7.5 mL/0.1 mmol of **2**), CH_3_CN (17.5 mL/0.1 mmol of **2**), and NH_3_ aq (30%, 8.5 mL/0.1 mmol of **2**) were added. After stirring at rt overnight, the solution was extracted with CH_2_Cl_2_, dried over Na_2_SO_4_, and concentrated in vacuo. The residue was purified by column chromatography and GPC.

### General Procedure B for the Synthesis of [2]Rotaxanes

A mixture of macrocyclic phenanthroline–CuI complex **2** (1.0 equiv), alkyne (2.5 equiv), K_2_CO_3_ (3.75 equiv), and I_2_ (1.25 equiv) in dry THF (6.25 mL/0.1 mmol of **2**) was stirred at 60 °C under Ar for 48 h. The solution was cooled to rt, and CH_2_Cl_2_ (7.5 mL/0.1 mmol of **2**), CH_3_CN (17.5 mL/0.1 mmol of **2**), and NH_3_ aq (30%, 8.5 mL/0.1 mmol of **2**) were added. After stirring at rt overnight, the solution was extracted with CH_2_Cl_2_. The organic layer was dried over Na_2_SO_4_ and concentrated in vacuo. The residue was purified by column chromatography and GPC.

#### [2]Rotaxane (**4a**) and Diyne (**17a**)

Following the general procedure A, **2** (77.3 mg, 0.10 mmol), **3a** (143 mg, 0.25 mmol), K_2_CO_3_ (138 + 138 mg, 1.0 + 1.0 mmol), and I_2_ (25.4 + 25.4 mg, 0.10 + 0.10 mmol) were used. The residue was purified by silica gel column chromatography (hexane/CHCl_3_ = 1/1) to yield **4a** (108 mg, 0.063 mmol, 86%) as a white solid. The diyne **17a** (28 mg, 0.024 mmol, 20%, based on **3a**) was also isolated as a white solid. Data for **4a**: mp 182.1–184.9 °C; ^1^H NMR (500 MHz, CDCl_3_): δ 8.38 (d, *J* = 8.6 Hz, 4H), 8.14 (d, *J* = 8.6 Hz, 2H), 7.96 (d, *J* = 8.6 Hz, 2H), 7.62 (s, 2H), 7.53 (d, *J* = 8.6 Hz, 12H), 7.38–7.41 (m, 28H), 7.30 (t, *J* = 7.5 Hz, 6H), 7.20 (d, *J* = 8.6 Hz, 4H), 7.15 (d, *J* = 8.6 Hz, 12H), 7.04 (t, *J* = 8.0 Hz, 1H), 7.00 (d, *J* = 8.6 Hz, 4H), 6.98 (t, *J* = 2.3 Hz, 1H), 6.42 (dd, *J* = 2.3, 8.6 Hz, 2H), 4.22 (t, *J* = 7.5 Hz, 4H), 4.08 (t, *J* = 6.3 Hz, 4H), 2.05 (quint, *J* = 7.5 Hz, 4H), 1.91 (quint, *J* = 6.6 Hz, 4H); ^13^C{^1^H} NMR (126 MHz, CDCl_3_): δ 160.4, 159.9, 156.3, 147.8, 146.1, 145.1, 140.4, 138.6, 136.4, 132.14, 132.08, 131.2, 130.7, 129.5, 129.2, 128.7, 127.2, 126.9, 126.2, 125.4, 119.0, 118.9, 115.1, 107.5, 101.8, 83.2, 75.1, 68.0, 67.7, 64.2, 26.2, 25.8; IR (ATR): 1601, 1586, 1485 cm^–1^; HRMS (MALDI): calcd for C_128_H_97_N_2_O_4_ ([M + H]^+^), 1425.7424; found, 1725.7443. Data for **17a**: mp 333.7–334.2 °C; ^1^H NMR (500 MHz, CDCl_3_): δ 7.59 (d, *J* = 8.0 Hz, 12H), 7.52 (d, *J* = 8.0 Hz, 12H), 7.45 (d, *J* = 8.6 Hz, 4H), 7.41 (t, *J* = 7.5 Hz, 12H), 7.30–7.33 (m, 22H); ^13^C{^1^H} NMR (126 MHz, CDCl_3_): δ 148.1, 145.2, 140.4, 138.8, 131.8, 131.4, 131.1, 128.8, 127.3, 127.0, 126.3, 119.4, 81.5, 74.1, 64.4; IR (ATR): 1599, 1486 cm^–1^; HRMS (MALDI): calcd for C_90_H_63_ ([M + H]^+^), 1143.4924; found, 1143.4961.

#### [2]Rotaxane (**4b**)

Following the general procedure A, **2** (77.3 mg, 0.10 mmol), **3b** (168 mg, 0.25 mmol), K_2_CO_3_ (138 + 138 mg, 1.0 + 1.0 mmol), and I_2_ (25.4 + 25.4 mg, 0.10 + 0.10 mmol) were used. The residue was purified by silica gel column chromatography (hexane/CHCl_3_ = 1/1) to yield **4b** (95 mg, 0.049 mmol, 49%) as a white solid; mp 191.8–195.9 °C; ^1^H NMR (500 MHz, CDCl_3_): δ 8.37 (d, *J* = 8.6 Hz, 4H), 8.22 (d, *J* = 8.0 Hz, 2H), 8.00 (d, *J* = 8.6 Hz, 2H), 7.72 (s, 2H), 7.57 (d, *J* = 6.9 Hz, 12H), 7.47 (d, *J* = 8.0 Hz, 12H), 7.44 (d, *J* = 8.6 Hz, 4H), 7.40 (t, *J* = 8.0 Hz, 12H), 7.37 (d, *J* = 8.6 Hz, 4H), 7.27–7.32 (m, 22H), 7.23 (d, *J* = 5.7 Hz, 4H), 7.16 (t, *J* = 8.0 Hz, 1H), 7.11 (d, *J* = 9.2 Hz, 4H), 7.02 (t, *J* = 2.3 Hz, 2H), 6.51 (dd, *J* = 2.3, 8.0 Hz, 2H), 4.10 (t, *J* = 7.5 Hz, 4H), 4.06 (t, *J* = 6.3 Hz, 4H), 1.99 (quint, *J* = 7.5 Hz, 4H), 1.88 (quint, *J* = 6.6 Hz, 4H); ^13^C{^1^H} NMR (126 MHz, CDCl_3_): δ 160.4, 159.8, 156.5, 147.2, 146.2, 145.3, 140.3, 138.6, 136.4, 132.5, 132.2, 131.5, 131.3, 131.0, 129.8, 129.2, 128.7, 127.3, 127.2, 126.9, 126.2, 125.4, 124.0, 121.0, 120.4, 119.3, 115.0, 107.1, 102.2, 92.2, 89.4, 83.0, 76.1, 67.9, 67.7, 64.2, 26.2, 25.7; IR (ATR): 1601, 1587, 1485, cm^–1^; HRMS (MALDI): calcd for C_144_H_105_N_2_O_4_ ([M + H]^+^), 1925.8069; found, 1925.8129.

#### [2]Rotaxane (**4c**) and Diyne (**17c**)

Following the general procedure A, **2** (15.5 mg, 0.020 mmol), **3c** (33.1 mg, 0.050 mmol), K_2_CO_3_ (28 + 28 mg, 0.20 + 0.20 mmol), and I_2_ (5.1 + 5.1 mg, 0.020 + 0.020 mmol) were used. The residue was purified by silica gel column chromatography (hexane/CHCl_3_ = 1/1) to yield **4c** (15 mg, 0.0077 mmol, 39%) as a white solid. The axle **17c** (8.7 mg, 0.0066 mmol, 26%, based on **3c**) was also isolated as a white solid. Data for **4c**: mp 181.3–184.2 °C; ^1^H NMR (500 MHz, CDCl_3_): δ 8.36 (d, *J* = 8.0 Hz, 4H), 8.15 (d, *J* = 8.6 Hz, 2H), 7.93 (d, *J* = 8.0 Hz, 2H), 7.68 (s, 2H), 7.57 (d, *J* = 8.0 Hz, 12H), 7.47 (d, *J* = 7.5 Hz, 12H), 7.40 (t, *J* = 7.5 Hz, 12H), 7.38 (d, *J* = 7.5 Hz, 4H), 7.29–7.32 (m, 18H), 7.14 (d, *J* = 8.6 Hz, 8H), 7.10 (t, *J* = 8.6 Hz, 1H), 7.00 (s, 1H), 6.91 (t, *J* = 6.9 Hz, 8H), 6.49 (d, *J* = 8.6 Hz, 2H), 4.16 (t, *J* = 8.0 Hz, 4H), 4.10 (t, *J* = 6.9 Hz, 4H), 3.77 (s, 4H), 2.02 (quint, *J* = 7.5 Hz, 4H), 1.91 (quint, *J* = 6.9 Hz, 4H); ^13^C{^1^H} NMR (126 MHz, CDCl_3_): δ 160.5, 159.9, 156.4, 146.1, 145.8, 144.5, 142.1, 140.5, 138.5, 138.0, 136.4, 132.9, 132.1, 131.4, 131.1, 129.6, 129.1, 129.0, 128.7, 128.0, 127.2, 126.9, 126.1, 125.4, 119.1, 119.0, 115.0, 107.8, 101.7, 83.0, 74.7, 68.0, 67.7, 64.0, 41.2, 26.0, 25.9; IR (ATR): 1602, 1587, 1487 cm^–1^; HRMS (MALDI): calcd for C_142_H_109_N_2_O_4_ ([M + H]^+^), 1905.8382; found, 1905.8391. Data for **17c**: mp 289.7–291.2 °C; ^1^H NMR (500 MHz, CDCl_3_): δ 7.59 (d, *J* = 8.0 Hz, 12H), 7.50 (d, *J* = 7.5 Hz, 12H), 7.44 (t, *J* = 8.0 Hz, 4H), 7.40 (t, *J* = 6.9 Hz, 12H), 7.29–7.34 (m, 18H), 7.23 (d, *J* = 7.5 Hz, 4H), 7.18 (d, *J* = 7.5 Hz, 4H), 7.08 (d, *J* = 8.0 Hz, 4H), 3.96 (s, 4H); ^13^C{^1^H} NMR (126 MHz, CDCl_3_): δ 145.8, 144.7, 142.5, 140.6, 138.6, 138.0, 132.6, 131.5, 131.3, 129.2, 128.7, 128.1, 127.2, 127.0, 126.1, 119.6, 81.5, 73.7, 64.1, 41.4; IR (ATR): 1487, 1427, 1411 cm^–1^; HRMS (MALDI): calcd for C_104_H_75_ ([M + H]^+^), 1323.5863; found, 1323.5844.

#### [2]Rotaxane (**4d**) and Diyne (**17d**)

Following the general procedure A, **2** (15.5 mg, 0.020 mmol), **3d** (33.9 mg, 0.050 mmol), K_2_CO_3_ (28 + 28 mg, 0.20 + 0.20 mmol), and I_2_ (5.1 + 5.1 mg, 0.020 + 0.020 mmol) were used. The residue was purified by silica gel column chromatography (hexane/CHCl_3_ = 1/1) to yield **4d** (11 mg, 0.0057 mmol, 28%) as a brown solid. The axle **17d** (16.2 mg, 0.012 mmol, 48% based on **3d**) was also isolated as a yellow solid. Data for **4d**: mp 199.8–201.0 °C; ^1^H NMR (500 MHz, CDCl_3_): δ 8.48 (d, *J* = 9.2 Hz, 4H), 8.14 (d, *J* = 8.6 Hz, 2H), 7.98 (d, *J* = 8.6 Hz, 2H), 7.65 (s, 2H), 7.59 (d, *J* = 7.5 Hz, 12H), 7.50 (d, *J* = 8.6 Hz, 12H), 7.41 (t, *J* = 7.5 Hz, 12H), 7.30–7.32 (m, 22H), 7.25 (d, *J* = 6.9 Hz, 4H), 7.15 (d, *J* = 8.6 Hz, 4H), 7.12 (t, *J* = 8.0 Hz, 1H), 7.06 (1H, s), 6.88 (d, *J* = 8.6 Hz, 4H), 6.61 (d, *J* = 8.6 Hz, 4H), 6.51 (dd, *J* = 2.3, 8.6 Hz, 2H), 4.23 (t, *J* = 8.0 Hz, 4H), 4.18 (t, *J* = 6.9 Hz, 4H), 3.17 (s, 6H), 2.06 (quint, *J* = 7.5 Hz, 4H), 1.96 (quint, *J* = 6.9 Hz, 4H); ^13^C{^1^H} NMR (126 MHz, CDCl_3_): δ 160.6, 160.1, 156.1, 148.8, 146.0, 145.8, 145.3, 141.5, 140.5, 138.5, 136.3, 133.6, 132.0, 131.9, 131.4, 129.5, 129.1, 128.7, 127.2, 127.1, 126.9, 126.1, 125.3, 122.0, 118.7, 116.8, 115.1, 111.5, 108.3, 101.3, 83.7, 74.3, 68.1, 67.8, 63.8, 39.8, 26.0, 25.9; IR (ATR): 1590, 1505, 1485 cm^–1^; HRMS (MALDI): calcd for C_142_H_111_N_4_O_4_ ([M + H]^+^), 1935.8600; found, 1935.8669. Data for **17d**: mp 250.9–252.0 °C; ^1^H NMR (500 MHz, CDCl_3_): δ 7.60 (d, *J* = 7.5 Hz, 12H), 7.53 (d, *J* = 8.0 Hz, 12H), 7.41 (t, *J* = 7.5 Hz, 12H), 7.35–7.37 (m, 16H), 7.31 (t, *J* = 7.5 Hz, 6H), 7.27 (d, *J* = 8.6 Hz, 4H), 7.06 (d, *J* = 8.6 Hz, 4H), 6.84 (d, *J* = 8.6 Hz, 4H), 3.33 (s, 6H); ^13^C{^1^H} NMR (126 MHz, CDCl_3_): δ 149.1, 145.8, 145.5, 142.0, 140.5, 138.6, 133.5, 132.2, 131.5, 128.8, 127.2, 127.0, 126.2, 122.6, 116.6, 111.8, 82.1, 73.1, 63.9, 40.1; IR (ATR): 1595, 1504, 1487 cm^–1^; HRMS (MALDI): calcd for C_104_H_76_N_2_ ([M]^+^), 1352.6003; found, 1352.5990.

#### [2]Rotaxane (**4e**)

A mixture of **2** (77.3 mg, 0.10 mmol), **3e** (225 mg, 0.25 mmol 2.5 equiv), K_2_CO_3_ (51.8 mg, 0.375 mmol, 3.75 equiv), and I_2_ (31.7 mg, 0.125 mmol, 1.25 equiv) in dry THF (6.25 mL) was stirred at 60 °C under Ar for 48 h. The mixture was cooled to rt, and CH_2_Cl_2_ (17.5 mL), CH_3_CN (17.5 mL), KCN (52.1 mg, 0.80 mmol, 8.0 equiv), and water (10 mL) were added. After stirring at rt for 1 h, the mixture was extracted with CH_2_Cl_2_, dried over Na_2_SO_4_, and concentrated in vacuo. The residue was purified by silica gel column chromatography (hexane/AcOEt = 2/1) and GPC to yield **4e** (112 mg, 0.047 mmol, 47%) as a white solid; mp 158.7–160.1 °C; ^1^H NMR (500 MHz, CDCl_3_): δ 8.39 (d, *J* = 7.5 Hz, 4H), 8.05 (d, *J* = 6.9 Hz, 2H), 7.69 (d, *J* = 8.6 Hz, 2H), 7.58–7.60 (m, 18H), 7.50 (d, *J* = 6.9 Hz, 12H), 7.40–7.44 (m, 16H), 7.29–7.34 (m, 18H), 7.23–7.25 (m, 8H), 7.15–7.17 (m, 8H), 7.09–7.11 (m, 5H), 7.05 (s, 1H), 6.79 (br, 8H), 6.49 (d, *J* = 8.0 Hz, 2H), 4.60 (s, 4H), 4.23 (d, *J* = 5.2 Hz, 4H), 4.15 (t, *J* = 7.5 Hz, 4H), 4.10 (t, *J* = 6.3 Hz, 4H), 3.98 (t, *J* = 5.7 Hz, 2H), 2.01 (quint, *J* = 6.9 Hz, 4H), 1.88 (quint, *J* = 6.3 Hz, 4H); ^13^C{^1^H} NMR (126 MHz, CDCl_3_): δ 160.4, 159.9, 156.2, 155.4, 146.0, 145.5, 144.9, 143.6, 141.2, 140.4, 139.5, 139.1, 138.6, 136.4, 132.9, 131.9, 131.5, 131.4, 129.6, 129.1, 128.7, 128.5, 127.5, 127.2, 127.2, 126.9, 126.6, 126.2, 125.9, 125.3, 124.8, 120.2, 119.8, 118.9, 115.0, 107.6, 101.8, 83.0, 75.0, 68.0, 67.7, 67.2, 63.9, 53.8, 47.1, 26.1, 25.8; IR (ATR): 1716, 1605, 1489 cm^–1^; HRMS (MALDI): calcd for C_172_H_131_N_4_O_8_ ([M + H]^+^), 2379.9961; found, 2379.9975.

#### [2]Rotaxane (**4f**) and Diyne (**17f**)

Following the general procedure A, **2** (40.2 mg, 0.052 mmol), **3f** (86.3 mg, 0.13 mmol), K_2_CO_3_ (72 + 72 mg, 0.052 + 0.052 mmol), and I_2_ (13 + 13 mg, 0.52 + 0.52 mmol) were used. The residue was purified by silica gel column chromatography (hexane/CHCl_3_ = 1/2) to yield **4f** (32 mg, 0.012 mmol, 24%) as a brown solid. The axle **17f** (41 mg, 0.031 mmol, 48%, based on **3f**) was also isolated as a brown solid. Data for **4f**: mp 206.3–208.0 °C. ^1^H NMR (500 MHz, CDCl_3_): δ 8.26 (d, *J* = 8.0 Hz, 2H), 7.84 (d, *J* = 8.6 Hz, 2H), 7.78 (s, 2H), 7.68 (s, 2H), 7.61 (d, *J* = 8.6 Hz, 4H), 7.58 (d, *J* = 7.5 Hz, 12H), 7.48 (d, *J* = 8.6 Hz, 12H), 7.40 (t, *J* = 7.5 Hz, 12H), 7.29–7.32 (m, 18H), 7.19 (d, *J* = 8.6 Hz, 4H), 7.02–7.05 (m, 5H), 6.93 (d, *J* = 8.6 Hz, 4H), 6.87 (d, *J* = 8.6 Hz, 4H), 6.58–6.60 (m, 5H), 6.39 (dd, *J* = 2.3, 8.0 Hz, 2H), 3.92 (t, *J* = 6.3 Hz, 4H), 3.84 (t, *J* = 6.9 Hz, 4H), 1.75–1.81 (m, 8H); ^13^C{^1^H} NMR (126 MHz, CDCl_3_): δ 160.3, 159.3, 159.2, 146.5, 146.1, 144.2, 140.6, 139.8, 138.9, 138.5, 136.6, 133.4, 132.7, 131.6, 131.5, 130.0, 129.8, 128.7, 128.5, 127.5, 127.2, 126.9, 126.0, 125.8, 121.9, 117.3, 115.5, 114.1, 111.4, 106.8, 101.8, 82.5, 72.9, 67.3, 63.7, 25.8, 25.4; IR (ATR): 3398, 1596, 1512, 1486 cm^–1^; HRMS (MALDI): calcd for C_140_H_107_N_4_O_4_ ([M + H]^+^), 1907.8287; found, 1907.8234. Data for **17f**: mp 272.4–274.2 °C; ^1^H NMR (500 MHz, CDCl_3_): δ 7.60 (d, *J* = 7.5 Hz, 12H), 7.52 (d, *J* = 8.0 Hz, 12H), 7.41 (t, *J* = 7.5 Hz, 12H), 7.35–7.38 (m, 16H), 7.31 (t, *J* = 8.0 Hz, 6H), 7.23 (d, *J* = 8.0 Hz, 4H), 7.04 (d, *J* = 8.6 Hz, 4H), 6.97 (d, *J* = 8.6 Hz, 4H), 5.87 (s, 2H); ^13^C NMR (126 MHz, CDCl_3_): δ 145.9, 144.0, 140.5, 139.3, 138.6, 133.8, 132.1, 131.4, 128.7, 127.2, 126.9, 126.2, 118.0, 116.0, 112.9, 81.9, 73.1, 63.8; IR (ATR): 3398, 1595, 1505, 1484, cm^–1^; HRMS (MALDI): calcd for C_102_H_73_N_2_ ([M]^+^), 1324.5690; found, 1324.5662.

The synthesis of **4f** was also studied by following procedure B. Compound **2** (86 mg, 0.11 mmol), **3f** (184 mg, 0.28 mmol), K_2_CO_3_ (57 mg, 0.42 mmol), and I_2_ (35 mg, 0.14 mmol) were used. The residue was purified by silica gel column chromatography (hexane/CHCl_3_ = 1/2) to yield **4f** (126 mg, 0.066 mmol, 60%) as a brown solid.

#### [2]Rotaxane (**4g**) and Diyne (**17g**)

Following the general procedure A, **2** (15.5 mg, 0.020 mmol), **3g** (33.9 mg, 0.050 mmol), K_2_CO_3_ (28 + 28 mg, 0.20 + 0.20 mmol), and I_2_ (5.1 + 5.1 mg, 0.020 + 0.020 mmol) were used. The residue was purified by silica gel column chromatography (hexane/CHCl_3_ = 1/1) to yield **4g** (2.5 mg, 0.0020 mmol, 6.4%) as a brown solid. The axle **17g** (14 mg, 0.010 mmol, 41% based on **3g**) was also isolated as a white solid. Data for **4g**: mp 187.9–188.6 °C; ^1^H NMR (500 MHz, CDCl_3_): δ 8.24 (d, *J* = 8.0 Hz, 2H), 8.03 (d, *J* = 7.5 Hz, 4H), 7.92 (d, *J* = 8.6 Hz, 2H), 7.75 (s, 2H), 7.58 (d, *J* = 8.0 Hz, 12H), 7.48 (d, *J* = 7.5 Hz, 12H), 7.40 (t, *J* = 6.9 Hz, 12H), 7.30–7.33 (m, 18H), 7.02 (d, *J* = 8.2 Hz, 4H), 6.98 (d, *J* = 8.0 Hz, 5H), 6.79 (d, *J* = 8.0 Hz, 4H), 6.55 (d, *J* = 8.6 Hz, 4H), 6.35 (d, *J* = 7.5 Hz, 3H), 5.11 (s, 2H), 3.95 (t, *J* = 6.3 Hz, 4H), 3.90 (s, 4H), 3.70 (t, *J* = 5.7 Hz, 4H), 1.76 (m, 8H); ^13^C{^1^H} NMR (126 MHz, CDCl_3_): δ 160.3, 159.6, 158.4, 146.7, 146.61, 146.55, 141.7, 140.8, 138.5, 136.7, 135.1, 133.0, 132.8, 131.9, 131.6, 130.3, 129.7, 128.9, 127.6, 127.4, 127.3, 127.1, 126.1, 125.9, 120.9, 120.1, 114.7, 112.2, 107.5, 100.9, 82.2, 74.2, 67.5, 67.4, 63.7, 48.1, 25.8, 25.5; IR (ATR): 3420, 3331, 1605, 1586, 1515, 1487 cm^–1^; HRMS (MALDI): calcd for C_142_H_111_N_4_O_4_ ([M + H]^+^), 1935.8600; found, 1935.8665. Data for **17g**: mp 249.7–250.9 °C;^1^H NMR (500 MHz, CDCl_3_): δ 7.59 (d, *J* = 8.0 Hz, 12H), 7.50–7.47 (m, 16H), 7.40 (t, *J* = 7.5 Hz, 12H), 7.34–7.29 (m, 22H), 7.08 (d, *J* = 8.6 Hz, 4H), 6.54 (d, *J* = 8.6 Hz, 4H), 4.33 (s, 4H), 4.08 (br, 2H); ^13^C{^1^H} NMR (100 MHz, CDCl_3_): δ 146.3, 145.8, 140.6, 140.4, 138.3, 135.9, 132.6, 132.4, 132.0, 131.5, 128.7, 127.3, 127.1, 126.9, 126.0, 122.2, 111.9, 93.9, 63.6, 48.2; IR (ATR): 3412, 1608, 1513, 1485 cm^–1^; HRMS (MALDI): calcd for C_104_H_77_N_2_ ([M + H]^+^), 1353.6081; found, 1353.6067.

#### [2]Rotaxane (**4h**) and Diyne (**17h**)

Following the general procedure A, **2** (77.3 mg, 0.10 mmol), **3h** (173 mg, 0.25 mmol), K_2_CO_3_ (138 + 138 mg, 1.0 + 1.0 mmol), and I_2_ (25.4 + 25.4 mg, 0.10 + 0.10 mmol) were used. The residue was purified by silica gel column chromatography (hexane/CHCl_3_ = 1/2) to yield **4h** (91 mg, 0.046 mmol, 46%) as a yellow solid. The axle **17h** (42 mg, 0.030 mmol, 80%, based on **3h**) was also isolated as a white solid. Data for **4h**: mp 214.2–216.6 °C; ^1^H NMR (500 MHz, CDCl_3_): δ 9.42 (s, 2H), 8.27 (d, *J* = 8.6 Hz, 2H), 7.97 (d, *J* = 8.0 Hz, 4H), 7.82 (s, 2H), 7.72 (d, *J* = 8.0 Hz, 2H), 7.58 (d, *J* = 8.0 Hz, 12H), 7.48–7.51 (m, 16H), 7.40 (t, *J* = 8.0 Hz, 12H), 7.27–7.35 (m, 26H), 7.10 (d, *J* = 8.6 Hz, 4H), 7.06 (t, *J* = 8.6 Hz, 1H), 6.93 (s, 1H), 6.57 (d, *J* = 8.6 Hz, 4H), 6.43 (dd, *J* = 2.3, 8.0 Hz, 2H), 4.04 (t, *J* = 5.7 Hz, 4H), 3.98 (t, *J* = 6.3 Hz, 4H), 1.83–1.87 (m, 8H); ^13^C{^1^H} NMR (126 MHz, CDCl_3_): δ 164.4, 160.5, 159.5, 159.1, 146.1, 145.9, 142.1, 140.5, 138.5, 136.6, 136.3, 135.3, 132.5, 132.0, 131.4, 131.1, 129.6, 129.5, 128.7, 127.6, 127.5, 127.2, 126.9, 126.1, 125.9, 124.2, 122.1, 119.6, 114.2, 107.3, 100.9, 81.8, 75.5, 67.3, 67.2, 63.9, 25.5, 25.3; IR (ATR): 1667, 1601, 1511, 1486 cm^–1^; HRMS (MALDI): calcd for C_142_H_107_N_4_O_6_ ([M + H]^+^), 1963.8185; found, 1963.8180. Data for **17h**: mp 260.6–262.6 °C;^1^H NMR (500 MHz, CDCl_3_): δ 7.83 (d, *J* = 8.6 Hz, 4H), 7.78 (s, 2H), 7.61 (d, *J* = 8.6 Hz, 4H), 7.60 (d, *J* = 8.0 Hz, 12H), 7.52 (d, *J* = 8.0 Hz, 12H), 7.41 (t, *J* = 7.5 Hz, 12H), 7.30–7.37 (m, 22H); ^13^C{^1^H} NMR (126 MHz, CDCl_3_): δ 164.7, 145.7, 143.3, 140.5, 138.7, 135.5, 135.3, 132.9, 131.8, 131.4, 128.7, 127.2, 127.1, 127.0, 126.3, 125.1, 119.4, 81.6, 76.1, 64.0; IR (ATR): 3439, 1682, 1600, 1519, 1487 cm^–1^; HRMS (MALDI): calcd for C_104_H_73_N_2_O_2_ ([M + H]^+^), 1381.5667; found, 1381.5610.

#### [2]Rotaxane (**4i**) and Diyne (**17i**)

Following the general procedure A, **2** (77.3 mg, 0.10 mmol), **3i** (179 mg, 0.25 mmol), K_2_CO_3_ (138 + 138 mg, 1.0 + 1.0 mmol), and I_2_ (25.4 + 25.4 mg, 0.10 + 0.10 mmol) were used. The residue was purified by silica gel column chromatography (hexane/CHCl_3_ = 1/2) to yield **4i** (98 mg, 0.048 mmol, 48%) as a white solid. The axle **17i** (62 mg, 0.043 mmol, 35% based on **3i**) was also isolated as a yellow solid. Data for **4i**: mp 219.2–225.7 °C; ^1^H NMR (500 MHz, CDCl_3_): δ 9.34 (s, 2H), 8.25 (d, *J* = 8.0 Hz, 2H), 7.91 (d, *J* = 8.0 Hz, 4H), 7.80 (s, 2H), 7.72–7.77 (m, 10H), 7.59 (d, *J* = 7.5 Hz, 12H), 7.52 (d, *J* = 8.6 Hz, 12H), 7.41 (t, *J* = 8.0 Hz, 12H), 7.30–7.36 (m, 26H), 7.16 (t, *J* = 2.3 Hz, 1H), 7.07 (t, *J* = 8.6 Hz, 1H), 6.48 (dd, *J* = 2.3, 8.6 Hz, 2H), 6.38 (d, *J* = 8.6 Hz, 4H), 4.14 (t, *J* = 6.3 Hz, 4H), 3.84 (t, *J* = 6.9 Hz, 4H), 1.84 (quint, *J* = 7.5 Hz, 4H), 1.77 (quint, *J* = 6.9 Hz, 4H); ^13^C{^1^H} NMR (126 MHz, CDCl_3_): δ 160.6, 159.2, 159.1, 147.0, 146.9, 146.7, 145.3, 140.4, 138.8, 136.5, 134.7, 133.0, 132.7, 131.8, 131.35, 131.28, 129.5, 129.4, 128.8, 127.4, 127.3, 126.9, 126.4, 125.8, 125.5, 121.5, 120.8, 120.5, 119.3, 114.1, 108.0, 100.6, 82.1, 74.6, 67.3, 67.1, 64.1, 25.24, 25.22; IR (ATR): 1601, 1586, 1516, 1483 cm^–1^; HRMS (MALDI): calcd for C_144_H_107_N_8_O_4_ ([M + H]^+^), 2011.8410; found, 2011.8481. Data for **17i**: mp 234.5–236.8 °C; ^1^H NMR (500 MHz, CDCl_3_): δ 8.19 (s, 2H), 7.88 (d, *J* = 6.9 Hz, 4H), 7.71 (d, *J* = 7.5 Hz, 4H), 7.61–7.60 (m, 16H), 7.56–7.53 (m, 16H), 7.42 (t, *J* = 7.5 Hz, 12H), 7.38 (d, *J* = 7.5 Hz, 12H), 7.33 (t, *J* = 7.5 Hz, 6H); ^13^C{^1^H} NMR (100 MHz, CDCl_3_): δ 148.0, 147.5, 145.2, 140.4, 139.0, 134.8, 133.1, 132.5, 131.4, 131.0, 128.8, 127.4, 127.0, 126.4, 125.7, 119.8, 118.0, 74.9, 64.2 (two signals are missing); IR (ATR): 1600, 1515, 1485 cm^–1^; HRMS (MALDI): calcd for C_106_H_73_N_6_ ([M + H]^+^), 1429.5891; found, 1429.5851.

### Synthesis of **4g** by the Removal of the Fmoc Group from **4e**

A solution of **4e** (72 mg, 0.030 mmol) in diethylamine (0.10 mL), acetonitrile (2.0 mL), and dichloromethane (8.0 mL) was stirred at rt under Ar for 4 h. To the mixture was added water, and the mixture was extracted with CH_2_Cl_2_ (3 × 3 mL). The combined organic layer was washed with brine, dried over Na_2_SO_4_, and concentrated in vacuo. The residue was purified by silica gel column chromatography (hexane/AcOEt = 1/1) and GPC to yield **4g** (49 mg, 0.025 mmol, 84%) as a white solid.

### Preparation and Observation of the ^1^H NMR Spectrum of **4f-*d***_**2**_

A solution of **4f** (27 mg, 0.014 mmol) in CH_3_OD (99 atom % D, 1.2 mL) and anhydrous dichloromethane (1.2 mL) was stirred at rt under Ar overnight. Volatiles were removed under reduced pressure to yield the desired deuterated compound, **4f-*d***_**2**_ (26 mg, 0.014 mmol, quint, 78 atom % D of the N–D bond, estimated by ^1^H NMR in CDCl_3_) as a light yellow solid.

In order to reduce the deuteration loss due to water and possible residual acidic impurities in the solvent used for recording NMR, it was imperative to include a simple pre-treatment for these solvents. NMR solvents (CDCl_3_ for confirming deuteration and deuterated toluene-*d*_8_ for VT NMR experiments) were thoroughly washed with equal volume of D_2_O followed by drying over sodium sulfate before use.

### X-ray Diffraction Studies

A suitable single crystal was selected in Fomblin Y perfluoropolyether (HVAC 140/13) at ambient temperature. All diffraction data were collected at −173 °C on a Bruker Apex II Ultra X-ray diffractometer equipped with a Mo Kα radiation (λ = 0.71073 Å) source. Intensity data were processed using the Apex3 software suite. The solution of the structures and the corresponding refinements were carried out using the Yadokari-XG^31^ graphical interface. The positions of the non-hydrogen atoms were determined by using the SHELXT-2014/5 and 2018/2^32^ program and refined on *F*^2^ by the full-matrix least-squares technique using the SHELXL-2018/3^33^ program. All non-hydrogen atoms were refined with anisotropic thermal parameters, while all hydrogen atoms were placed using AFIX instructions.

Compound **4a**(**a**): C_128_H_96_N_2_O_4_·2(toluene)·(hexane). Single crystals for X-ray diffraction were grown from toluene/hexane solution. The diffraction data are summarized in Table S1.

Compound **4a**(**b**): C_128_H_96_N_2_O_4_·CHCl_3_·(solvents). Single crystals for X-ray diffraction were grown from CHCl_3_/MTBE solution. Accessible voids were found in the unit cell. Attempts to model the solvent molecules (CHCl_3_, MTBE, and/or H_2_O) were not successful due to heavy disorder of the molecules. The diffuse electron density associated with the solvent molecules was removed by the PLATON/SQUEEZE^34^ program. The diffraction data are summarized in Table S2.

## Supporting Information Available

VT-NMR studies, copies of ^1^H and ^13^C NMR spectra of new compounds, 2D NMR spectra, the diffraction data, HRMS data of [2]rotaxanes, and X-ray data for **4a**(**a**) and **4a**(**b**) (PDF)

## Accession Codes

CCDC 2119495–2119496 contain the supplementary crystallographic data for this paper. These data can be obtained free of charge via www.ccdc.cam.ac.uk/data_request/cif, or by emailing data_request@ccdc.cam.ac.uk, or by contacting The Cambridge Crystallographic Data Centre, 12 Union Road, Cambridge CB2 1EZ, UK; fax: +44 1223 336033.
