# Supplementary material for: Conformational Control of [2]Rotaxane by Hydrogen Bond
Source: J Org Chem. 2022 Apr 7;87(9):5744–59. doi: 10.1021/acs.joc.2c00086 (PMC9087201; doi:10.1021/acs.joc.2c00086)

Supporting Information

Conformational Control of [2]Rotaxane by Hydrogen Bond

Yusuke Kawasaki,[a] Showkat Rashid,[a] Katsuhiko Ikeyatsu,[a] Yuichiro Mutoh,[a,#] Yusuke Yoshigoe,[a] Shoko Kikkawa,[b] Isao Azumaya,[b] Shoichi Hosoya,[c] and Shinichi Saito[*, a]

[a]*Department of Chemistry, Faculty of Science, Tokyo University of Science, 1-3 Kagurazaka, Shinjuku, Tokyo 162-8601, Japan*

[b]*Faculty of Pharmaceutical Sciences, Toho University, 2-2-1 Miyama, Funabashi, Chiba 274-8510, Japan*

[c] *Research Center for Medical and Dental Sciences, Tokyo Medical and Dental University, 1-5-45 Yushima, Bunkyo-ku, Tokyo 113-8510, Japan*

*Email: ssaito@rs.tus.ac.jp

Contents

1. VT ^{1}H NMR spectra of **4c**, **4f**, and **4h**. S2
2. Prediction of chemical shift of NH signal of **4h** and calculation of activation free energy at the coalescence point. S4
3. Copies of ^{1}H and ^{13}C{^{1}H} NMR spectra of **2-17** S5
4. 2D NMR spectra S39
5. Data for X-ray analysis
   5.1 Crystal data and structure refinement for **4a**(a) S46
   5.2 Crystal data and structure refinement for **4a**(b) S47

6. Mass Spectra of [2]Rotaxanes S48

1. VT ^{1}H NMR spectra

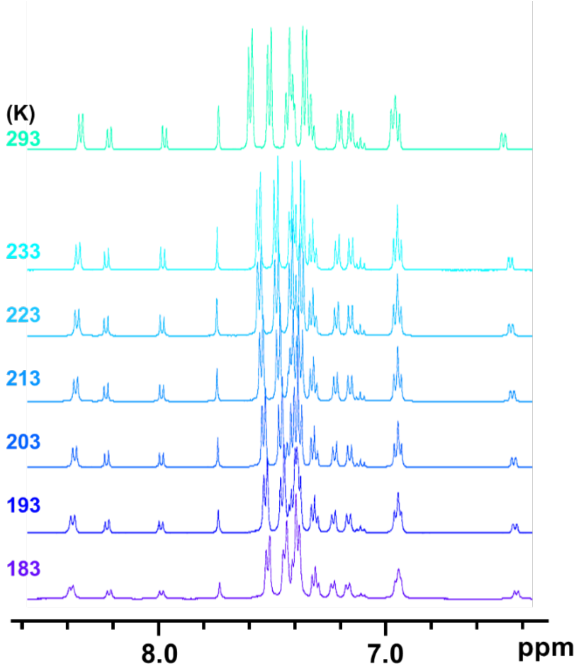

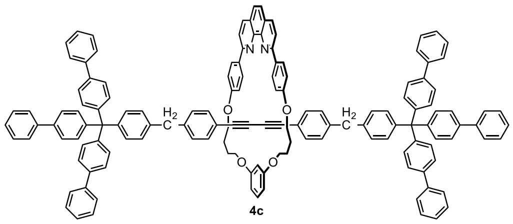

Figure S1. VT ^{1}H NMR spectra of 4c (500 MHz, CD_2Cl_2)

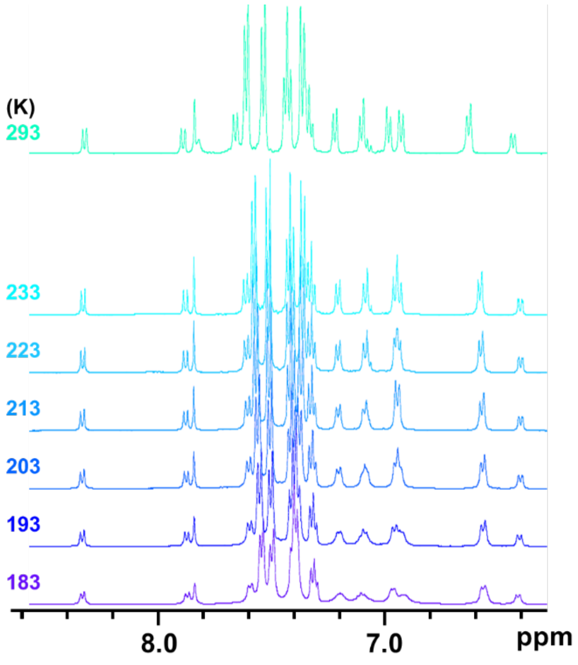

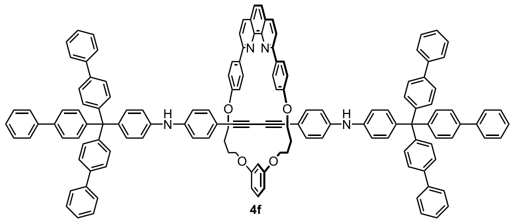

Figure S2. VT ^{1}H NMR spectra of 4f (500 MHz, CD_2Cl_2)

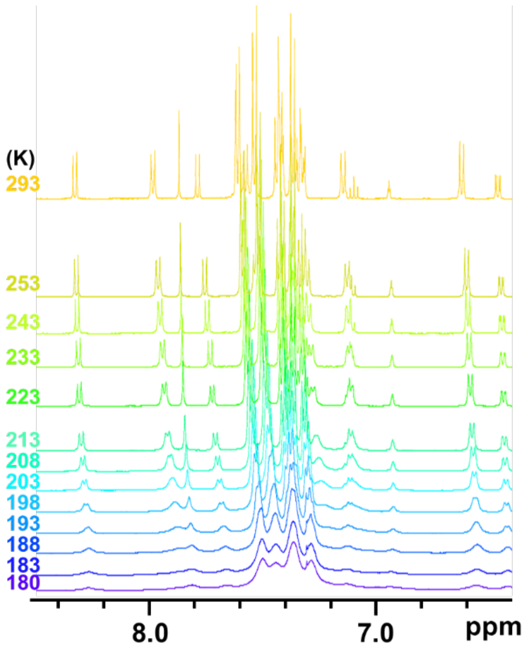

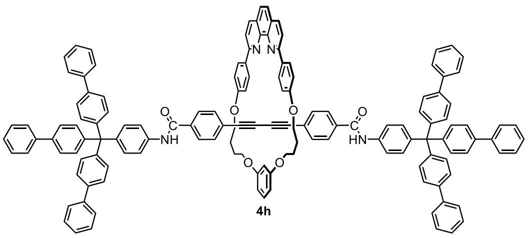

Figure S3. VT ^{1}H NMR spectra of 4h (500 MHz, CD_2Cl_2)

2. Prediction of chemical shift of NH signal of 4h and calculation of activation free energy at the coalescence point.

In compound **4h**, two non-equivalent signals of NH proton should be observed at temperatures below the coalescence point. However, at 180 K, one of the signals in higher magnetic field could not be observed due to the overlap with other signals. Therefore, the difference in chemical shifts of signals that should be observed non-equivalently at 180 K was predicted by the following procedure. First, very good agreement was found when fitting the chemical shift values of the signals of the equivalent amide protons observed at higher temperatures than the coalescence point as a quadratic function of temperature.

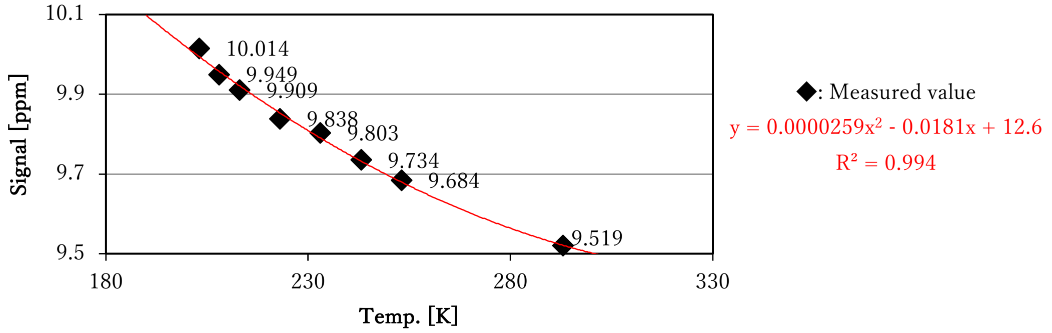

Then, the average value of the chemical shift of the inequivalent signal observed at 180 K ([2] in Table S1) was predicted by extrapolation using this function. The difference between this value and the chemical shift of the measured non-equivalent amide proton signal ([1] in the table) was doubled, predicting the chemical shift difference of the non-equivalent signals. The activation free energy of shuttling process at the coalescence point (8.28 ± 0.22 kcal/mol; T_c = 75 K ± 5 K) was calculated based on the following equation:

$$k_c = \pi \times \Delta\nu/\sqrt{2}$$

$$\Delta G^{\ddagger} = -RT_c \ln(k_c \times h/k_B \times T_c)$$

$$R = 1.987 \text{ cal·K}^{-1}\text{·mol}^{-1}$$

$$h = 6.626 \times 10^{-34} \text{ J·Hz}^{-1}$$

$$k_B = 1.381 \times 10^{-23} \text{ J·K}^{-1}$$

Table S1.

<Measured>	Temp. [K]	Chemical shift	
Chemical shift of equivalent signals measured at 293-203 K (The amide protons are magnetically equivalent)	293	9.519	[ppm]
	253	9.684	[ppm]
	243	9.734	[ppm]
	233	9.803	[ppm]
	223	9.838	[ppm]
	213	9.909	[ppm]
	208	9.949	[ppm]
	203	10.014	[ppm]
One of non-equivalent signals measured at 180 K (observed in lower magnetic field)	180	11.653	[ppm] ---[1]
**<Calculated>**	**Temp. [K]**	**Chemical shift**, $\Delta\nu$, $\Delta G^{\ddagger}$	
Expected average chemical shift of the non-equivalent signals (at 180 K)	180	10.317	[ppm] ---[2]
$\Delta\nu$[ppm] --- [1] – [2]		1.336	[ppm]
$\Delta\nu$ [Hz] *500.16 MHz		1336	[Hz]
The calculated $\Delta G^{\ddagger}$ for the shuttling process	75±5	8.28±0.22	[kcal/mol]

3. Copies of ^{1}H and ^{13}C[^{1}H] NMR spectra of 2-17

^{1}H NMR Spectrum of **2** ($CDCl_3$, 400 MHz).

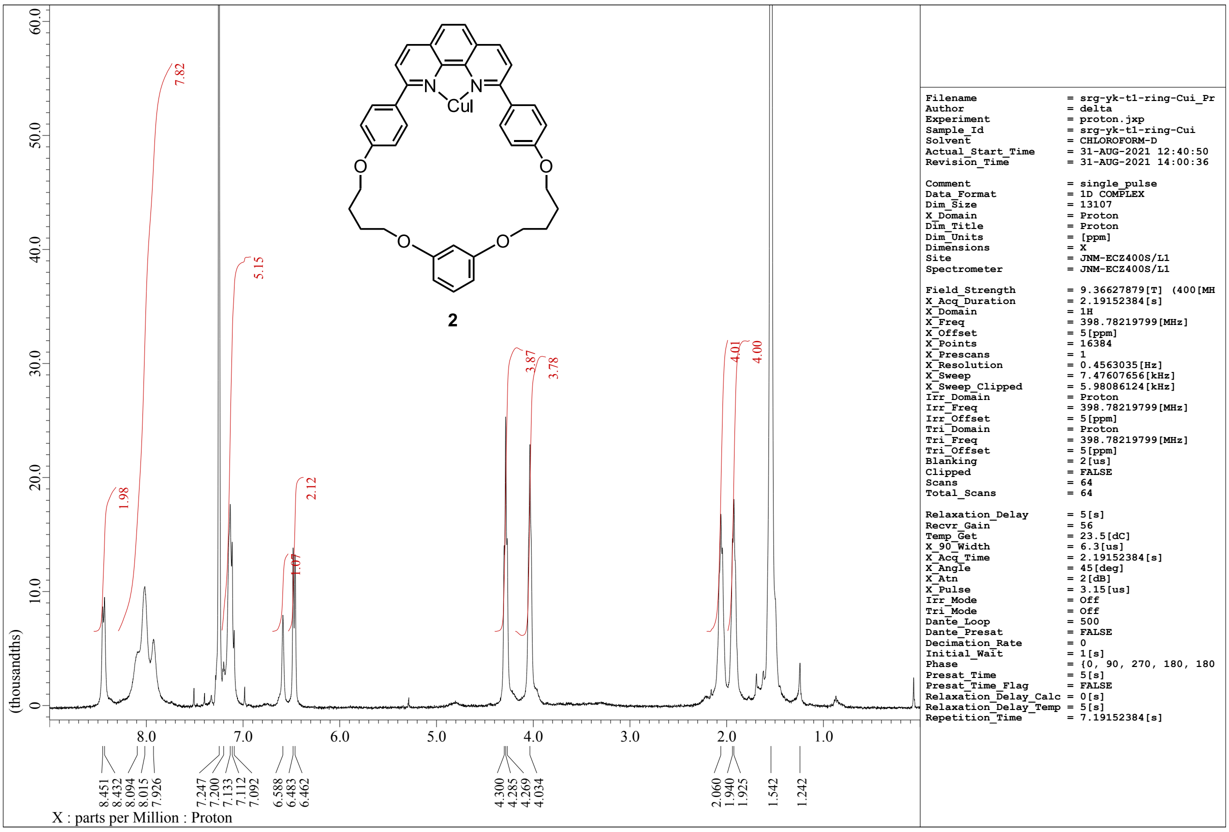

^{13}C{^{1}H} NMR Spectrum of **2** (DMSO–d_6, 100 MHz, 423 K).

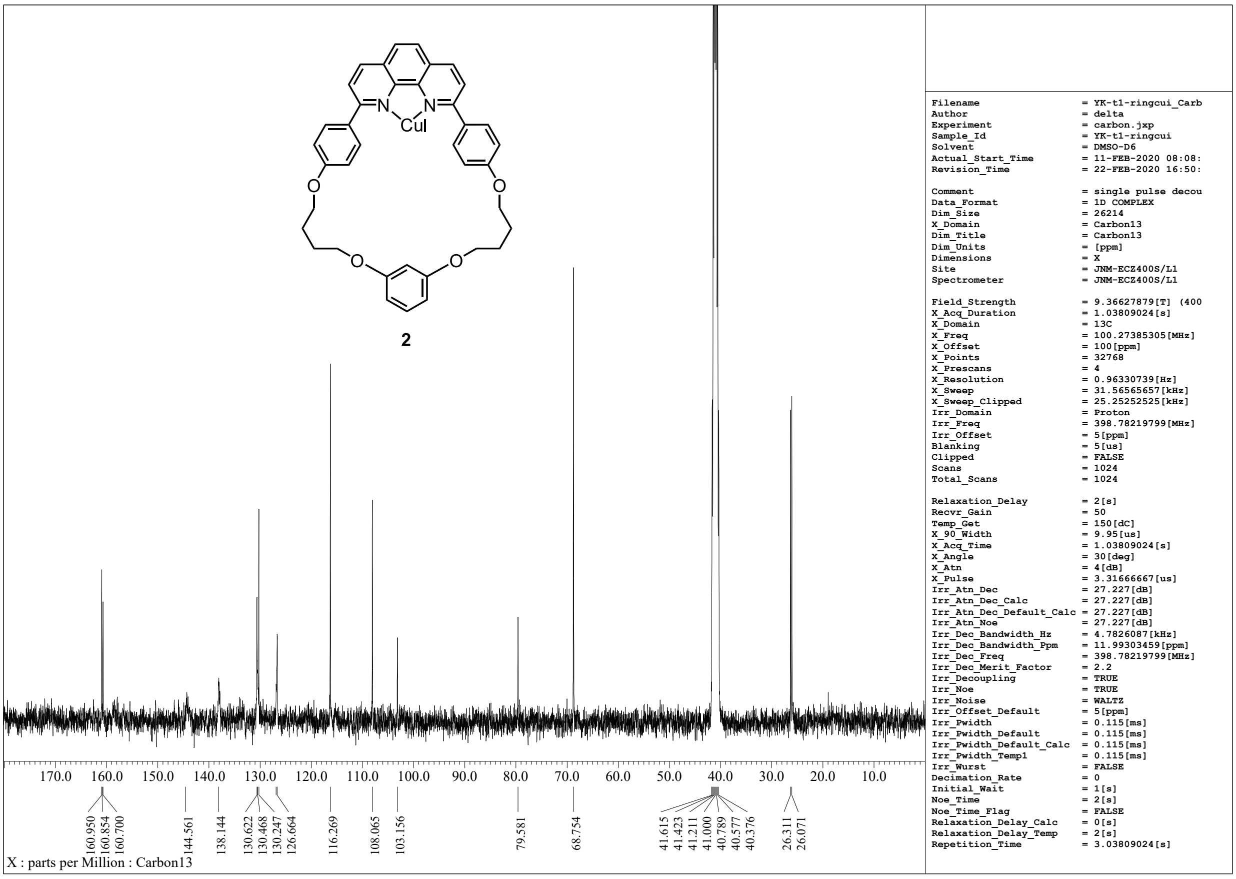

^{1}H NMR Spectrum of **6** ($CDCl_3$, 500 MHz).

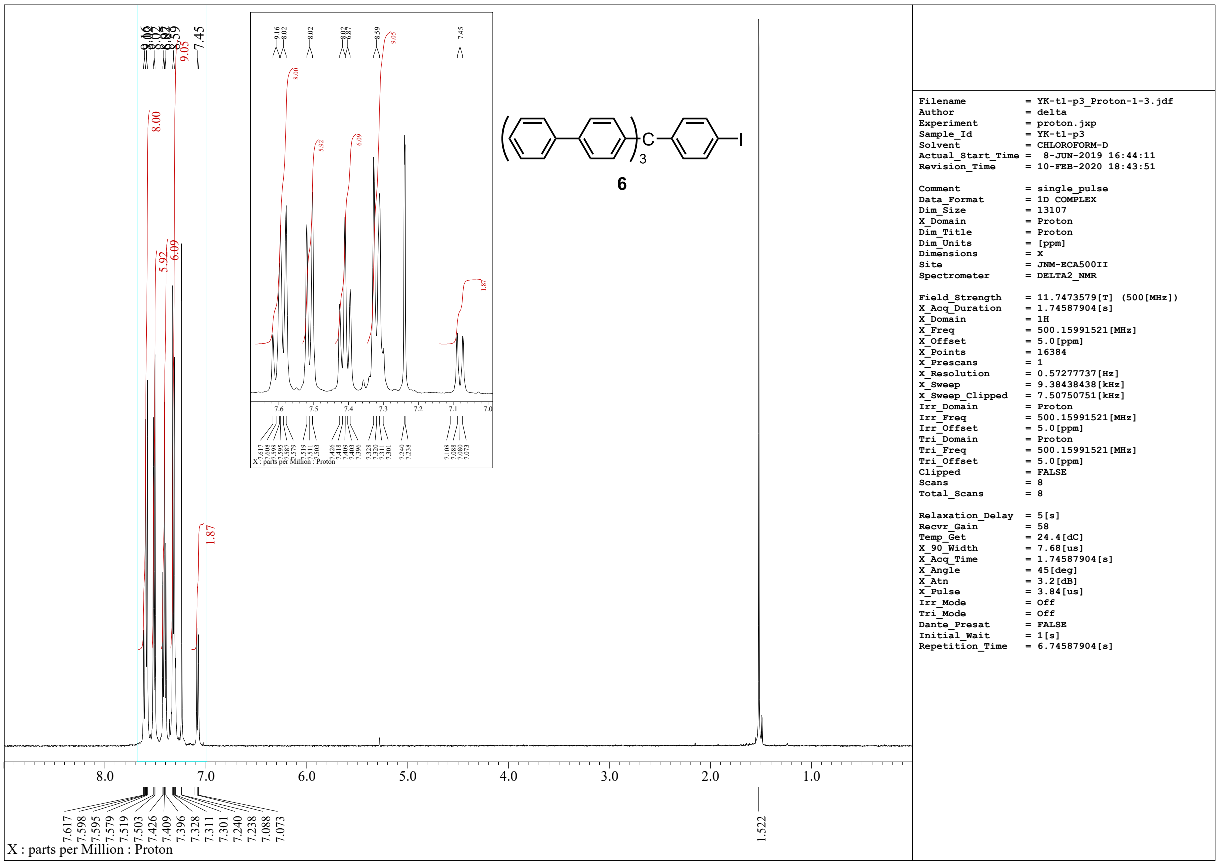

$^{13}C\{^{1}H\}$ NMR Spectrum of **6** ($CDCl_3$, 100 MHz).

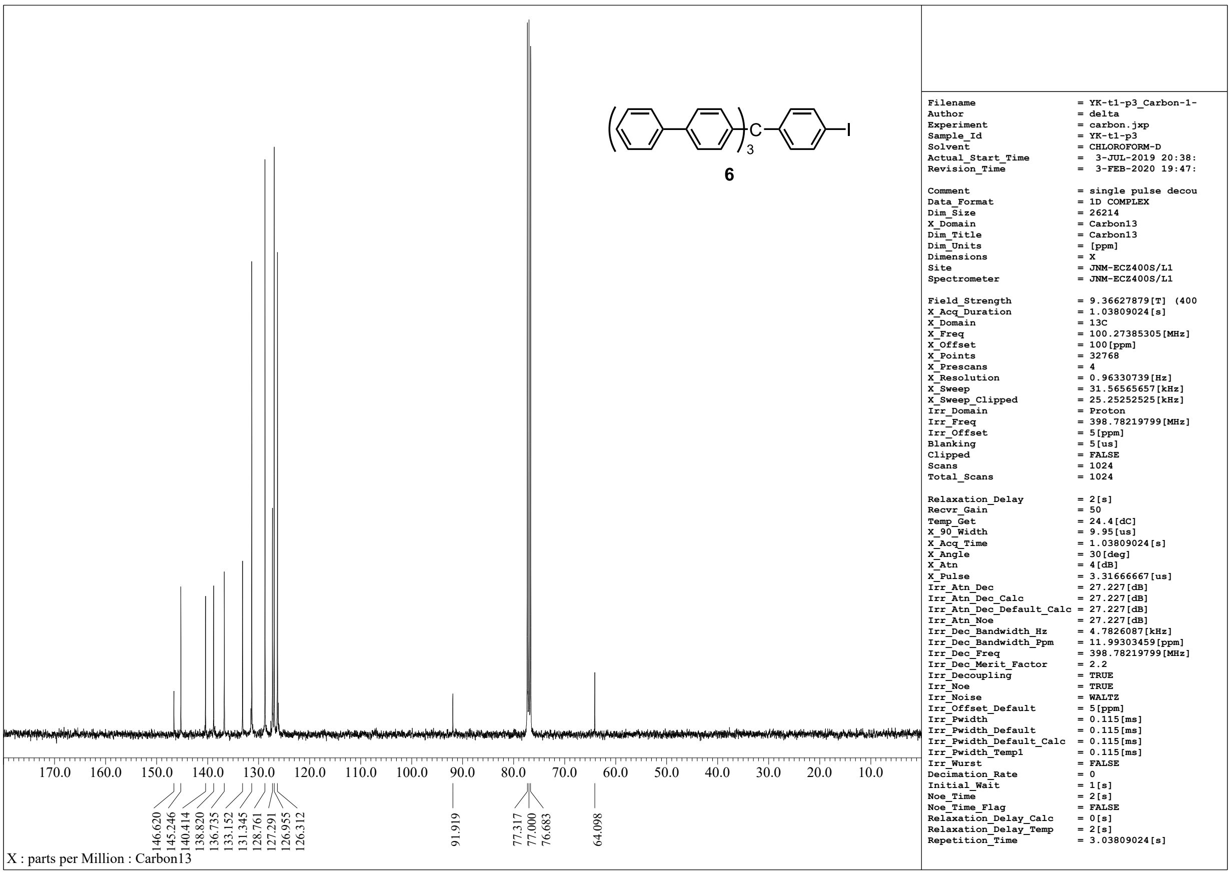

^{1}H NMR Spectrum of **3a** ($CDCl_3$, 500 MHz).

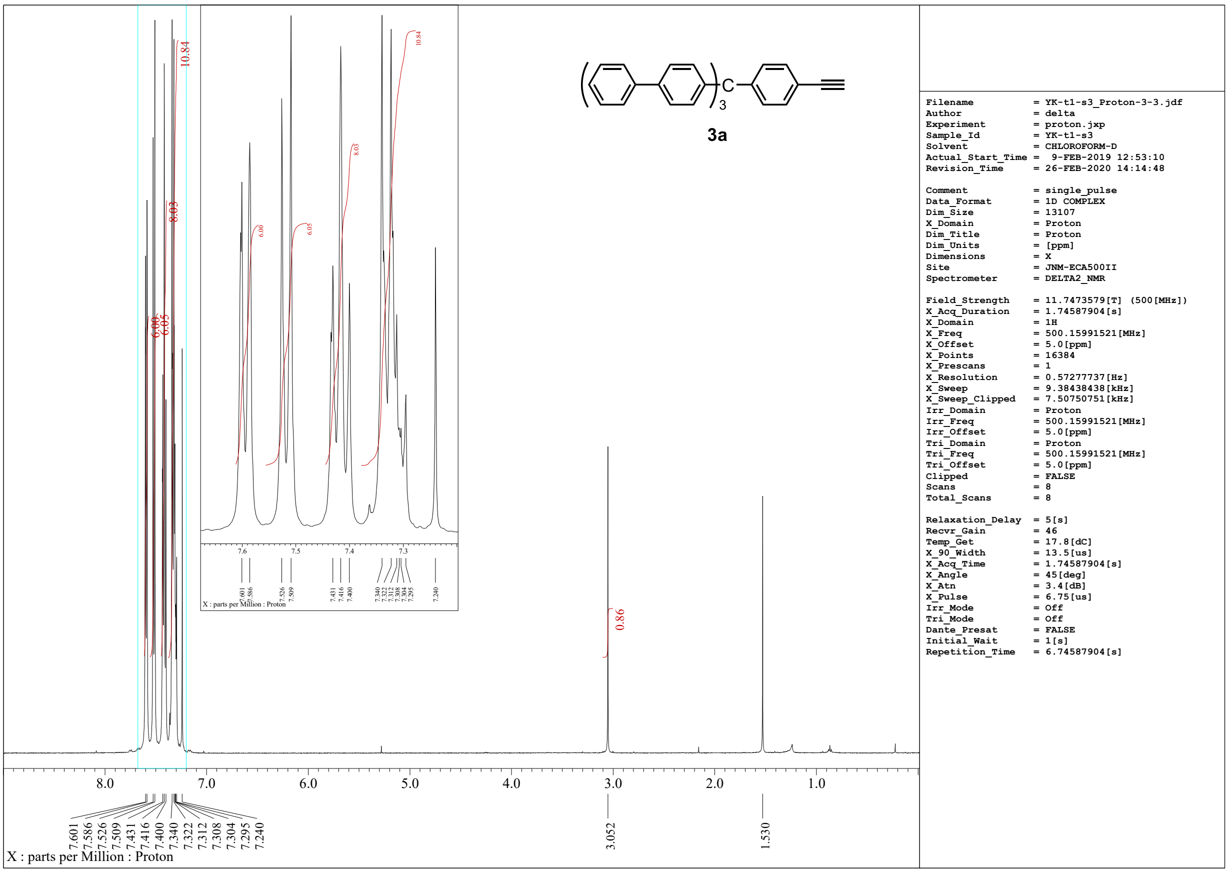

$^{13}C\{^{1}H\}$ NMR Spectrum of **3a** ($CDCl_3$, 126 MHz).

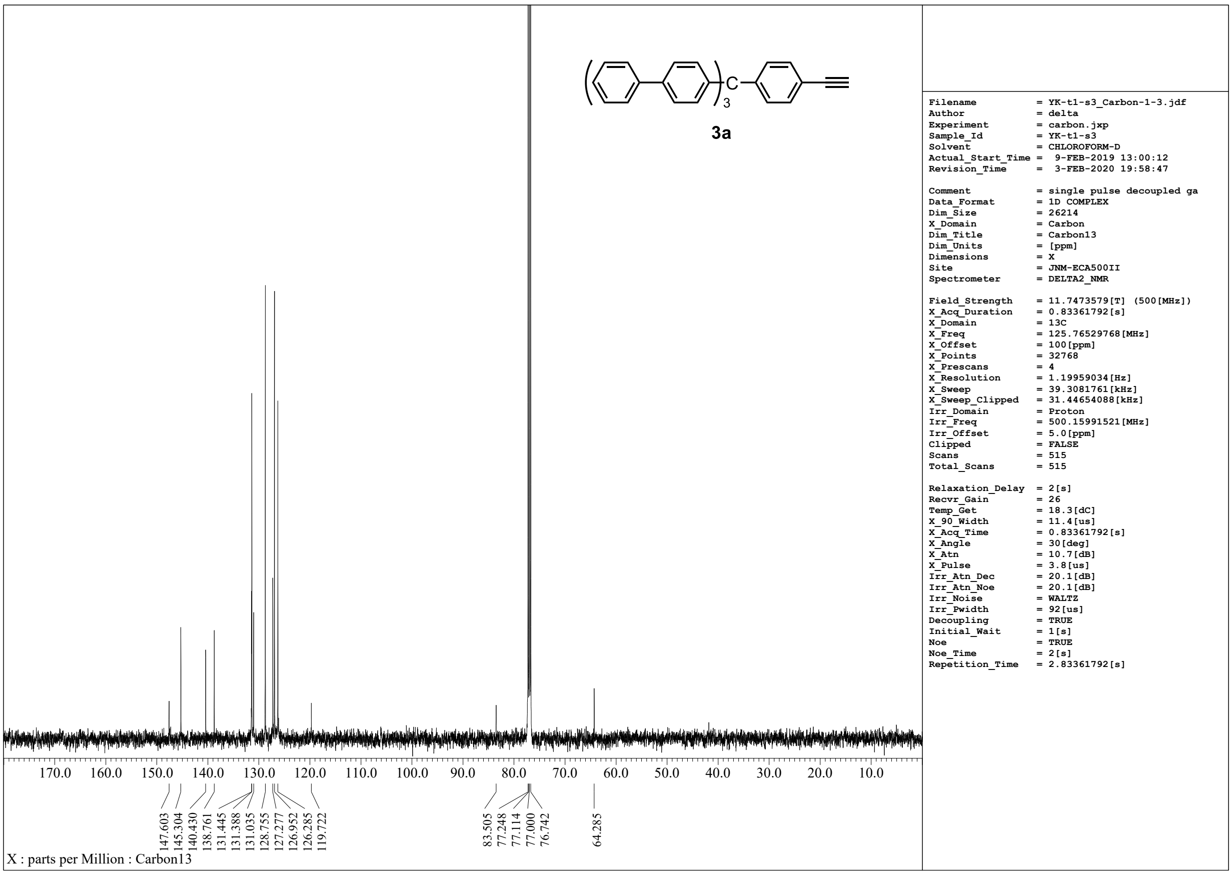

^{1}H NMR Spectrum of **3b** ($CDCl_3$, 500 MHz).

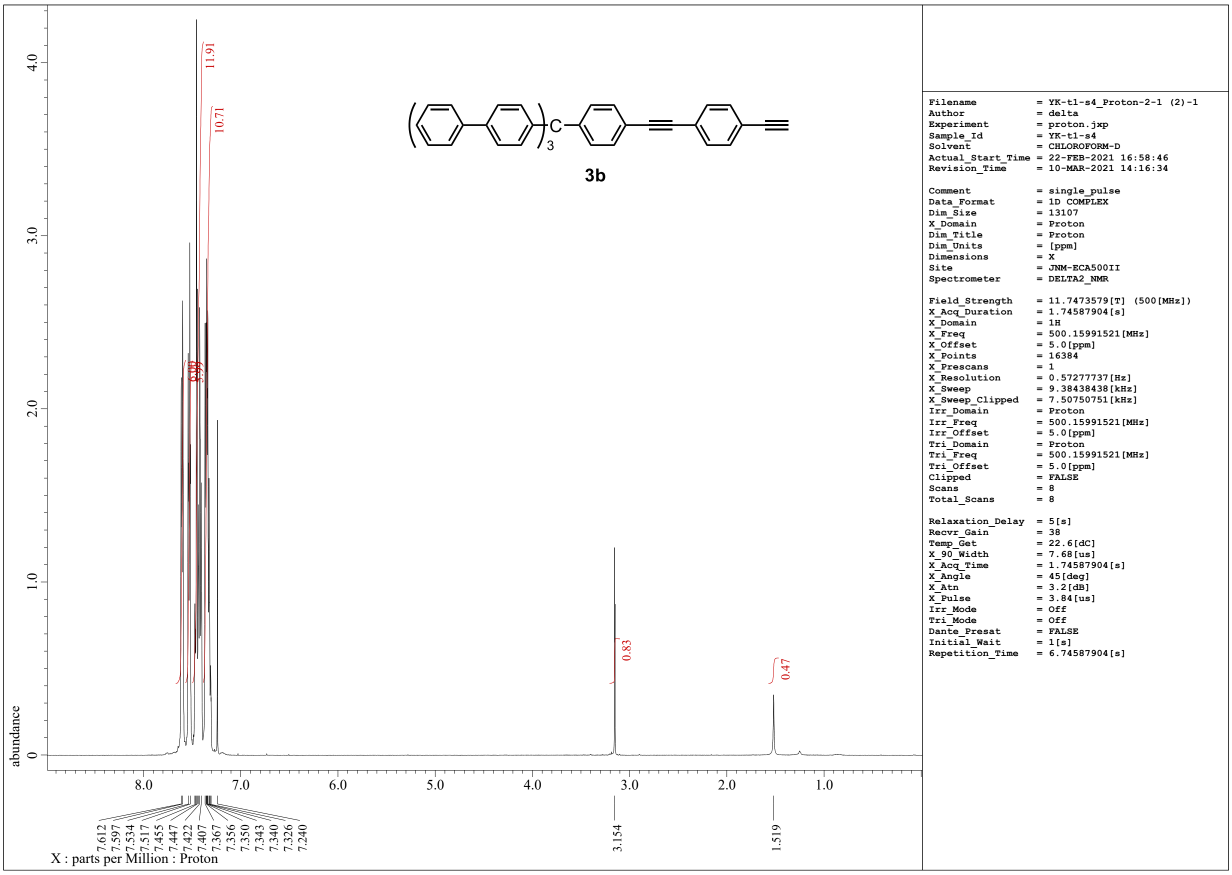

$^{13}C\{^{1}H\}$ NMR Spectrum of **3b** ($CDCl_3$, 126 MHz).

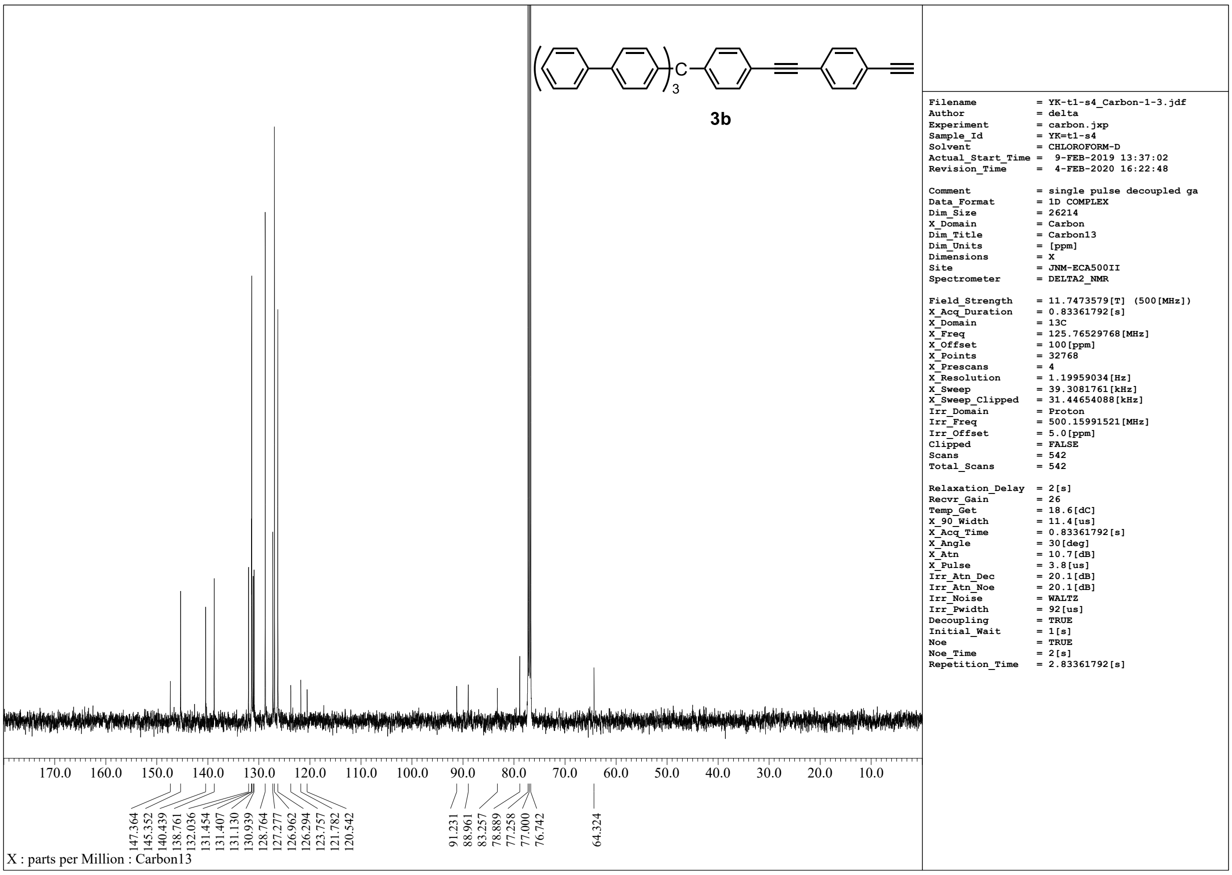

^{1}H NMR Spectrum of **8** ($CDCl_3$, 500 MHz).

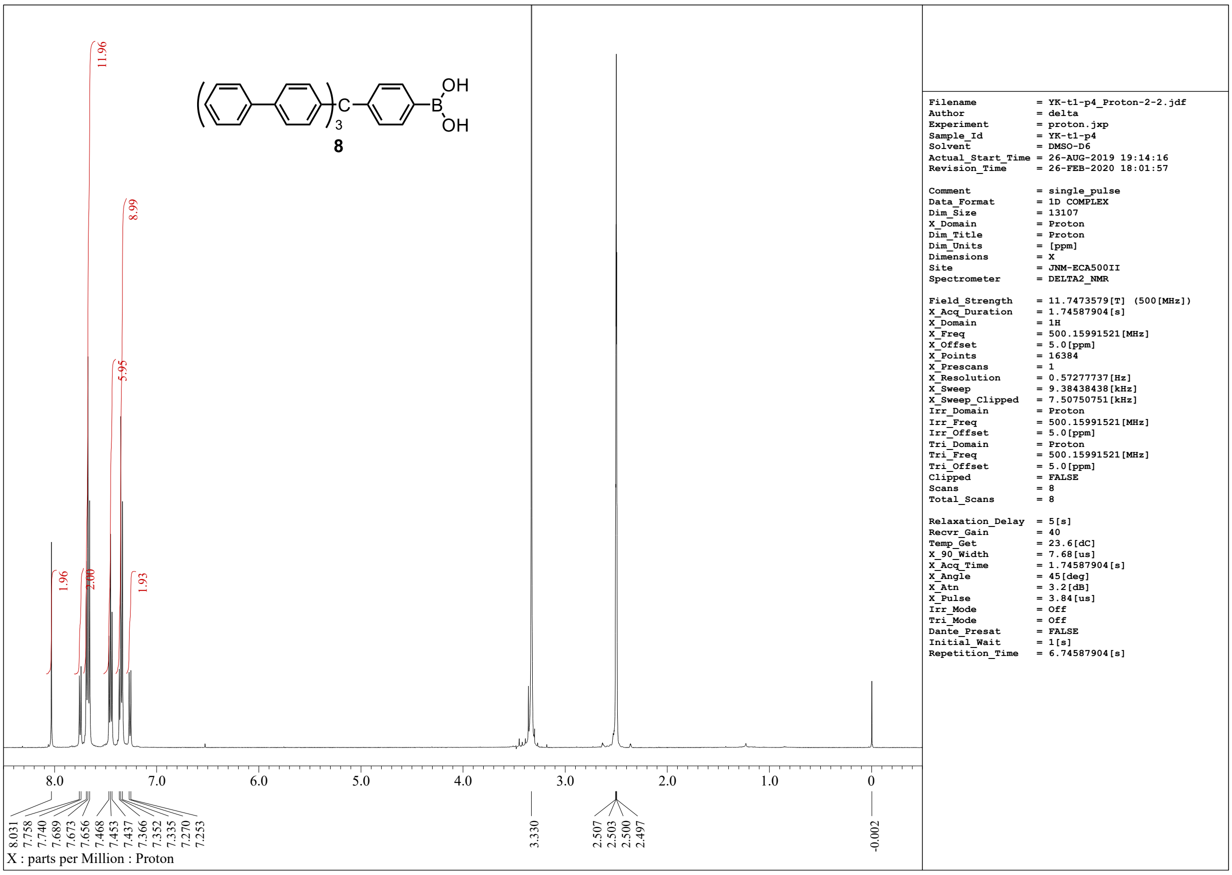

$^{13}C\{^{1}H\}$ NMR Spectrum of **8** ($CDCl_3$, 126 MHz).

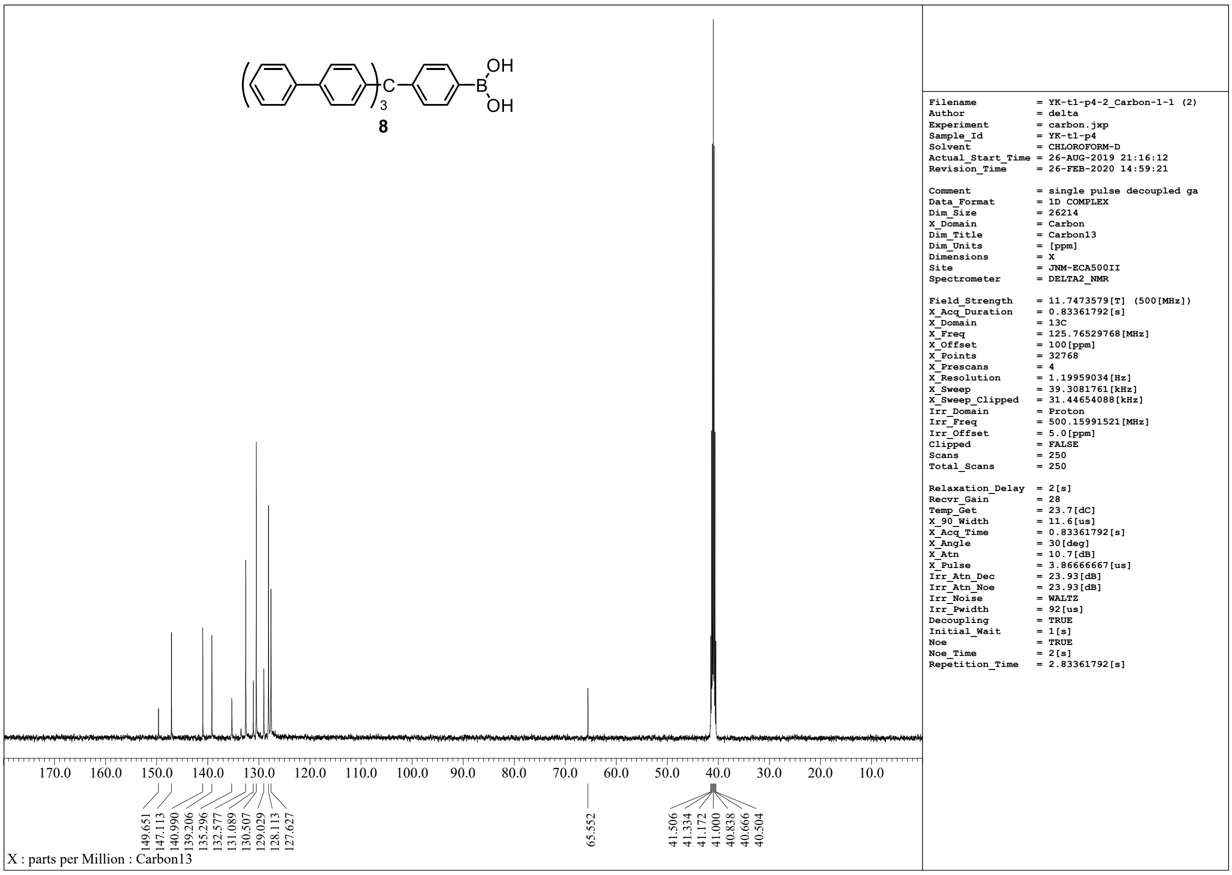

^{1}H NMR Spectrum of **10** ($CDCl_3$, 500 MHz).

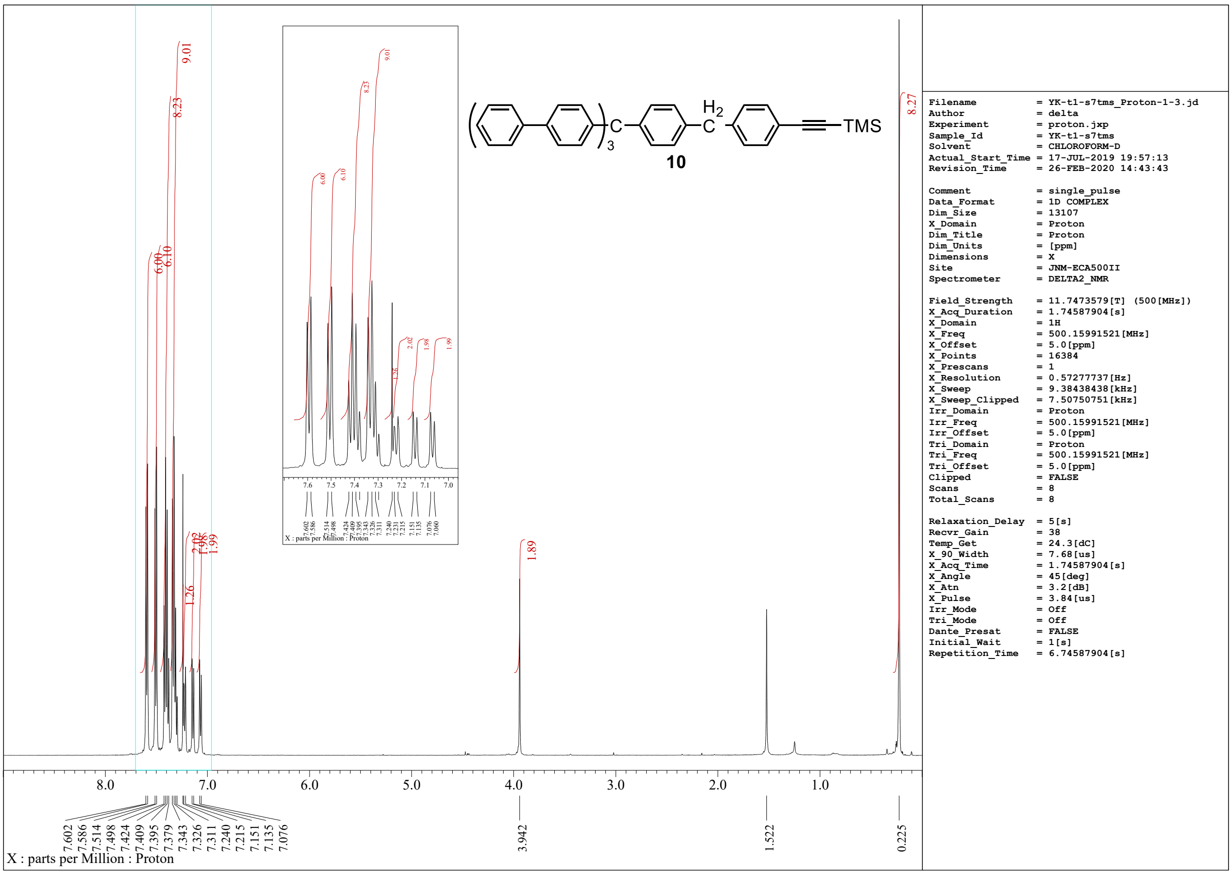

^{13}C{^{1}H} NMR Spectrum of **10** ($CDCl_3$, 100 MHz).

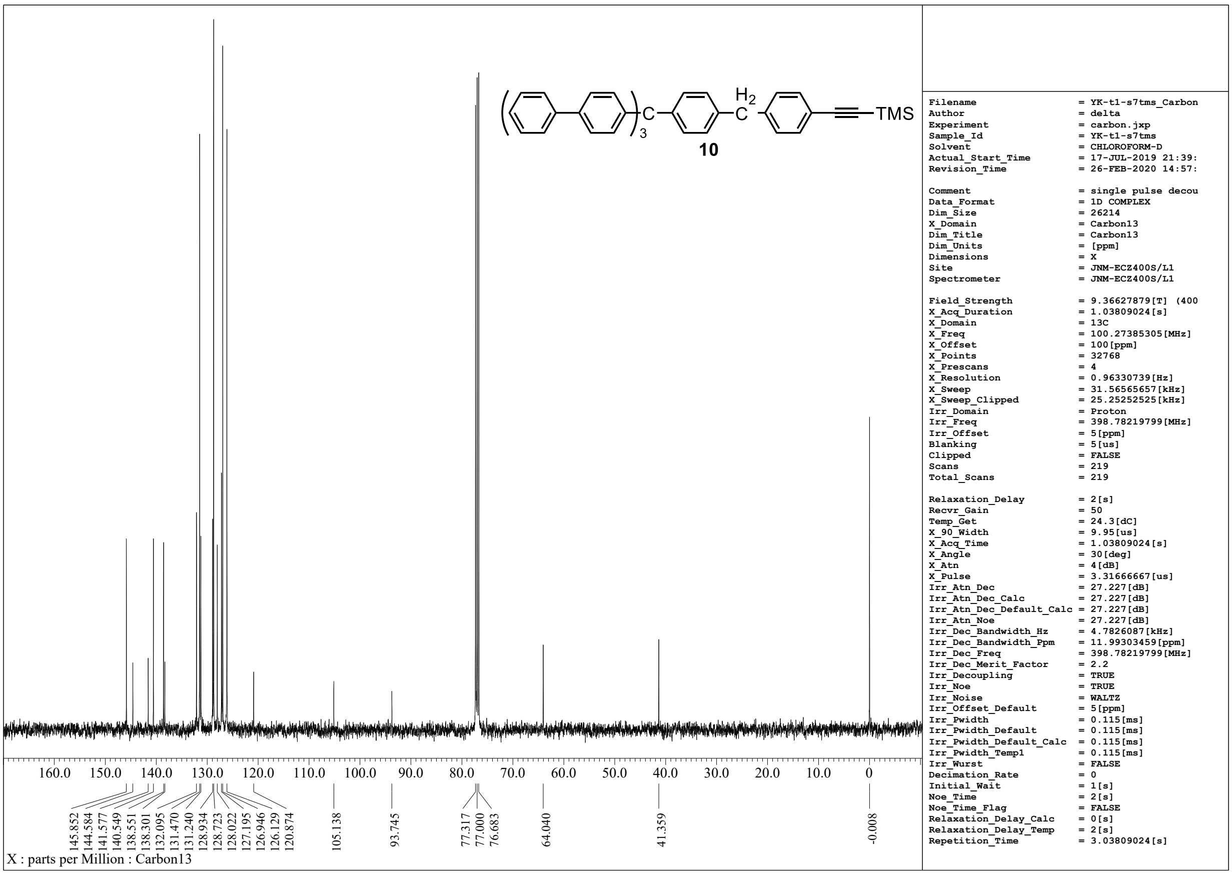

^{1}H NMR Spectrum of **3c** ($CDCl_3$, 500 MHz).

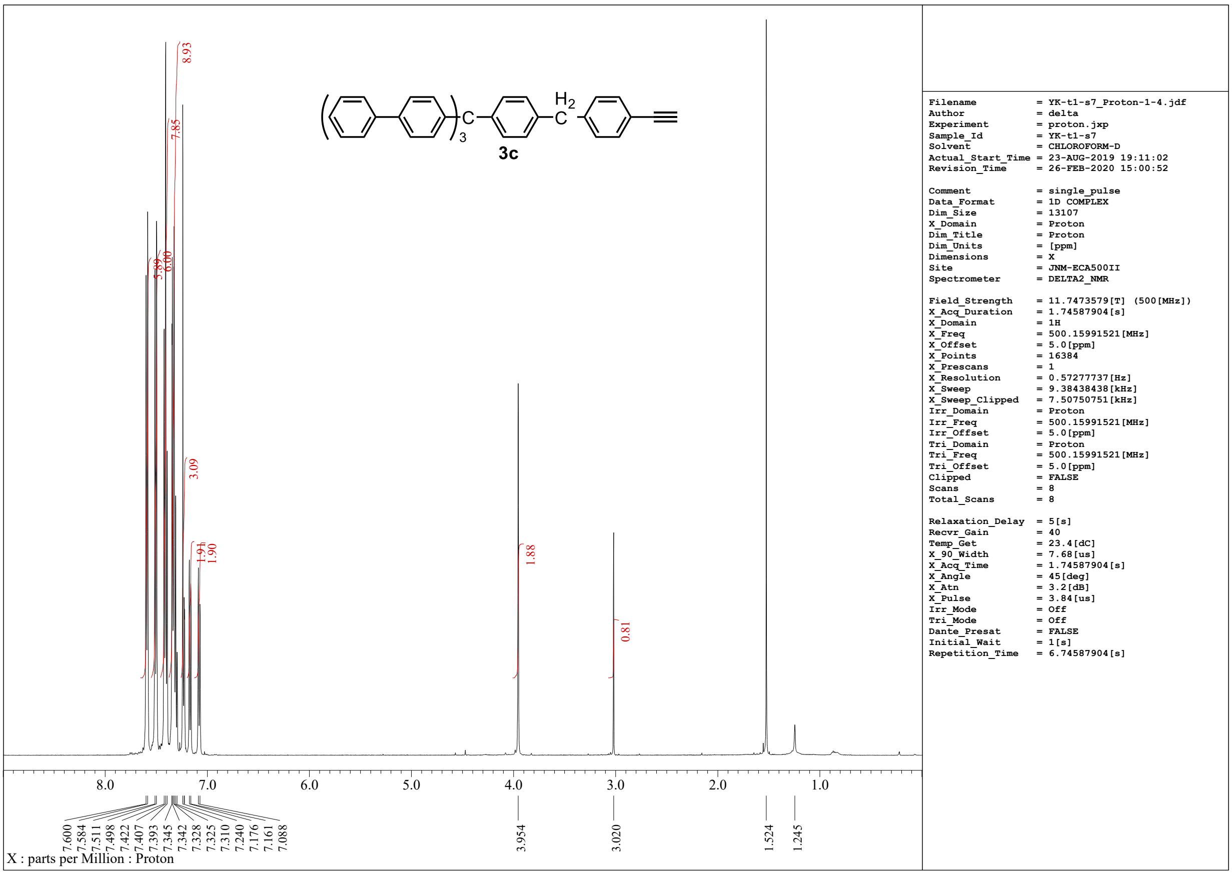

^{13}C{^{1}H} NMR Spectrum of **3c** ($CDCl_3$, 100 MHz).

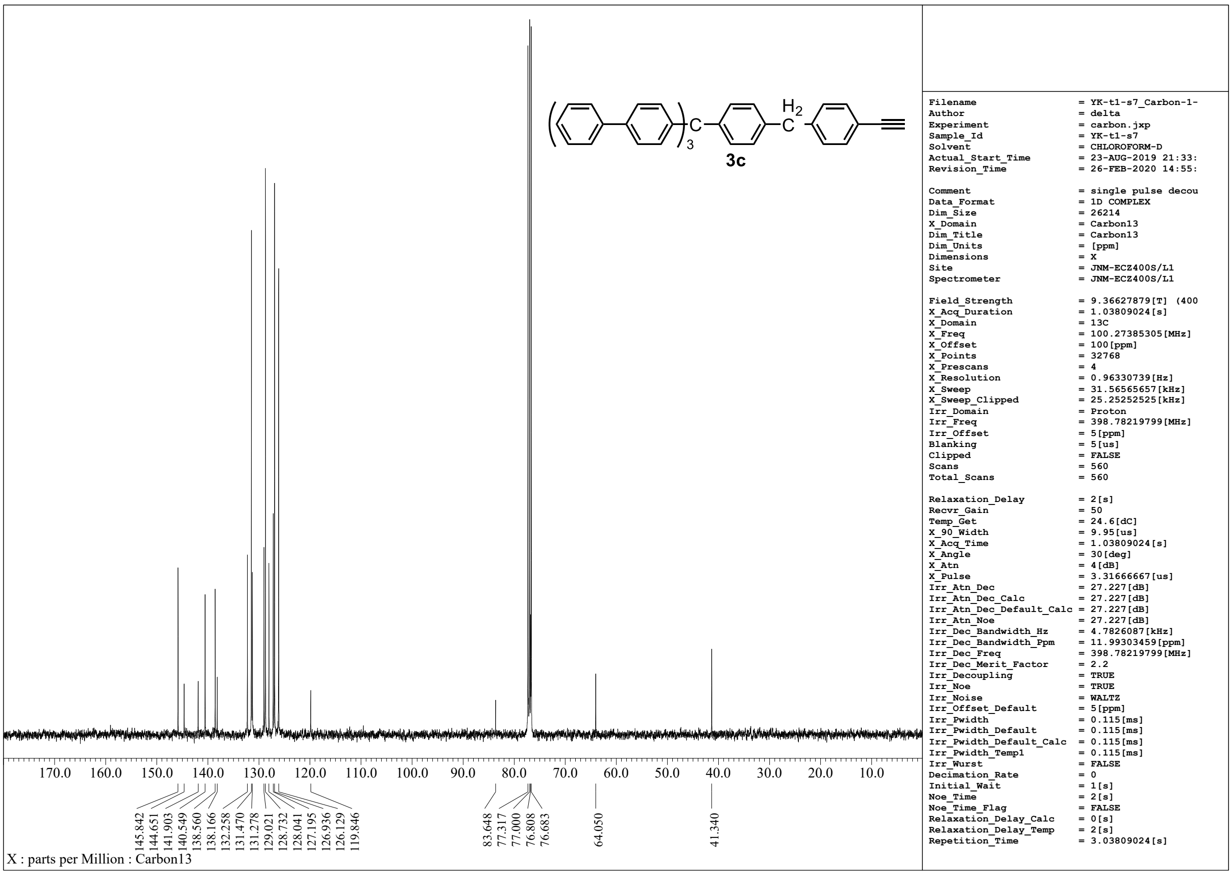

^{1}H NMR Spectrum of **11** ($CDCl_3$, 500 MHz).

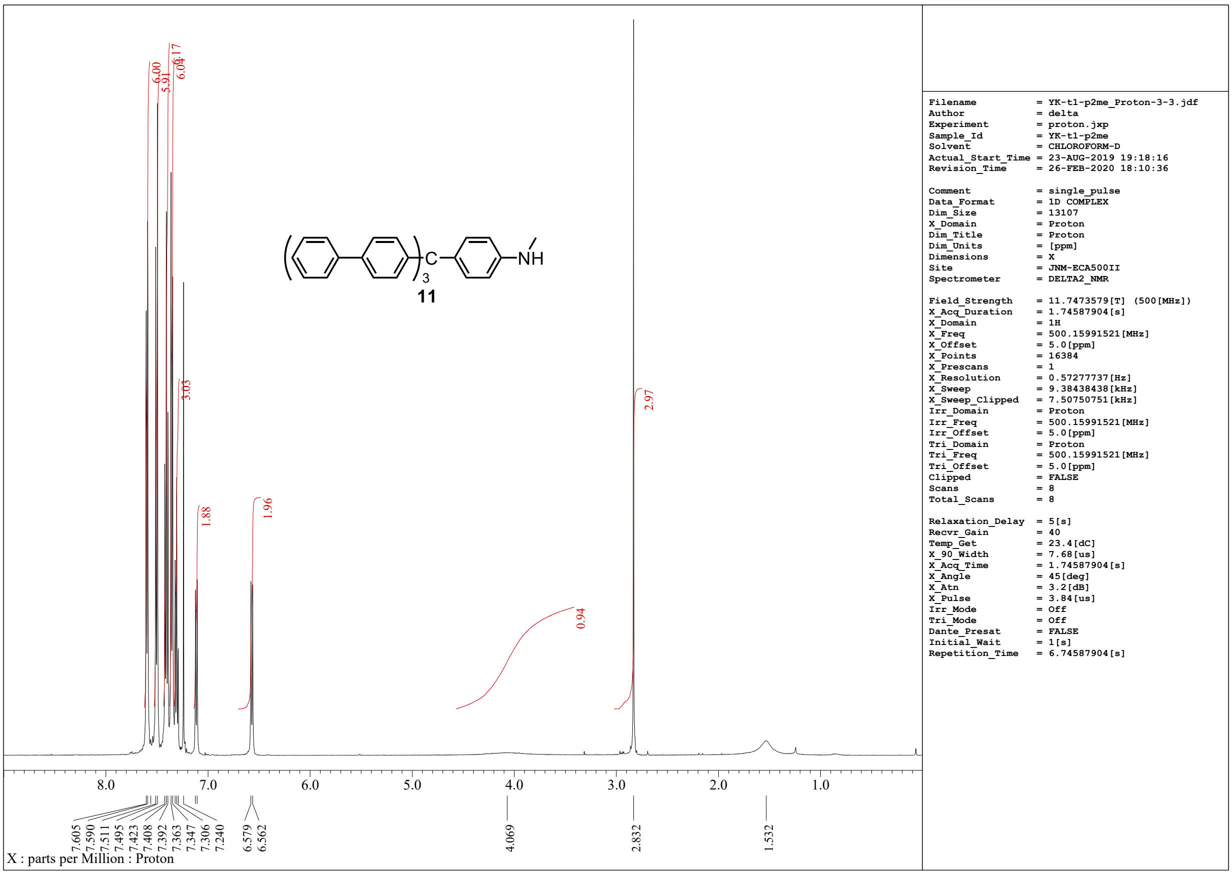

$^{13}C\{^{1}H\}$ NMR Spectrum of **11** ($CDCl_3$, 100 MHz).

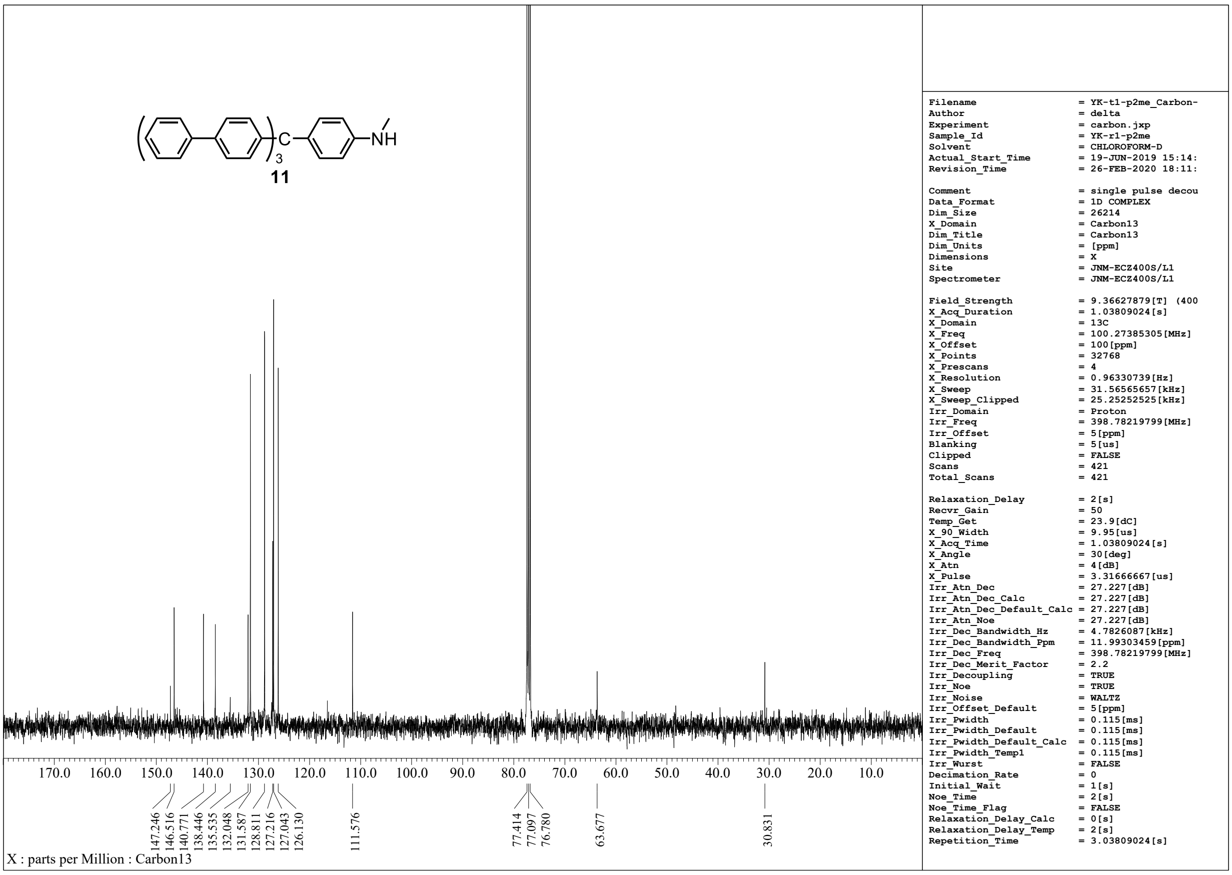

^{1}H NMR Spectrum of **13** ($CDCl_3$, 500 MHz).

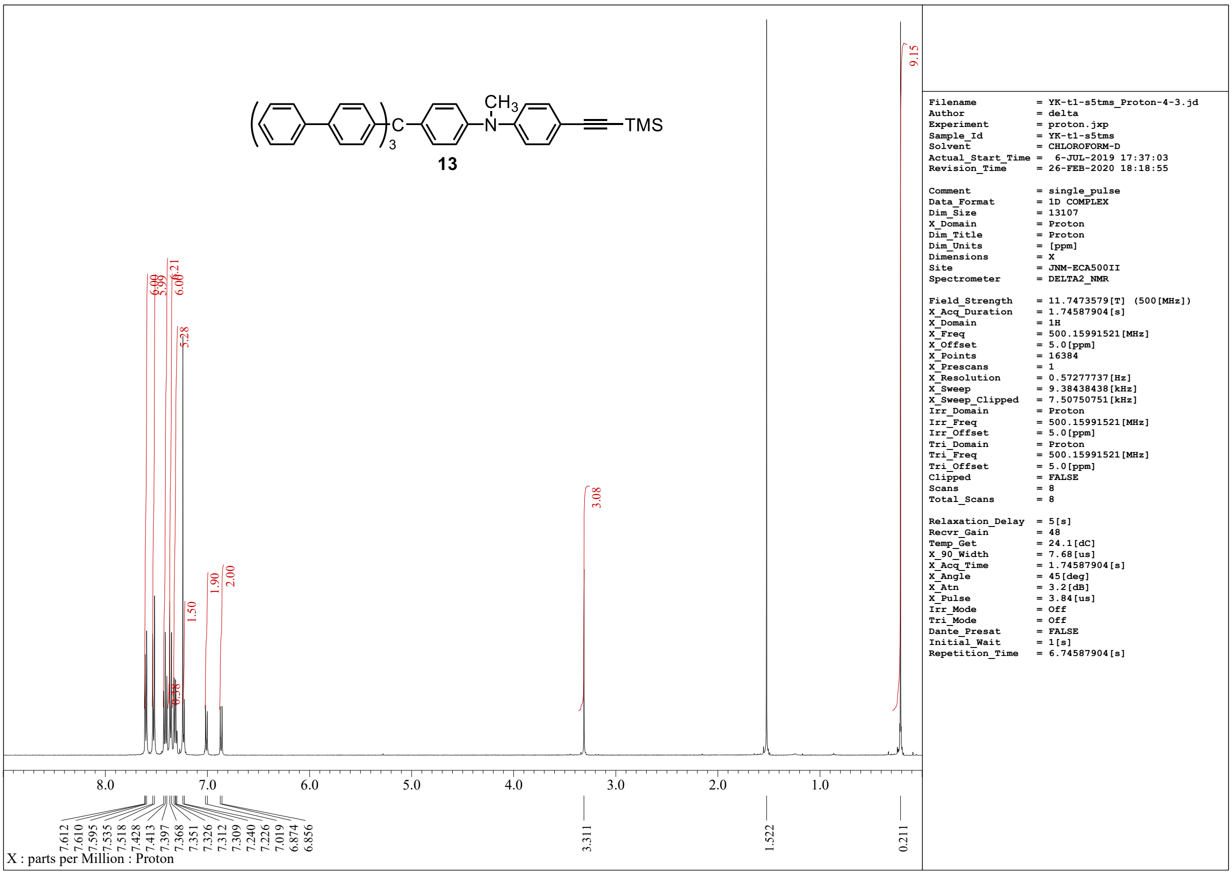

$^{13}C\{^{1}H\}$ NMR Spectrum of **13** ($CDCl_3$, 100 MHz).

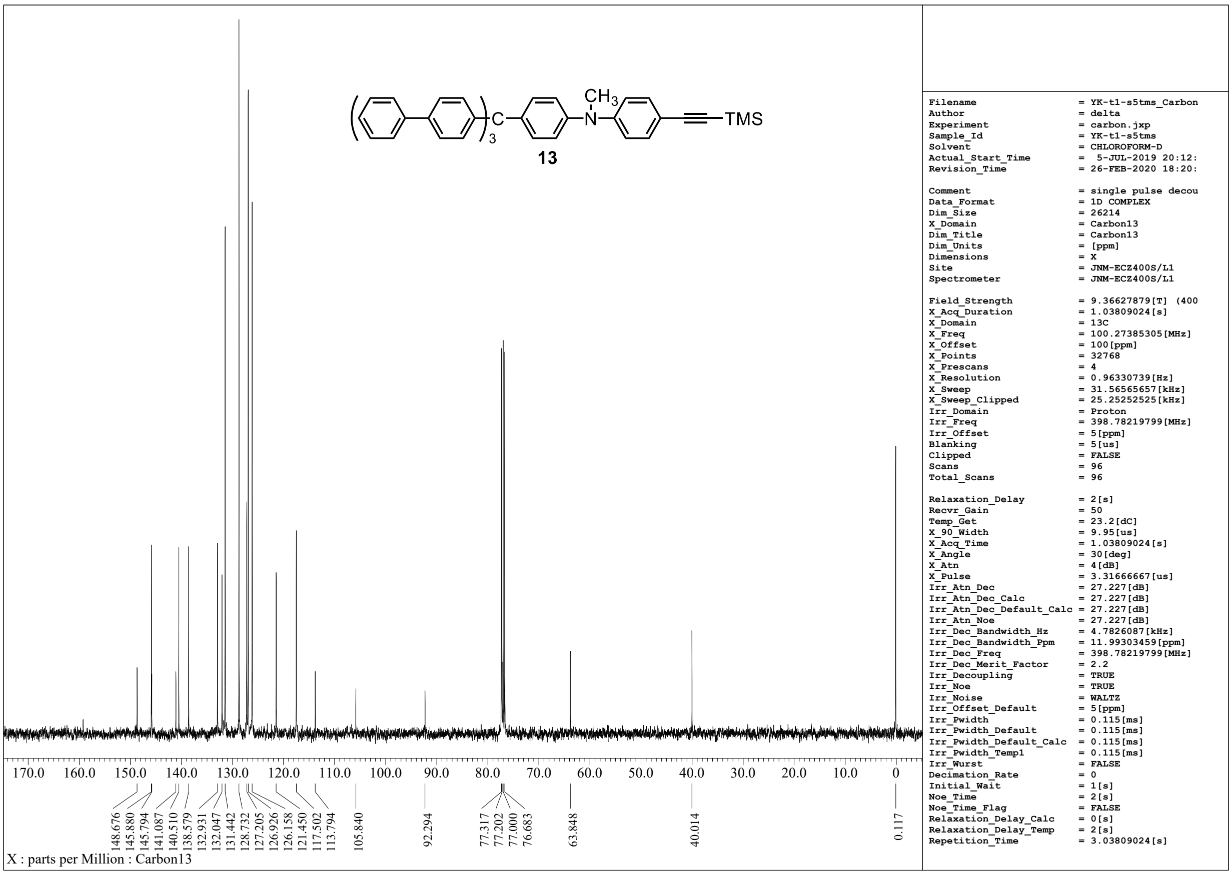

^{1}H NMR Spectrum of **14** ($CDCl_3$, 500 MHz).

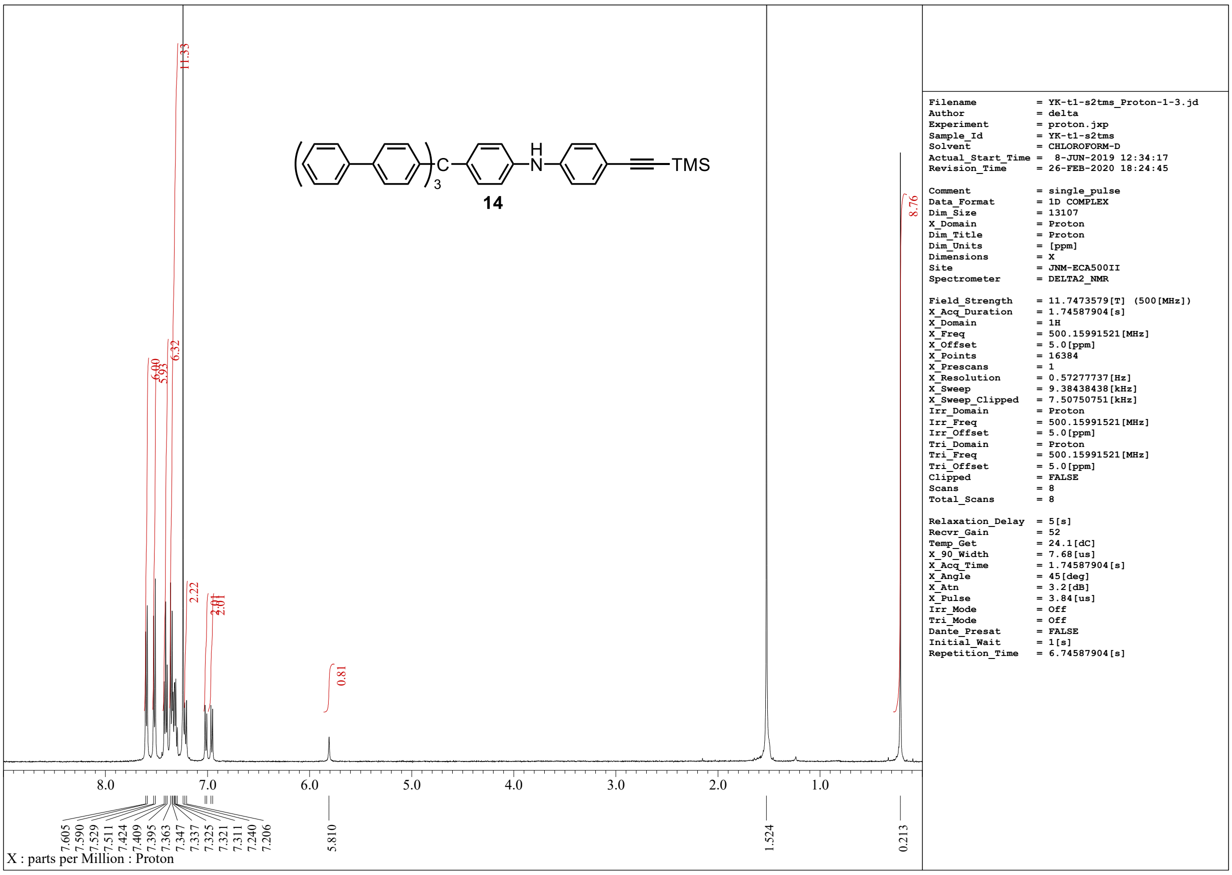

^{13}C{^{1}H} NMR Spectrum of **3a** ($CDCl_3$, 76 MHz).

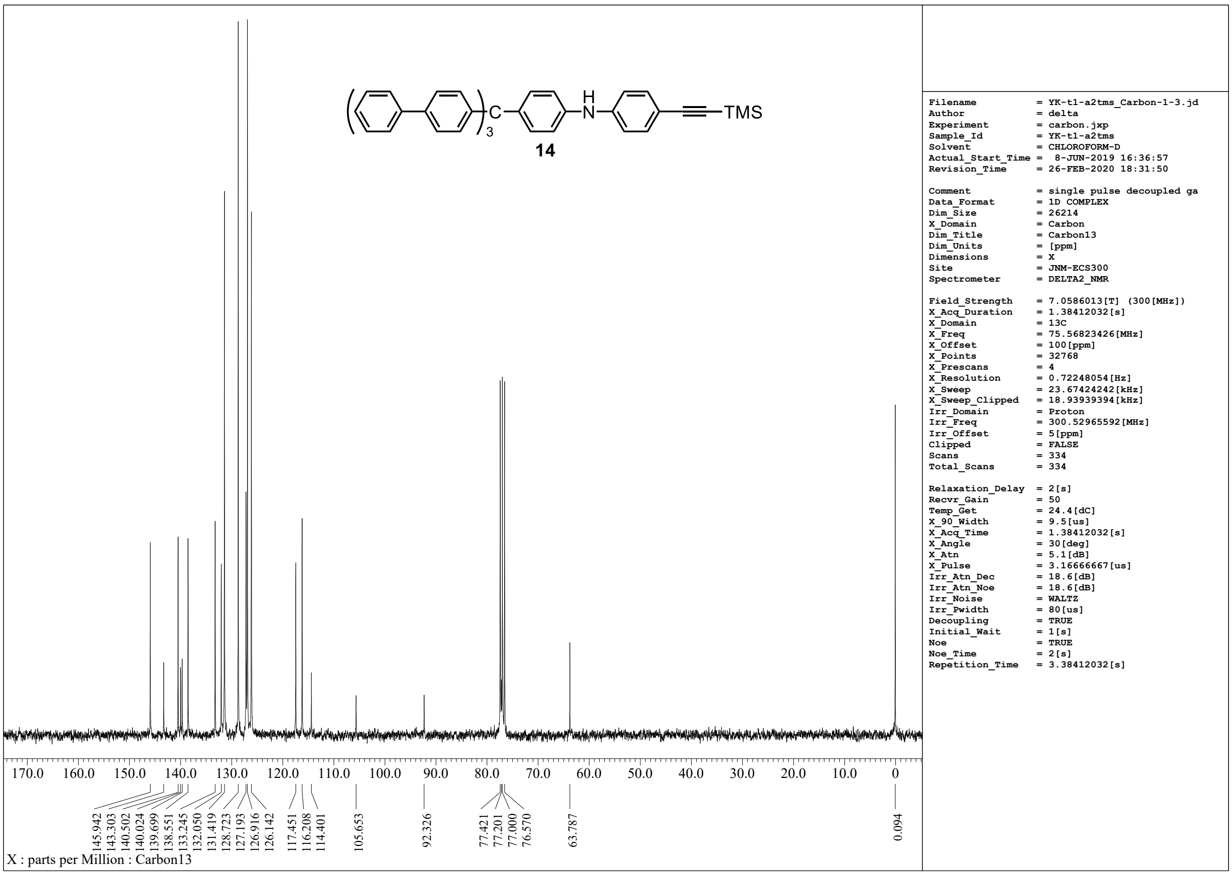

^{1}H NMR Spectrum of **3d** ($CDCl_3$, 500 MHz).

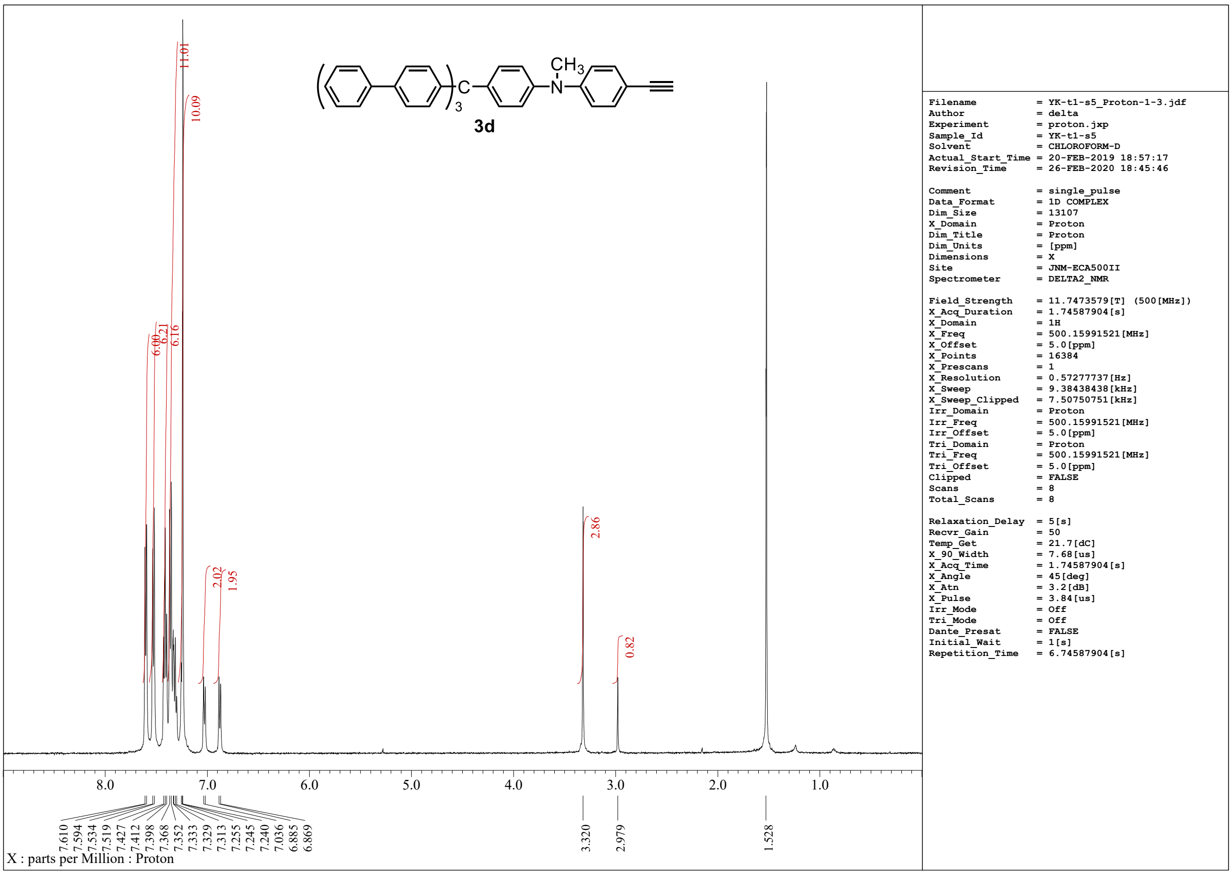

^{13}C{^{1}H} NMR Spectrum of **3d** ($CDCl_3$, 126 MHz).

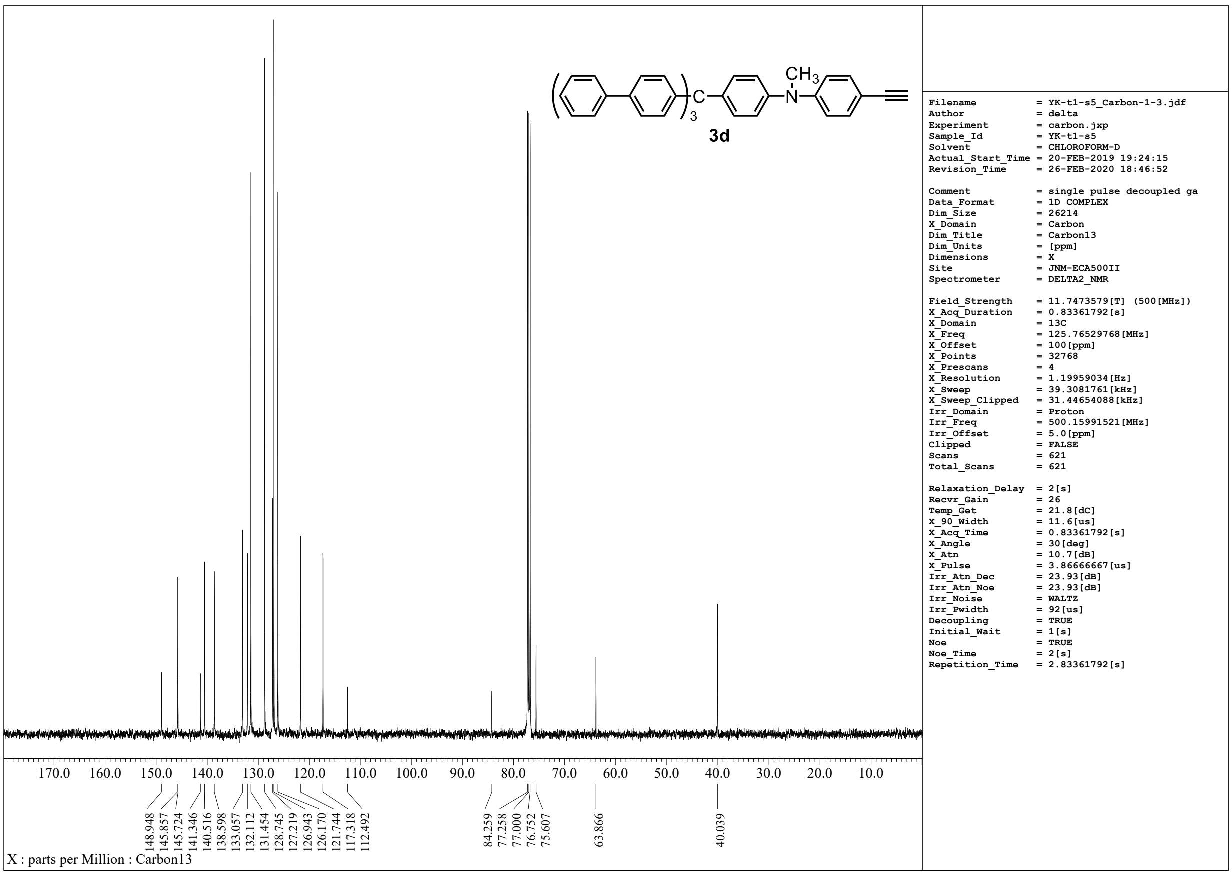

^{1}H NMR Spectrum of **3f** ($CDCl_3$, 500 MHz).

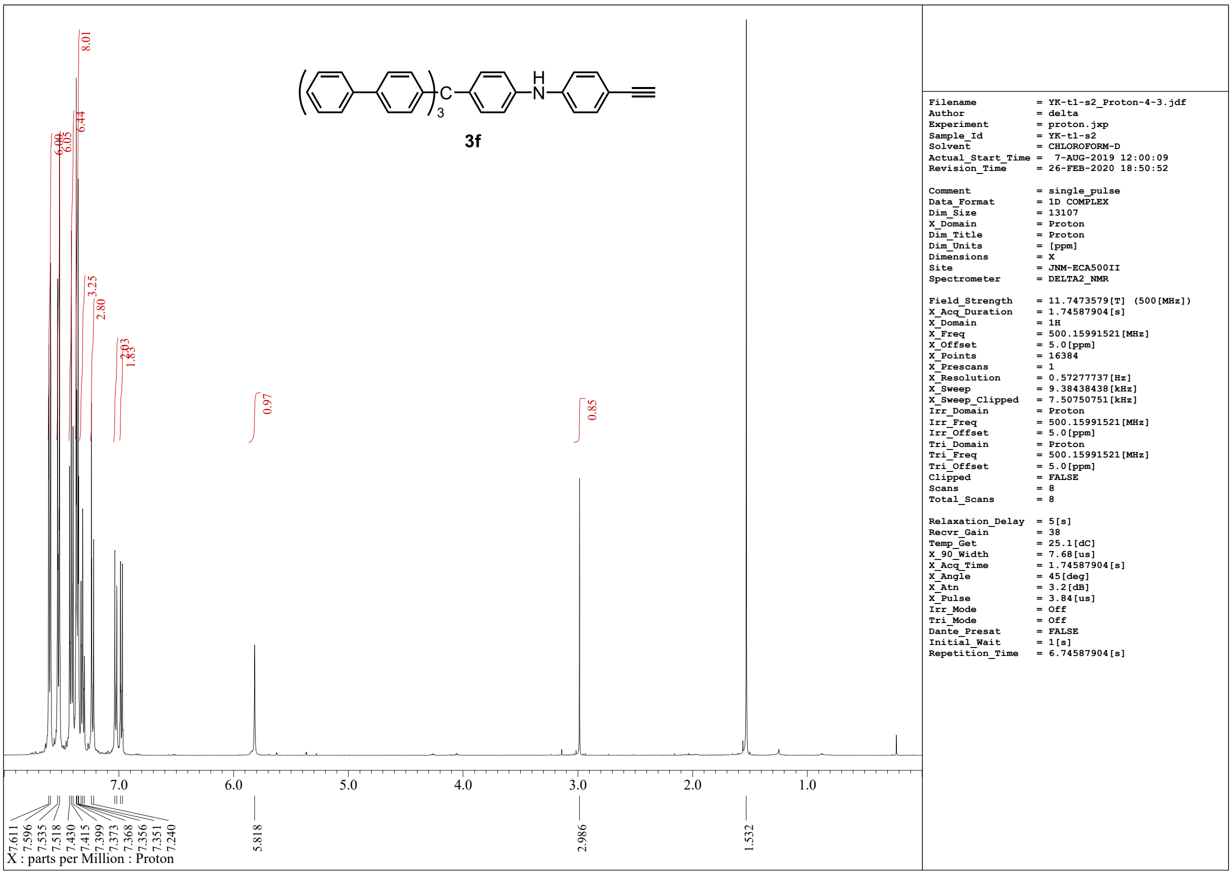

$^{13}C\{^{1}H\}$ NMR Spectrum of **3f** ($CDCl_3$, 126 MHz).

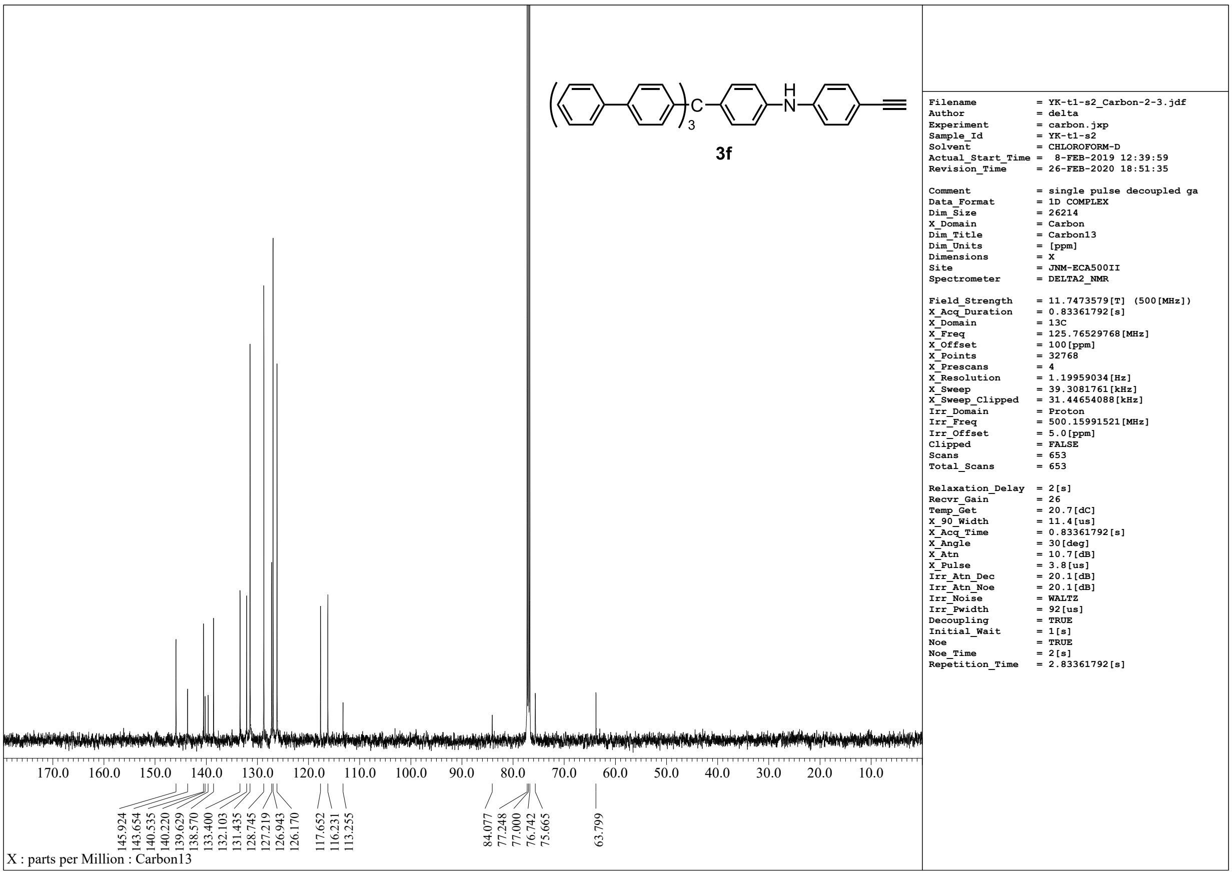

^{1}H NMR Spectrum of **3h** ($CDCl_3$, 500 MHz).

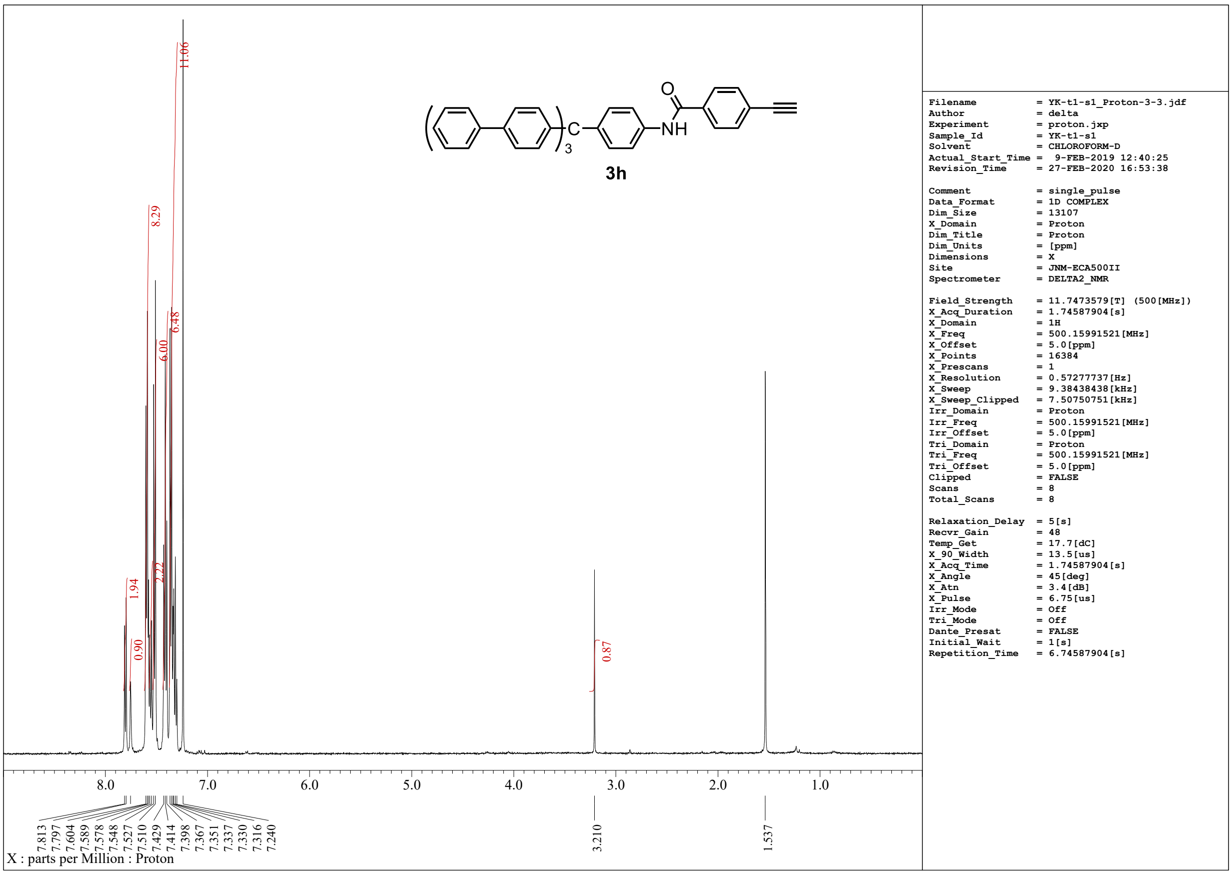

^{13}C{^{1}H} NMR Spectrum of **3h** ($CDCl_3$, 126 MHz).

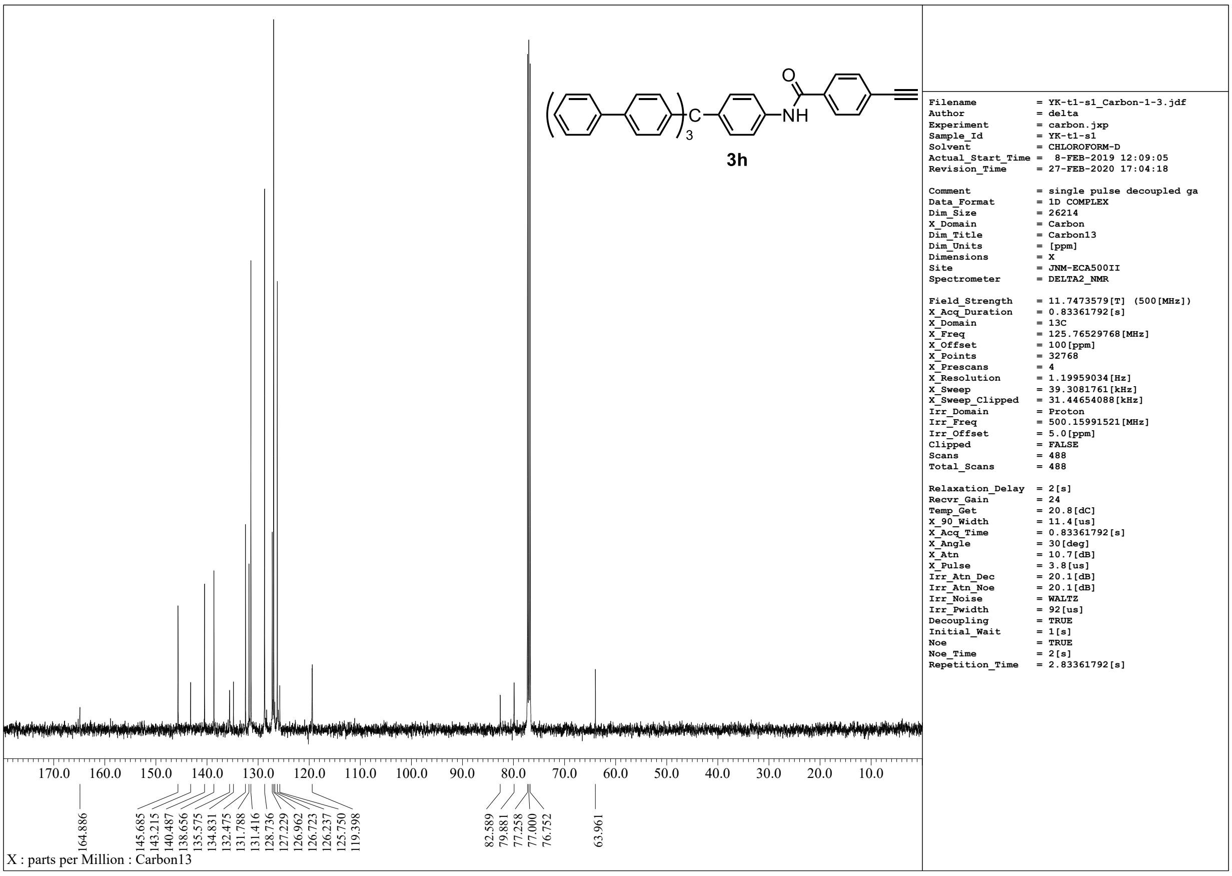

^{1}H NMR Spectrum of **3g** ($CDCl_3$, 500 MHz).

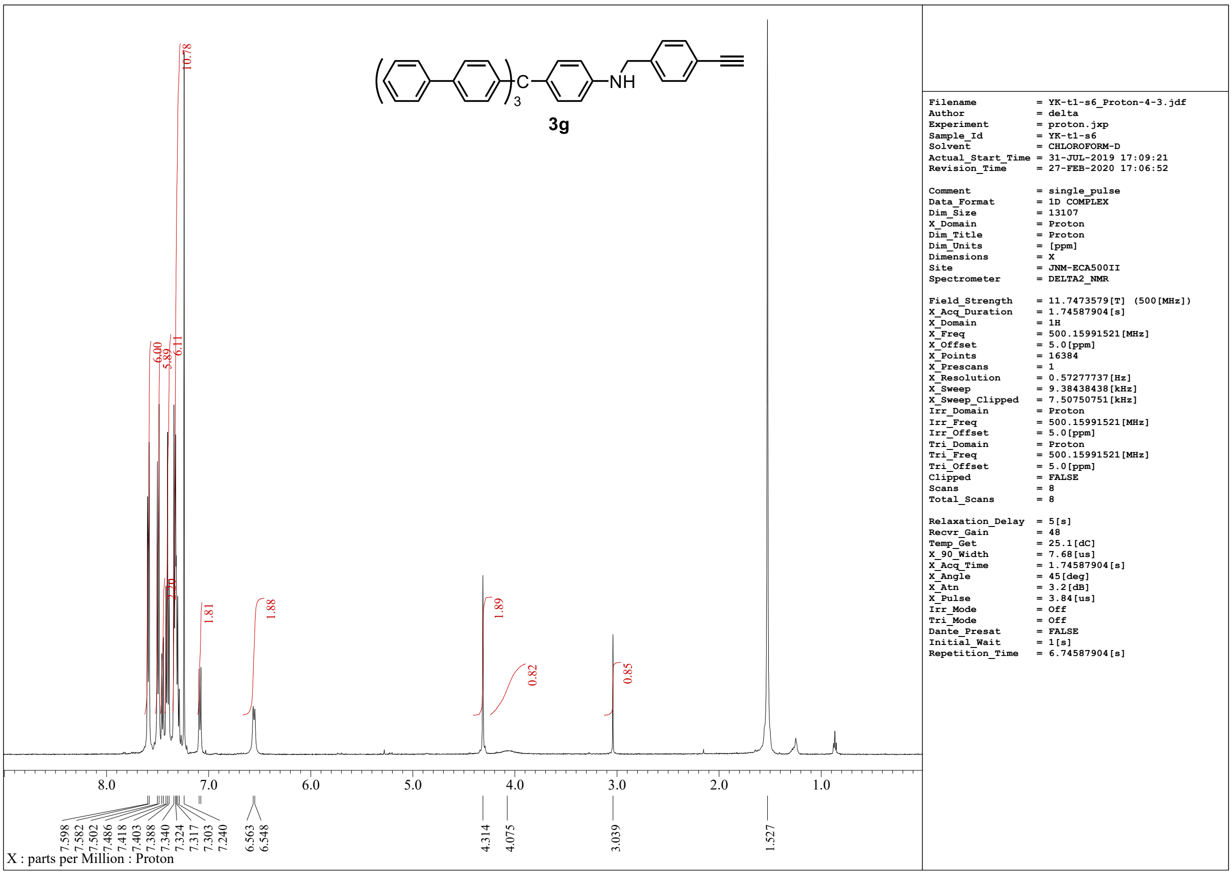

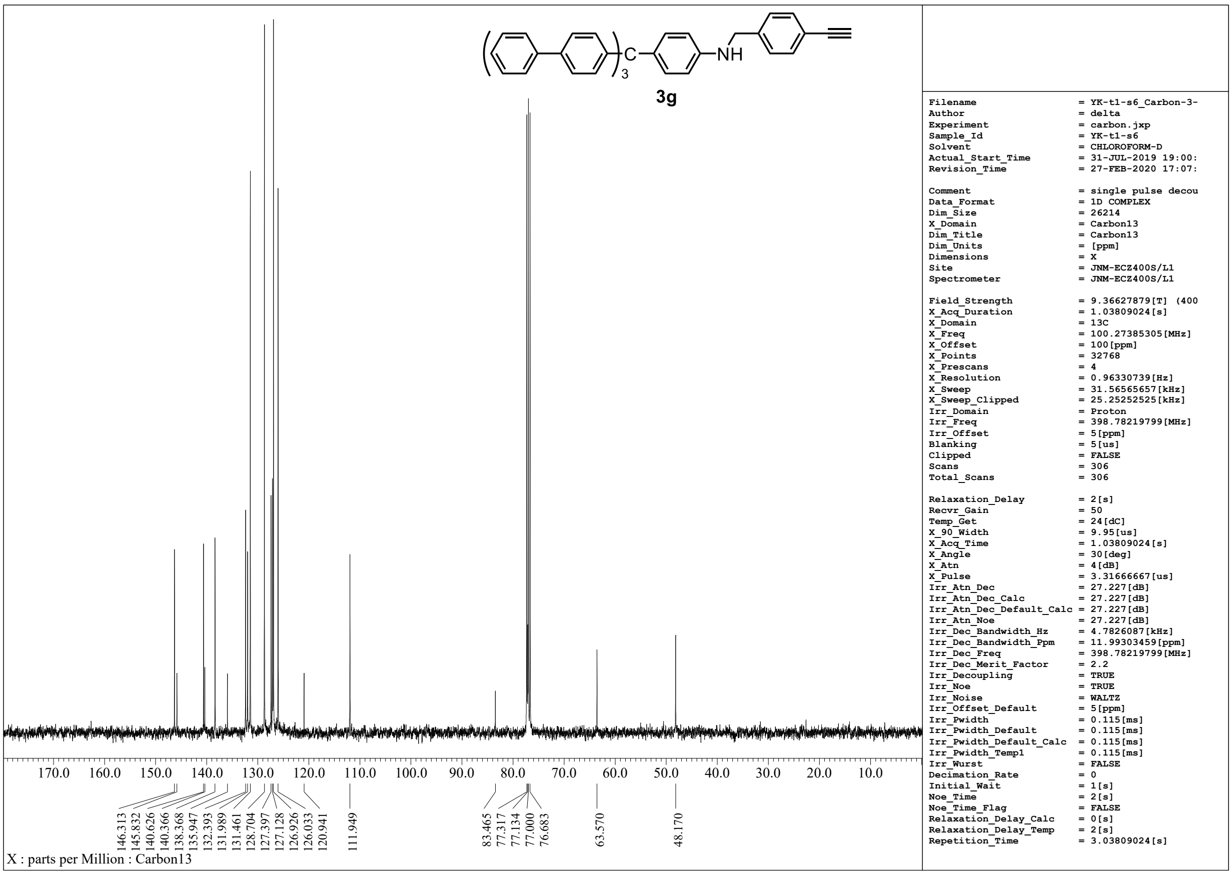

^{1}H NMR Spectrum of **3e** ($CDCl_3$, 500 MHz).

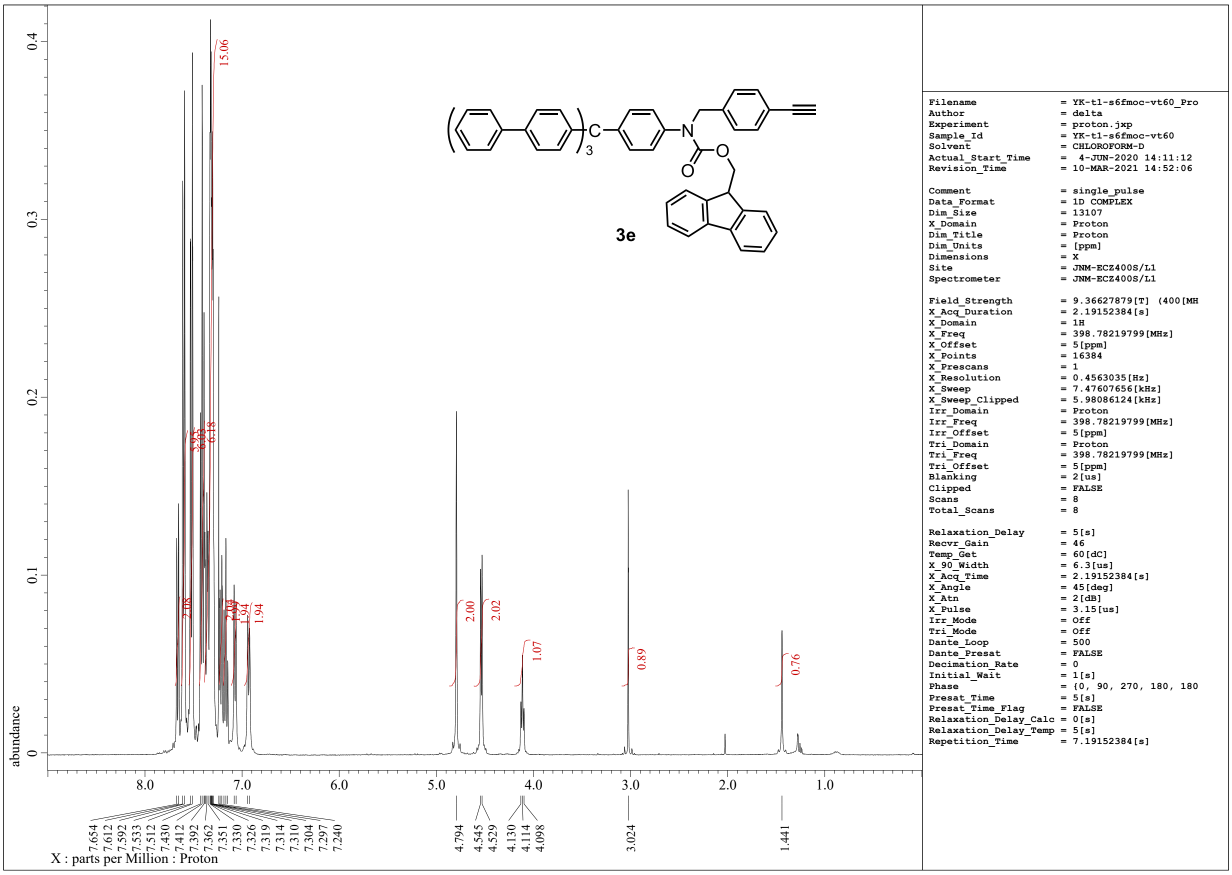

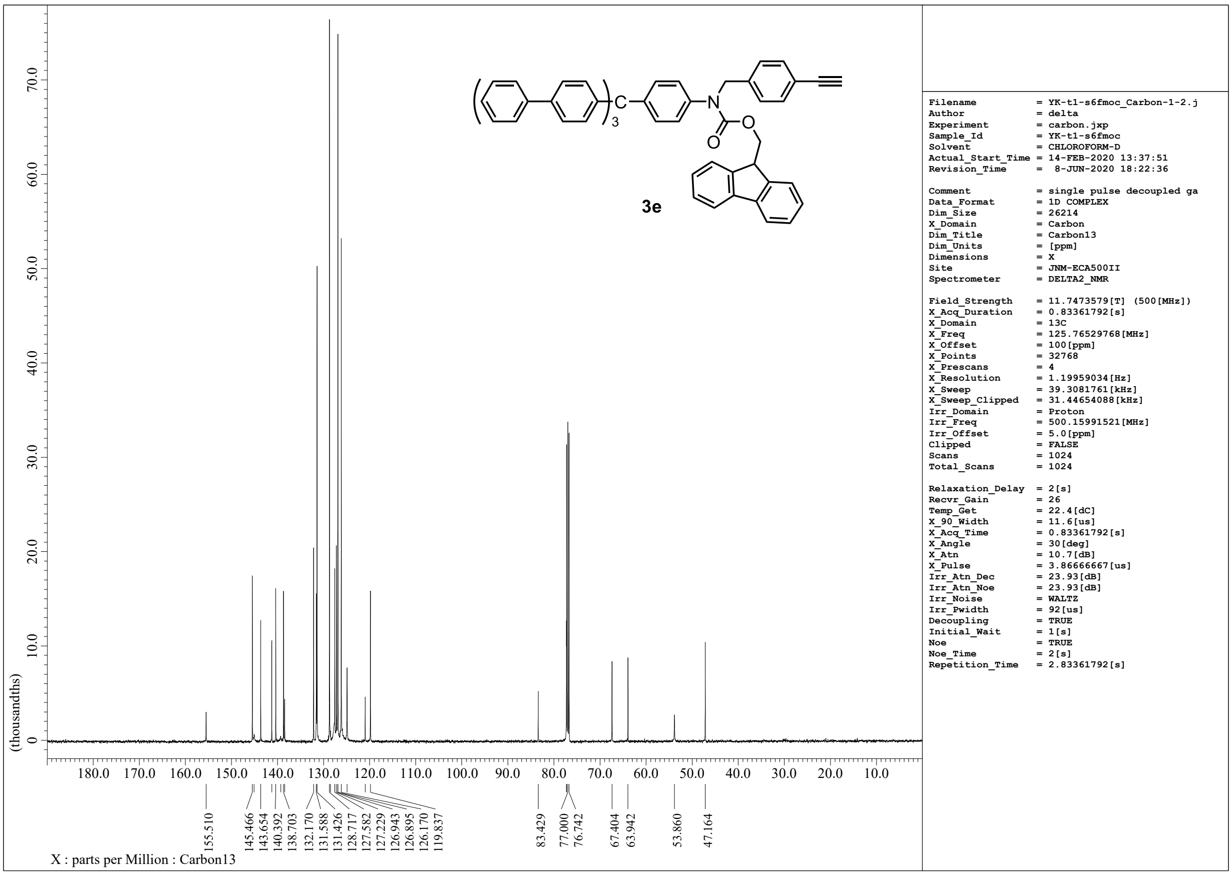

^{1}H NMR Spectrum of **16** ($CDCl_3$, 500 MHz).

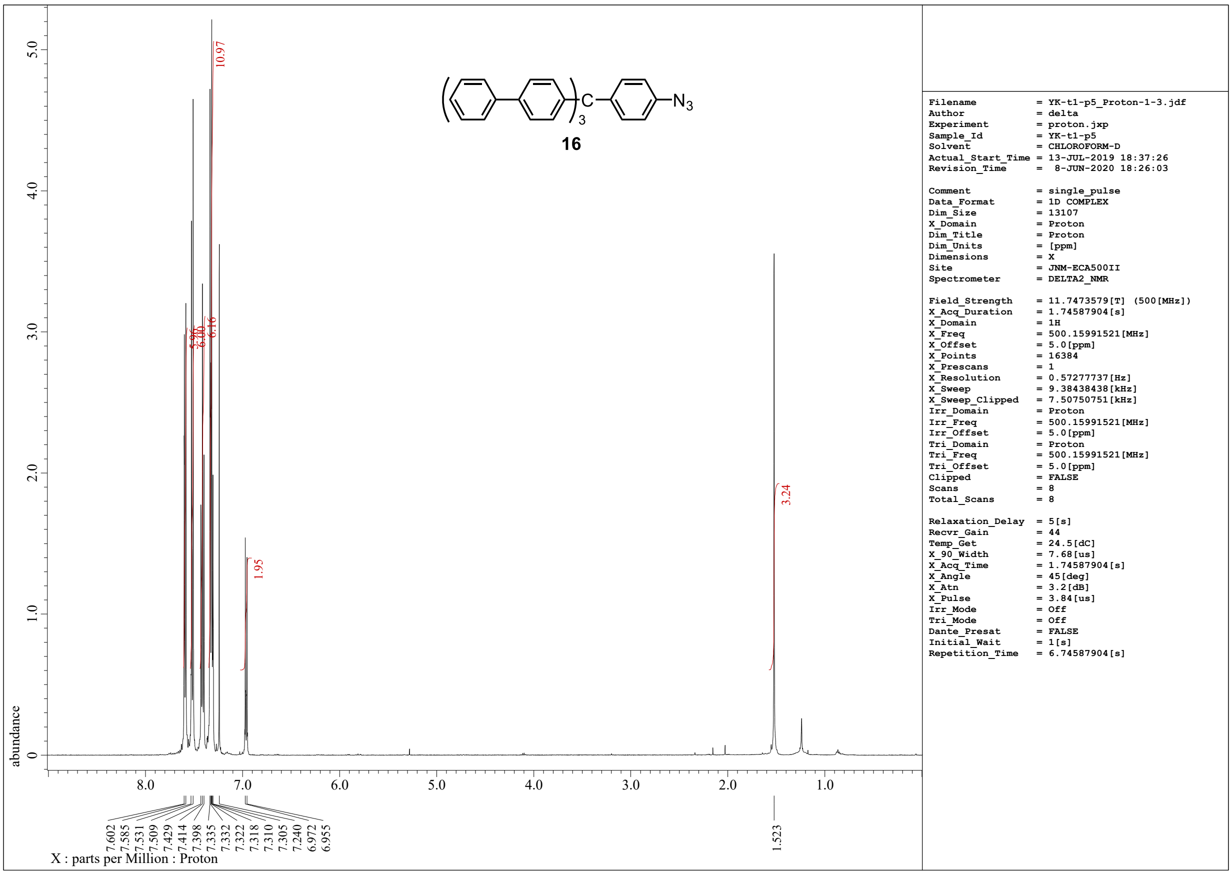

^{13}C{^{1}H} NMR Spectrum of **16** ($CDCl_3$, 76 MHz).

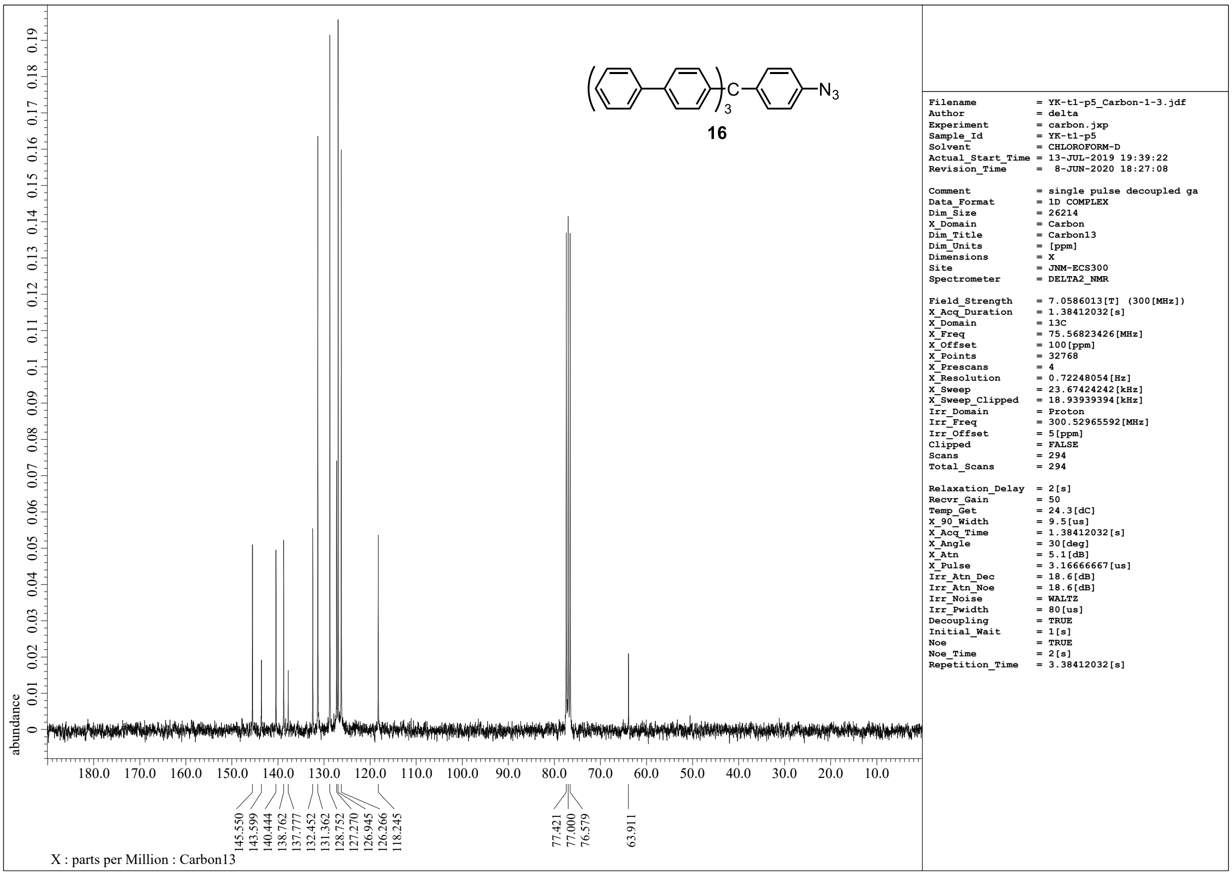

^{1}H NMR Spectrum of **3i** ($CDCl_3$, 500 MHz).

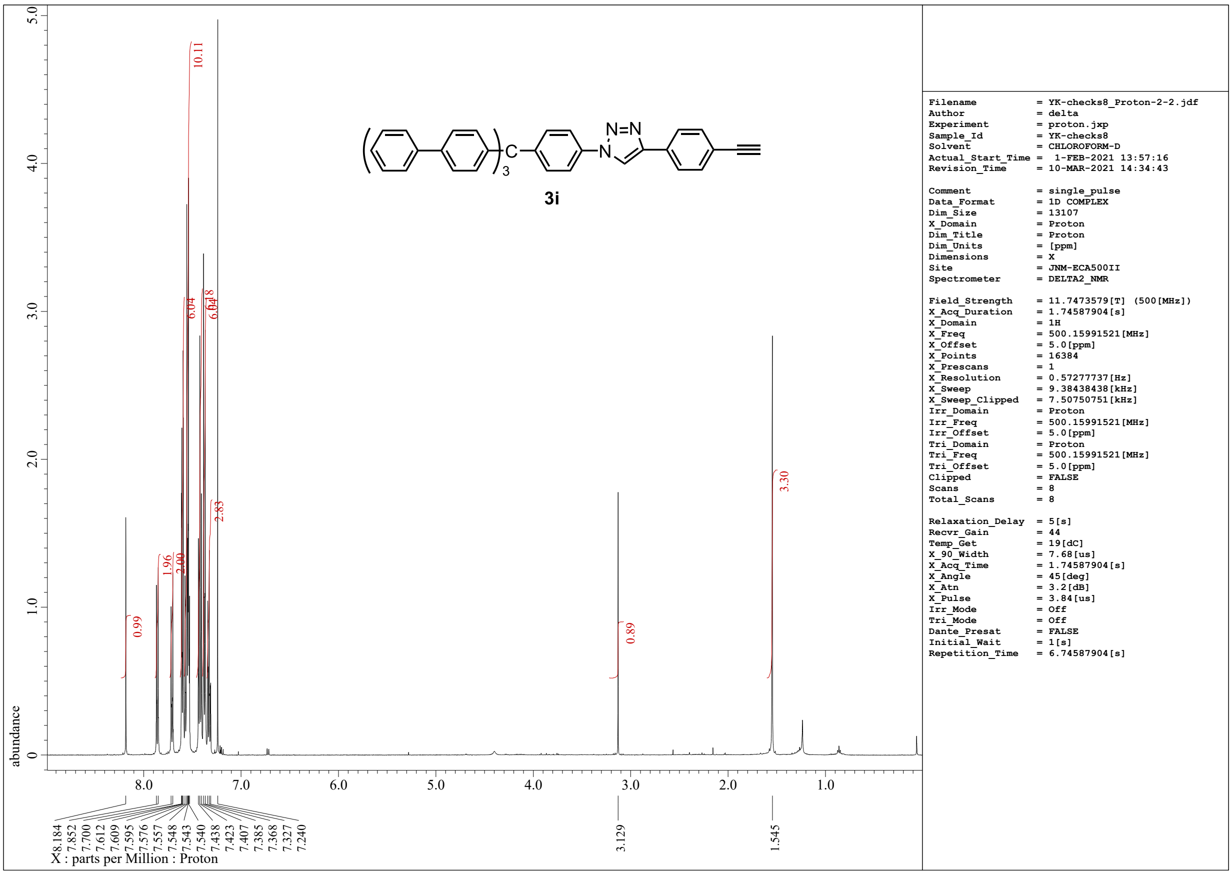

^{13}C{^{1}H} NMR Spectrum of **3i** ($CDCl_3$, 126 MHz).

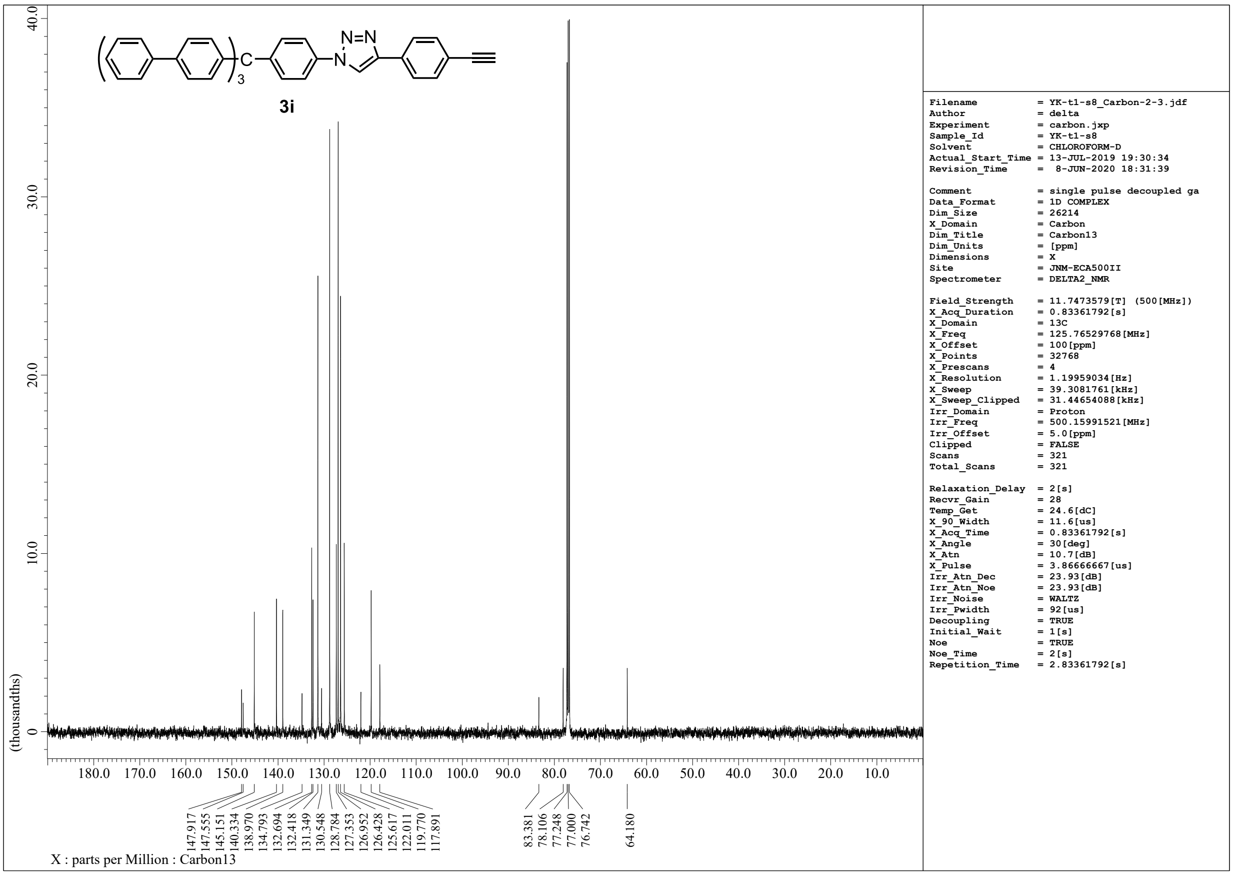

^{1}H NMR Spectrum of **4a** ($CDCl_3$, 500 MHz).

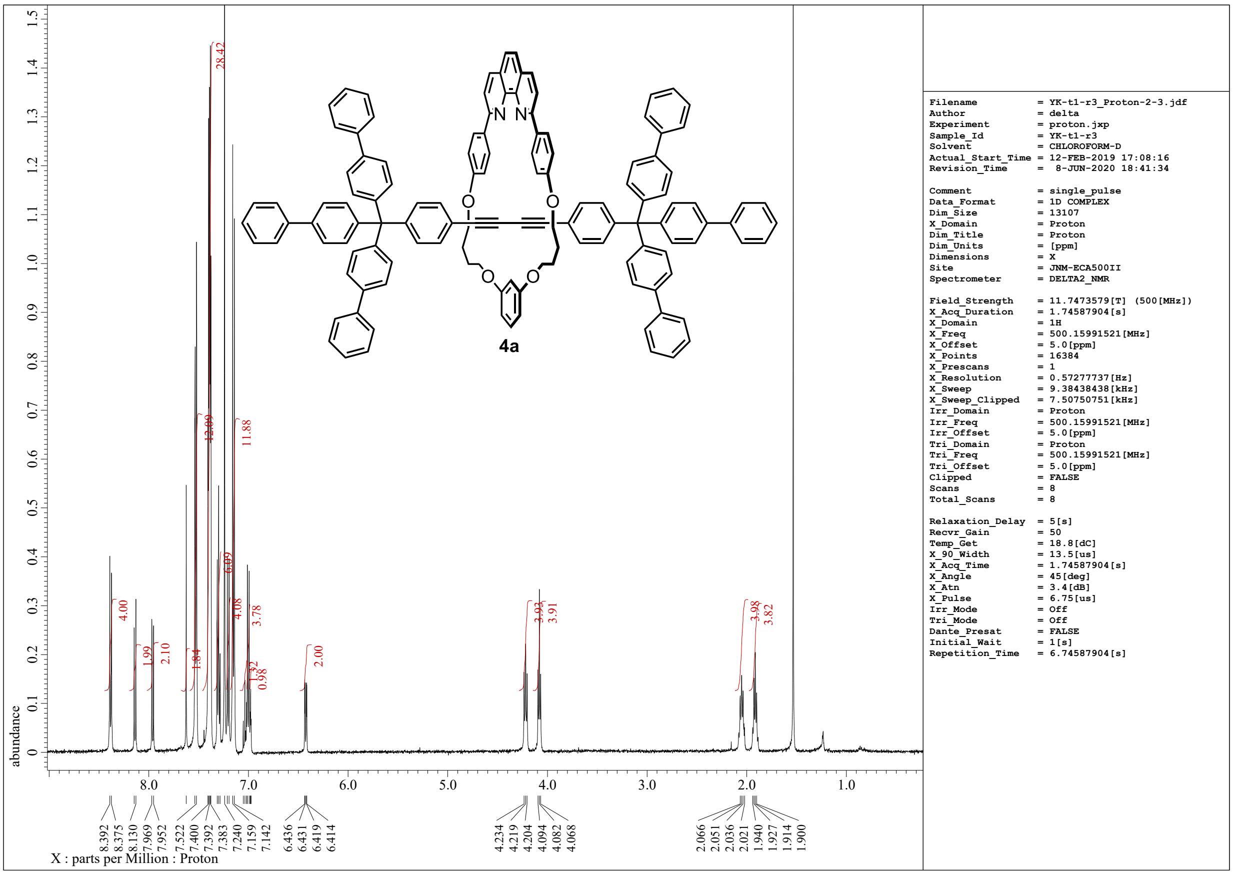

$^{13}C\{^{1}H\}$ NMR Spectrum of **4a** ($CDCl_3$, 126 MHz).

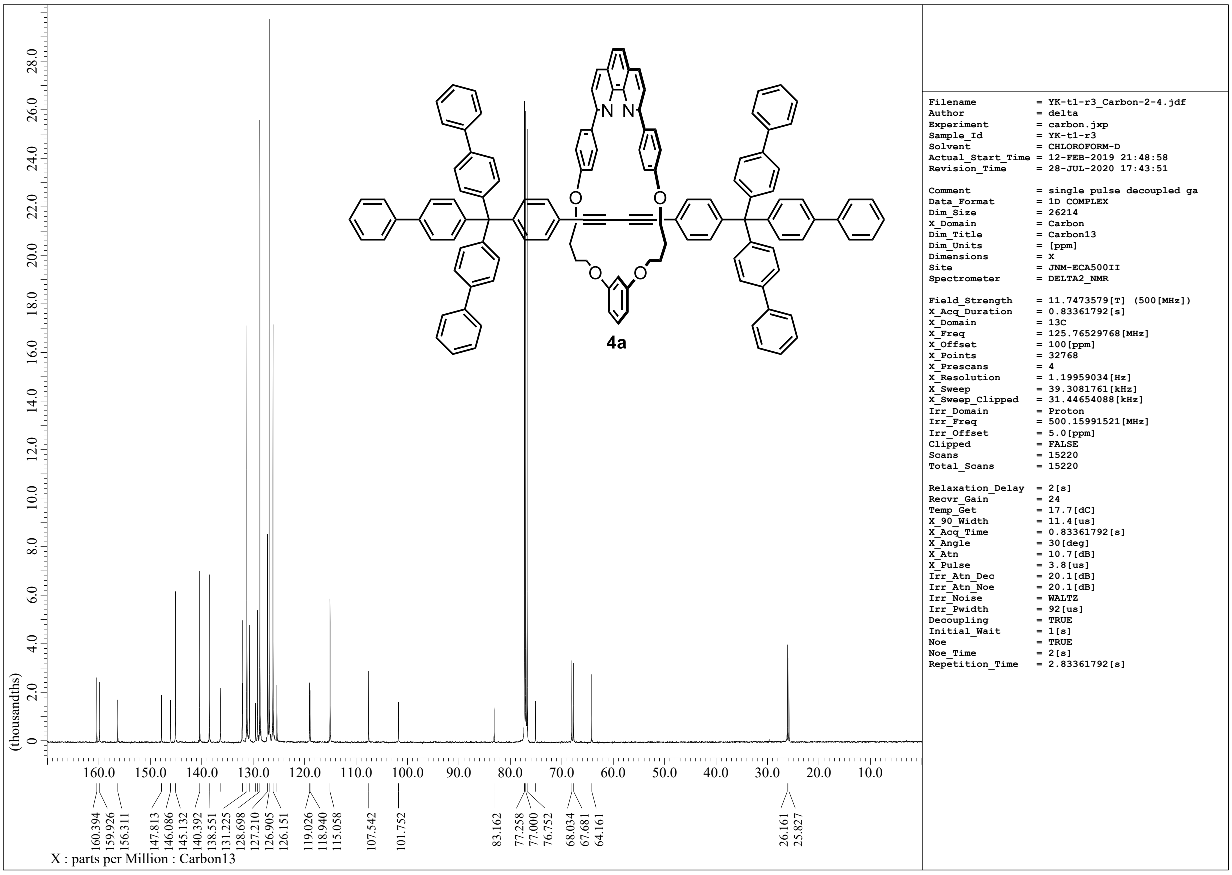

^{1}H NMR Spectrum of **17a** ($CDCl_3$, 500 MHz).

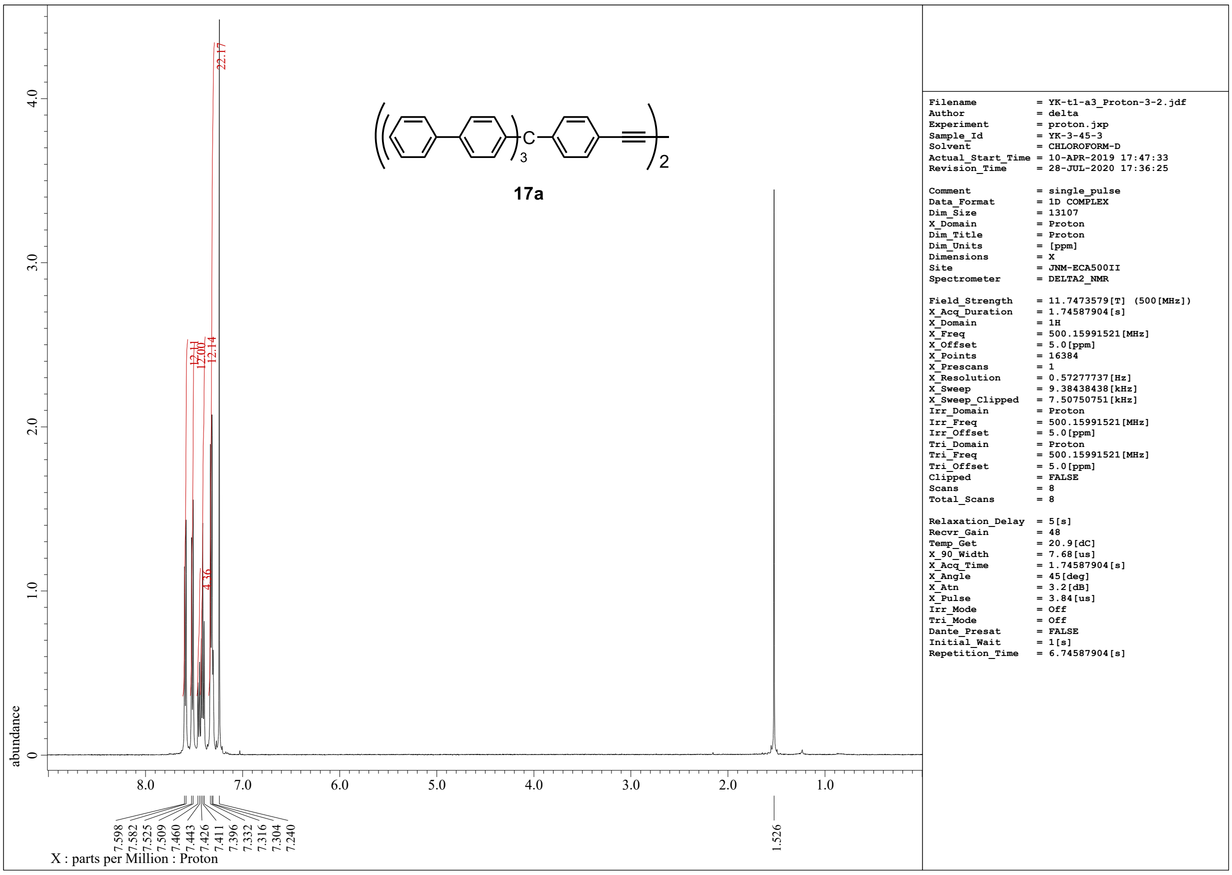

^{13}C{^{1}H} NMR Spectrum of **17a** ($CDCl_3$, 126 MHz).

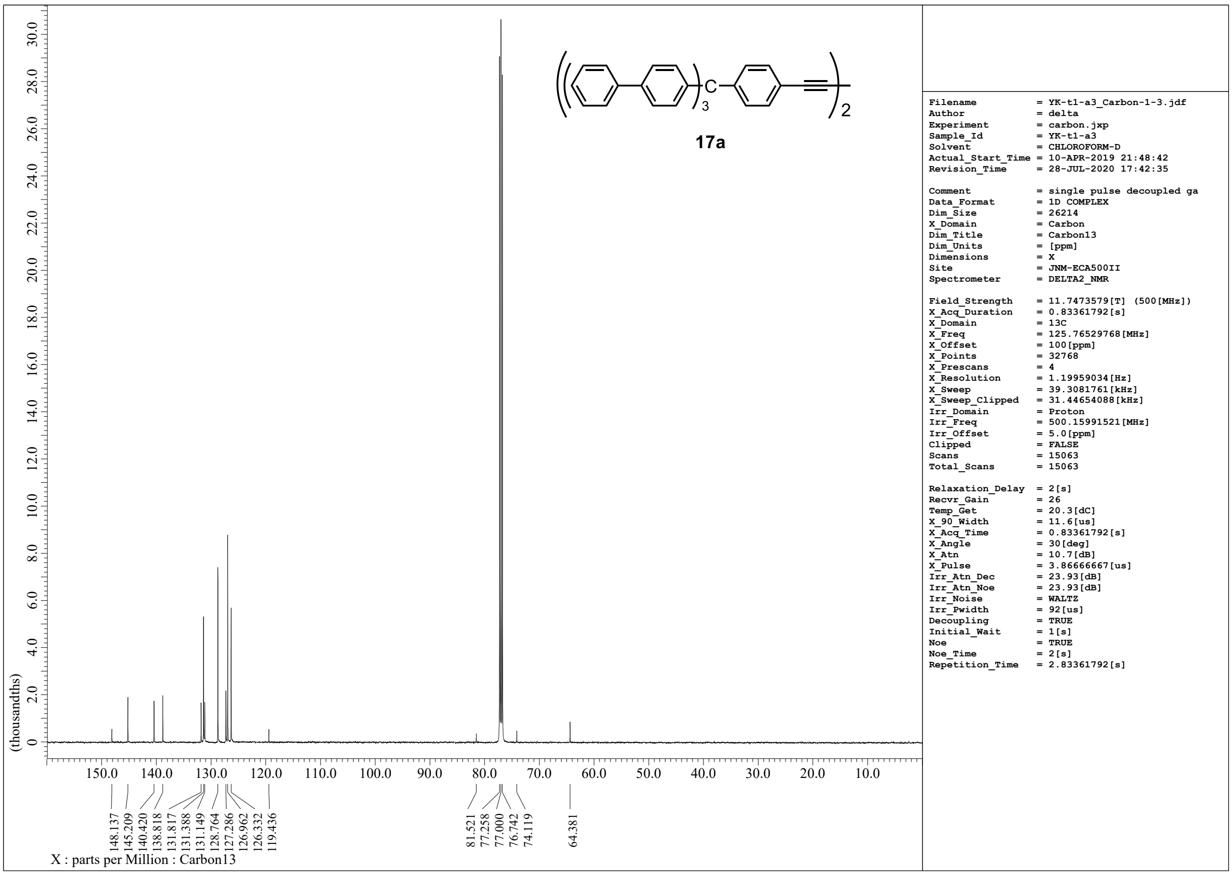

^{1}H NMR Spectrum of **4b** ($CDCl_3$, 500 MHz).

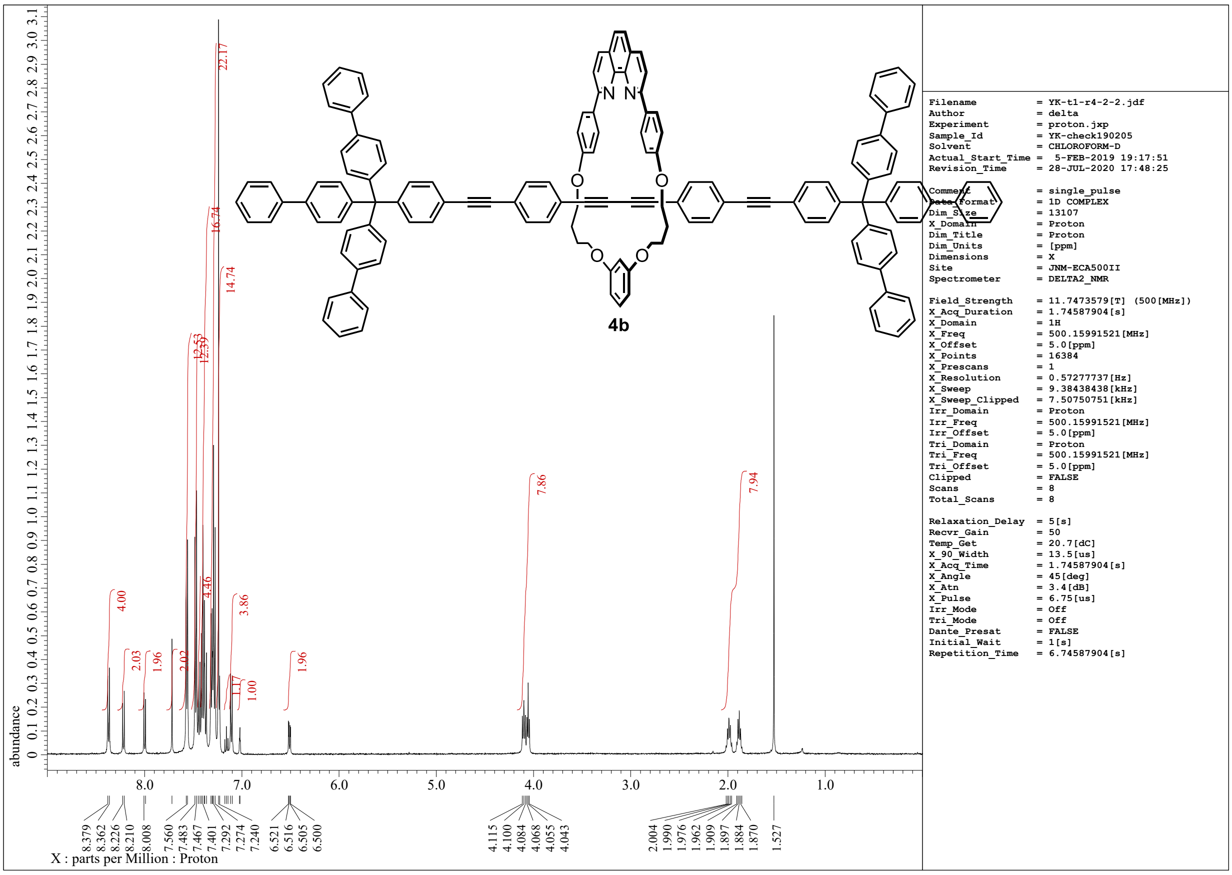

^{13}C{^{1}H} NMR Spectrum of **4b** ($CDCl_3$, 126 MHz).

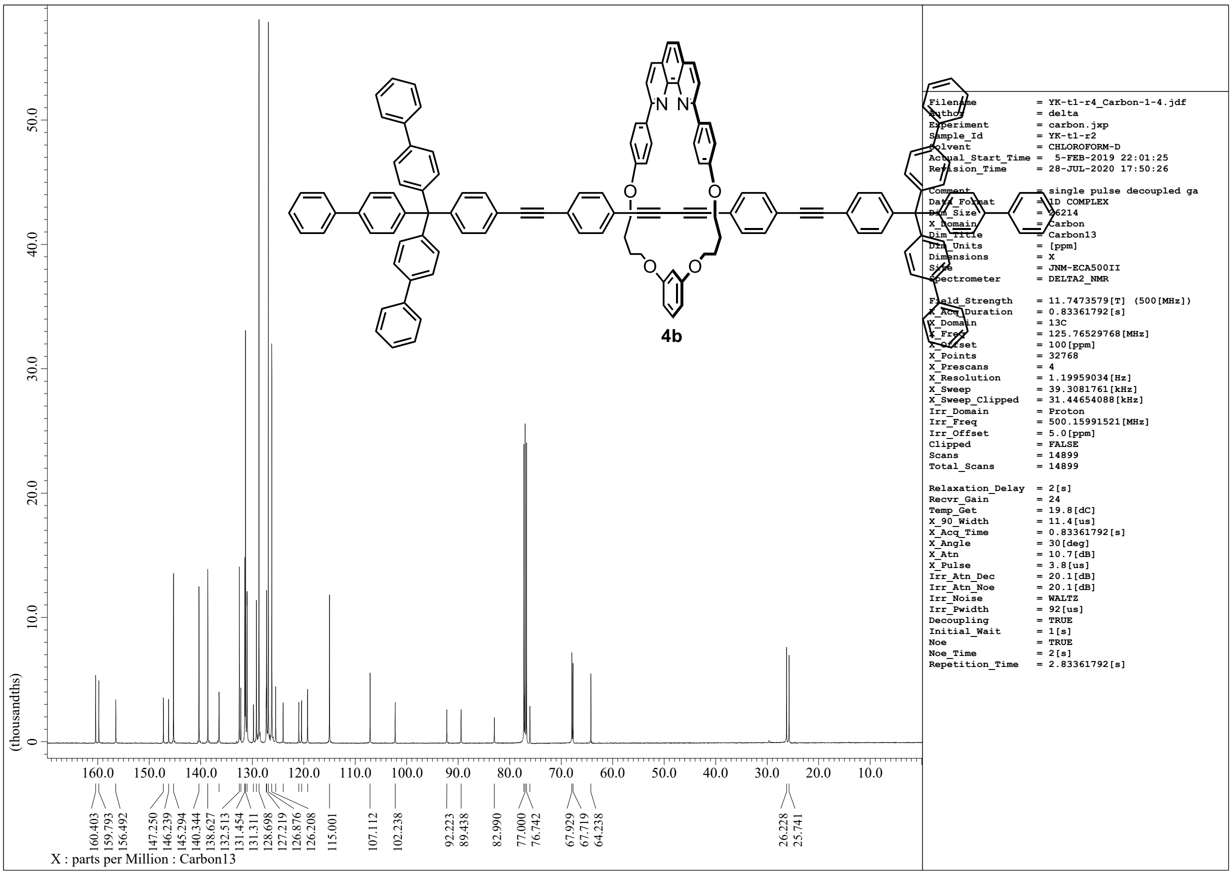

^{1}H NMR Spectrum of **4c** ($CDCl_3$, 500 MHz).

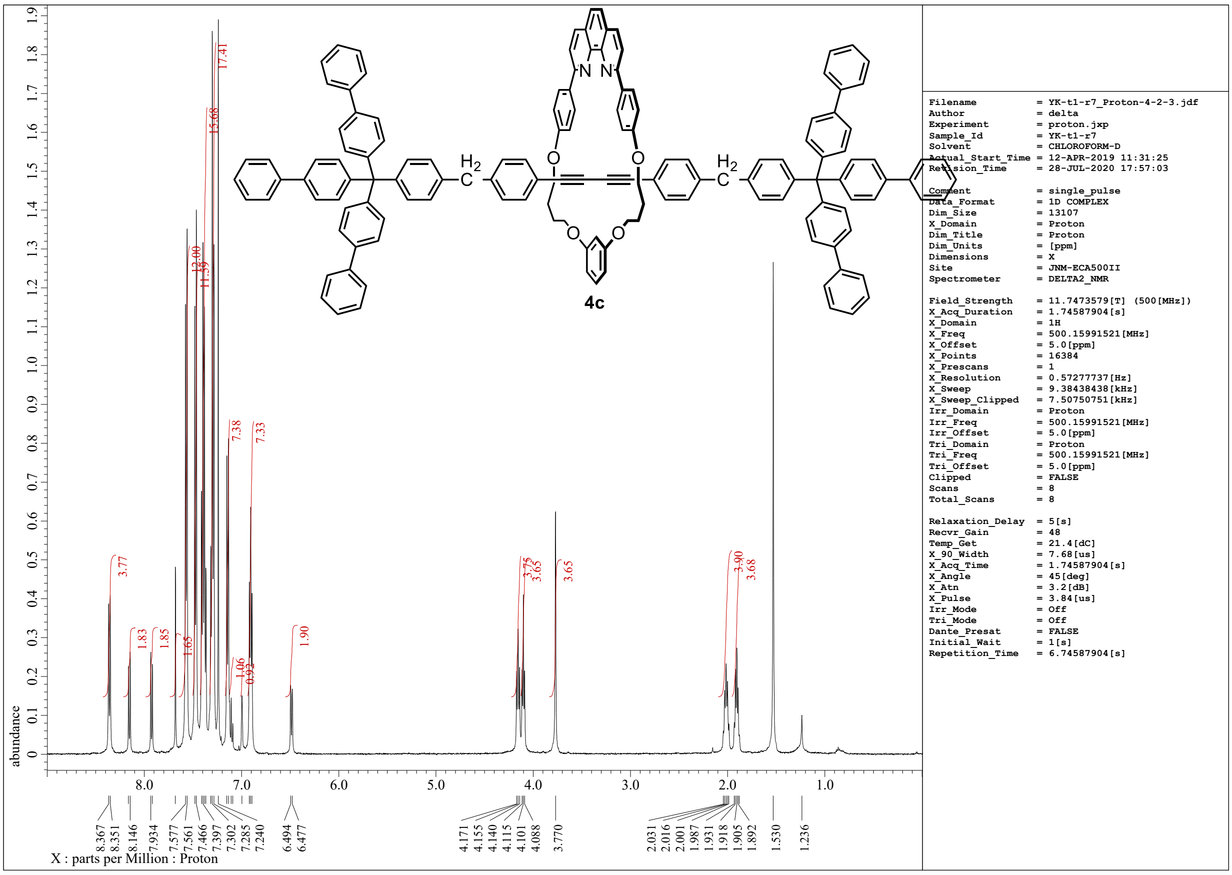

$^{13}C\{^{1}H\}$ NMR Spectrum of **4c** ($CDCl_3$, 126 MHz).

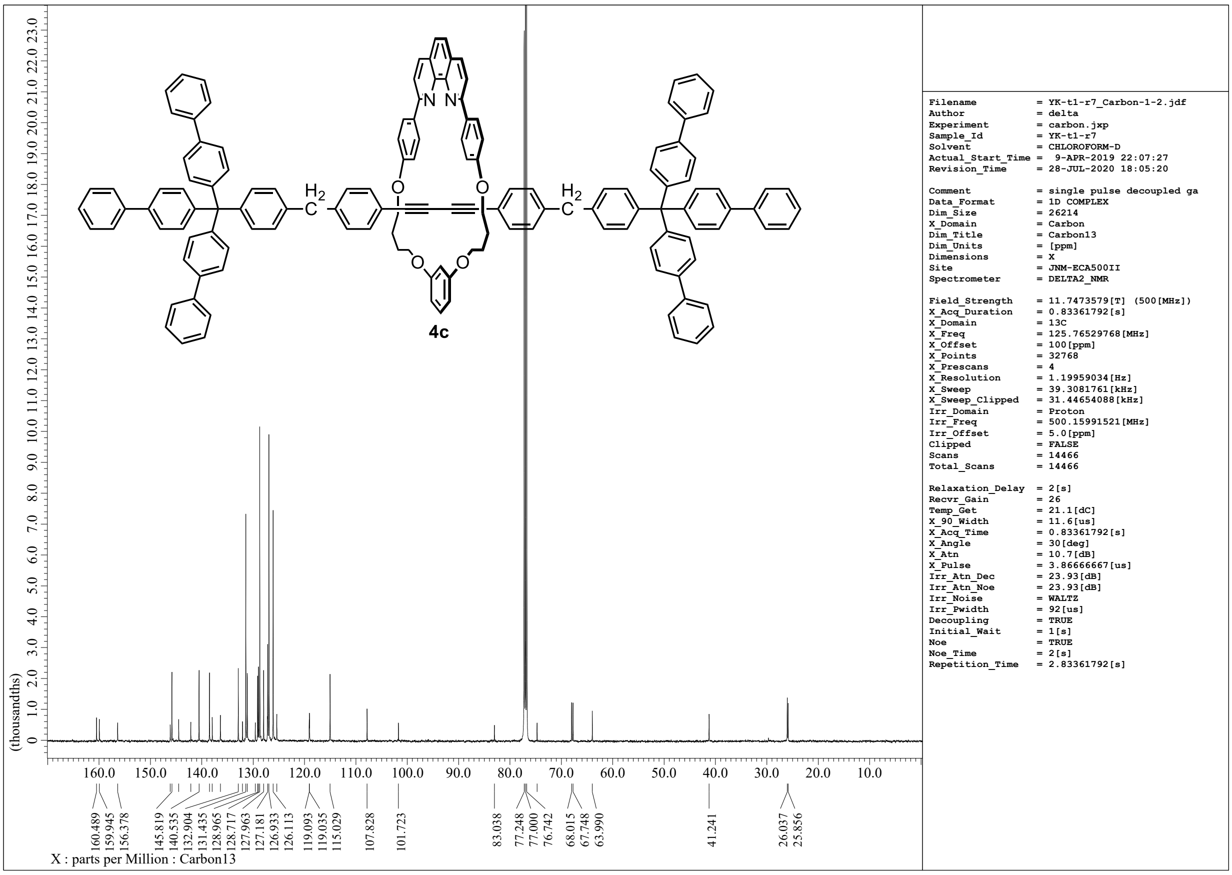

^{1}H NMR Spectrum of **17c** ($CDCl_3$, 500 MHz).

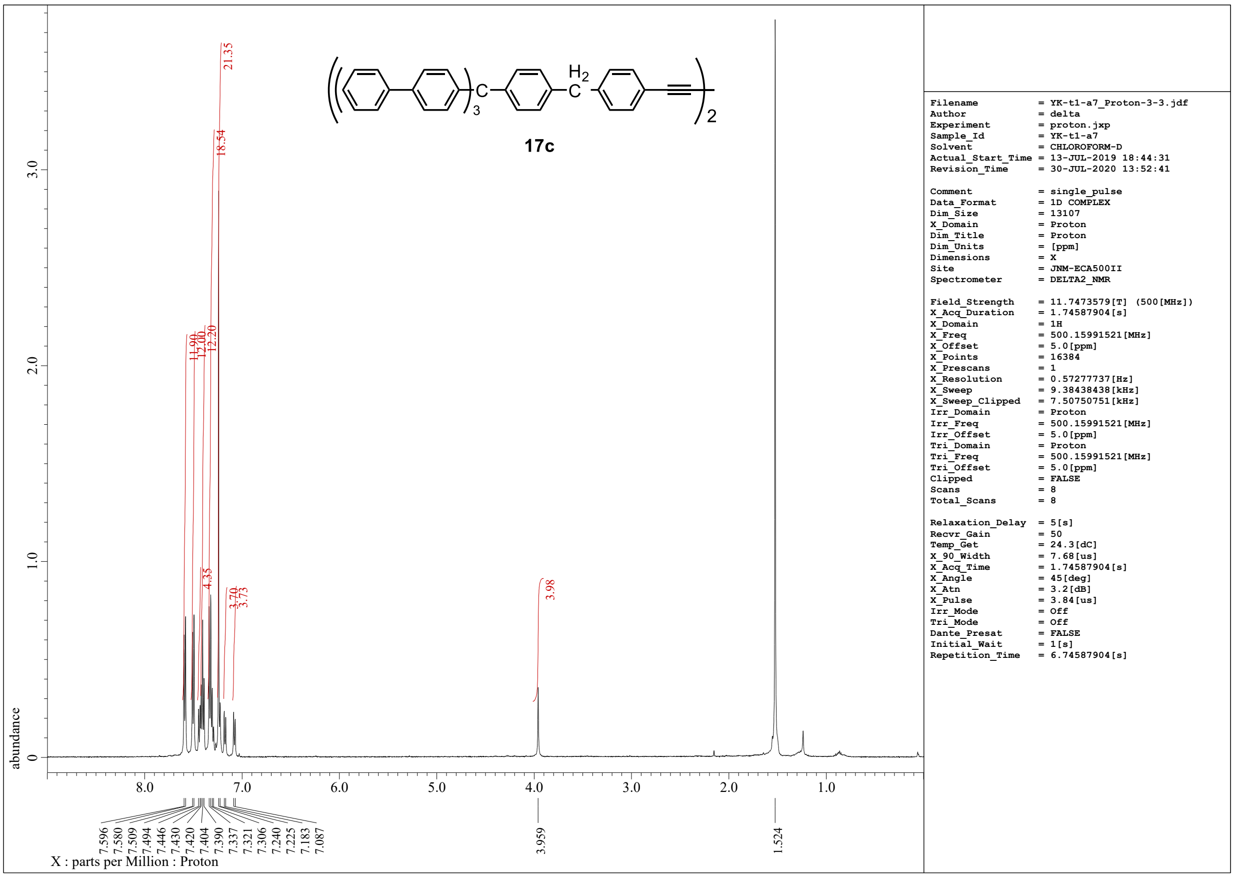

$^{13}C\{^{1}H\}$ NMR Spectrum of **17c** ($CDCl_3$, 126 MHz).

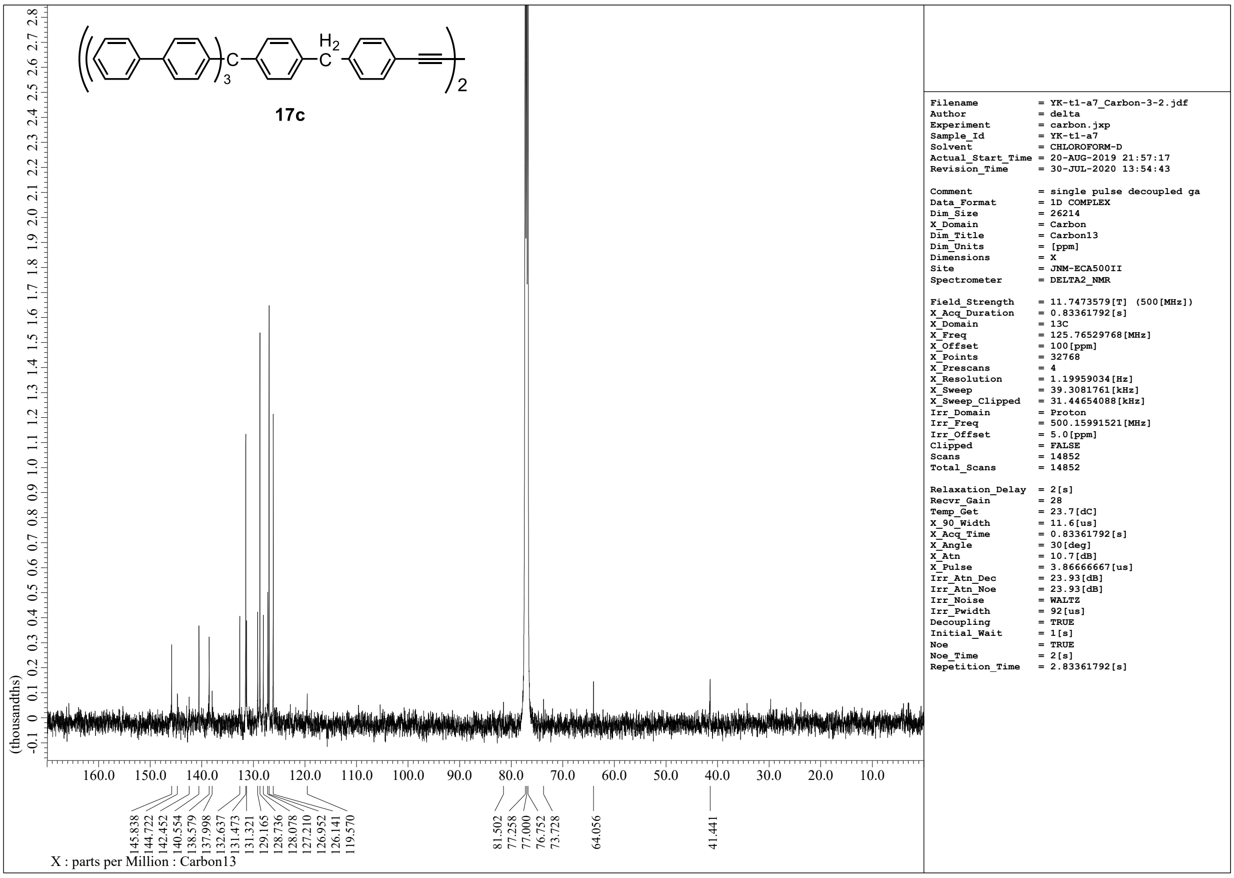

^{1}H NMR Spectrum of **4d** ($CDCl_3$, 500 MHz).

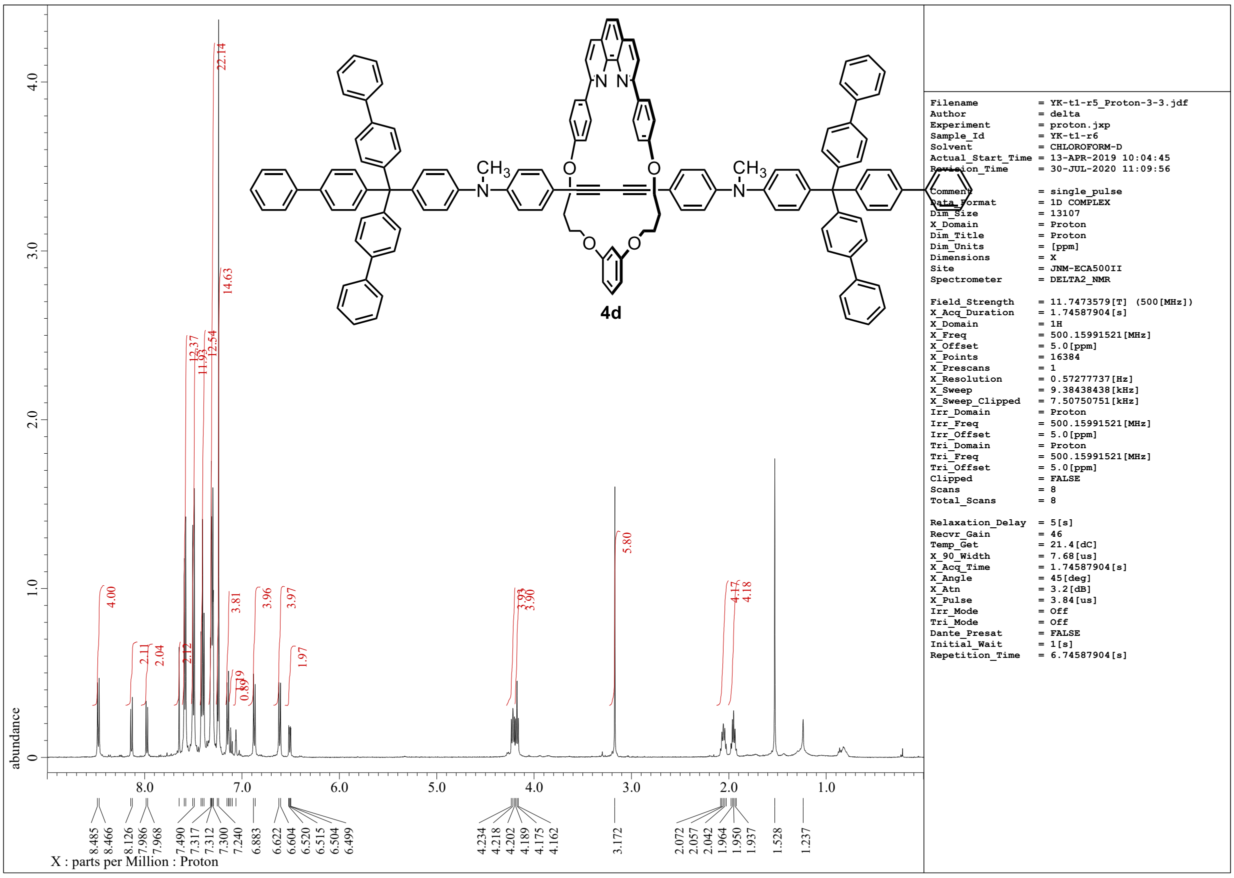

^{13}C{^{1}H} NMR Spectrum of **4d** ($CDCl_3$, 126 MHz).

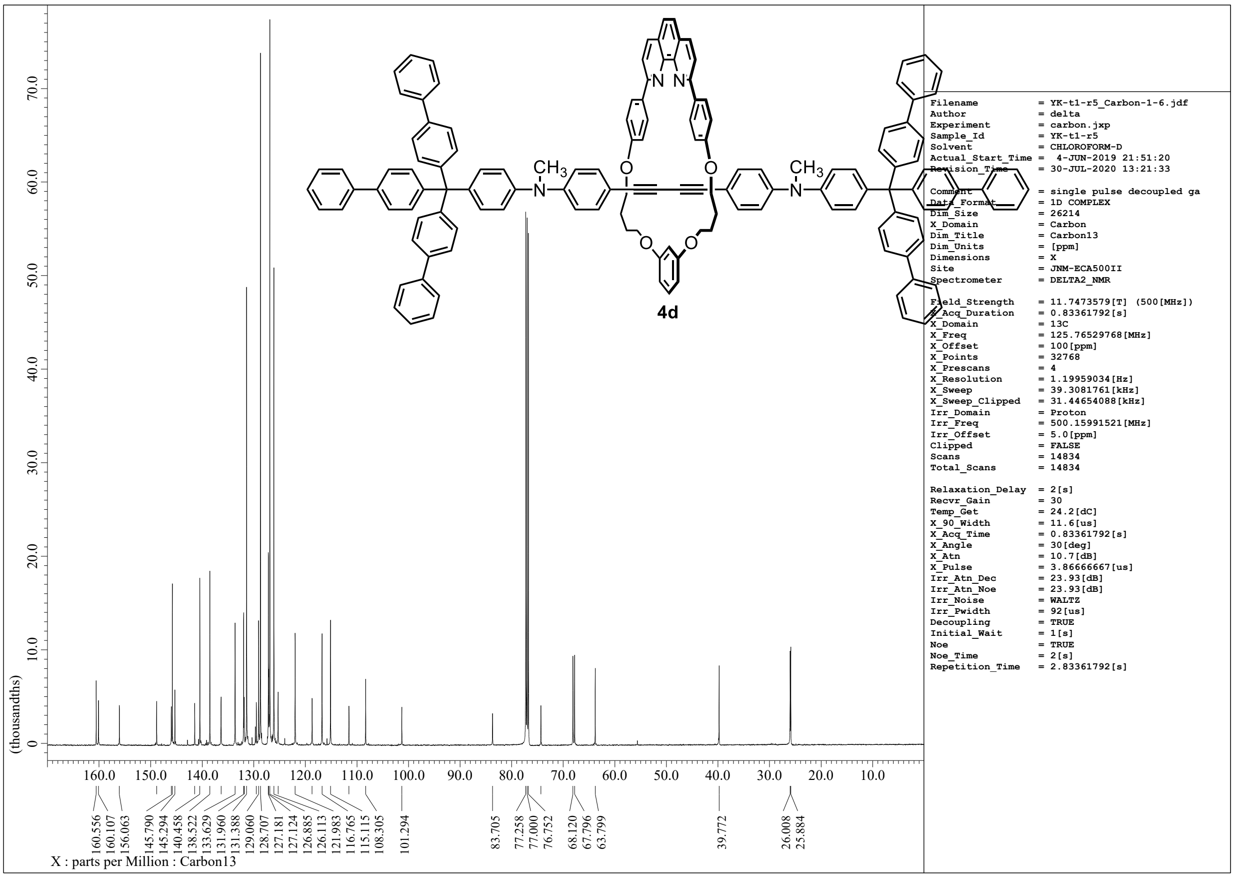

^{1}H NMR Spectrum of **17d** ($CDCl_3$, 500 MHz).

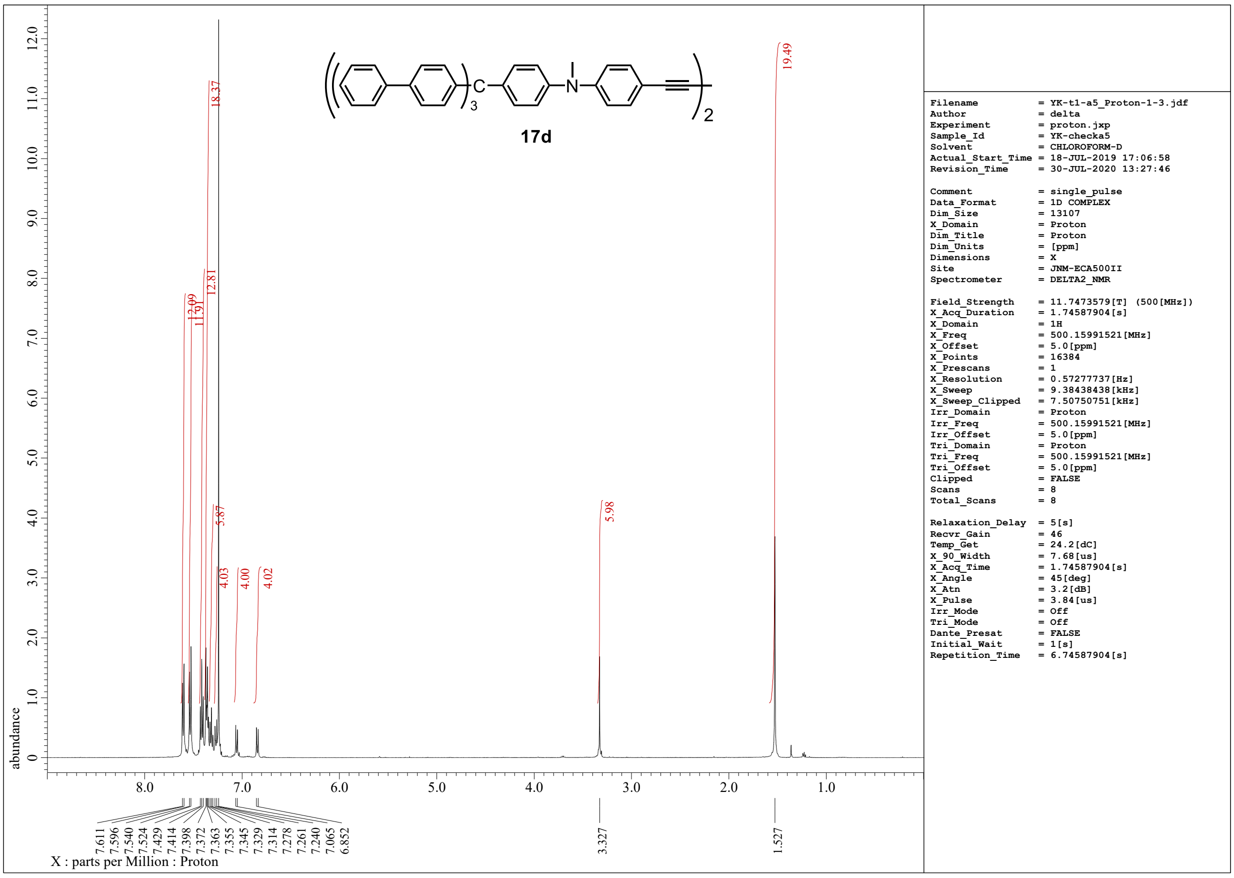

$^{13}C\{^{1}H\}$ NMR Spectrum of **17d** ($CDCl_3$, 126 MHz).

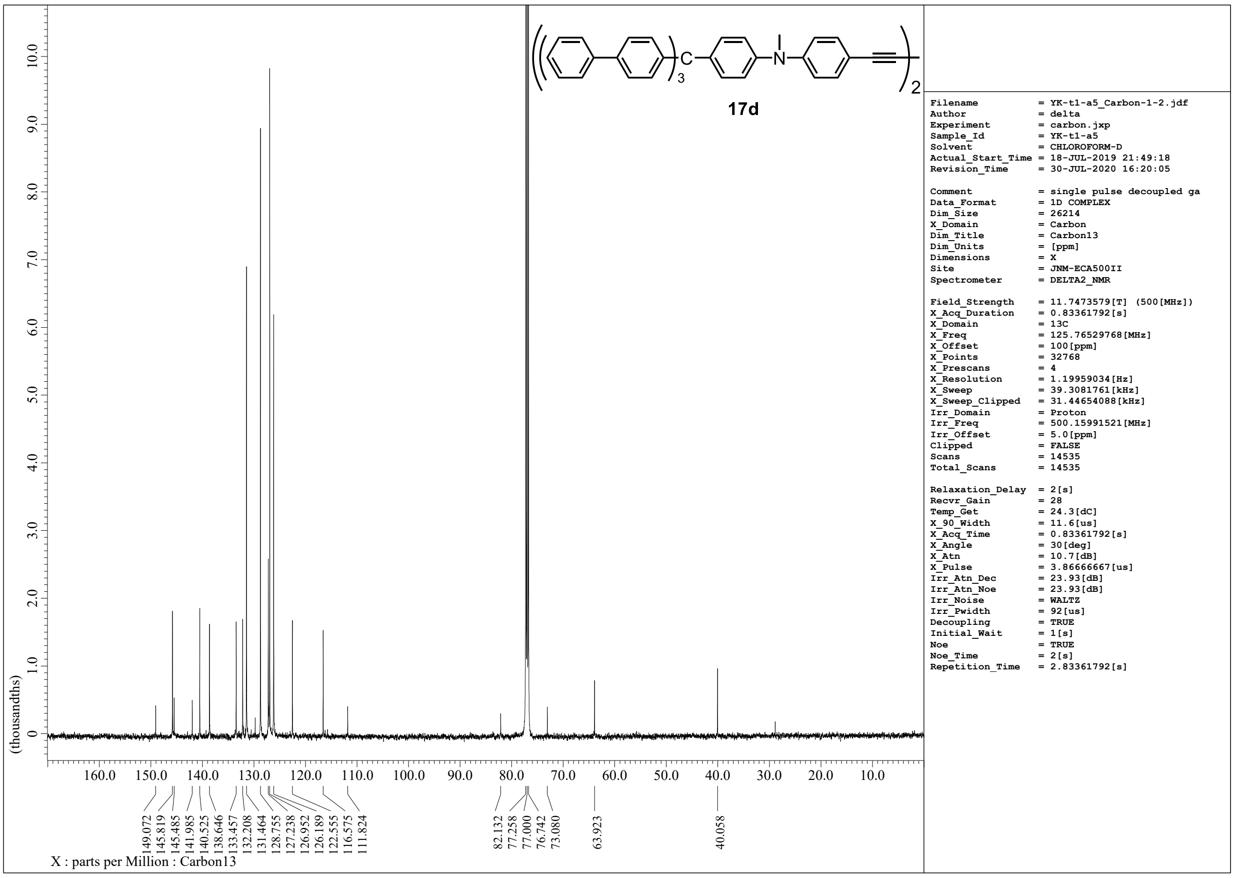

^{1}H NMR Spectrum of **4e** ($CDCl_3$, 500 MHz).

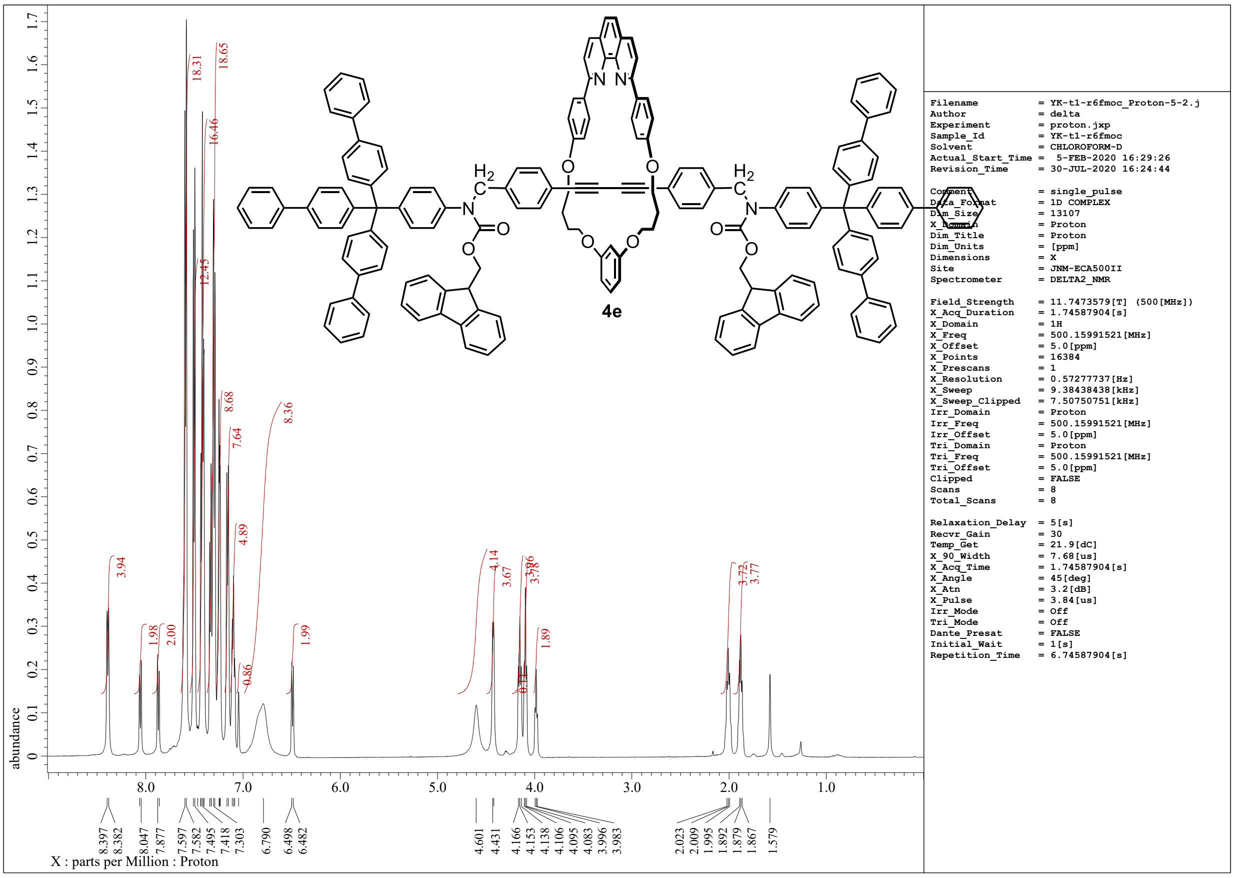

$^{13}C\{^{1}H\}$ NMR Spectrum of **4e** ($CDCl_3$, 126 MHz).

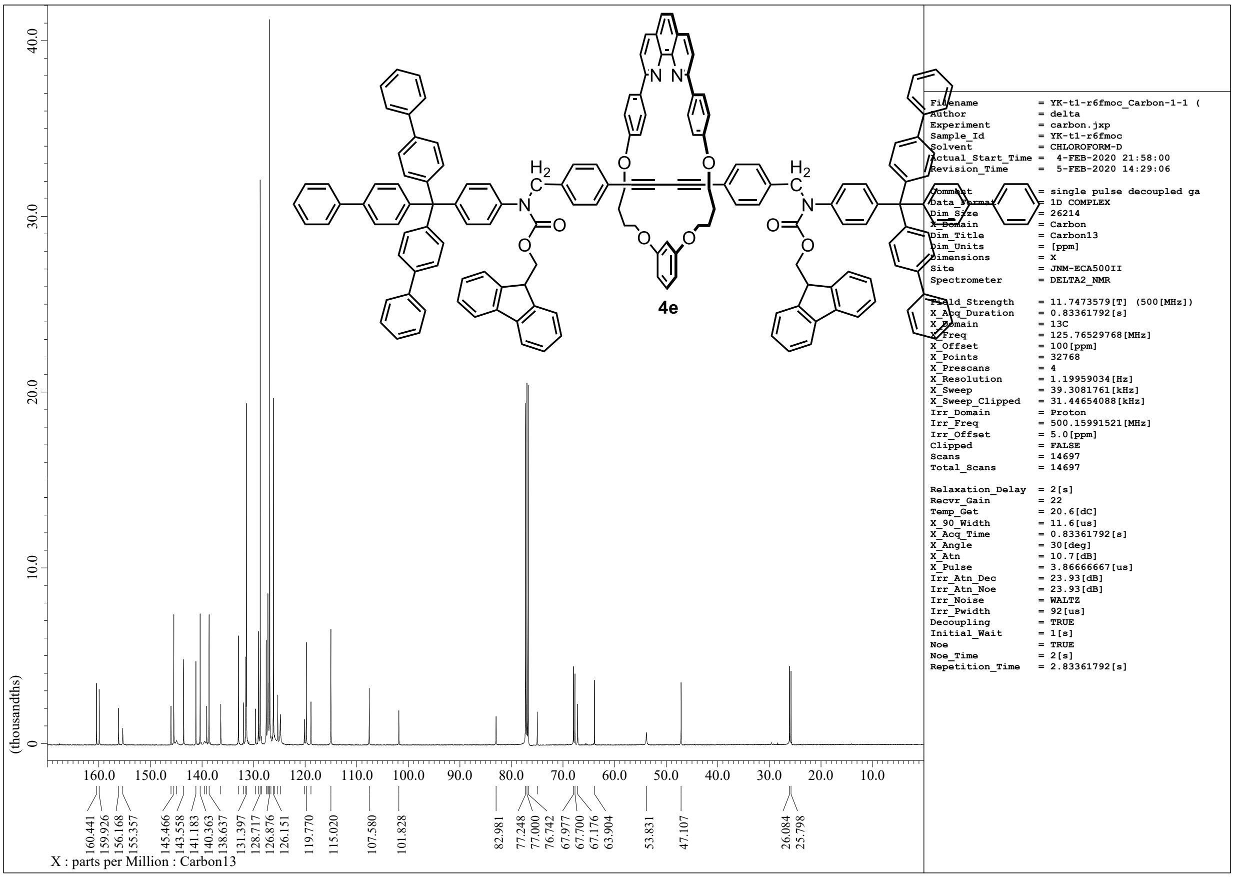

^{1}H NMR Spectrum of **4f** ($CDCl_3$, 500 MHz).

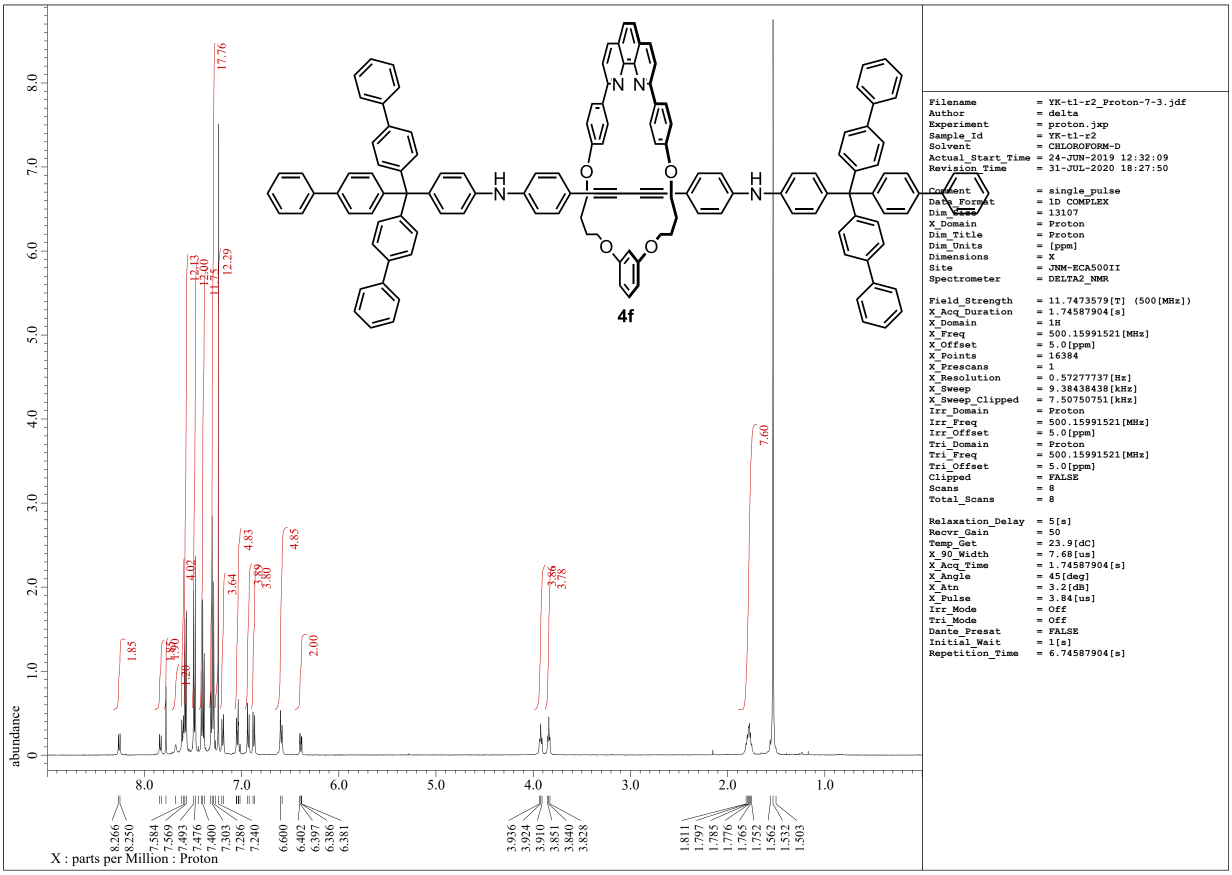

$^{13}C\{^{1}H\}$ NMR Spectrum of **4f** ($CDCl_3$, 126 MHz).

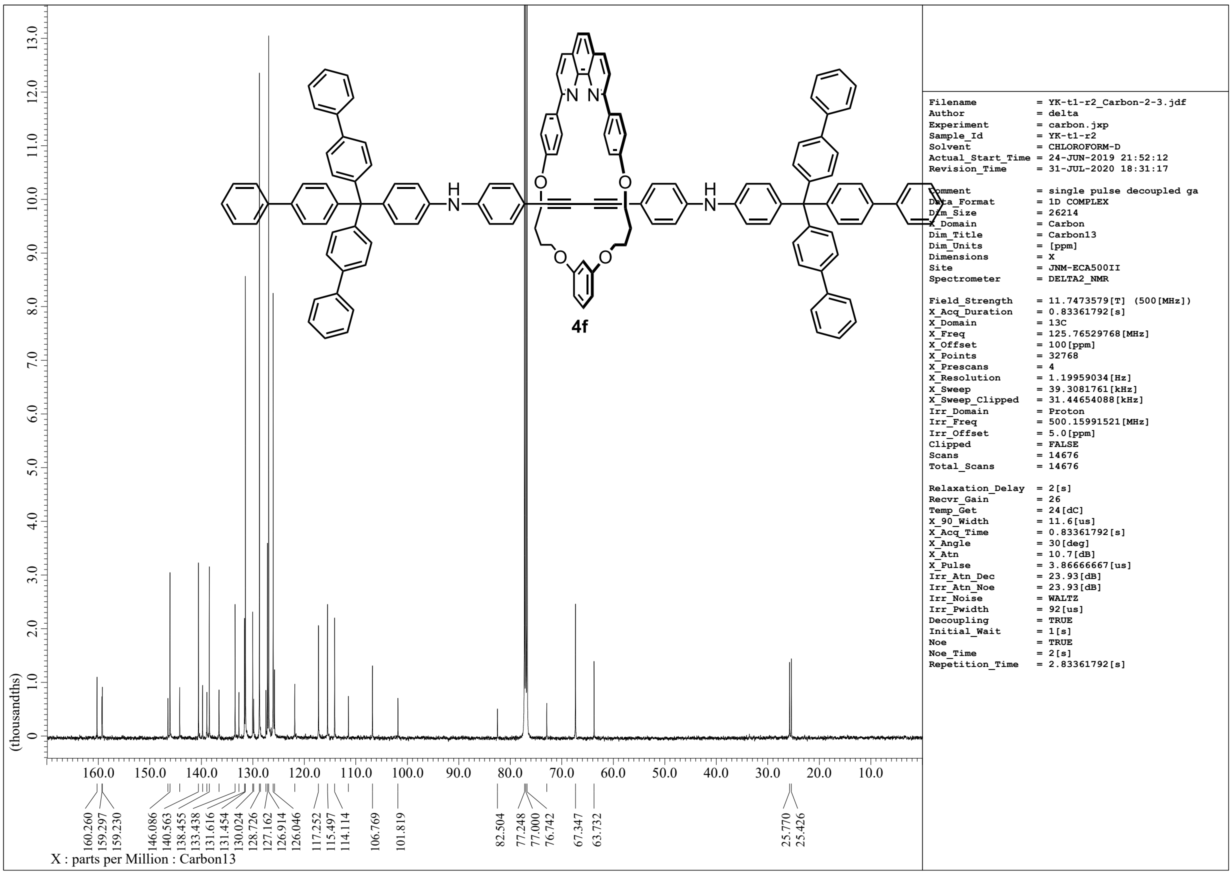

Partial ^{1}H NMR Spectrum of **4f** ($CDCl_3$, 500 MHz).

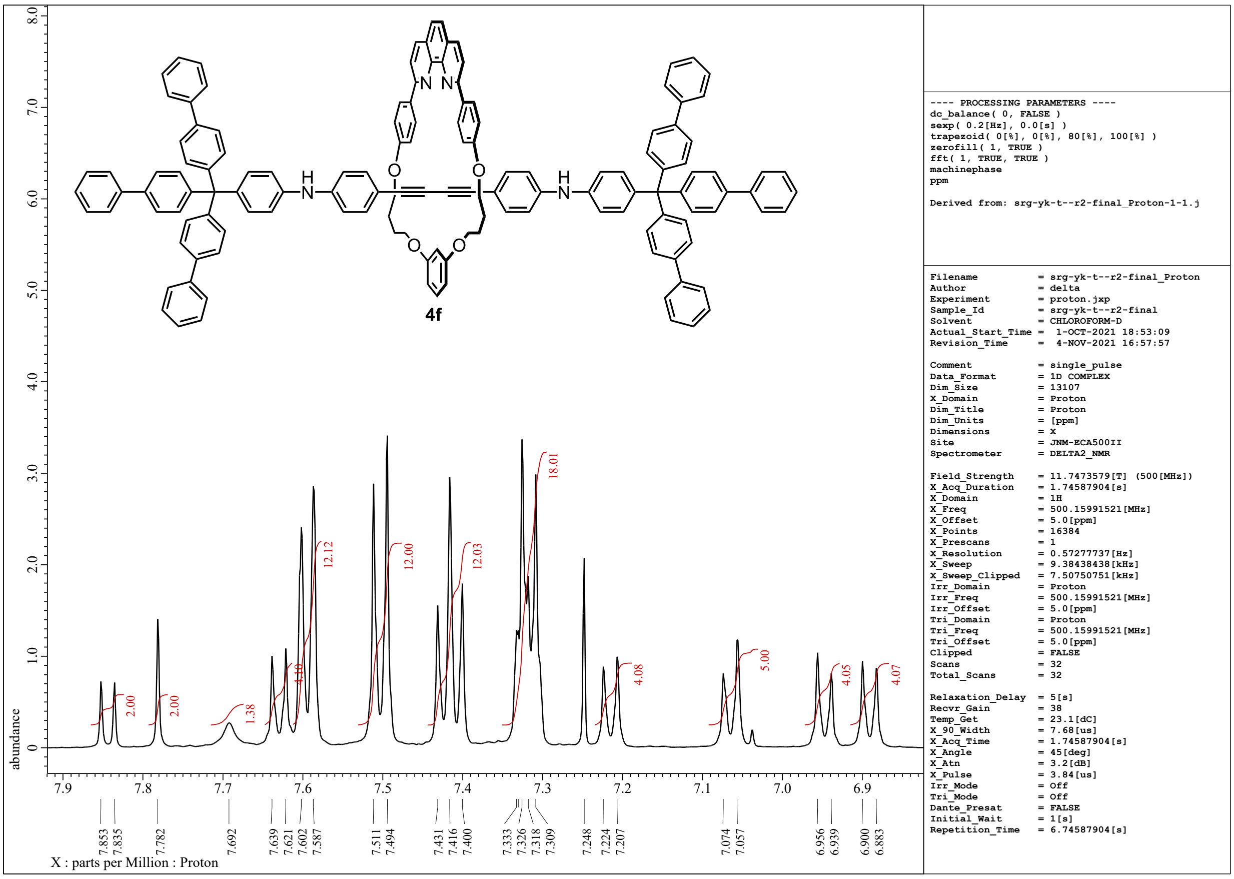

Partial ^{1}H NMR Spectrum of **4f-*d*$_2$** ($CDCl_3$, 500 MHz).

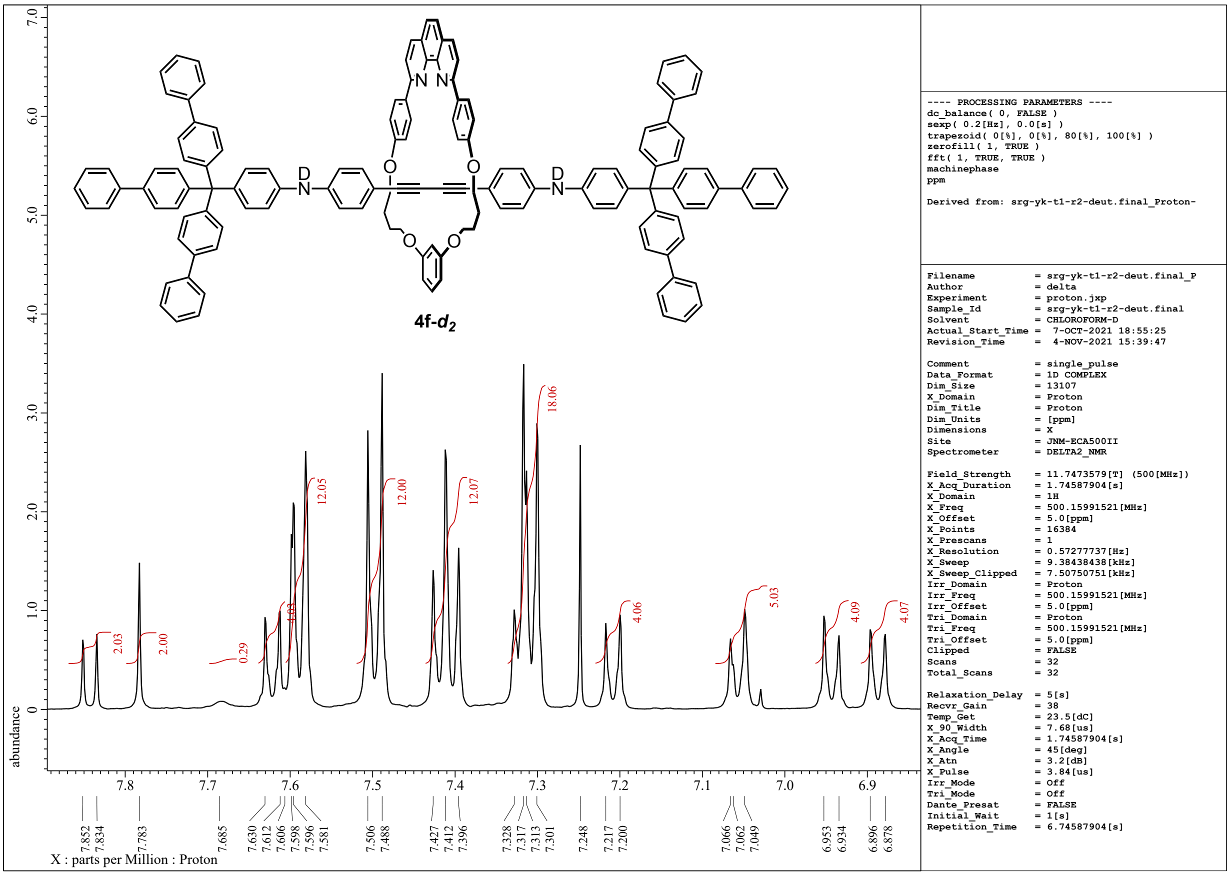

^{1}H NMR Spectrum of **17f** ($CDCl_3$, 500 MHz).

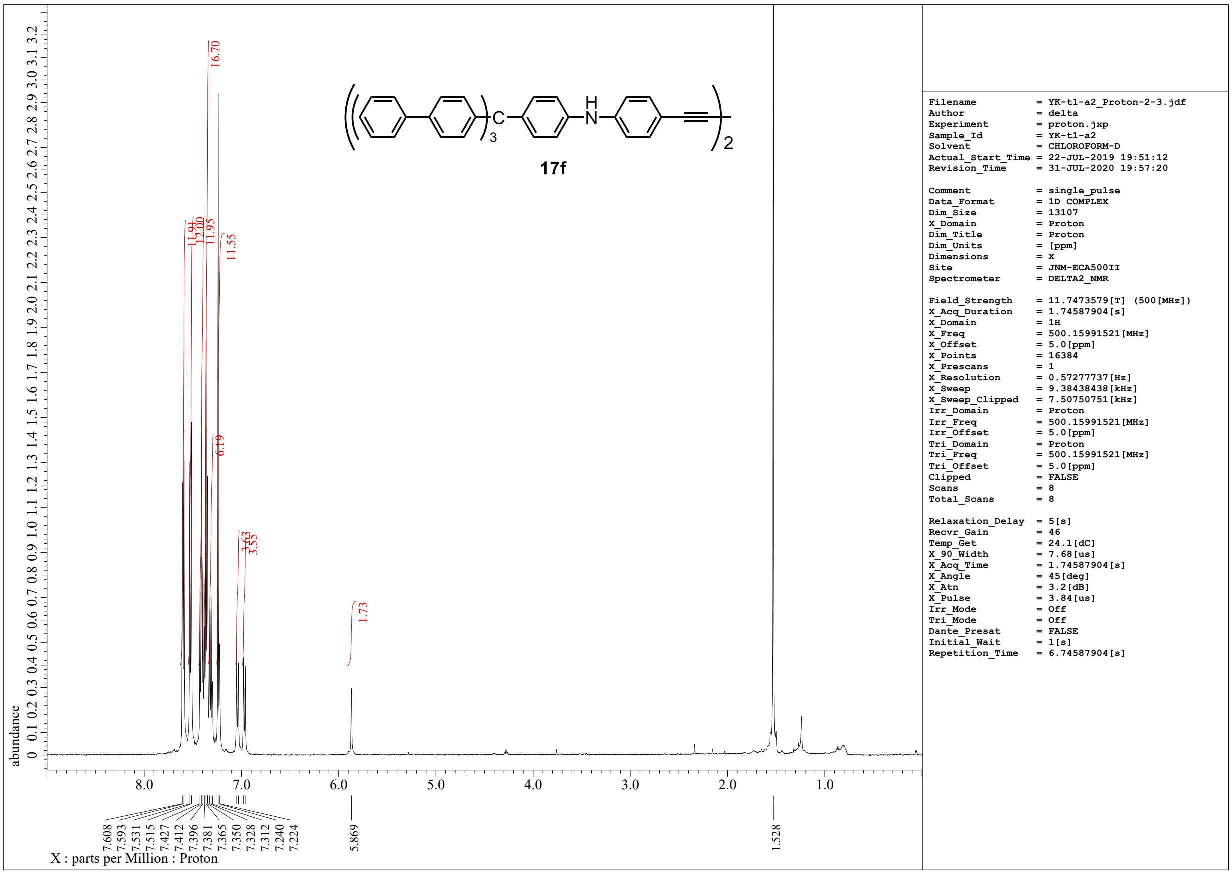

^{13}C{^{1}H} NMR Spectrum of **17f** ($CDCl_3$, 126 MHz).

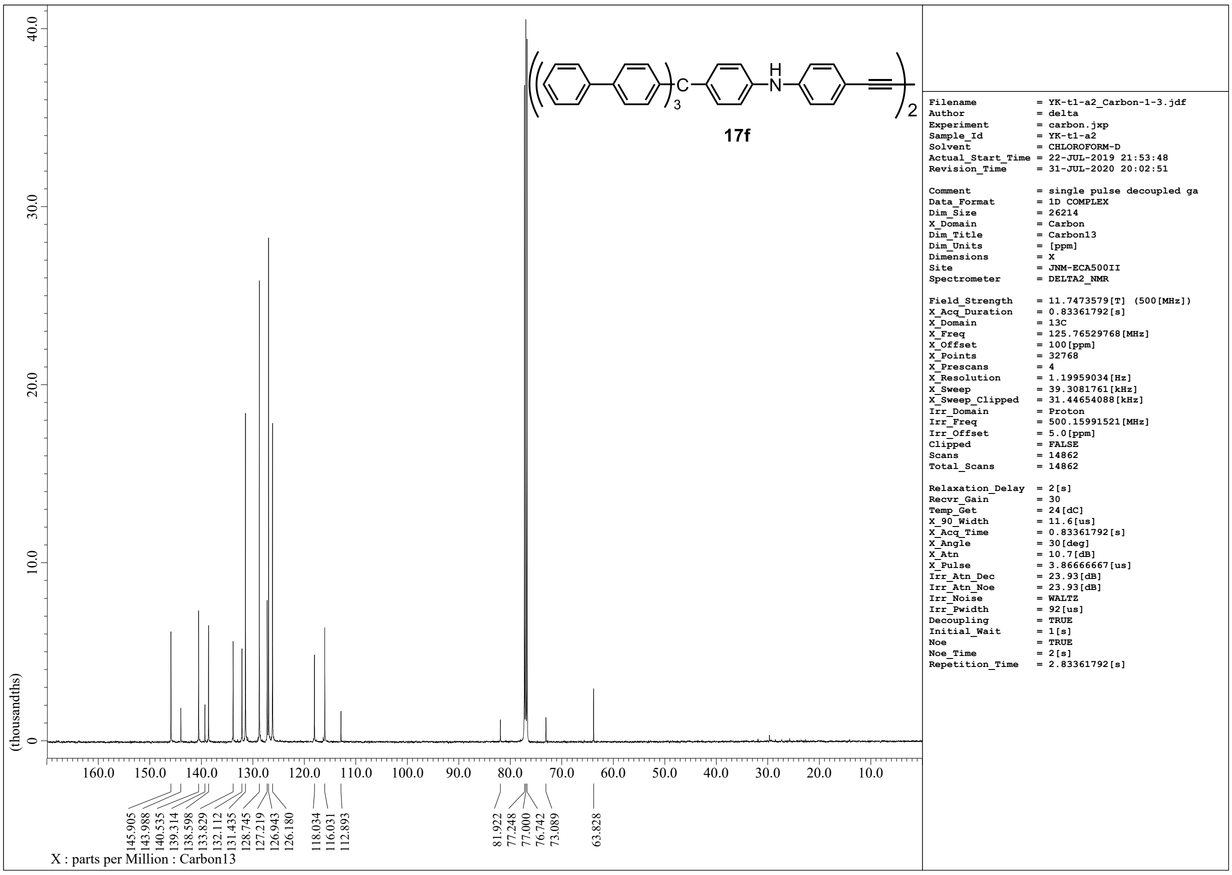

^{1}H NMR Spectrum of **4g** ($CDCl_3$, 500 MHz).

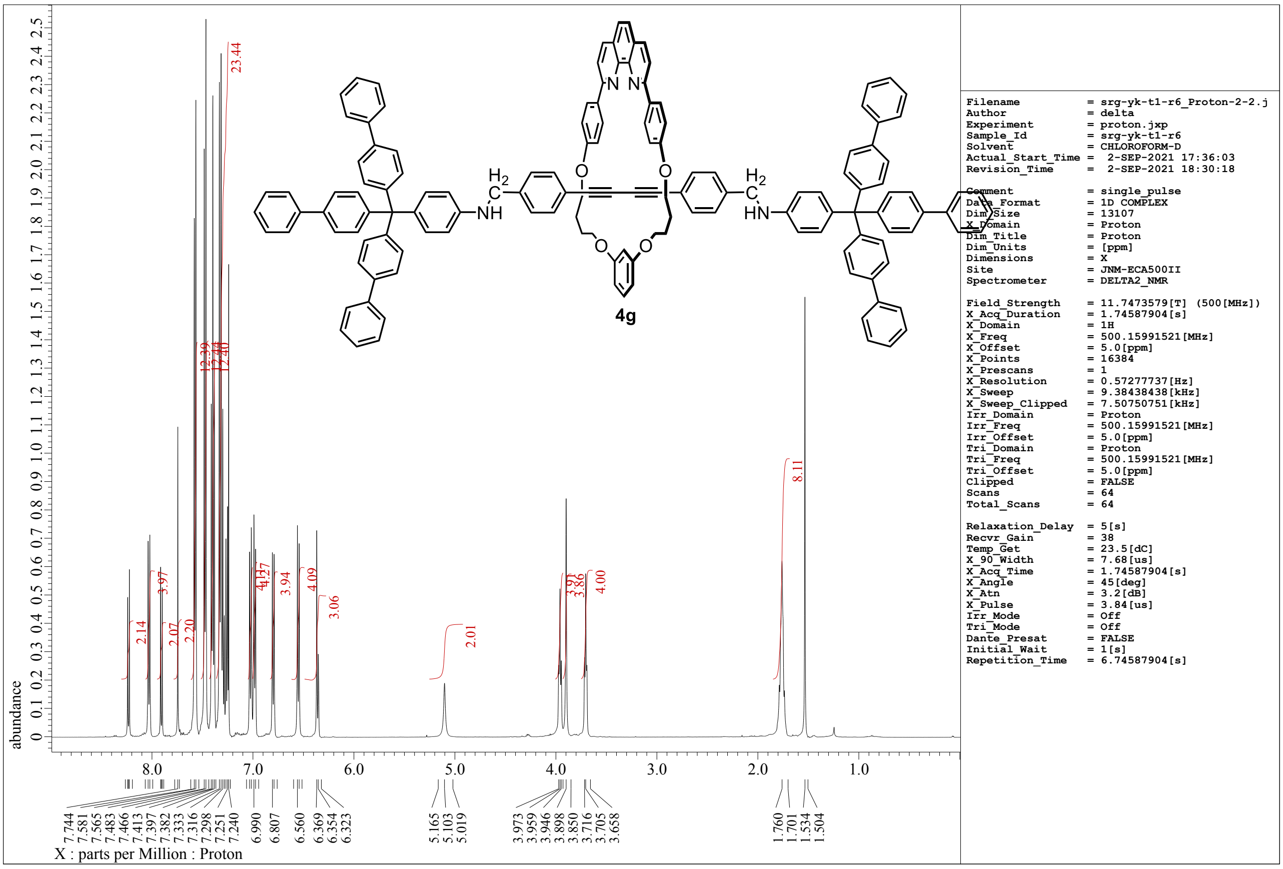

$^{13}C\{^{1}H\}$ NMR Spectrum of **4g** ($CDCl_3$, 100 MHz).

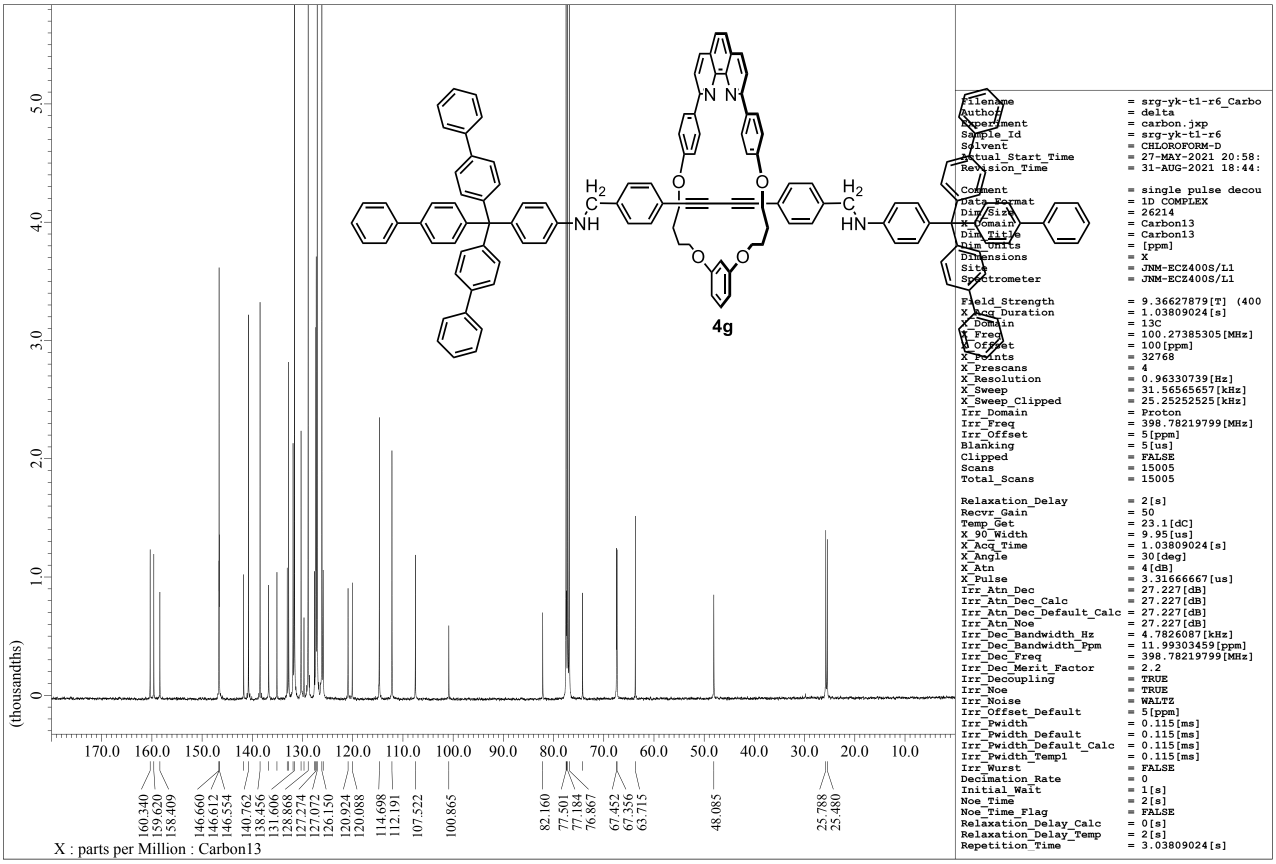

^{1}H NMR Spectrum of **17g** ($CDCl_3$, 500 MHz).

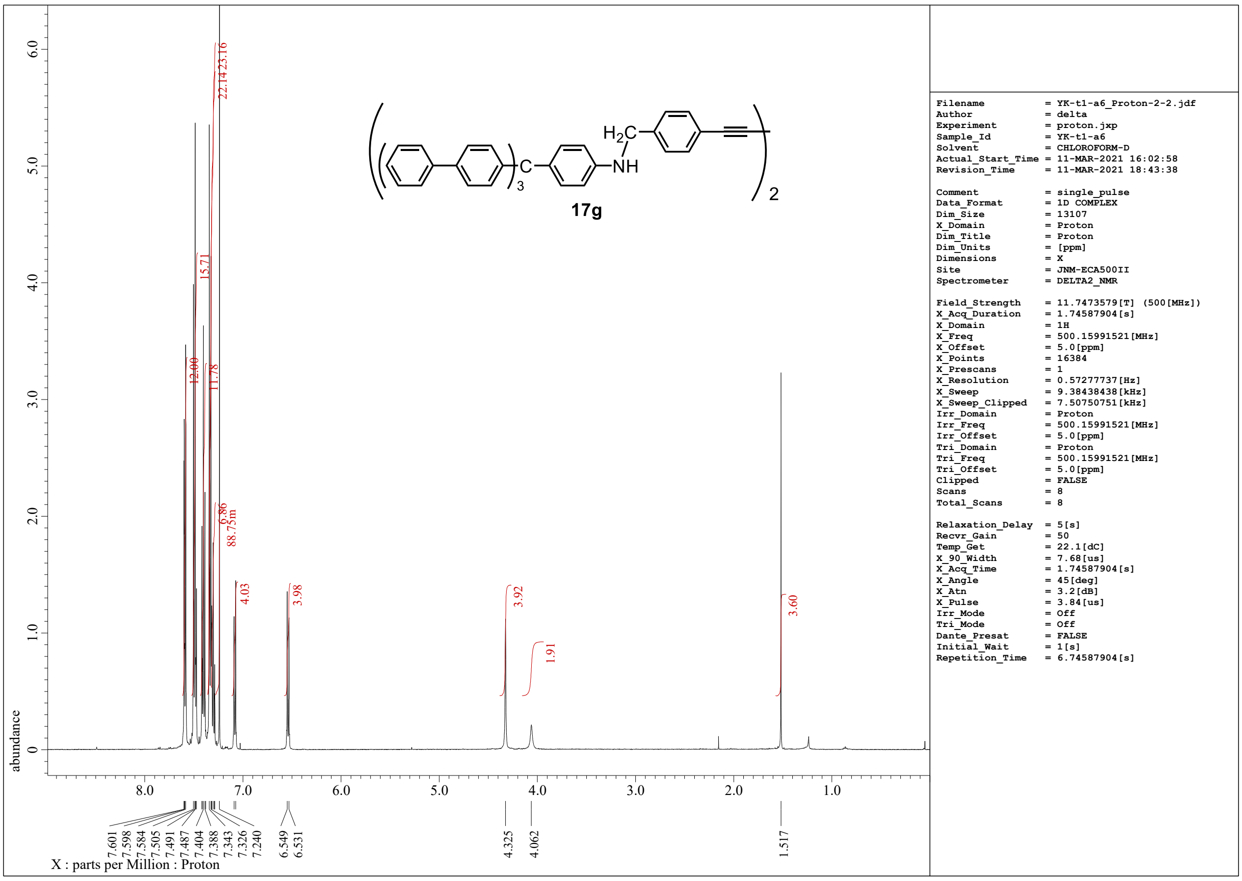

^{13}C{^{1}H} NMR Spectrum of **17g** ($CDCl_3$, 100 MHz).

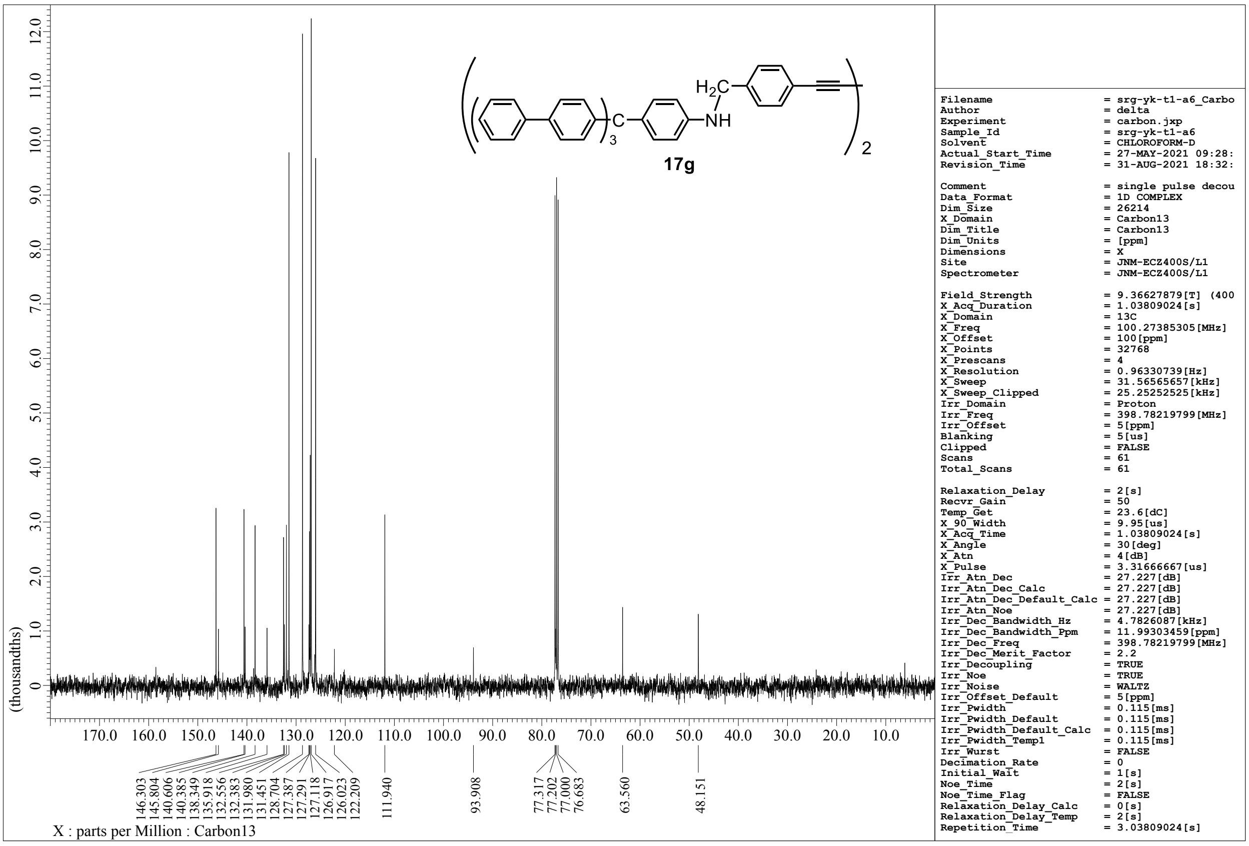

^{1}H NMR Spectrum of **4h** ($CDCl_3$, 500 MHz).

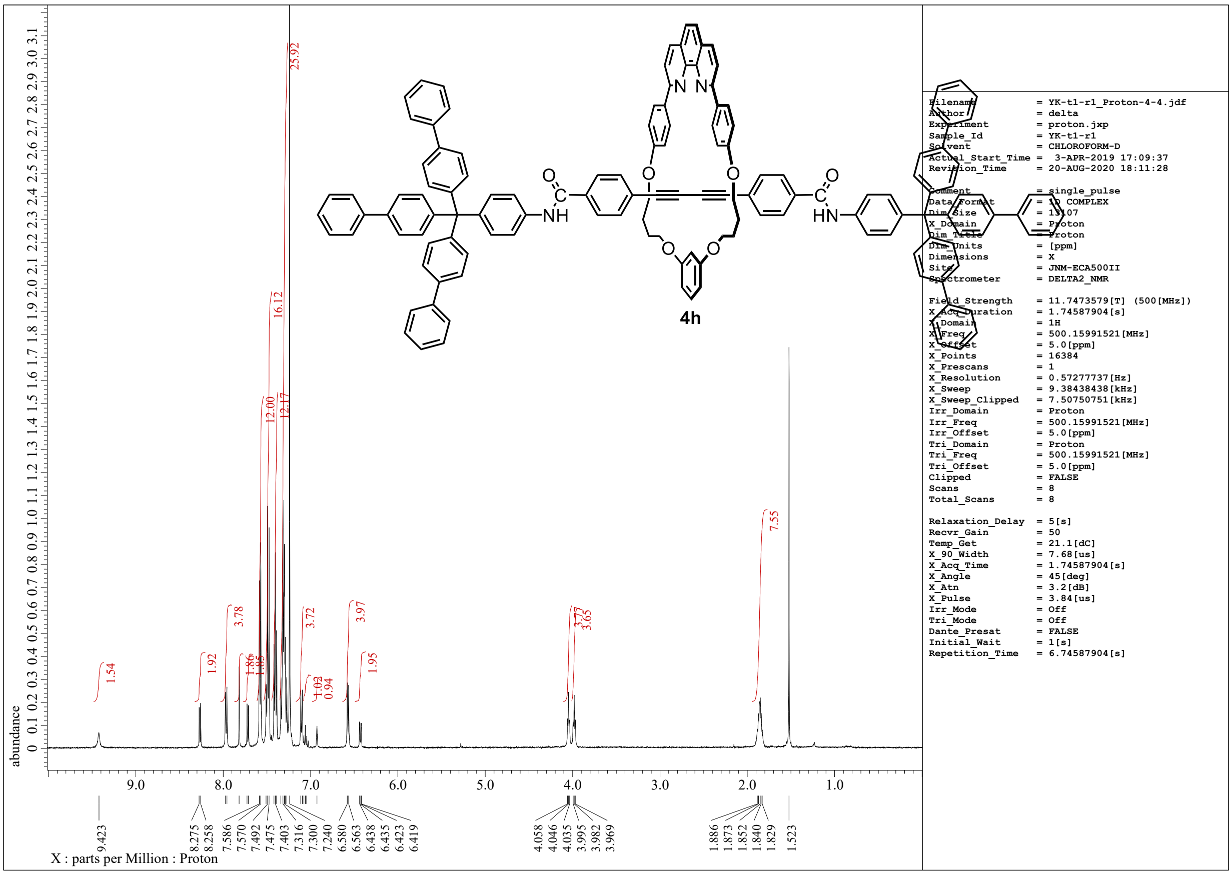

^{13}C{^{1}H} NMR Spectrum of **4h** ($CDCl_3$, 126 MHz).

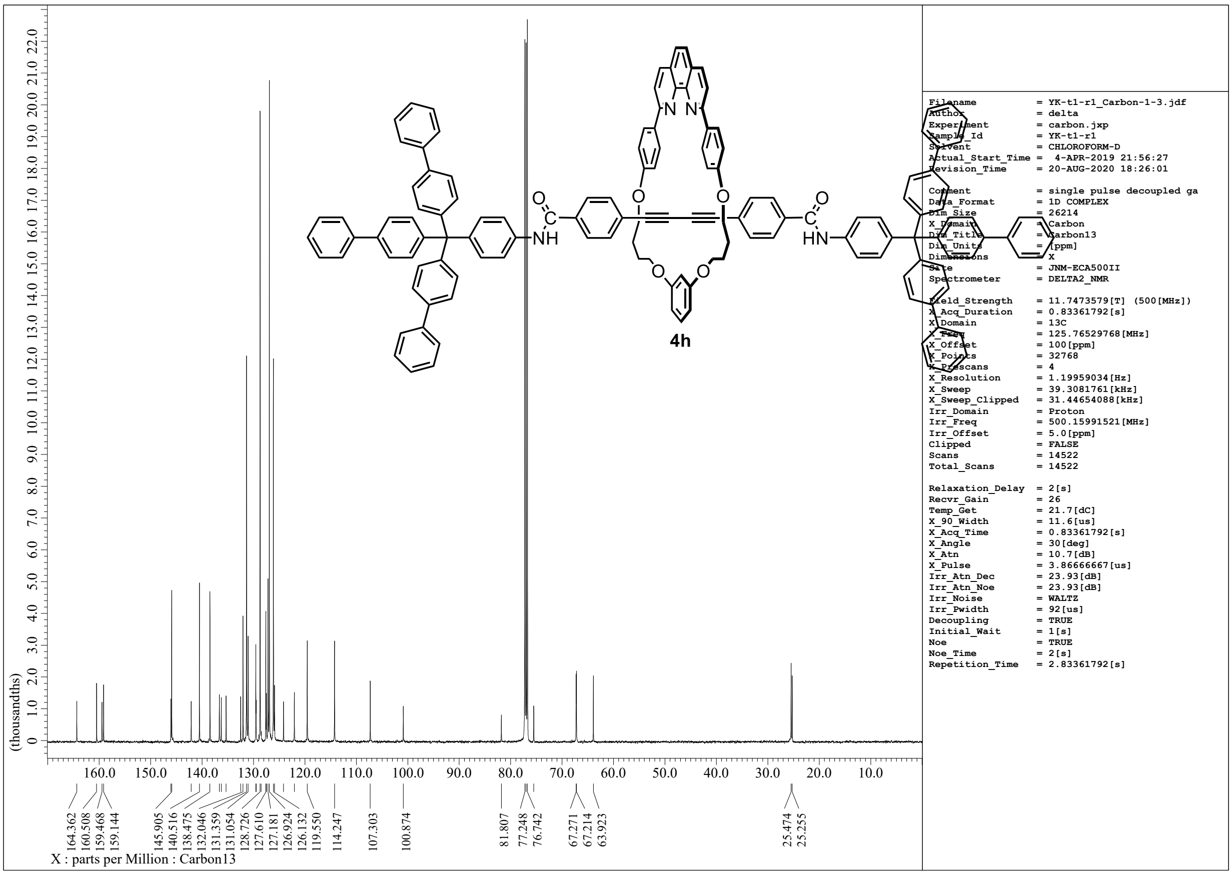

^{1}H NMR Spectrum of **17h** ($CDCl_3$, 500 MHz).

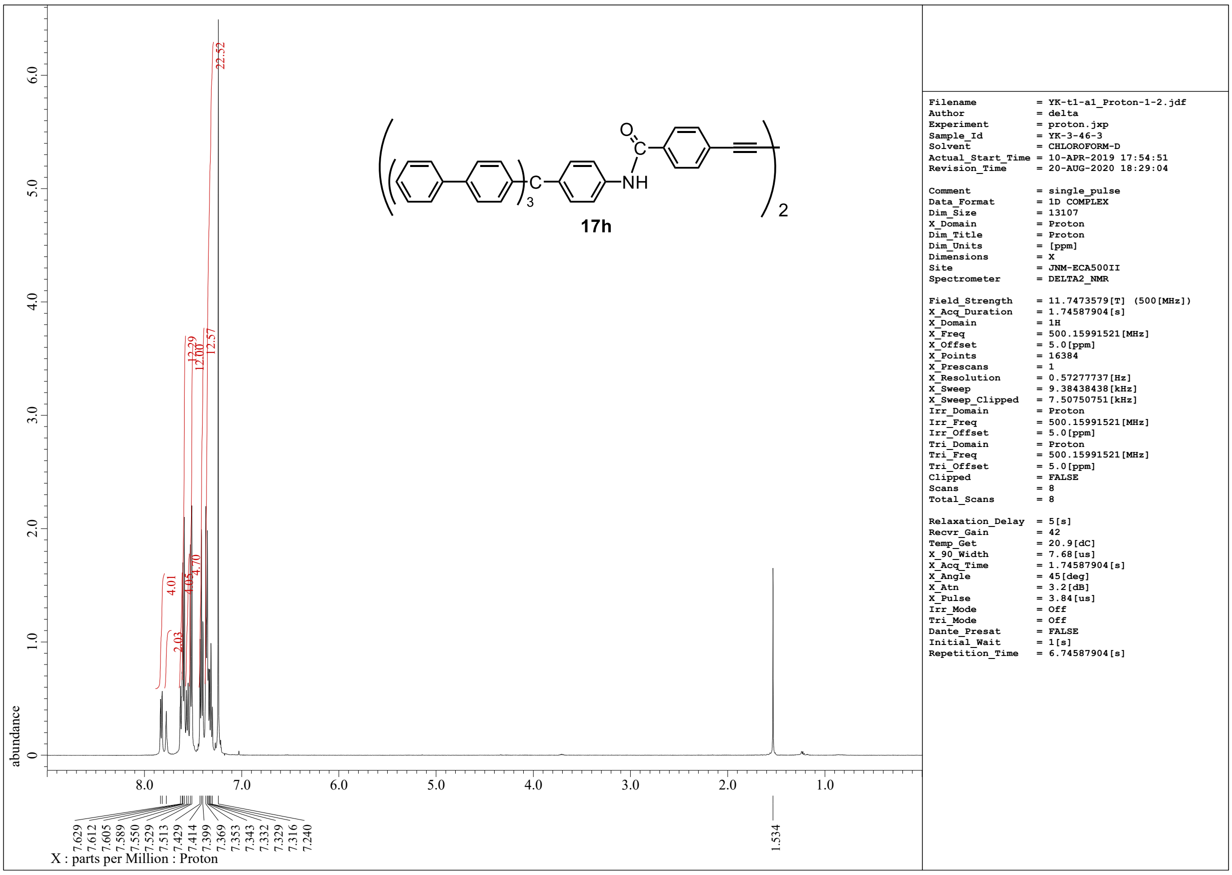

$^{13}C\{^{1}H\}$ NMR Spectrum of **17h** ($CDCl_3$, 126 MHz).

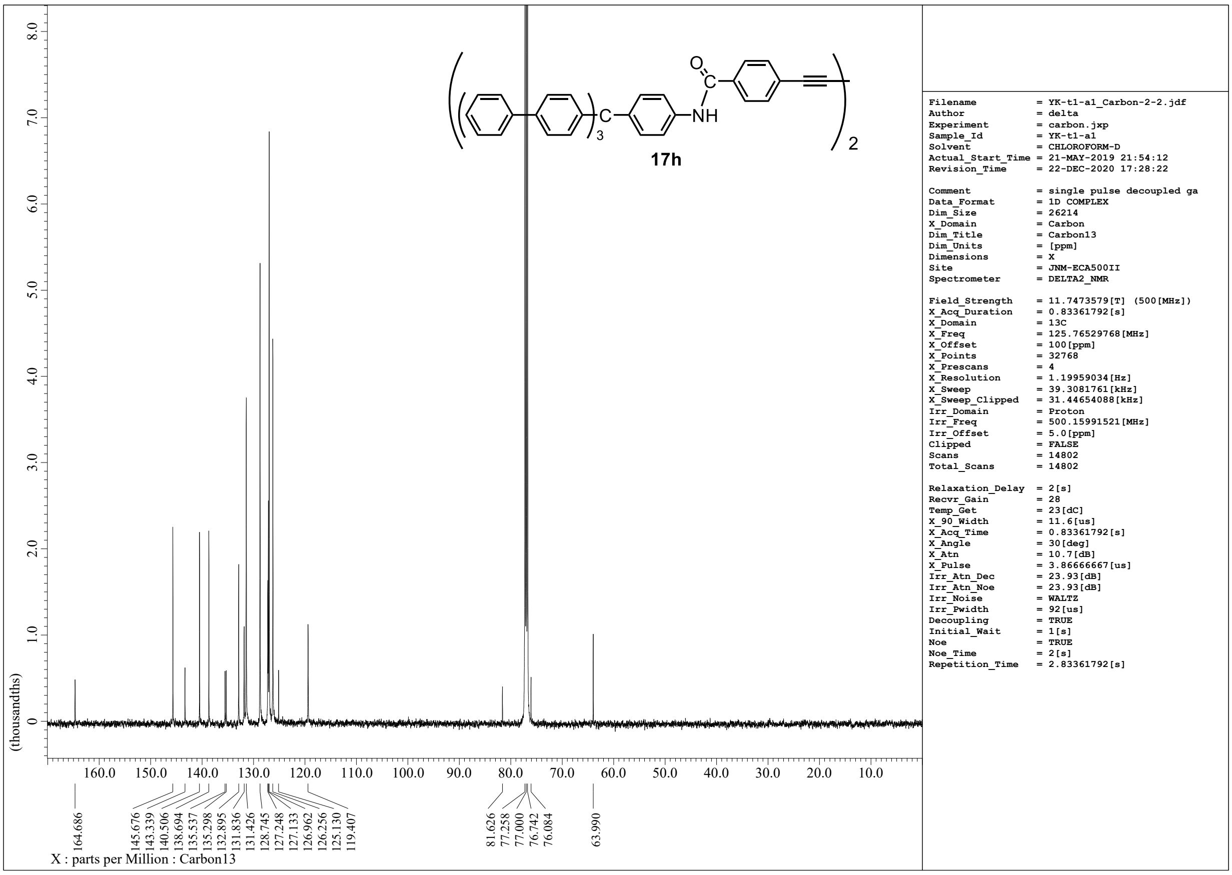

^{1}H NMR Spectrum of **4i** ($CDCl_3$, 500 MHz).

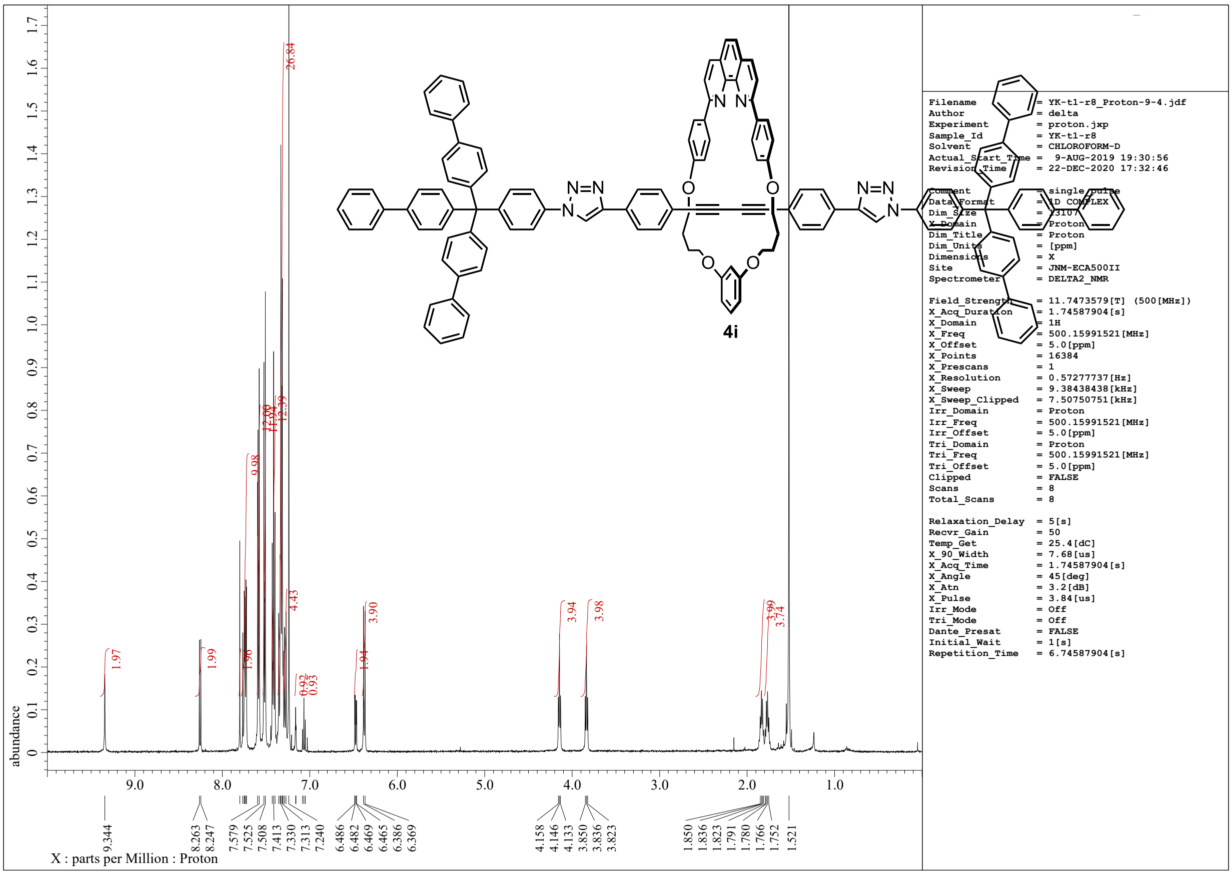

^{13}C{^{1}H} NMR Spectrum of **4i** ($CDCl_3$, 126 MHz).

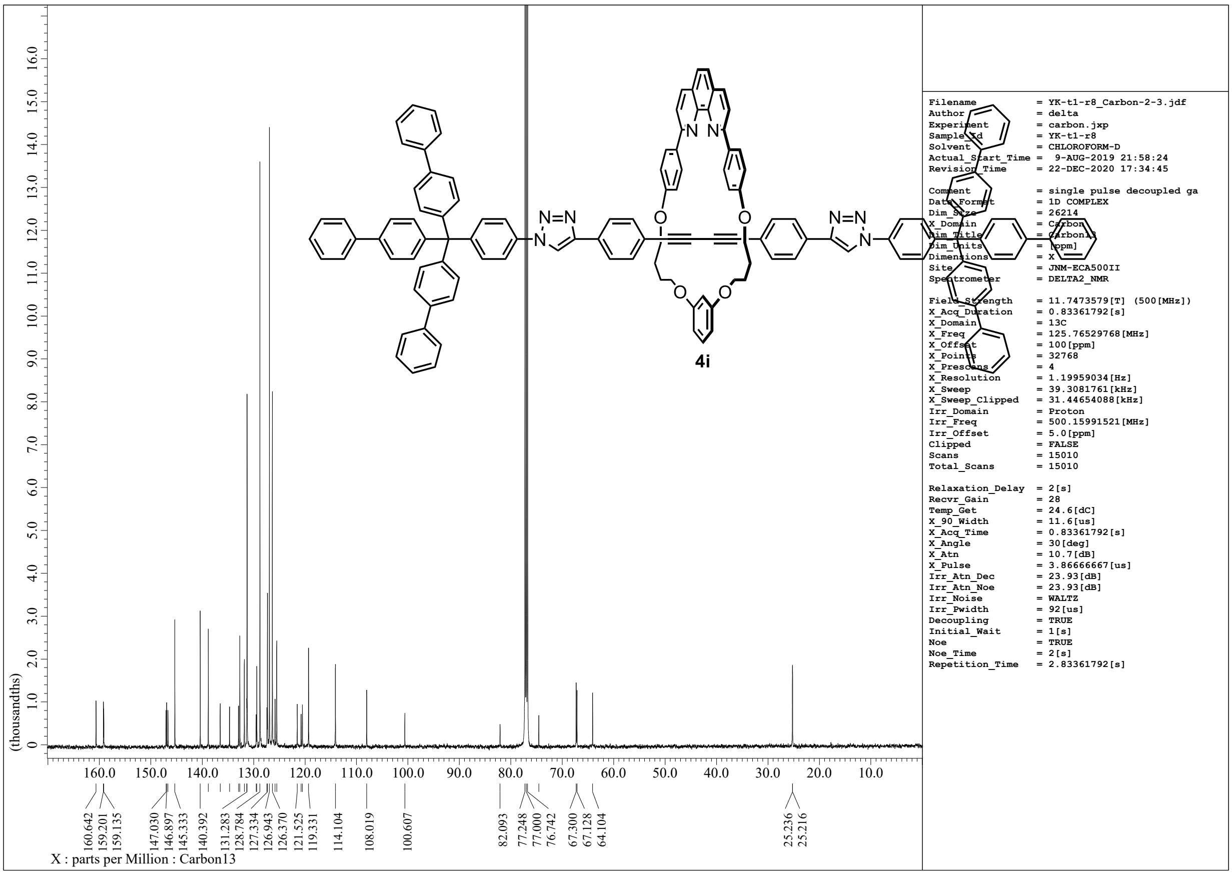

^{1}H NMR Spectrum of **17i** ($CDCl_3$, 500 MHz).

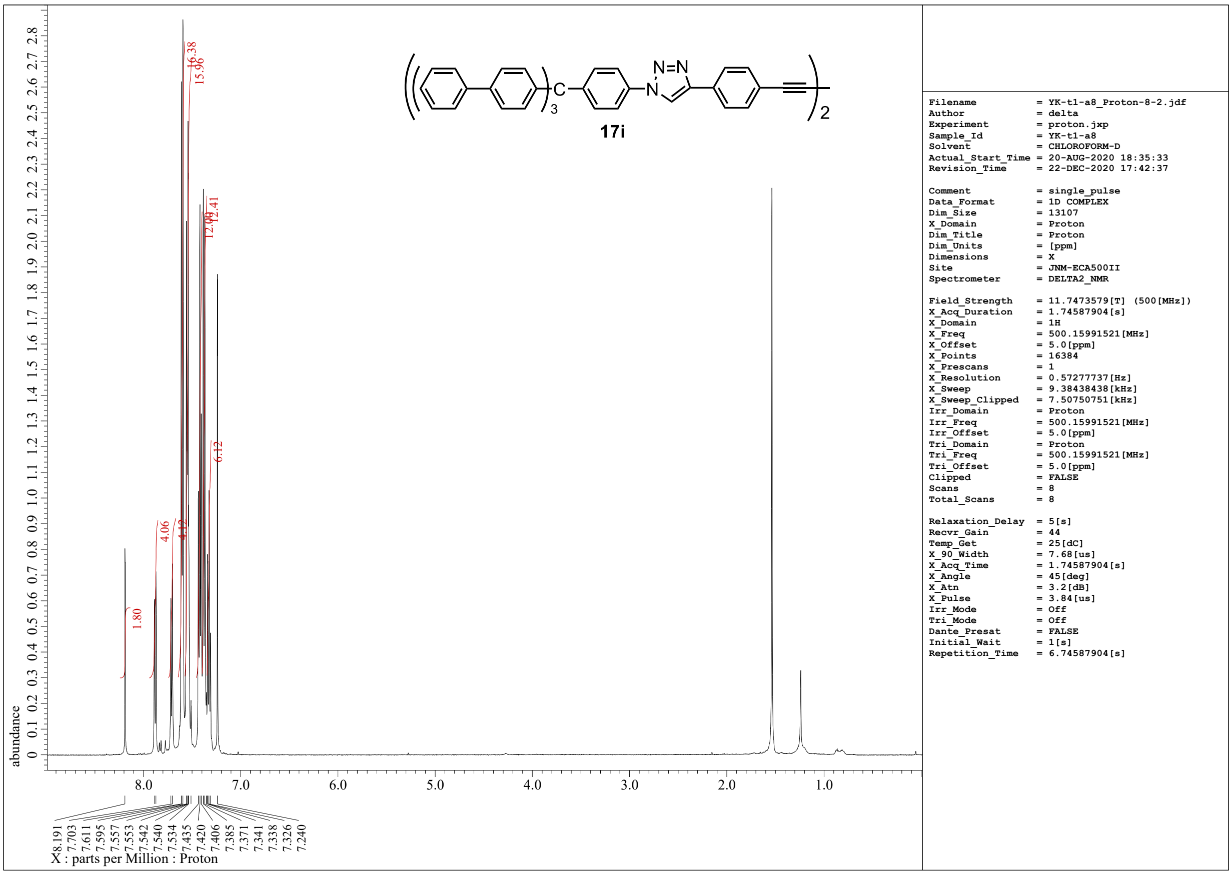

$^{13}C\{^{1}H\}$ NMR Spectrum of **17i** ($CDCl_3$, 100 MHz).

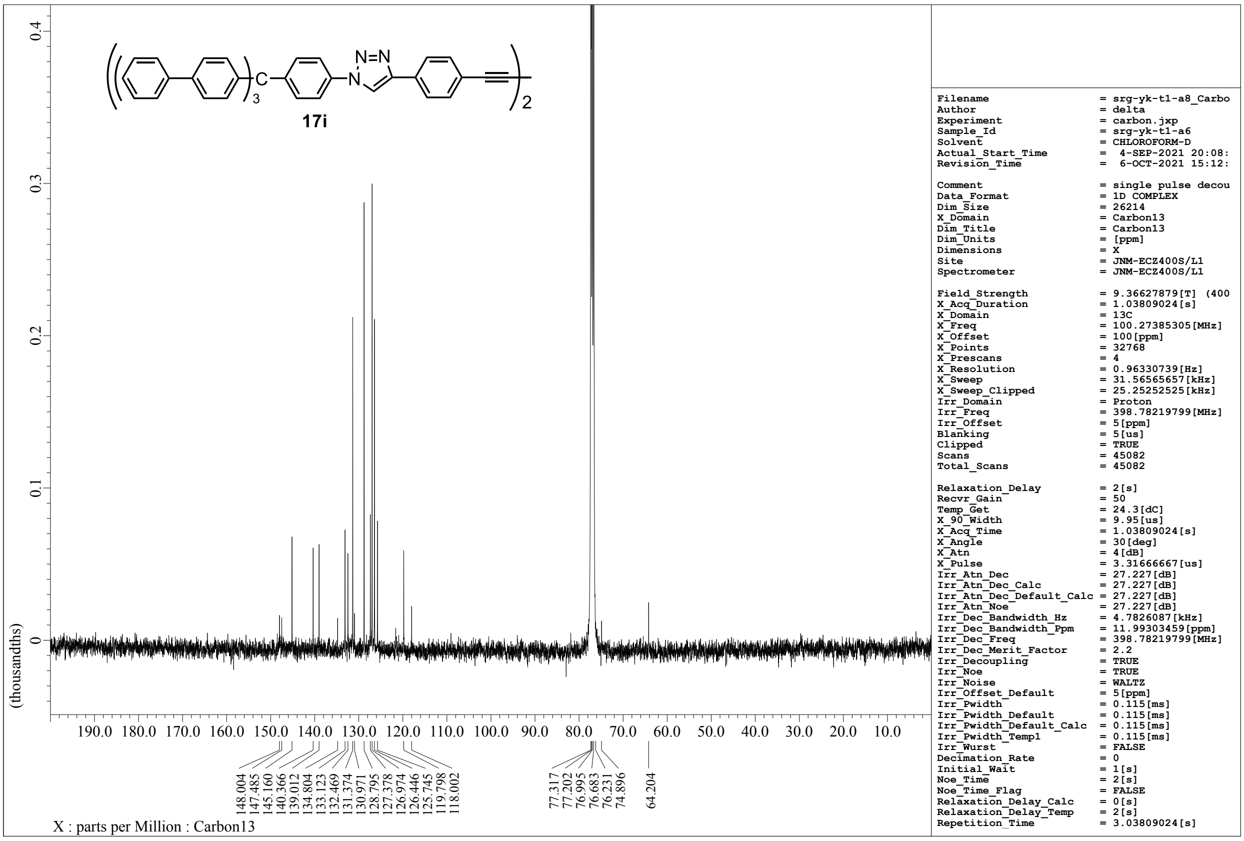

4. 2D NMR Spectra

^1H-^1H COSY NMR Spectrum of **4c** ($CDCl_3$, 400 MHz).

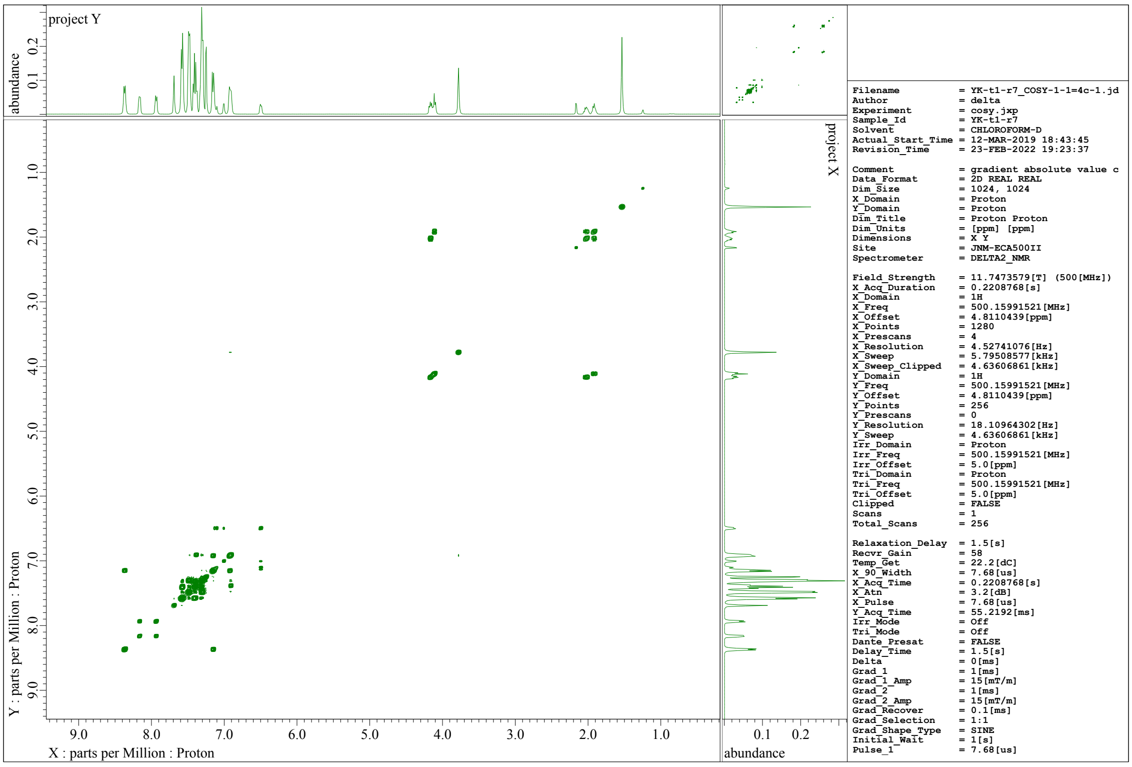

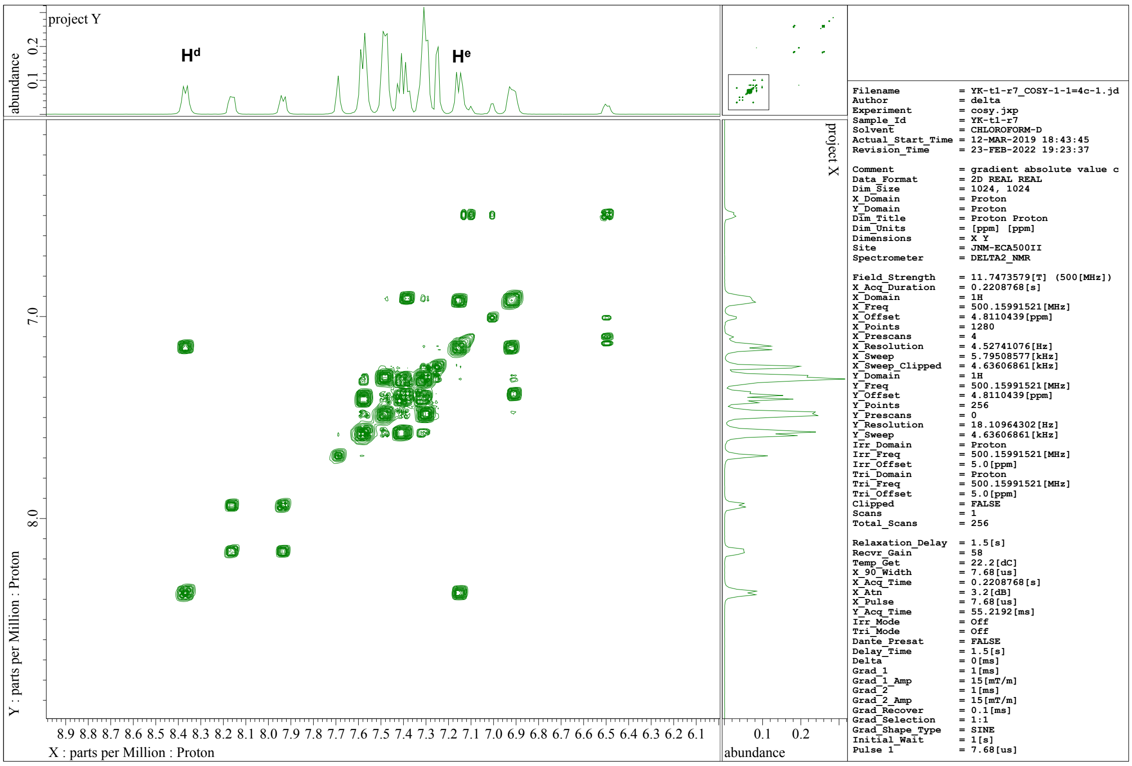

^1H-^1H COSY NMR Spectrum of **4f** ($CDCl_3$, 400 MHz).

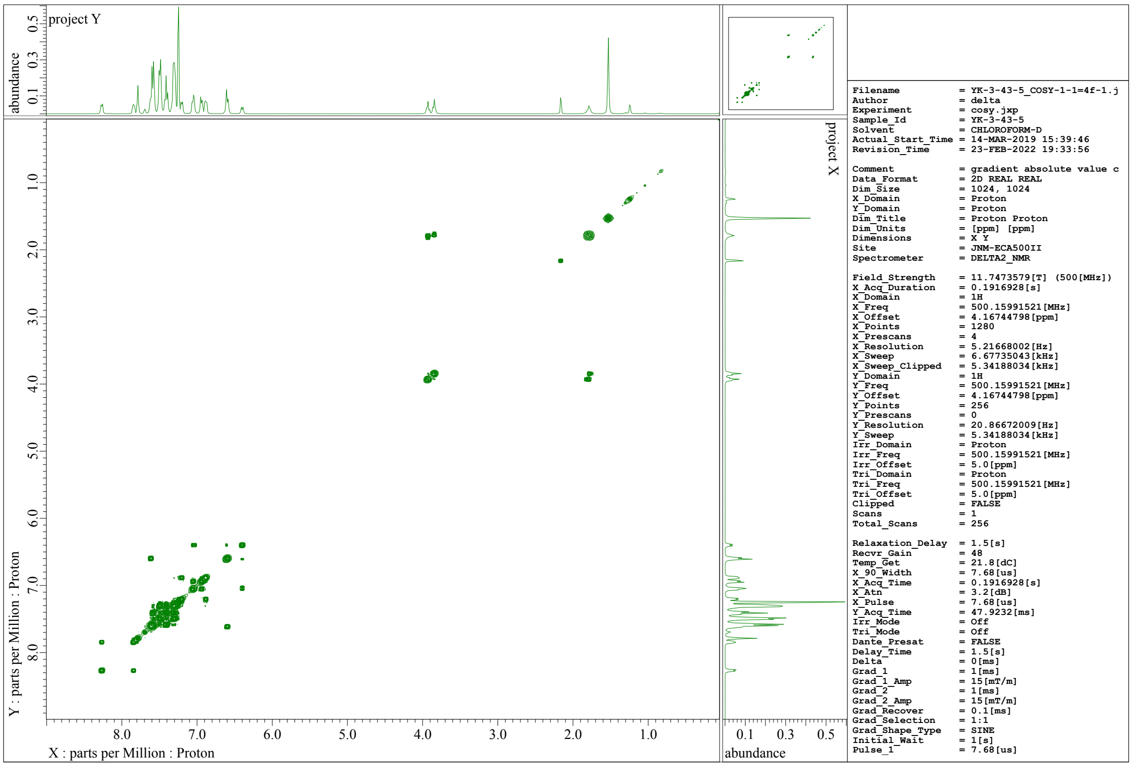

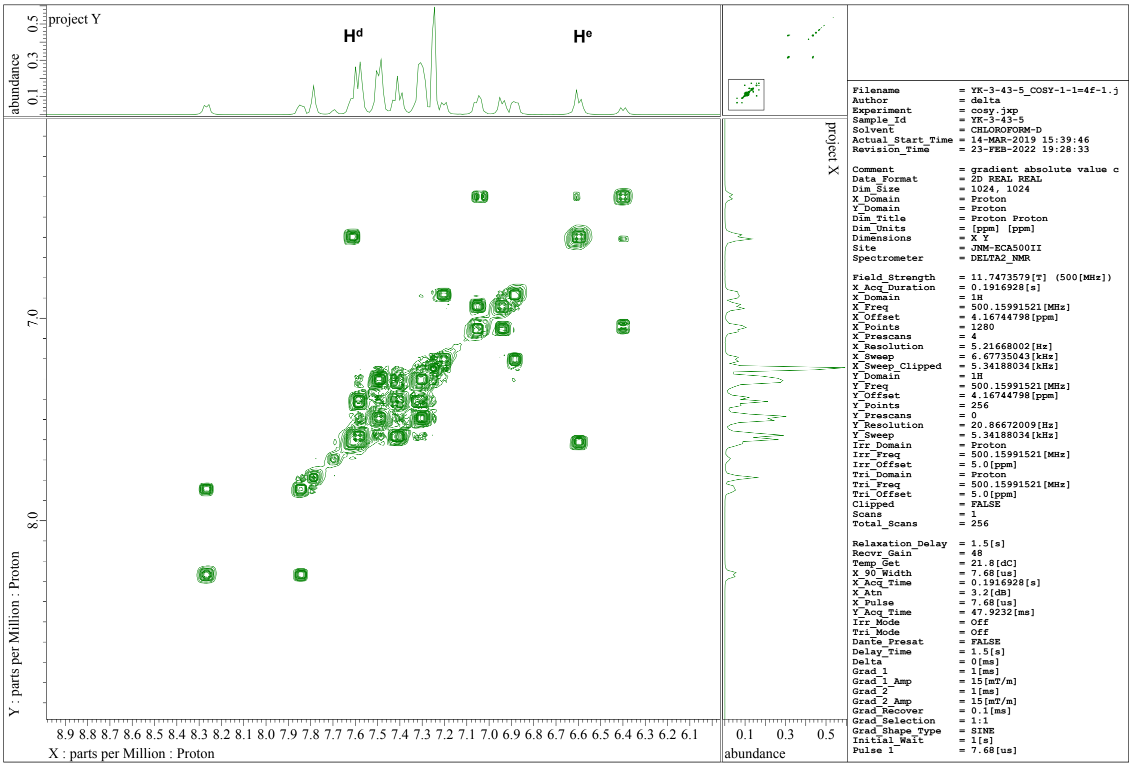

^1H-^1H NOESY NMR Spectrum of **4f** ($CDCl_3$, 400 MHz).

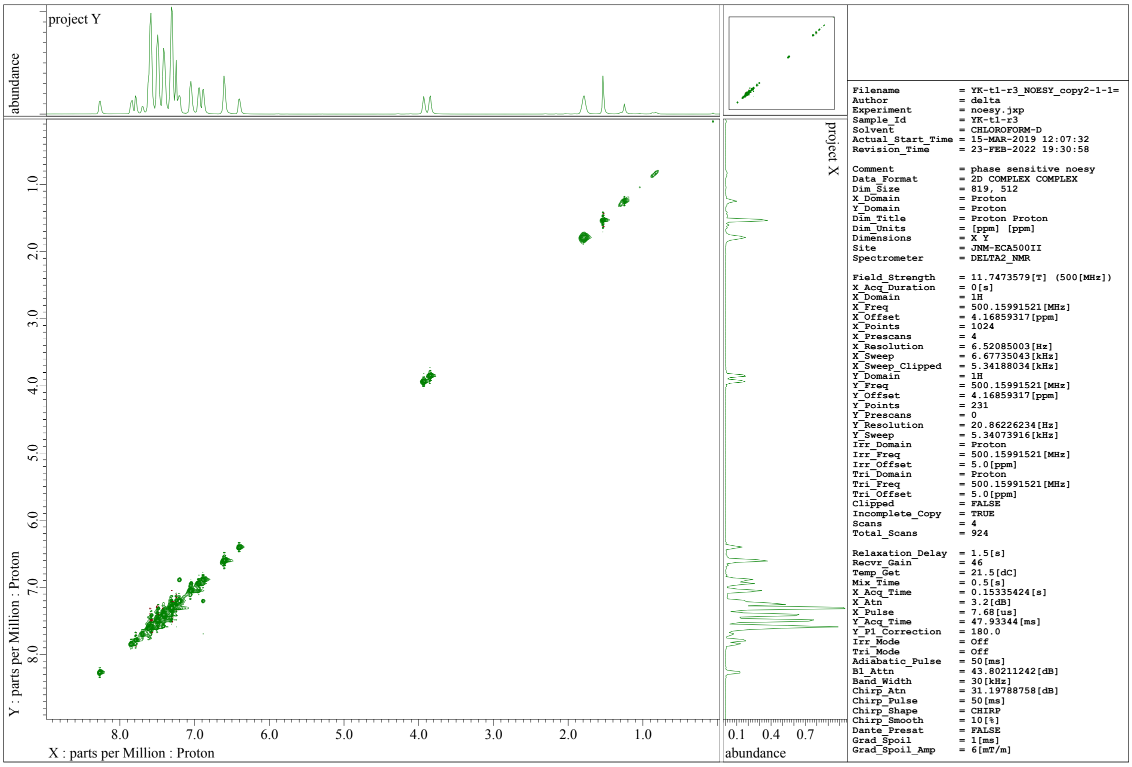

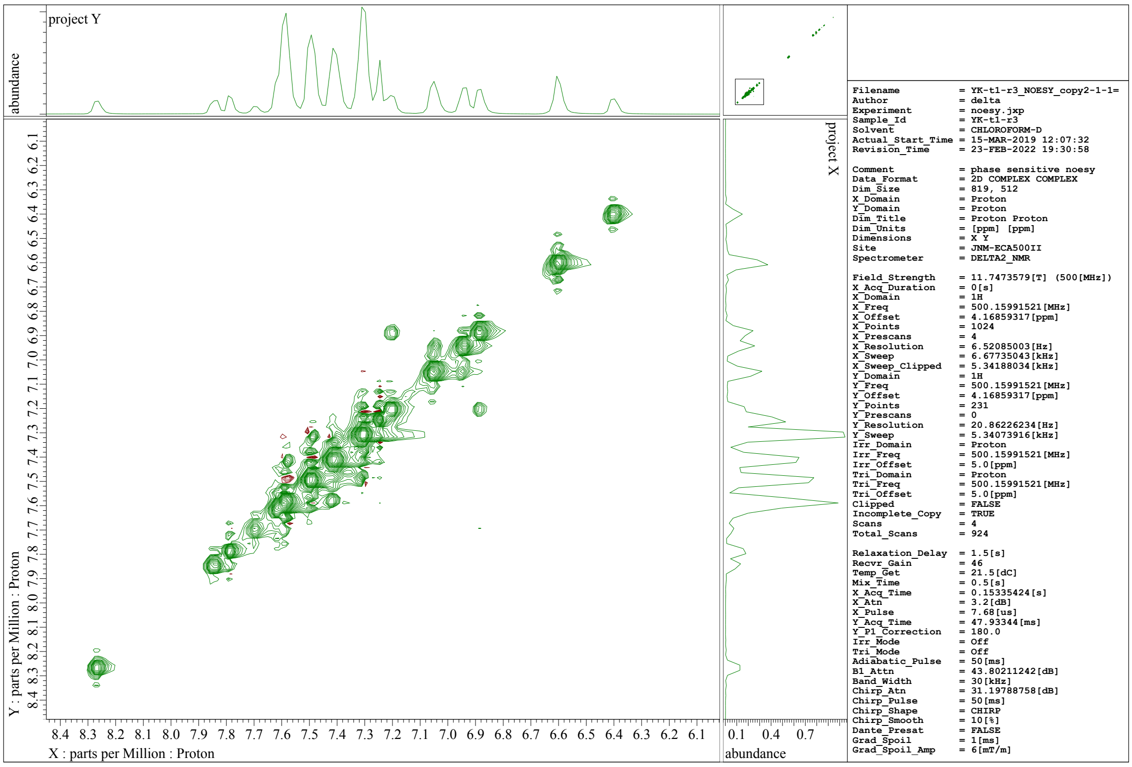

^{1}H-^{1}H EXSY NMR Spectra of **4f** (toluene-d_8, 400 MHz, 193 K).

Mixing time 100 ms

srg-yk-t1-r2_193K_100ms

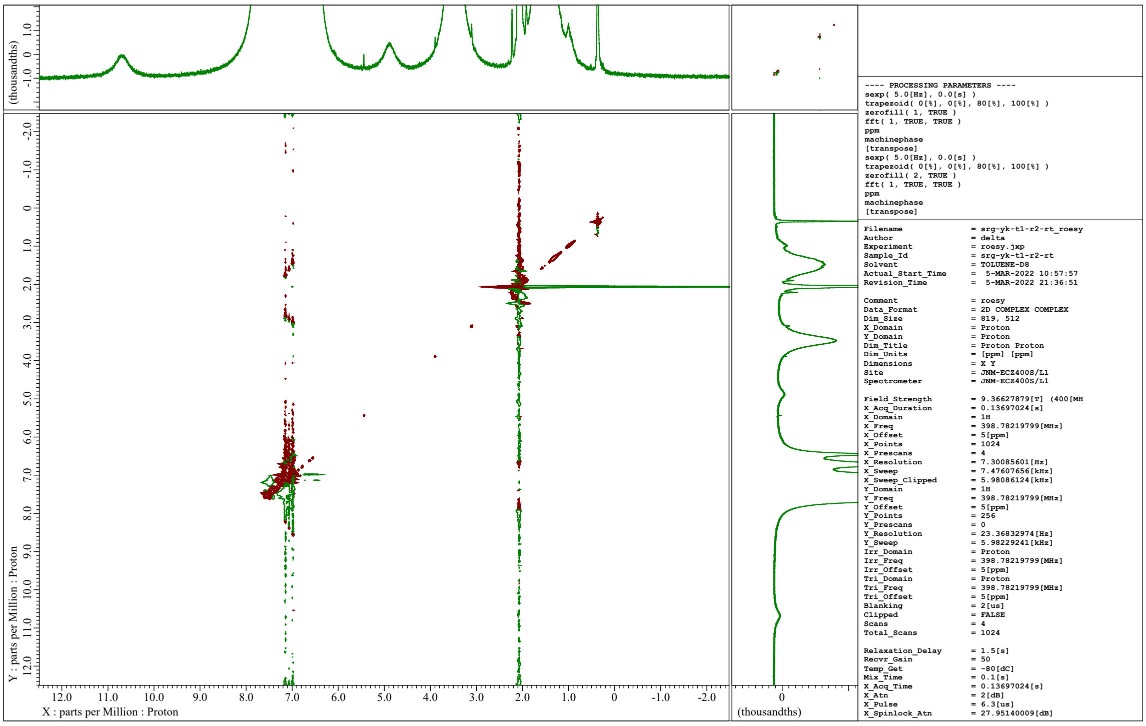

Mixing time 500 ms

srg-yk-t1-r2_193K_500ms

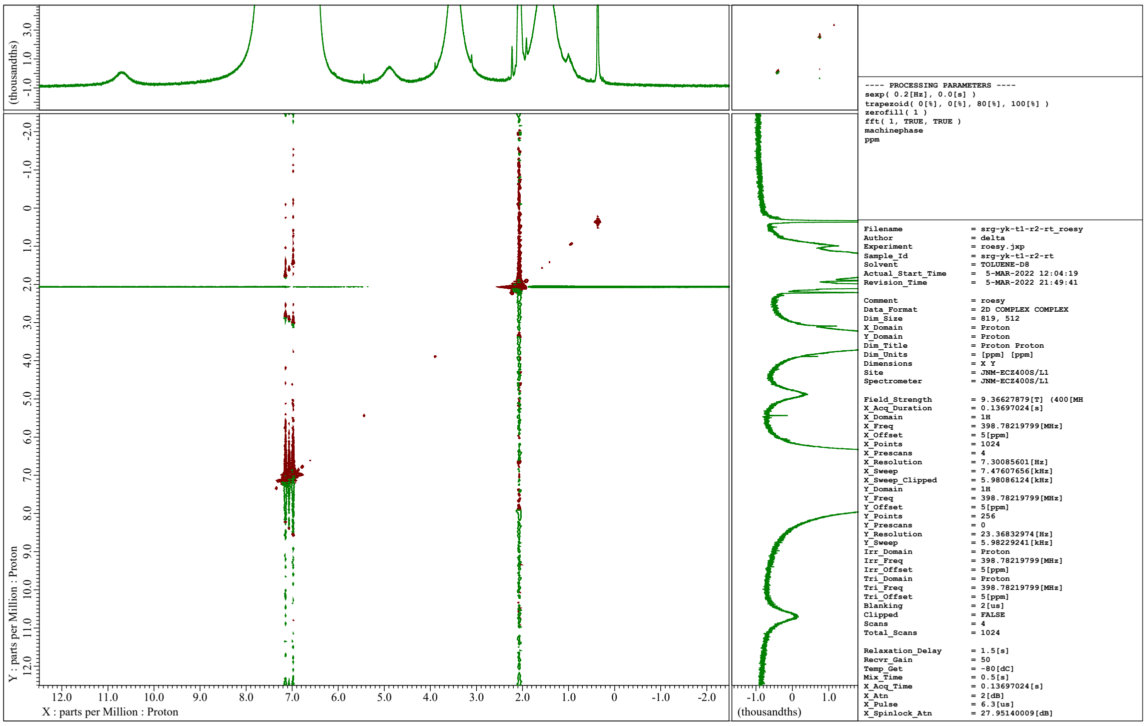

^{1}H-^{1}H COSY NMR Spectrum of **4g** ($CDCl_3$, 400 MHz).

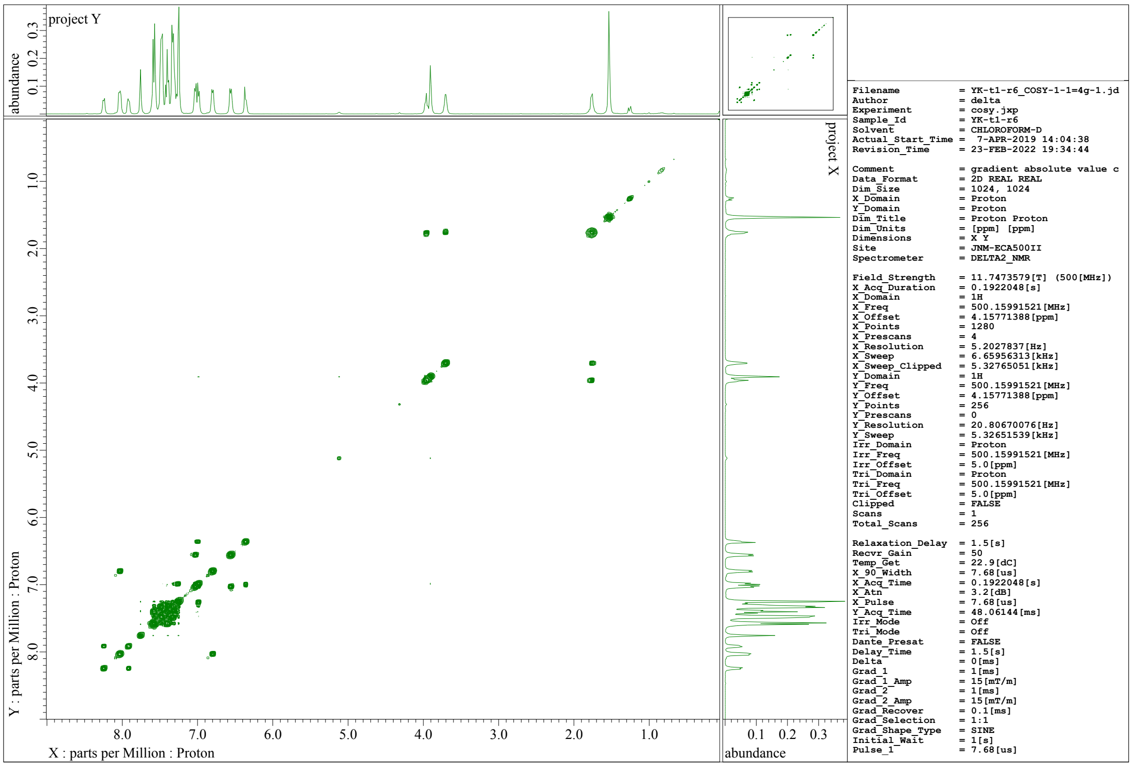

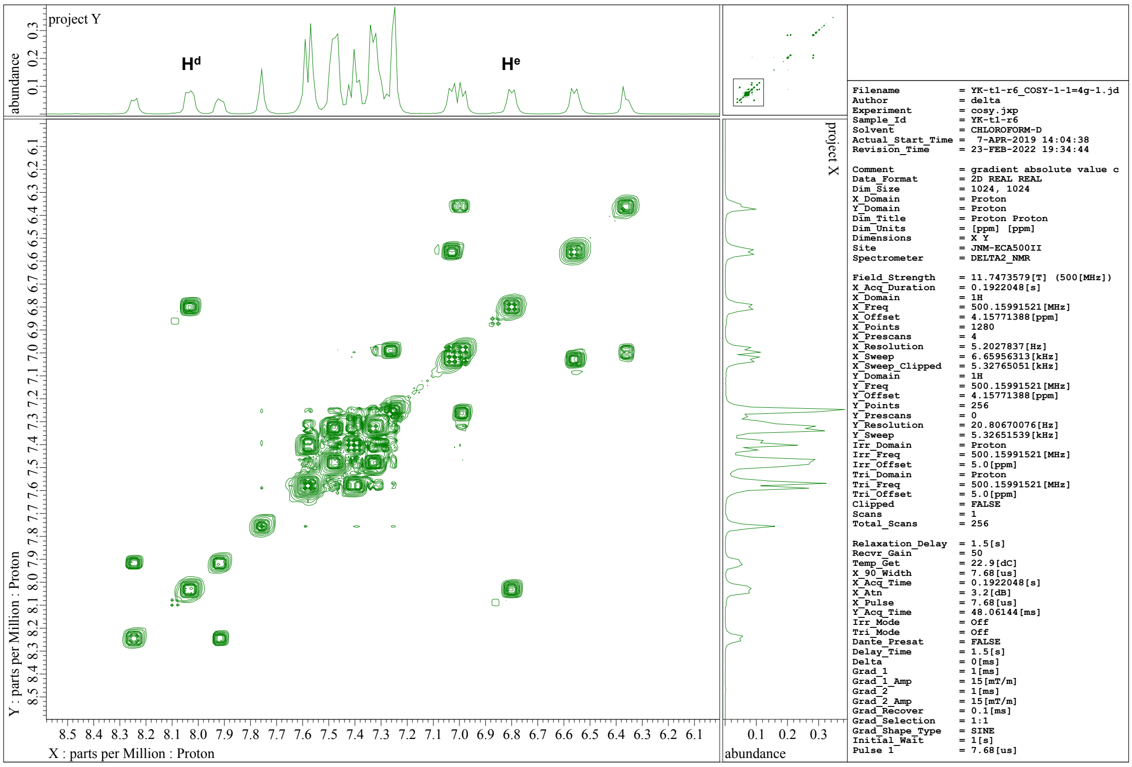

^{1}H-^{1}H COSY NMR Spectrum of **4h** ($CDCl_3$, 400 MHz).

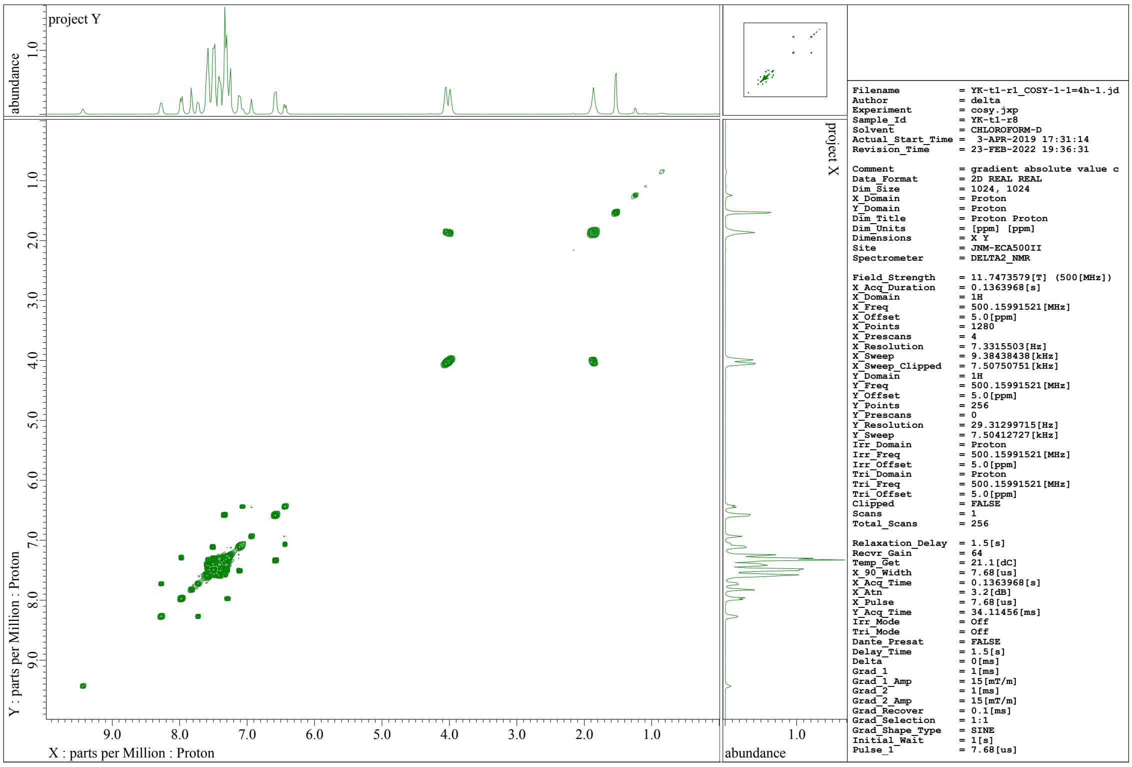

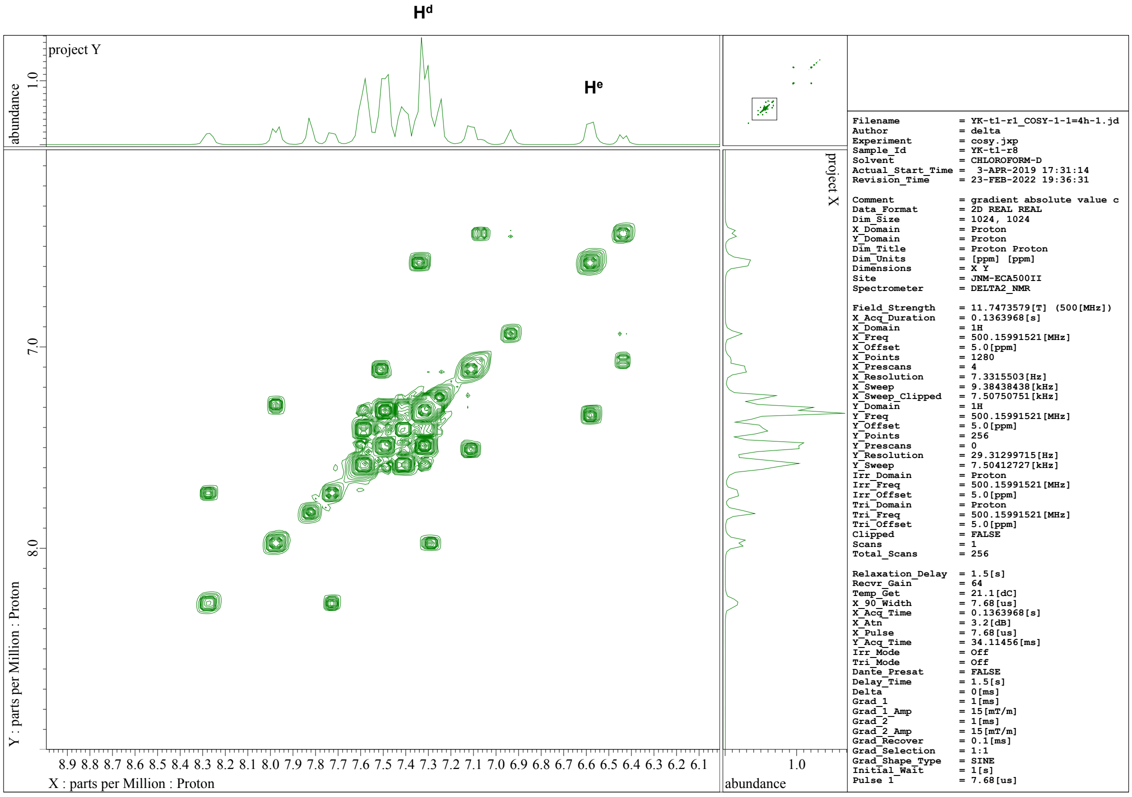

^{1}H-^{1}H EXSY NMR Spectra of **4h** (CD_2Cl_2, 400 MHz, 180 K).

Mixing time 100 ms

srg-yk-4h_180K_100ms

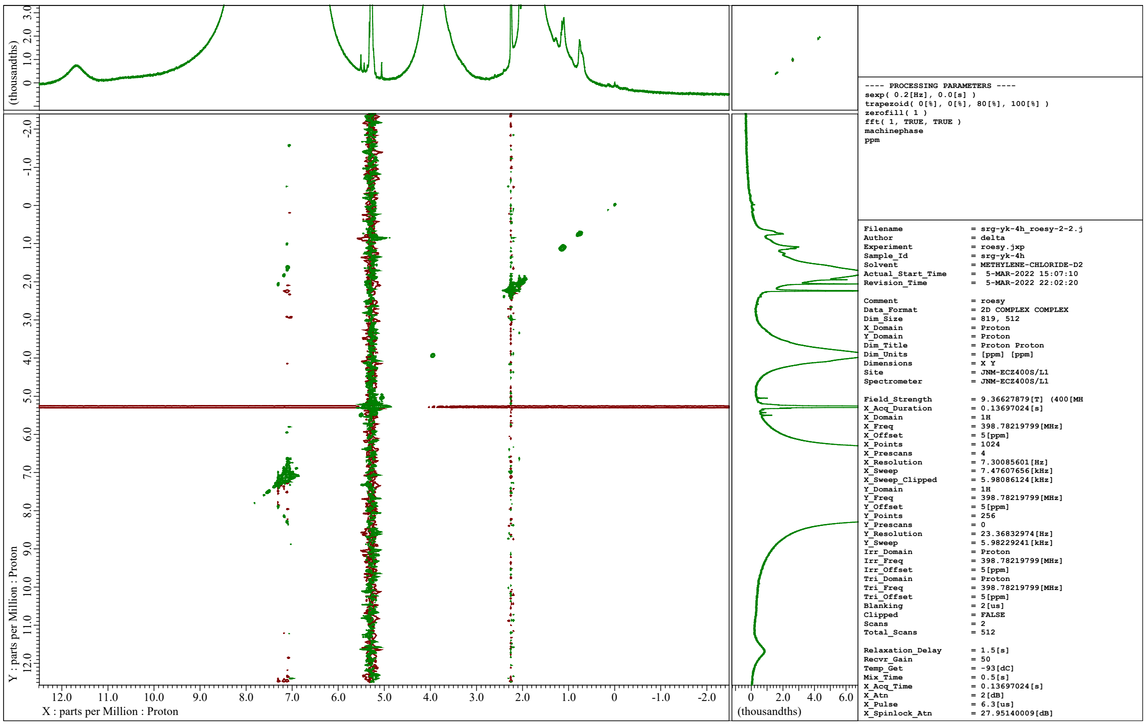

Mixing time 500 ms

srg-yk-4h_180K_500ms

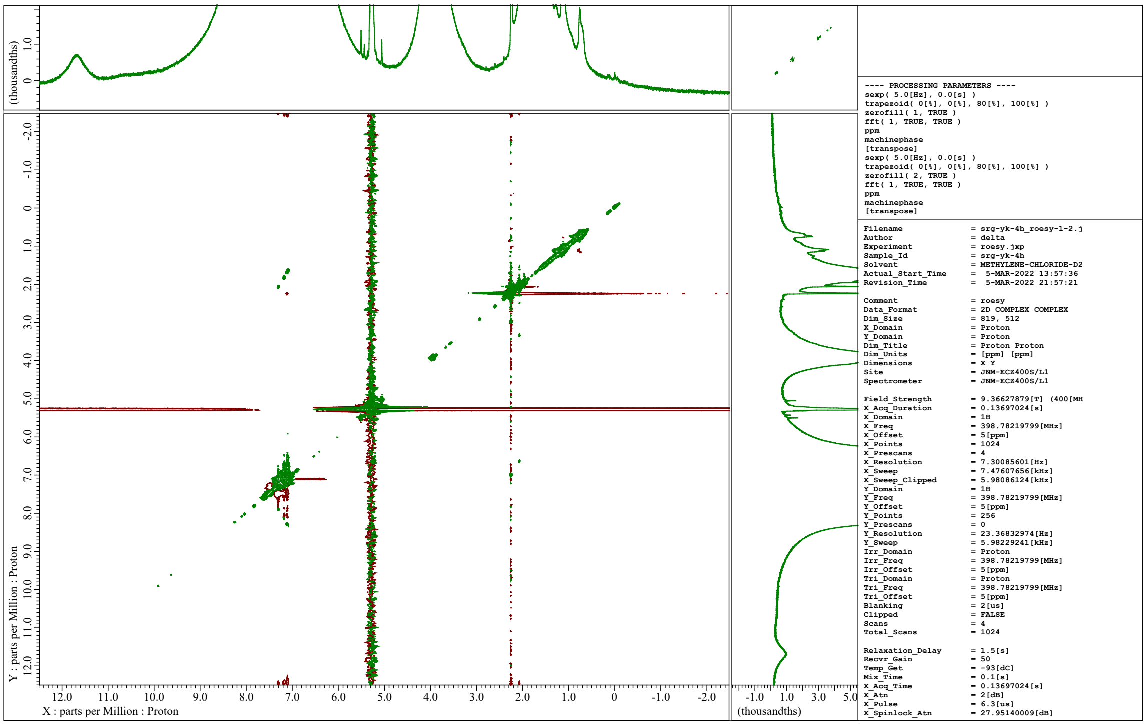

5. Data for X-ray Analysis

5.1 Crystal data and structure refinement for 4a(a)

Table S2. Crystal data and structure refinement for **4a**(a)

	**4a**(a)
CCDC#	2119495
Empirical formula	$C_{148}H_{126}N_2O_4$
Formula weight	1996.50
Temperature (K)	100(2)
Wavelength (Å)	0.71073
Crystal size (mm^3)	0.340 × 0.170 × 0.070
Crystal system	Triclinic
Space group	$P\bar{1}$
Unit cell dimensions, *a* (Å)	13.9579(11)
Unit cell dimensions, *b* (Å)	15.6525(13)
Unit cell dimensions, *c* (Å)	27.196(2)
α, deg	83.0130(10)
β, deg	87.2410(10)
γ, deg	72.9260(10)
Volume (Å^3)	5637.1(8)
Z	2
Density (calculated) (Mg/m^3)	1.176
Absorption coefficient (mm^{-1})	0.069
F(000)	2120
Theta range for data collection, deg	1.487 to 27.486
Index ranges	$-17 \le h \le 18$, $-20 \le k \le 20$, $-34 \le l \le 34$
Reflections collected	63315
Independent reflections	25086 (R_{int} = 0.0297)
R_1	0.0616
wR_2	0.1552
R_1 (all data)	0.0872
wR_2 (all data)	0.1756
Largest diff. peak and hole (e Å^{-3})	0.676 and −0.513
Completeness to theta = 25.242°	99.5 %
Absorption correction	Semi-empirical from equivalents
Max. and min. transmission	0.993 and 0.928
Refinement method	Full-matrix least-squares on F^2
Data / restraints / parameters	25086 / 270 / 1497
Goodness-of-fit on F^2	1.011

5.2 Crystal data and structure refinement for 4a(b)

Table S3. Crystal data and structure refinement for **4a**(b).

	**4a**(b)
CCDC#	2119496
Empirical formula	$C_{129}H_{97}Cl_3N_2O_4$
Formula weight	1845.43
Temperature (K)	100 (2)
Wavelength (Å)	0.71073
Crystal size (mm^3)	0.340 × 0.170 × 0.070
Crystal system	Triclinic
Space group	$P\bar{1}$
Unit cell dimensions, *a* (Å)	14.0475(15)
Unit cell dimensions, *b* (Å)	15.3985(17)
Unit cell dimensions, *c* (Å)	27.528(3)
α, deg	86.6980(10)
β, deg	87.4890(10)
γ, deg	75.4470(10)
Volume (Å^3)	5751.4 (11)
Z	2
Density (calculated) (Mg/m^3)	1.066
Absorption coefficient (mm^{-1})	0.130
F(000)	1936
Theta range for data collection, deg	1.368 to 27.483
Index ranges	$-17 \leq h \leq 18$, $-19 \leq k \leq 19$, $-35 \leq l \leq 35$
Reflections collected	64733
Independent reflections	25625 (R_{int} = 0.0308)
R_1	0.0593
wR_2	0.1462
R_1 (all data)	0.0858
wR_2 (all data)	0.1618
Largest diff. peak and hole (e Å^{-3})	0.404 and −0.355
Completeness to theta = 25.242°	99.4 %
Absorption correction	Semi-empirical from equivalents
Max. and min. transmission	0.991 and 0.900
Refinement method	Full-matrix least-squares on F^2
Data / restraints / parameters	25625 / 0 / 1477
Goodness-of-fit on F^2	1.026

6. Mass Spectra of Rotaxanes 4a-i

Mass Spectrum of **4a**.

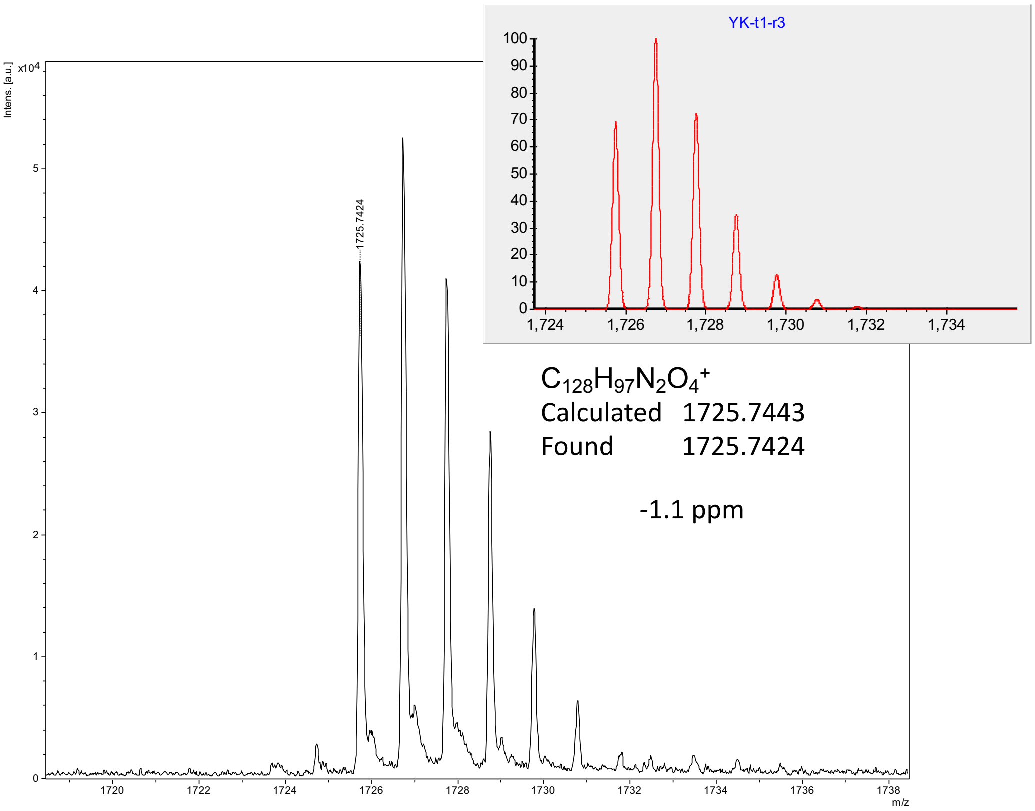

Mass Spectrum of **4b**.

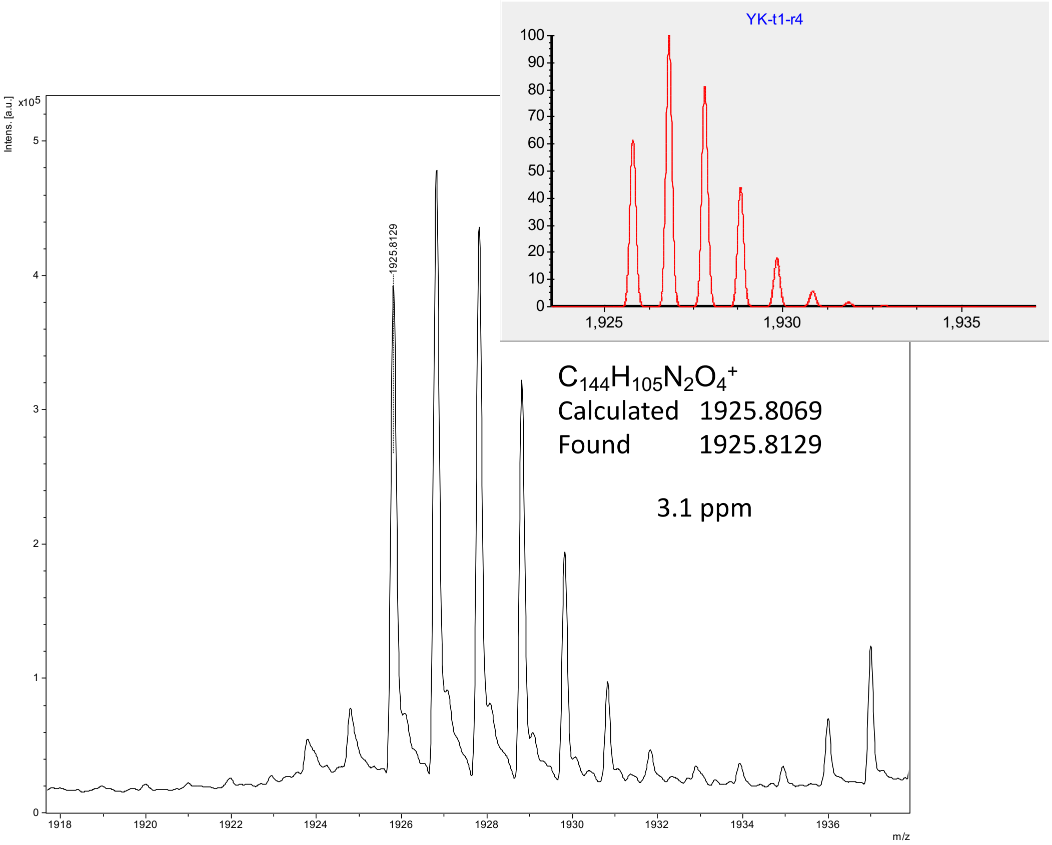

Mass Spectrum of **4c**.

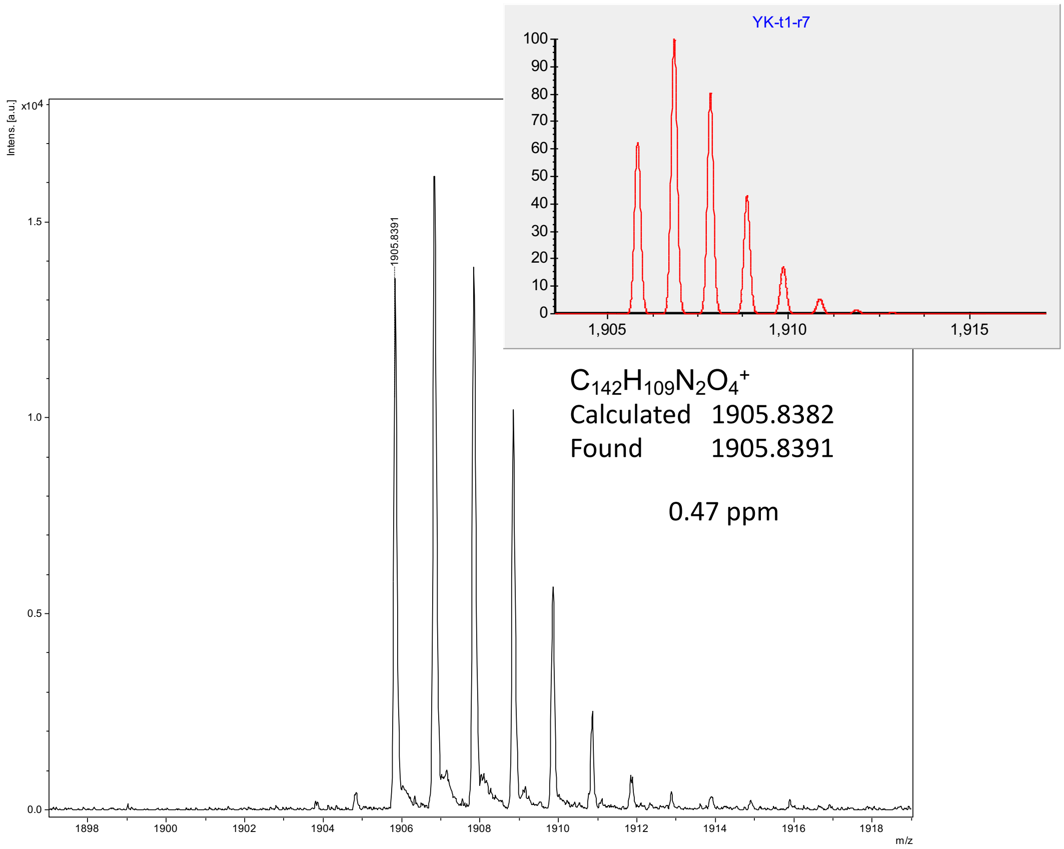

Mass Spectrum of **4d**.

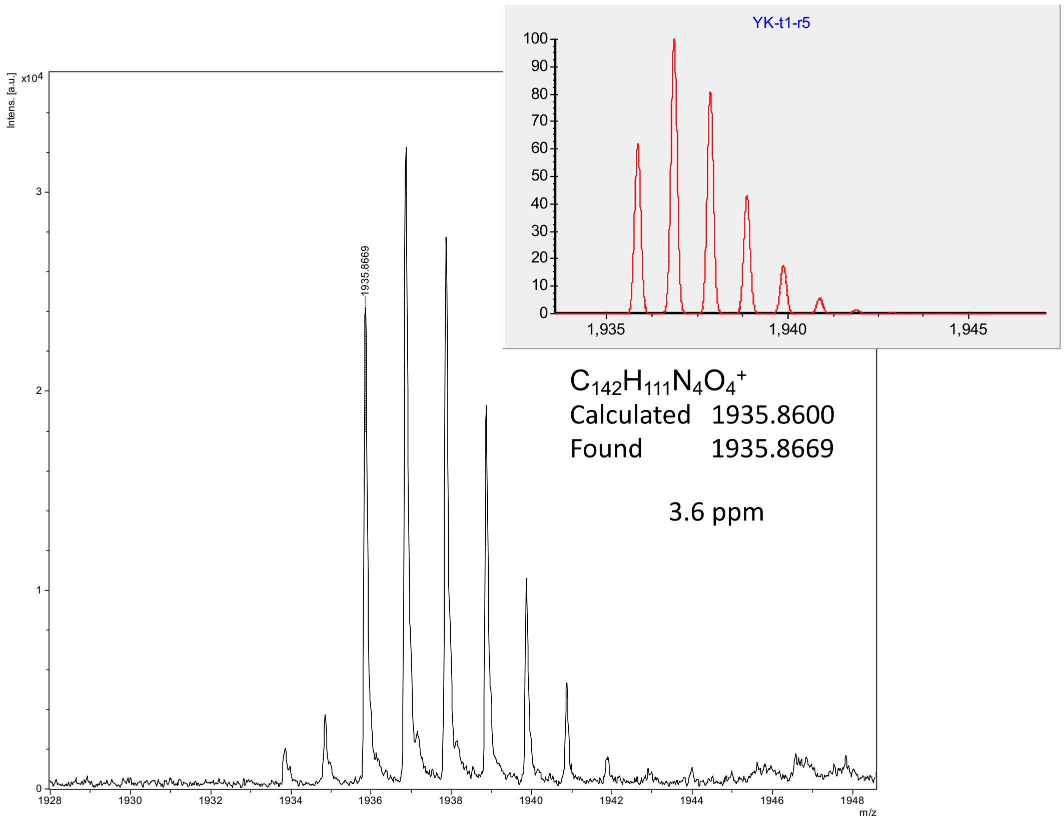

Mass Spectrum of **4e**.

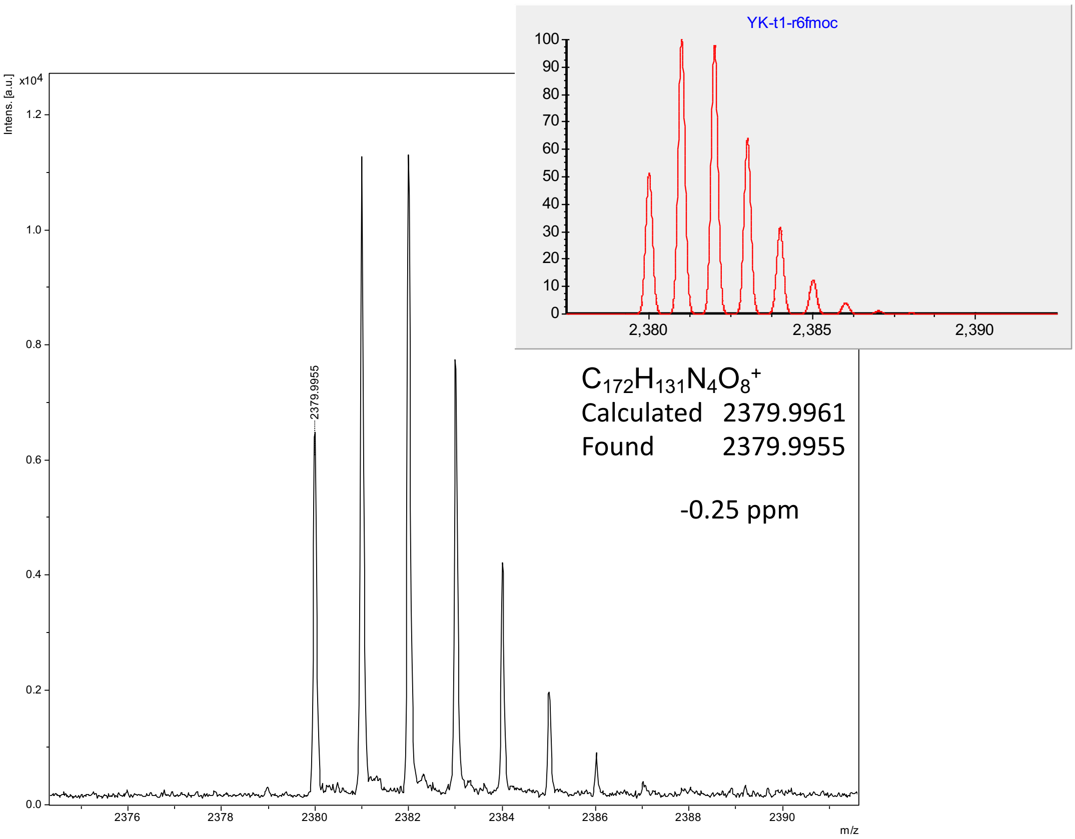

Mass Spectrum of **4f**.

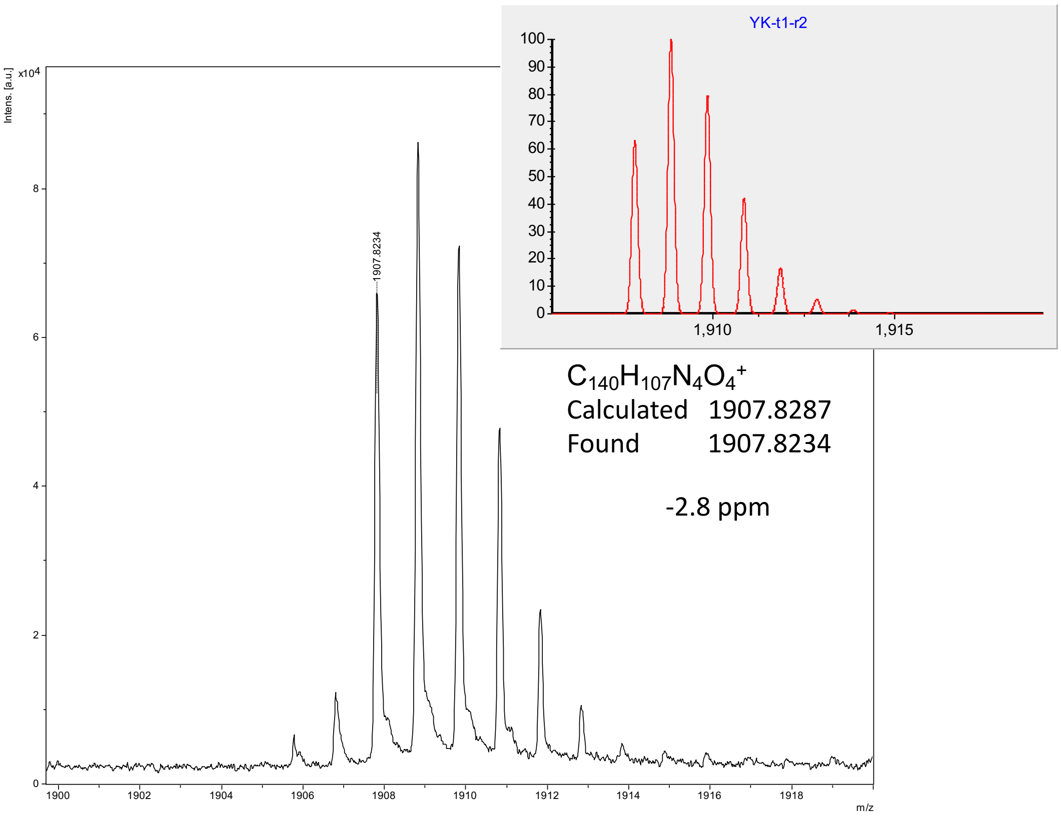

Mass Spectrum of **4g**.

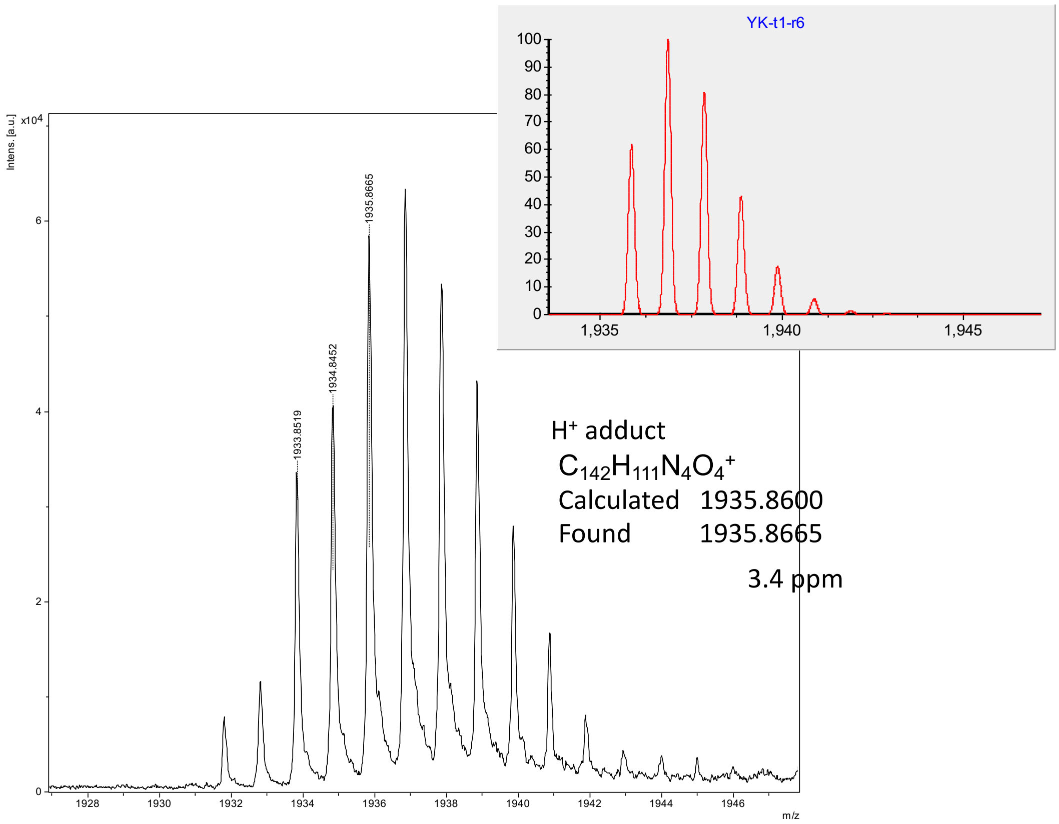

Mass Spectrum of **4h**.

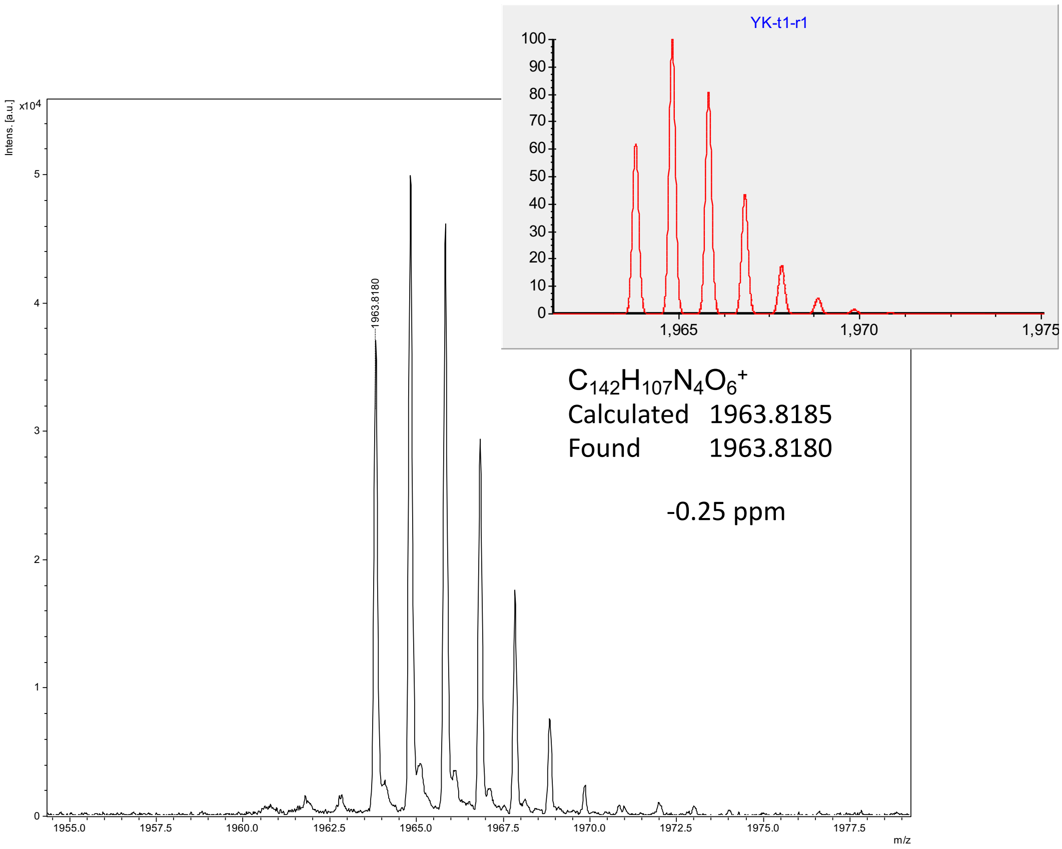

Mass Spectrum of **4i**.

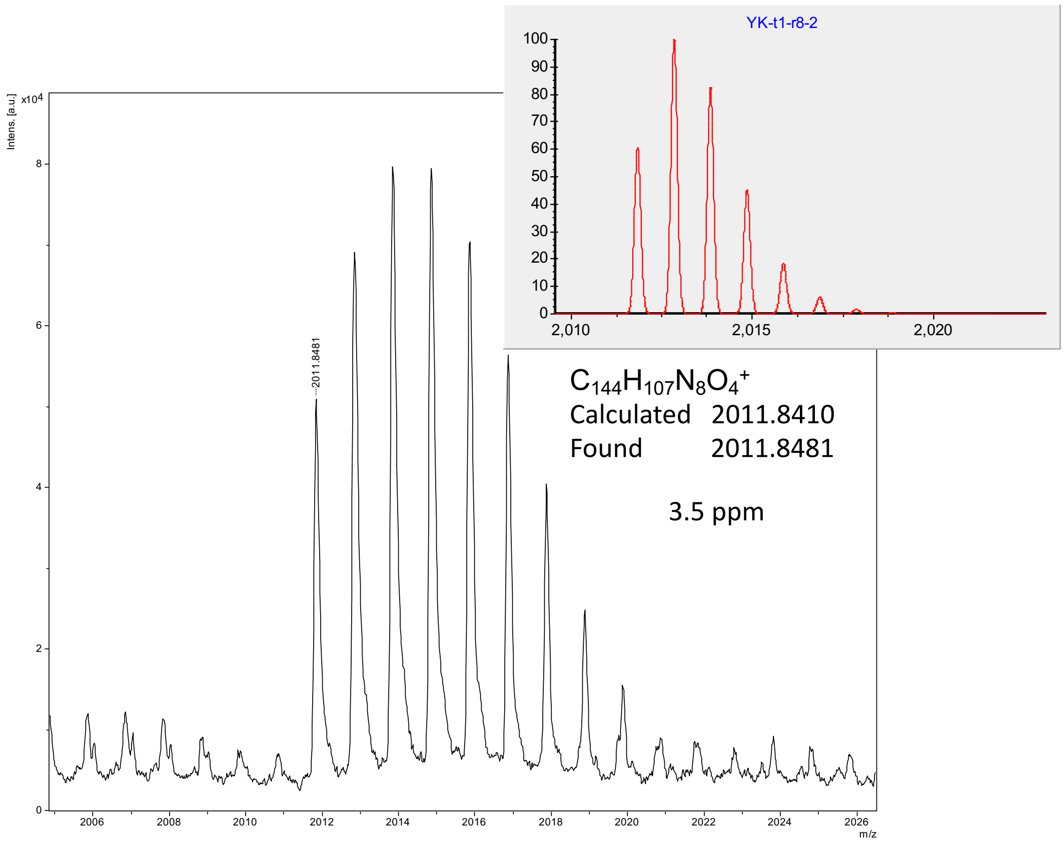

Supplement: Supplementary file 1 — jo2c00086_si_001.pdf [file jo2c00086_si_001.pdf]
